# The Structure of Blood Coagulation Factor XIII Is Adapted to Oxidation

**DOI:** 10.3390/biom10060914

**Published:** 2020-06-17

**Authors:** Alexandra Vasilyeva, Lyubov Yurina, Alexander Shchegolikhin, Maria Indeykina, Anna Bugrova, Alexey Kononikhin, Eugene Nikolaev, Mark Rosenfeld

**Affiliations:** 1 N. M. Emanuel Institute of Biochemical Physics, Russian Academy of Sciences, 119334 Moscow, Russia; lyu.yurina@gmail.com (L.Y.); shchegol@deom.chph.ras.ru (A.S.); mariind@yandex.ru (M.I.); anna.bugrova@gmail.com (A.B.); rosenfeld41@mail.ru (M.R.); 2 V.L. Talrose Institute for Energy Problems of Chemical Physics, N.N. Semenov Federal Center of Chemical Physics, Russian Academy of Sciences, Chernogolovka, 142432 Moscow, Russia; 3 Moscow Institute of Physics and Technology (State University), Dolgoprudny, 141701 Moscow, Russia; 4 Skolkovo Institute of Science and Technology, 121205 Moscow, Russia; ennikolaev@rambler.ru

**Keywords:** blood coagulation factor XIII (FXIII), hypochlorite-induced oxidation, oxidative modifications, reactive oxygen species (ROS), HPLC-MS/MS, structural adaptation

## Abstract

The blood coagulation factor XIII (FXIII) plays a critical role in supporting coagulation and fibrinolysis due to both the covalent crosslinking of fibrin polymers, rendering them resistant to plasmin lysis, and the crosslinking of fibrin to proteins of the fibrinolytic system. The hypochlorite-mediated oxidation of the blood coagulation factor XIII (FXIII) at the different stages of its enzymatic activation is studied for the first time in this paper. The consolidated results obtained with the aid of MS/MS, electrophoresis, and colorimetry demonstrate that in the process of FXIII’s conversion into FXIIIa, the vulnerability of FXIII to hypochlorite-induced oxidation increased as follows: native FXIII < FXIII + Ca^2+^ << FXIII + Ca^2+^/thrombin. The modification sites were detected among all the structural regions of the catalytic FXIII-A subunit, except for the activation peptide, and embraced several sushi domains of the FXIII-B subunit. Oxidized amino acid residues belonging to FXIII-A are surface-exposed residues and can perform an antioxidant role. The regulatory FXIII-B subunits additionally contribute to the antioxidant defense of the catalytic center of the FXIII-A subunits. Taken together, the present data along with the data from previous studies demonstrate that the FXIII proenzyme structure is adapted to oxidation.

## 1. Introduction

FXIII plays a critical role in supporting coagulation and fibrinolysis due to both the covalent crosslinking of fibrin polymers, rendering them resistant to plasmin lysis, and the crosslinking of fibrin to proteins of the fibrinolytic system. FXIII is a proenzyme; its active form (FXIIIa) is a transglutaminase (TG; protein-glutamine: amine γ-glutamyltransferase, EC 2.3.2.13) that forms ε(γ-glutamyl)lysyl crosslinks between two polypeptide chains [1]. FXIIIa crosslinks fibrin γ- and α-chains into γ-chain dimers and α-chain polymers, respectively. The rapid γ-dimer formation is the result of a reciprocal intermolecular bond formation between the Lys406 of one γ-chain and the Gln398/399 residue of another aligning γ-chain. Cross-linking of the α-chains, a much slower process, occurs among multiple glutamine and lysine residues, resulting in α-oligomers and high molecular weight α-polymers. The cross-linking of α-and γ-chains confers final stability to the fibrin clot, providing strength, rigidity, and resistance for fibrinolysis [1].

FXIII is a heterotetrameric proenzyme FXIII-A2B2 that consists of two single-stranded catalytic A subunits (FXIII-A2), each having a molecular weight of 83 kDa, and two identical single-stranded inhibitory/carrier B subunits (FXIII-B2), with a molecular weight of 80 kDa each [1]. All four subunits are held together by non-covalent bonds [1,2].

The catalytic FXIII-A subunit is a single polypeptide chain of 731 residues, including nine cysteine residues, none of which form disulfide bonds [1]. The polypeptide chain forms five distinct structural domains: an N-terminal 37 amino acid long region on the FXIII-A subunit called the activation peptide (FXIII-AP), β-sandwich (Gly38-184Phe), the catalytic core domain (Asn185-515Arg) containing the active site (residues Cys314, His373, and Asp396) and β-barrel 1 (Ser516-628Thr), and β-barrel 2 (Ile629-731Met) [1,2].

The non-catalytic carrier/regulatory FXIII-B subunit is spatially folded into ten sushi domains, each of which is formed by approximately 60 amino acid residues with no free cysteine groups [3]. The structure of either sushi domain is stabilized with two disulfide bonds [1].

The conversion of FXIII-A2B2 into the active form of the enzyme (EC 2.3.2.13) is a multistage process. The first stage involves thrombin-catalyzed proteolytic cleavage of the Arg37-Gly38 bond at the amino-terminus of the FXIII-A subunit leading to the release of the activation peptide [4]. This cleavage leads to the conversion of the FXIII-A2B2 heterotetramer into the FXIII-A′2B2 heterotetramer. The second stage of activation is accompanied by the binding of calcium ions to FXIII-A′ resulting in conformational changes to trigger the dissociation of the FXIII-A′2B2 heterotetramer into the dimer, FXIII-A′2 and FXIII-B2 subunits. During the last stage, in the presence of calcium ions, FXIII-A′2 also undergoes large-scale conformational rearrangements that cause the opening up of the closed zymogenic dimeric form of FXIII-A′2 into an open form of the enzyme, FXIII-A2*, thus giving the substrate access to the catalytic site [4,5,6]. Most recently, novel results have provided evidence that the dimeric molecule FXIII-A2* tends to split into two monomers of FXIII-A*, each one retaining transglutaminase activity [7]. As shown by Singh et al. [8], the transition of the zymogenic heterotetramer to active, open, monomeric FXIII-A* involves the formation of a transient FXIII-AB heterodimer in which the FXIII-A subunits are incompletely saturated with calcium and are still loosely bound to one of the FXIII-B monomers.

The potentially active FXIII subunit is also present in the cytoplasm of platelets, monocytes, monocyte-derived macrophages, dendritic cells, chondrocytes, osteoblasts, and osteocytes. In addition to its contribution to hemostasis, FXIII has multiple extra- and intracellular functions [1].

Proteins are well known to be among the main targets of reactive oxygen species (ROS), which can alter a protein’s structure and functions [9]. Currently, studies focusing on oxidative modifications of FXIII are rare in the literature [10,11,12]. The ozone-induced oxidation of FXIII has previously been demonstrated to affect the transglutaminase activity of FXIIIa, which depends largely on the stage of the conversion of FXIII into FXIIIa, during which protein oxidation is carried out [10]. High-resolution mass spectrometry showed that the numerous oxidation sites (including Met residues) on the catalytic subunit found upon the ozone-induced oxidation of native FXIII plasma, FXIII plasma activated solely with Ca^2+^, and fully activated FXIII plasma (i.e., treated with thrombin and Ca^2+^) differ significantly [11,12]. These findings suggest that in the process of the proenzyme’s conversion into FXIIIa, a portion of the amino acid residues originally buried within the protein globule becomes exposed to the outside, thus becoming a vulnerable target for oxidizing agents.

Unlike ozone, another oxidizing agent, hypochlorite, which was chosen in the current study, is a physiological oxidant. Hypochlorite (produced in vivo through the reaction of hydrogen peroxide and Cl^−^ with aid from the enzyme myeloperoxidase released from neutrophils upon their activation) is considered to be an important pathophysiological factor in oxidative stress [13]. Activated neutrophils can generate up to 425 μM HOCl/h in vitro [14] and are able to damage a number of plasma proteins, such as alpha 1-protease inhibitor [15], α1-antitrypsin [16], human plasminogen [17], proteinase inhibitor alpha-2-macroglobulin [18], human serum albumin and ceruloplasmin [19], and fibrinogen [20].

However, to date, the hypochlorite-induced oxidation of coagulation factor XIII remains completely unexplored. For this purpose, by applying HPLC-MS/MS and biochemical methods, the effect of hypochlorite (HOCl/−OCI) on the chemical and spatial structure of FXIII and its enzymatic activity is studied in this paper for the first time.

The present study aims to provide evidence that (1) the FXIII proenzyme is the least vulnerable target for hypochlorite compared to its activated forms, with FXIII partly activated by Ca^2+^ or FXIII fully activated by Ca^2+^/thrombin; (2) fully activated FXIII is highly vulnerable to hypochlorite action; (3) a portion of the Met and Cys residues on the catalytic subunit is solvent-exposed, allowing them to serve as innate antioxidants; and (4) the regulatory subunits contribute to antioxidant defense of the catalytic domain; the FXIII proenzyme structure is adapted to an oxidative attack.

## 2. Materials and Methods

### 2.1. Isolation of Human FXIII and Fibrinogen

A pool of blood plasma collected from healthy women and men was provided by the Moscow Central Station for Blood Transfusion. FXIII was isolated from pooled human blood plasma by a fractional precipitation procedure with ammonium sulphate and subsequent ion exchange chromatography on a DEAE-ToyoPearl M650 (Tosoh Bioscience, San Francisco, CA, USA) [21] and stored at 4 °C in 50 mM Tris-HCl with 150 mM NaCl and 5 mM EDTA, pH 7.4.

A BioVision Colorimetric FXIIIa Activity Assay Kit (BioVision, Milpitas, CA, USA) was used to determine the specific transglutaminase activity of the activated FXIII isolated from human plasma, normalized by the protein concentration determined at λ = 280 nm, assuming A 1 cm 0.1% = 1.38 for FXIII. Activated FXIII presented a specific activity of 1836 ± 79 Loewy U mg^−1^ (for all FXIIIa samples, the measurements of transglutaminase activity were performed with three biological replicates). Loewy U mL^−1^ is the highest dilution of the enzyme capable of forming an insoluble clot under the conditions described by Loewy et al. [22] (plasma equivalent units per deciliter (PEU/dL), 1 PEU = 108 Loewy U). BioVision’s Factor XIIIa activity assay kit utilizes the transglutaminase activity of FXIIIa to cross-link an amine-containing substrate to the glutamine-containing substrate, resulting in the loss of ammonia, which can be quantitatively measured by a colorimetric assay at 340 nm.

**Figure 1:**
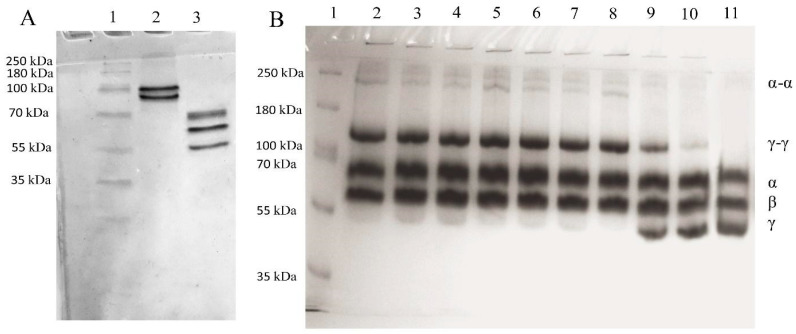
(**A**) Track 1—molecular weight markers: FXIII (track 2) and fibrinogen (track 3) isolated from plasma. The chains were separated by reducing 12% SDS-PAGE; (**B**) electrophoretic patterns of cross-linked fibrin chains in the presence of the different samples of FXIIIa: track 1—molecular weight standards; tracks 2–4—fibrin chains cross-linked by FXIIIa obtained from native proenzyme (FXIII), 50 μM oxFXIII and 150 μM oxFXIII; tracks 5–7—fibrin chains cross-linked by FXIIIa obtained from FXIII + Ca^2+^, 50 μM oxFXIII + Ca^2+^ and 150 μM oxFXIII + Ca^2+^; tracks 8–10—fibrin chains cross-linked by FXIIIa obtained from untreated FXIII + Ca^2+^/Thr; 50 μM oxFXIII + Ca^2+^/Thr and 150 μM oxFXIII + Ca^2+^/Thr; track 11—the fibrinogen Aα (66 kDa), Bβ (54 kDa), and γ (48 kDa) chains are marked. The chains were separated by reducing 6% SDS-PAGE. Approximately 8 μg of fibrinogen was applied to each track. The protein bands were visualized by staining with Coomassie Blue R250.

Fibrinogen was purified from the plasma using glycine precipitation [23]. The protein concentration was measured by Bradford using a protein assay dye reagent concentrate (Bio-Rad, Hercules, CA, USA). The homogeneity of the FXIII and fibrinogen isolated from plasma was evaluated by 12% SDS-PAGE (Figure 1A) in a Laemmli buffer [24]. The proteins were stained with Coomassie Brilliant Blue (R250) dye (20278, Thermo Fisher Scientific, Waltham, MA, USA). The molecular mass of the polypeptide chains of the studied protein was determined using a mixture of the protein markers PageRuler™ Plus Prestained Protein Ladder (Thermo Fisher Scientific, Waltham, MA, USA, 10 to 250 kDa).

### 2.2. Sample Preparation

The transformation of FXIII into FXIIIa in the presence of thrombin (T6884, Sigma-Aldrich, Schnelldorf, Germany) and calcium ions was accomplished as reported in [10]. To inactivate the enzymatic activity of thrombin, hirudin (H7016, Sigma-Aldrich, Schnelldorf, Germany) was added to the thrombin-activated FXIII before incubation with an oxidizer or in a buffer (control sample) [25]. Full thrombin inactivation was tested with the chromogenic substrate S-2238 (Diapharma, West Chester, OH, USA) [26]. Thrombin, previously inactivated by hirudin, was added to the samples of oxidized and native proenzyme and the FXIII samples treated with 5 mM Ca^2+^.

### 2.3. Covalent Linking of Fibrin Chains

Covalent linking of the fibrin chains was catalyzed by different samples of FXIIIa in a 0.05 M Tris–HCl buffer with pH 7.4 containing 0.15 M NaCl and 5 × 10^−3^ M CaCl_2_. In total, 0.05 mL of thrombin solution (0.25 units NIH) and 0.01 mL of FXIIIa were added to 1 mL of fibrinogen solution at a concentration of 2 mg/mL. Fibrin cross-linking was terminated over 45 min. The existence of covalent cross-linking of the polypeptide chains for the stabilized fibrin formed under the action of FXIIIa was detected using a PAGE (6%) of reduced samples in the presence of 1% β-mercaptoethanol.

### 2.4. Exposure of FXIII at the Different Stages of Its Activation to Hypochlorite

FXIII (1 mg/mL) was oxidized by hypochlorite (13440, Sigma-Aldrich, Schnelldorf, Germany) under conditions similar to those described for the hypochlorite-induced oxidation of fibrinogen [20]. Each series of FXIII samples was separated into three portions, which were independently oxidized with 50 or 150 μM HOCl/−OCI and incubated for 1 h at 37 °C. The oxidation reaction was quenched with a 10 molar excess of L-methionine. HOCl/−OCI concentrations were standardized at pH 11 at 292 nm using an extinction coefficient of 350 M^−1^ cm^−1^ [27,28].

The non-oxidized sample was made to have the same composition as the 50 μM sample via the addition of prequenched HOCl/−OCI and methionine. The removal of methionine from the solution was carried out with Amicon Ultra—0.5 mL Centrifugal Filters (10 kDa, UFC501096, Merck, Darmstadt, Germany) according to the manufacturer’s protocol.

A series of FXIII samples with different prehistories were prepared. Their codes are as follows: untreated proenzyme (FXIII); FXIII oxidized by 50 μM or 150 μM HOCl/−OCI (50 μM oxFXIII and 150 μM oxFXIII, respectively); FXIII treated with a 5 mM Ca^2+^ solution (FXIII + Ca^2+^); the samples of FXIII + Ca^2+^ oxidized by 50 μM or 150 μM HOCl/−OCI (50 μM oxFXIII + Ca^2+^ and 150 μM oxFXIII + Ca^2+^, respectively); FXIIIa prepared by the activation of FXIII with thrombin in the presence of 5 mM Ca^2+^ (FXIII + Ca^2+^/Thr) and the samples of FXIII + Ca^2+^/Thr oxidized with 50 μM and 150 μM HOCl/–OCI (50 μM oxFXIII + Ca^2+^/Thr or 150 μM oxFXIII + Ca^2+^/Thr, respectively).

### 2.5. Enzymatic Digestion and HPLC-MS/MS Analysis

The samples were digested with trypsin (V5280, Promeg, Madison, WI, USA) in accordance with the manufacturer’s protocol. Briefly, the protein was hydrolyzed with trypsin at an enzyme/protein ratio of 1:50 (2 μg of trypsin/100 μg of protein) 16 hours at 37 °C. The reaction was stopped by adding formic acid to a final concentration of 0.1%. Water was filtered through a four-stage Milli-Q system (Millipore–Waters, Lane Cove, NSW, Australia) equipped with a 0.2 μm pore size filter.

HPLC-MS/MS experiments were carried out on an Agilent 1100 nano-LC (Agilent Technologies Inc., Santa Clara, CA, USA) coupled to a 7T LTQ-FT Ultra (Thermo, Bremen, Germany) high resolution mass spectrometer. For chromatographic separation, 1 μL of each sample was injected onto a homemade C18 column (75 μm × 12 cm, Reprosil-Pur Basic C18, 3 μm; Dr. Maisch HPLC GmbH, Ammerbuch-Entringen, Germany) using the method described by Ishihama et al. [29]. The following mobile phase was then used: solvent A: 0.1% formic acid in H_2_O and solvent B: acetonitrile. Chromatography was performed with a linear gradient by increasing the relative content of solvent B from 3% to 50% over 60 min. The ion spray voltage was set to 2.3 kV. The mass-spectrometric analysis of the peptide fractions was performed using the Xcalibur software (Thermo Electron, Bremen, Germany) with automatic spectra measurements in the 2-stage mode. In the first stage, the accurate masses of the peptides were measured in the ICR cell in the range *m*/*z* = 300–1600 with a resolution R = 50,000 at *m*/*z* = 400 (the number of ions in the ICR cell was set to 5 × 10^6^). In the second step, the five most intensive peaks from the first stage were subjected to collision-induced dissociation (CID), and the fragment spectra were registered in a linear ion trap (the number of ions in LTQ was set to 3 × 10^4^). After fragmentation, the corresponding parent masses were dynamically excluded from consideration for the next 30 s [30]. For each series of samples, as mentioned above, three biological replicates were carried out, for which measurements were done in triplicate to ensure that the obtained data were reliable and reproducible.

### 2.6. Data Processing

FXIII tryptic peptides were identified by searching the UniProtKB database (UP000005640–9606 HUMAN, Homo sapiens) using the PEAKS Studio software (v. 8.5, Bioinformatics Solutions Inc., Waterloo, ON, Canada) with trypsin as the enzyme and one-sided non-specific cleavage allowed; the mass accuracy for the precursor ion was set to 15 ppm, and the mass accuracy for the MS/MS fragments was set to 0.50 Da. The cut-off false discovery rate (FDR) for the peptides was set to <0.1%. Peptides with a maximum allowance of three variable oxidative post-translation modifications per peptide according to the recommendations are given in [31,32,33]. The list of detected oxidative modifications is provided in Table 1.

## 3. Results

### 3.1. Effect of Oxidation on the Enzymatic Activity of FXIII

As seen from the electrophoresis profile (Figure 1), in the presence of FXIIIa, the polypeptide fibrin chains underwent covalent cross-linking with the formation of γ-γ dimers [34] and polydisperse α-α polymers [35]. The oxidation of native FXIII (tracks 3, 4) had no effect on the subsequent process of the covalent cross-linking of the fibrin network. When FXIII was partially activated by Ca^2+^ (tracks 6, 7), the content of the γ-γ-dimers produced remained unchanged, while the content of accumulated α-α-polymers in the fibrin was slightly decreased. This indicates that the transglutaminase activity of the oxidized protein was almost completely preserved upon incubation with 50 or 150 μM hypochlorite. oxFXIIIa showed decreased enzymatic activity, as evidenced by both the moderate γ-γ dimer content and the trace amounts of α-α-polymers (track 9). The oxidation by 150 μM hypochlorite led to a significant reduction in the γ and α polypeptide chains’ participation in crosslinking (track 10). These data demonstrate that the enzymatic activity depends strongly on the stage of conversion of FXIII into FXIIIa, at which point subsequent oxidation was carried out.

**Figure 2:**
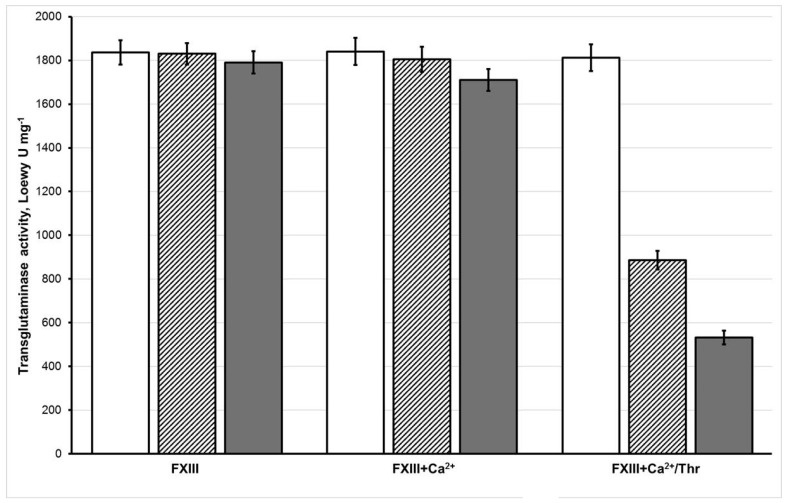
The transglutaminase activity values (Loewy U mg^−1^) for the various FXIII samples subjected to 50 μM (hatched columns) and 150 μM (gray columns) hypochlorite and for non-oxidized FXIII (white columns). The transglutaminase activity is expressed as the means ± S.D., *n* = 6.

The transglutaminase activity values measured by the colorimetric method for various FXIII samples also confirmed this conclusion (Figure 2). For non-oxidized (FXIII) and oxidized (samples 50 μM oxFXIII and 150 μM oxFXIII) of FXIII, the transglutaminase activity values hardly changed, being equal to 1836 ± 55, 1830 ± 49, and 1790 ± 51 Loewy U mg^−1^, respectively. The results indicate that the transglutaminase activity value of FXIII in the sample FXIII + Ca^2+^ was 1841 ± 62 Loewy U mg^−1^, which then decreased to 1804 ± 57 and 1710 ± 41 Loewy U mg^−1^ upon incubation with 50 or 150 μM hypochlorite (samples 50 μM oxFXIII + Ca and 150 μM oxFXIII + Ca^2+^). With oxidized fully activated FXIII (50 μM oxFXIII + Ca^2+^/Thr or 150 μM oxFXIII + Ca^2+^/Thr), the protein suffered significantly under both moderate and severe oxidation. As a result, the transglutaminase activity values decreased from 886 ± 43 to 532 ± 31 Loewy U mg^−1^, while in the control (FXIII + Ca^2+^/Thr sample) these values were equal to 1812 ± 61 Loewy U mg^−1^. Thus, one can conclude that the catalytic XIII-subunits A* in the fully activated FXIII are the most vulnerable targets for the oxidizer.

### 3.2. Analysis of Native and Oxidized FXIII Using High-Resolution Tandem Mass-Spectrometry (LC–MS/MS)

**Figure 3:**
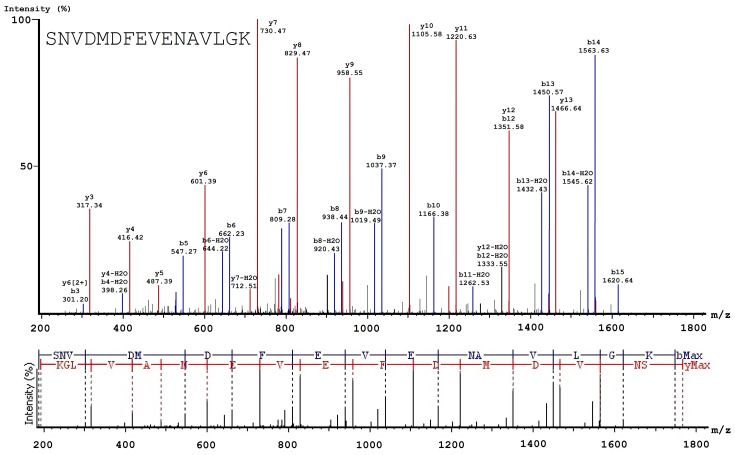
Representative example of the de novo sequencing validating quantification and identification of non-modified (parent) peptides. MS/MS spectrum of the doubly charged ion at *m*/*z* 883.92 for the 150 μM oxFXIII sample identified as SNVDMDFEVENAVLGK. The y1 ion observed at *m*/*z* 147.11 was assigned to the C-terminal residue K and used to determine the remaining amino acid residues by calculating the mass difference of adjacent y-ions.

**Figure 4:**
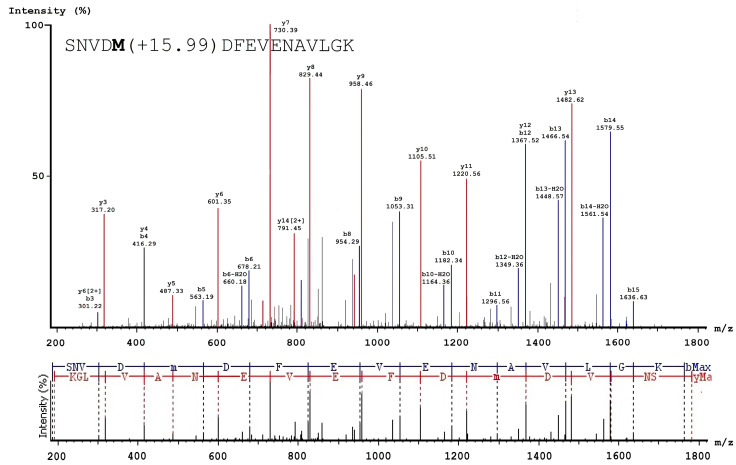
Representative example of the de novo sequencing validating quantification and identification of hypochlorite-modified peptides. The MS/MS spectrum of the doubly charged ion at *m*/*z* 891.92 from the 150 μM oxFXIII sample identified as SNVDM(+15.99)DFEVENAVLGK with an oxidatively modified methionine residue. The y1 ion observed at *m*/*z* 147.11 was assigned to the C-terminal residue K and used to identify the remaining amino acid residues by calculating the mass difference of adjacent y-ions. The tryptic peptide contained the oxidation site corresponding to Met383 in the FXIII-B subunit. The oxidized Met M (+15.99) corresponded to a mass shift of 165.2 Da (149.21 Da for methionine and 15.99 Da due to the addition of one oxygen atom).

After acquisition of the MS/MS spectra (Figure 3 and Figure 4), a list of peptide molecular weights was obtained to determine of hypochlorite-induced changes in the oxidation level of the FXIII molecule. An increase in the content (area) of the oxidized peptide formed, and a decrease in the content of the non-oxidized peptides was observed in the various oxidized FXIII samples (treated with 50 μM and 150 μM HOCl/−OCl) compared to native FXIII at different activation stages (Table S1).

**Figure 5:**
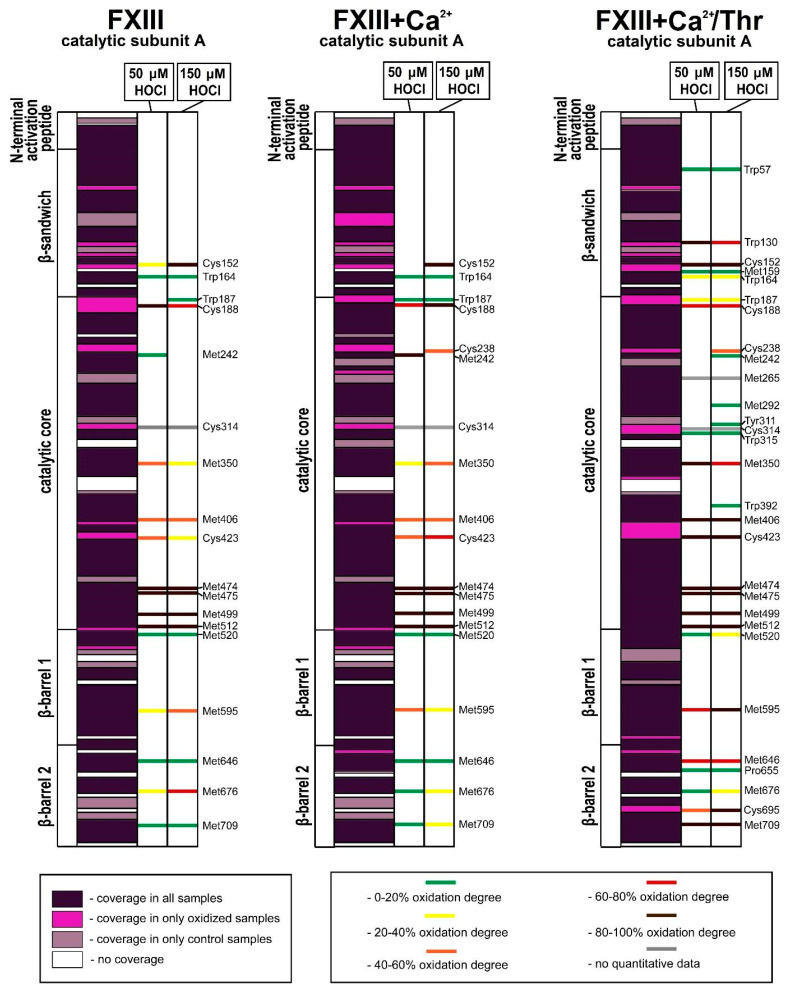
Sequence coverage (left columns) and oxidative modifications in the catalytic FXIII-A subunit of the various FXIII samples subjected to 50 μM (middle columns) and 150 μM hypochlorite (right columns). FXIII, FXIII + Ca^2+^, and FXIII + Ca^2+^/Thr are the samples of the catalytic FXIII-A subunit in the non-activated FXIII, the FXIII activated only by Ca^2+^, and the FXIII activated by the combined action of Ca^2+^ and thrombin, respectively. The label color depends on the overall oxidation degree.

**Figure 6:**
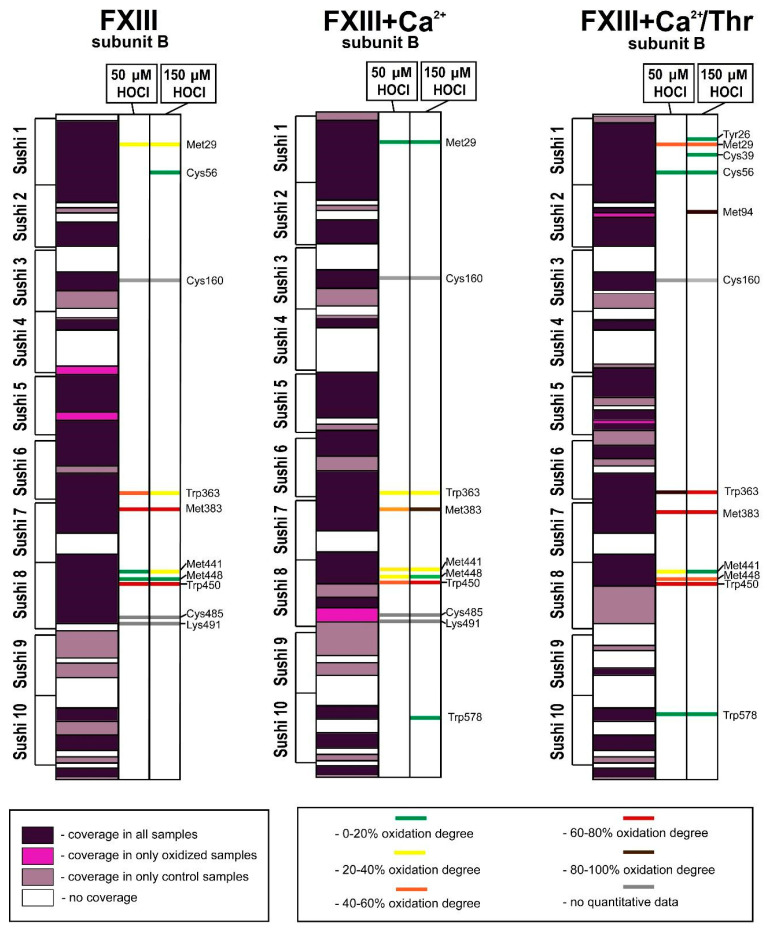
Sequence coverage (left columns) and oxidative modifications in the non-catalytic FXIII-B subunit of the various FXIII samples subjected to 50 μM (middle columns) and 150 μM hypochlorite (right columns). FXIII, FXIII + Ca^2+^, and FXIII + Ca^2+^/Thr are the samples of the regulatory FXIII-B subunit in the non-activated FXIII, the FXIII activated only by Ca^2+^, and the FXIII activated by the combined action of Ca^2+^ and thrombin, respectively. The label color depends on the overall oxidation degree.

Oxidized amino acid residues were detected in all parts of the FXIII-A subunit, except for the N-terminal activation peptide. For the XIII-A subunit, the relative amount of amino acid residues involved in oxidative modifications was equal to 2.8% ± 0.1% in the FXIII oxidized by 50 μM HOCl/−OCI and the FXIII oxidized by 150 μM; for the FXIII + Ca^2+^ oxidized by 50 μM and 150 μM HOCl/−OCI, this value was equal to 2.8% ± 0.1%. For the catalytic subunit A* of the FXIII + Ca^2+^/Thr treated with 50 or 150 μM of HOCl/−OCI, the relative amount of modified amino acid residues was 3.6% ± 0.15% and 4.2% ± 0.1%, respectively. When comparing the oxidative susceptibility of the whole FXIII molecule at different stages of its activation, the enzymatic form of FXIII (FXIII + Ca^2+^/Thr) was most vulnerable to oxidation (Figure 5 and Figure 6). For all samples, the most oxidatively vulnerable structural areas of the FXIII-A subunit were β-barrel−2 and the catalytic core. The mass spectrometry data demonstrate that in the native structure of FXIII, only some of the initial Met residues (Met242, Met350, Met406, Met474, Met475, Met499, Met512, Met520, Met595, Met646, Met676, and Met709) underwent modifications, the oxidation degrees of which strongly differ.

For the FXIII-B subunit, the relative amount of damaged amino acid residues was equal to 1.5% ± 0.1% for the FXIII oxidized by 50 μM HOCl/−OCI and 1.7% ± 0.1% for the FXIII oxidized by 150 μM; 1.7% ± 0.1% for FXIII + Ca^2+^ oxidized by 50 μM and 2.0% ± 0.1% for 150 μM HOCl/−OCl, respectively. For the FXIII-B subunit of FXIII + Ca^2+^/Thr treated with 50 and 150 μM hypochlorite, these values increased up to 2.4% ± 0.1% and 3.8% ± 0.2%, respectively. In the catalytic subunit, the mass spectrometry results indicate that the non-catalytic subunit underwent the greatest alteration in the sample of FXIII fully activated by Ca^2+^/Thr. For the catalytic core, these parameters for the samples of non-activated FXIII, FXIII + Ca^2+^, and FXIII + Ca^2+^/Thr treated with 50 or 150 μM hypochlorite were equal to 3.3% ± 0.1% and 3.4% ± 0.1%; 3.7% ± 0.1% and 3.7% ± 0.1%; and 3.5 %± 0.1% and 5.1% ± 0.1%, respectively.

For the FXIII-B subunit, the most easily oxidizable structural area is the eighth sushi-domain, in which the amount of modified amino acid residues reached its maximum values in the oxidized sample of FXIII-Ca^2+^/Thr (13.0% ± 0.1% for 50 μM HOCl/−OCl and 13.0% ± 0.1% for 150 μM HOCl/−OCl, respectively; Figure 5 and Figure 6).

## 4. Discussion

A comparison of the mass-spectrometry data obtained for the native and hypochlorite-oxidized samples of FXIII convincingly demonstrates that the catalytic subunit undergoes significant chemical alterations. Since, in proteins, Cys and Met are the primary targets for hypochlorite [36,37], these residues in the catalytic subunit were the main residues subjected to modifications. The HPLC-MS/MS data indicate that of the 19 Met residues present in the primary structure of the protein, 14 were identified in the untreated sample of FXIII, while only five Met residues, Met136, Met247, Met380, Met679, and Met 731, were not covered. For the FXIII sample untreated with HOCl/−OCl, the 12 Met residues proved to be oxidatively damaged to varying degrees. These residues belong to the catalytic core domain (Met242, Met350, Met406, Met474, Met475, Met499, and Met512), the β-barrel 1 domain (Met520 and Met595), and the β-barrel 2 domain (Met646, Met676, and Met709). That the above methionines exhibit increased susceptibility to oxidation suggests that they are surface-exposed residues [38]. Exposed methionine residues are oxidized with negligible effects on biological activity and serve as a pool of targets to scavenge ROS and protect functionally crucial residues [38,39]. The other two non-oxidized Met residues, Met159 and Met265, are likely buried within the protein globule and become exposed to the surface during conformational rearrangements of the molecule due to its activation.

Methionine oxidation in proteins is one of the most commonly occurring oxidative modifications in proteins due to the special susceptibility of methionine to oxidative conditions. Accordingly, this observation indicates that methionine residues act like intramolecular antioxidants and protect other amino acids from oxidation. In other words, methionine residues embedded in the primary structure of plasma proteins may serve as innate ROS interceptors [39]. The ability of exposed methionines to scavenge different forms of ROS to protect crucial amino acid residues from being oxidized was previously demonstrated for some plasma proteins [40,41,42,43]. Hence, that the FXIII-A subunit is exceedingly plentiful in Met residues, many of which are likely to be located on the protein surface and could contribute to primary antioxidant defenses against oxidant-induced injury.

The results of electrophoresis and colorimetry demonstrate that the transglutaminase activity of oxidation-modified FXIIIa decreased, strongly depending both on the oxidant amount chosen and the stage of FXIII conversion into FXIIIa in which oxidation was carried out (Figure 1 and Figure 2). The proenzyme exhibited the least vulnerability to oxidation. The proenzyme treated with 150 μM HOCl/−OCl completely retained its enzymatic activity inherent to the unaffected protein, while the FXIIIa treated with 50 μM HOCl/−OCl demonstrated drastically reduced enzymatic activity. These results are completely consistent with those obtained earlier for the ozone-induced oxidation of FXIII (Figure S1) [10].

When comparing the results of mass spectrometry with the results obtained by electrophoresis and colorimetry, none of the oxidized amino acids residues found in the proenzyme treated with HOCl/−OCl are functionally significant for the enzyme, whose activity does not decrease in this group of samples regardless of the amount of hypochlorite used. However, the degree of damage to the residues correlates with the amount of oxidizing agent.

Another interesting observation is that an increase in the degree of damage to amino acid residues belonging to one group of samples occurs not only with growth in the amount of oxidizing agent but also for different groups of samples subjected to the same amount of hypochlorite. This is clearly a consequence of the process of the conversion of the proenzyme into the enzyme, during which the catalytic FXIII-A subunit becomes an increasingly more vulnerable target for oxidation.

**Figure 7:**
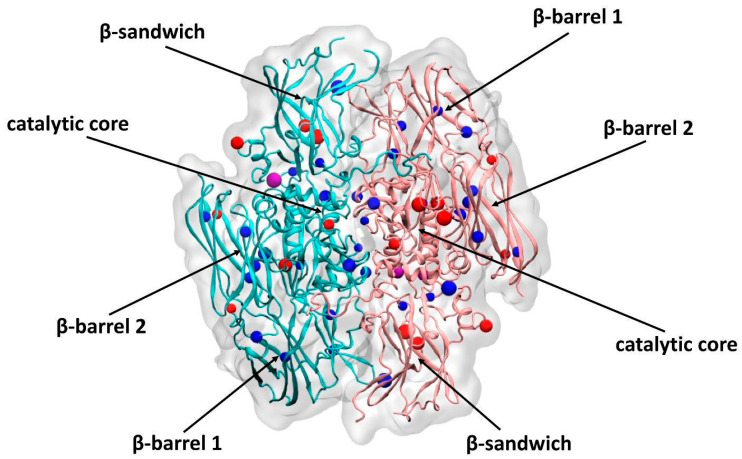
The presentation of the catalytic FXIII-subunit dimer crystal structure (PDB ID: 1F13) with the oxidative modifications detected in the oxidized samples. The left (blue) and right (pink) monomers represent the catalytic subunit structure of FXIII treated with 50 μM and 150 μM hypochlorite, respectively. The amino acid residues damaged by hypochlorite in the 50 μM oxFXIII and 150 μM oxFXIII samples, respectively, are drawn in blue balls; the residues damaged in the 50 μM oxFXIII + Ca^2+^ and 150 μM oxFXIII + Ca^2+^ samples are violet; the residues damaged in the 50 μM oxFXIII + Ca^2+^/Thr or 150 μM oxFXIII + Ca^2+^/Thr samples are red. The size of each ball depends on the molecular mass of the modified residue (the oxidation degree of each hypochlorite-modified residue is shown in Table S2). The image was made using the VMD1.9.3 (University of Illinois at Urbana, IL, USA; Champaign, IL, USA) program.

As shown by the mass-spectrometry data, the crucial residues, Tyr560 and Trp279, remained in their native form in all of the samples. However, since FXIIIa dramatically reduced its activity when exposed to hypochlorite, the effect was driven by damage to the amino acid residues in this group of samples (Trp130 and Cys695), as well as Tyr311 and Trp315, located in the immediate vicinity of the catalytic center of the enzyme (Figure 7). Due to a lack of knowledge regarding the functionality of each of these residues, it can be assumed that they are endowed with certain functions, which, to one degree or another, can affect the activity of the enzyme.

Interestingly, the human α2-macroglobulin treated with hypochlorite at a molar protein/ratio close to that used in the present study, completely lost its antiproteolytic activity [40]. Although the quaternary structures of the proteins differ, the various effects of hypochlorite on the proteins suggest a high antioxidant capacity of the FXIII proenzyme structure.

In the oxidized samples of FXIII, FXIII + Ca^2+^, and FXIII + Ca^2+^/Thr, some of Cys residues are also targets for the oxidant. The Cys238 residues in FXIII-A2′B2 and FXIIIa are involved in the oxidative damage only with 150 μM of the oxidant. Cys238 is believed to be the surface-exposed residue in the fully activated molecule [1]. Nevertheless, that the residue remains unaffected by moderate oxidation contradicts this finding. Most likely, when FXIII is activated, the residue initially localized within the protein core begins to migrate to the hydrophilic region, while still being partly spatially inaccessible. The Cys423 residue is not covered in the control samples of FXIII and FXIII + Ca^2+^/Thr. Since the Cys423 residue is covered and non-oxidized for the control FXIII + Ca^2+^ sample, this residue possibly does not undergo oxidative alterations in the control FXIII sample. Upon both mildly and strongly induced oxidation, the Cys423 residue is oxidatively modified in all the oxidized samples. Furthermore, the oxidation degree of the Cys423 residue (like Met residues) mainly trends toward a more significant modification. In many cases, the unwanted oxidation of Cys may result in oxidative damage thereby modifying protein function [44]. As mentioned above, none of the oxidized amino acid residues found in the oxidized proenzyme are functionally significant for the enzyme. Consequently, the Cys152, Cys188, and Cys423 located on the FXIII-A subunits may serve as antioxidant residues that, together with the Met residues, are capable of protecting the catalytic protein center against oxidation.

Likely, the FXIII-B subunits also contribute to the antioxidant defense of the catalytic subunit. In their native conformations the interactions between all four subunits of FXIII are well known to provide support for the most compact, globular structure of the multimeric protein complex. As a result, the entrance to the catalytic center is closed to any substances, including small molecules [4], such as HOCl/−OCI.

The mass-spectrometry data indicate the significant contribution of sulfur-containing residues of methionines and cystines to the total amount of oxidation sites emerging in the XIII-B2 subunits due to hypochlorite-induced oxidation of FXIII. The abundant cystine residues present in FXIII-B [3] have high reactivity to hypochlorite [44,45]. It is important to note that, like the catalytic subunit, the vulnerability of the regulatory subunit to induced oxidation increases with FXIII activation in the following order: native FXIII < FXIII + Ca^2+^ < FXIII + Ca^2+^/Thr (Figure 6). Additionally, in the last stage of FXIII activation, the new oxidation sites (Tyr26, Cys39, and Met94) that arise in FXIII-B2 provide insight into the conformational changes in FXIII-B2 that occur upon FXIII dissociation.

A recent report by Protopopova et al. [7] suggests partial wrapping of the B subunits around the central core of FXIII-A2, thus additionally protecting the catalytic center from being involved in oxidation. The protective functions of the FXIII-B subunits (as antioxidants) might explain why their deficiency leads to a dramatic reduction in the FXIII-A concentration in plasma—due to its instability [4,46]. Finally, as recently shown, the cell-derived FXIII composed only of the two catalytic FXIII-subunits (cFXIII), subjected to oxidation under conditions similar to those of the blood coagulation factor XIII, exhibited profound changes in the spatial structure of their proteins, which resulted in a much greater loss of transglutaminase activity compared to that of oxidized FXIIIa [47].

Thus, the tight packing of both the tetrameric FXIII structure and the exposed Met and Cys residues on the FXIII-A subunits, as well as the protective function of the FXIII-B subunits, could be the main factors underlying the high tolerance of the protein to oxidizing agents.

It is worth noting that the results of the current study were obtained using FXIII alone, while in the bloodstream, FXIII circulates together with other proteins that are present in greater abundance than FXIII. In one way or another, each of the blood plasma proteins is able to intercept the ROS, which is likely to limit FXIII oxidation under healthy conditions. Most recently, it was shown in plasma samples collected from healthy volunteers that 500 HOCl/−OCl μM does not alter the enzymatic activity of FXIII [48,49]. In this regard, it should be emphasized that the authors added the oxidizing agent to blood plasma in which FXIII was present in an inactive form. As shown in the current study, the proenzyme has maximum resistance to the action of hypochlorite, and it seems reasonable to suppose that only these amino acid residues could undergo oxidation (for example, Met residues), which is not vital.

However, at the inflammation site, the local HOCl/−OCl level can reach millimolar concentrations [50]. In the blood plasma, FXIII is known to be non-covalently associated with fibrinogen [51,52], which, by binding to the integrin alphaMbeta2 (Mac−1) expressed on activated leukocytes, provides a key link between thrombosis and inflammation [53,54,55]. Therefore, the effect of neutrophil oxidants on FXIII is likely local.

For this reason, future studies should aim to identify the sites of oxidative modifications employing FXIII extracted from the blood plasma of patients suffering thrombotic disorders associated with inflammation and the production of oxidants.

## 5. Conclusions

To date, the ability of plasma proteins to function in a ROS-generating environment remains unclear and is still considered one of the most important factors of protein oxidation. For the first time, the structural and functional damages to blood coagulation factor XIII treated with hypochlorite (recognized the main oxidizer in blood plasma) were analyzed in this study. The present study provides experimental data demonstrating that (1) the hypochlorite-induced oxidation of FXIII damages the chemical structure of the protein, as well as the transglutaminase activity that is strongly dependent upon the stage of FXIII’s conversion into FXIIIa, at which point oxidation was carried out; (2) the modification sites are distributed among all the structural regions of the catalytic subunit FXIII-A, except for the activation peptide and the embrace of some of sushi domains of the inhibitory/carrier FXIII-B subunits; (3) FXIII is less vulnerable to oxidation than the other investigated samples of FXIII, such as the FXIII activated by only Ca^2+^ or fully activated by Ca^2+^/thrombin; and (4) it is supposed that the structure of FXIII is adapted to detrimental ROS action due to the following reasons: The globular structure of the multimeric protein complex is more compact than the activated forms of FXIII; protein is exceedingly plentiful in methionine and cystine residues, many of which are likely to be located on the protein surface. Such surface-exposed residues are oxidized with negligible effects on biological activity and are capable of scavenging ROS to protect other crucial residues against ROS toxicity; the FXIII-B subunits, we believe, can act as antioxidant structures to mitigate the toxic effects of the reactive species on the catalytic subunits.

Taken together, the current results along with data from previous studies suggest that FXIII can be provided the antioxidant capacity necessary to withstand ROS action. The ability of proteins circulating in the blood plasma to engage in antioxidant self-defense may be among the leading factors that play an important role in maintaining the protein’s structure and stability upon oxidation.

## Figures and Tables

**Table 1:** Detected oxidative modifications and their numbers for the samples of FXIII oxidized by 50 and 150 μM HOCl/–OCI in the FXIII subunits.

Δm (Monoisotopic)	Modification	Composition	oxFXIII		oxFXIII + Ca^2+^		oxFXIII + Ca^2+^/Thr	
			50 μM HOCl/−OCl	150 μM HOCl/–OCI	50 μM HOCl/–OCl	150 μM HOCl/–OCl	50 μM HOCl/–OCl	150 μM HOCl/–OCl
+15.994915	Oxidation	O	19	20	19	19	26	28
+31.989828	Dioxidation	O (2)			1		1	1
+47.984744	Trioxidation	O (3)	5	5	3	5	5	7
+27.994915	Formylation	C O	-	-	-	-	1	1
−48.003371	Prompt loss of side chain from oxidized Methionine	H (−4) C (−1) S (−1)	1	1	2	2	2	3
+3.994915	Tryptophan oxidation to kynurenin	C(−1) O	-	-	–	2	1	2
+13.979265	Tryptophan oxidation to oxolactone	H(−2) O	2	2	2	3	4	4
+19.989829	Tryptophan oxidation to hydroxykynurenin	C(−1) O(2)	1	-	1	-	-	-
+33.961028	Chlorination of tyrosine residues	H(−1) Cl	-	-	-	-	-	2
+14.9632	Lysine oxidation to a-aminoadipic acid	H(−3) N(−1) O(2)	1	1	1	1	-	-
−33.987721	Dehydroalanine (from Cysteine)	H(−2) S(−1)	2	2	2	3	1	3
−27.994915	Pyrrolidone from Proline	C(−1) O(−1)	-	-	-	-	1	1

## Supplementary Materials

The following are available online at https://www.mdpi.com/2218-273X/10/6/914/s1, Figure S1: Presentation of the catalytic FXIII-subunit dimer crystal structure (PDB ID: 1F13) with the oxidative modifications detected in the oxidized samples: FXIII-A2B2 (A); FXIII-A2B2 + Ca^2+^ (B); and FXIII-A2B2 + Ca^2+^/thrombin (C). The left monomer represents the catalytic subunit structure of FXIII treated with 150 μM hypochlorite. The covered sequences of the structural elements are colored blue; the non-covered sequences are not shown. The amino acid residues damaged by hypochlorite are drawn in red balls. The right monomer is the catalytic subunit structure of FXIII (colored pink) treated with 1 µM of ozone per 1 mg of the protein. The amino acid residues damaged by ozone are shown in blue balls. The purple balls represent the positions of shared residues involved in oxidative damage when the protein was treated with ozone or hypochlorite. Table S1: The list of identified peptides containing oxidatively modified residues in the FXIII subunits. Various modifications of amino acid residues characterized by a certain mass are shown. Modifications with a low (<100) Ascore value, as well as modifications with quantitative values of less than 1%, are not considered in Table S1. The oxidation degree (%) of the modified peptides normalized to the total areas of the peaks of unmodified and modified peptides (estimated modification levels) for various types of modifications of specific amino acid residues for the control (non-oxidized FXIII) and samples of FXIII oxidized by 50 and 150 μM HOCl/−OCl. For each series of samples, as mentioned above, three biological replicates were carried out, for which measurements were done in triplicate to ensure that the obtained data were reliable and reproducible. Table S2: Exported data on peptides and the modifications found (using Peaks Studio 8.5).
