# Supplementary material for: The Structure of Blood Coagulation Factor XIII Is Adapted to Oxidation
Source: Biomolecules. 2020 Jun 17;10(6):914. doi: 10.3390/biom10060914 (PMC7355775; doi:10.3390/biom10060914)
Supplement: Supplementary file 1 [file biomolecules-10-00914-s001.pdf]

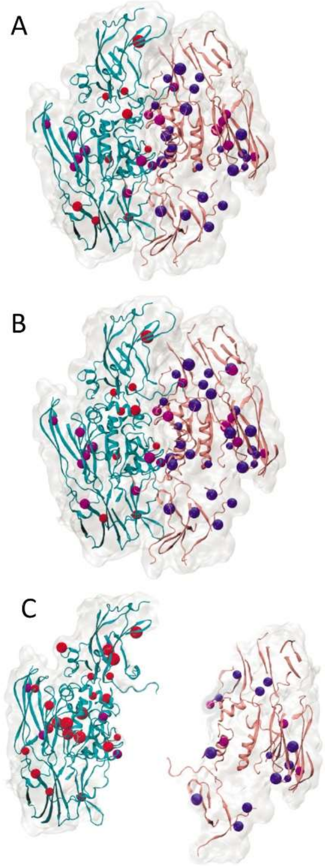

Figure S1. The presentation of the catalytic FXIII-subunit dimer crystal structure (PDB ID: 1F13) with the oxidative modifications detected in the oxidized samples: FXIII-A_2B_2 (A); FXIII-A_2B_2 + Ca^{2+} (B); and FXIII-A_2B_2 + Ca^{2+}/thrombin (C). The left monomer represents the catalytic subunit structure of FXIII treated with 150 μM hypochlorite. The covered sequences of the structural elements are colored blue; the non-covered sequences are not shown. The amino acid residues damaged by hypochlorite are drawn in red balls. The right monomer is the catalytic subunit structure of FXIII (colored pink) treated with 1μM of ozone per 1 mg of the protein. The amino acid residues damaged by ozone are shown in blue balls. The purple balls represent the positions of shared residues involved in oxidative damage when the protein was treated with ozone or hypochlorite.

Table S1. The list of identified peptides containing oxidatively modified residues in the FXIII subunits. Various modifications of amino acid residues characterized by a certain mass are shown. Modifications with a low (<100) Ascore value, as well as modifications having quantitative values of less than 1%, are not considered in Table S1. The oxidation degree (%) of the modified peptides normalized to the total areas of the peaks of unmodified and modified peptides (estimated modification levels). For each series of samples, as above mentioned, three biological replicates were carried out, for each of which the measurements were done in triplicate to ensure that the obtained data are reliable and reproducible.

**Oxidized peptides**	**Oxidize d amino acid residue**	**Modificatio n type**	**Oxidation degree, %**									**Structural part**
			FXIII	50 μM oxFXI II	150 μM oxFXI II	FXIII + Ca^{2+}	50 μM oxFXI II + Ca^{2+}	150 μM oxFXI II + Ca^{2+}	FXIII + Ca^{2+}/T hr	50 μM FXIII + Ca^{2+}/T hr	150 μM FXIII + Ca^{2+}/ Thr	
**Chain A**												
K.ER**W(+13.98)**DTNKVDHHTDKYENNK.L	**Trp 57**	Tryptophan oxidation to oxolactone	n/m	n/m	n/m	n/m	n/m	n/m	n/m	1-20%	1-20%	**β-sandwich**
K.ER**W(+3.99)**DTNK(+42.01)VDHHTDKYENNK.L		Tryptophan oxidation to kynurenin	n/m	n/m	n/m	n/m	n/m	n/m	n/m		1-20%	
K.GTYIPVPIVSELQSGK**W(+15.99)**GAK.I<br>I.PVPIVSELQSGK**W(+15.99)**GAK.I<br>V.PIVSELQSGK**W(+15.99)**GAK.I	**Trp 130**	Oxidation	n/c	n/c	n/m	n/c	n/c	n/m	n/c	80-100%	60-80%	
R.LSIQSSPK**C(+47.98)**IVGK.F	**Cys 152**	Trioxidation	n/c	40-60%	80-100%	n/c	n/c	80-100%	n/c	n/c	60-80%	
R.LSIQSSP(+31.99)K**C(+15.99)**IVGK.F<br>R.LSIQSSPK(+31.99)**C(+15.99)**IVGK.F<br>R.LSIQSSP(+15.99)K**C(+15.99)**IVGK.F		Oxidation	n/c	n/m	n/m	n/c	n/c	n/m	n/c	20-40%	1-20%	
R.**M(+15.99)**YVAVWTPYGVLR.T<br>R.**M(+15.99)**YVAVW(+15.99)TPYGVLR.T	**Met 159**	Oxidation	n/m	n/m	n/m	n/m	n/m	n/m	n/m	1-20%	1-20%	
R.MYVAV**W(+15.99)**TPYGVLR.T<br>R.M(+15.99)YVAV**W(+15.99)**TPYGVLR.T<br>R.MY(-2.02)VAV**W(+15.99)**TPYGVLR.T<br>R.MY(+15.99)VAV**W(+15.99)**TPYGVLR.T	**Trp 164**	Oxidation	n/m	1-20%	1-20%	n/m	1-20%	1-20%	n/m	1-20%	20-40%	
R.NPETDTYILFNP(+31.99)**W(+15.99)**CEDDAVYLDNEK.E	**Trp 187**	Oxidation	n/c	n/m	1-20%	n/c	1-20%	1-20%	n/c	20-40%	20-	**Catalytic**

R.NPETDTYILFNP(+31.99)**W(+15.99)**CEDDAVYLDNEKER.E<br>R.NPETDTYILFNP(+15.99)**W(+15.99)**C(+15.99)EDDAVYLDNEK.E<br>R.NPETDTYILFNP**W(+15.99)**C(+31.99)EDDAVYLDNEK.E<br>R.NPETDTYILFNP**W(+15.99)**C(+15.99)ED(+15.99)DAVYLDNEK.E											40%	**core**
R.NPETDTYILFNPW**C(+47.98)**EDDAVYLDNEK.E<br>R.NPETDTYILFNPW**C(+47.98)**EDDAVYLDNEKER.E<br>R.NPETDTYILFNPW**C(+47.98)**EDDAVYLDNEKEREEYVLNDIGVIFYGEVNDIK.T<br>Y.ILFNPW**C(+47.98)**EDDAVYLDNEK.E	**Cys 188**	Trioxidation	n/c	60-80%	60-80%	n/c	40-60%	60-80%	n/c	20-40%	60-80%	
R.NPETDTYILFNPW**C(+15.99)**ED(+15.99)DAVYLDNEK.E<br>R.NPETDTYILFNP(+31.99)W**C(+15.99)**EDDAVYLDNEK.E<br>R.NPETDTYILFNP(+15.99)W**C(+15.99)**ED(+15.99)DAVYLDNEK.E<br>R.NPETDTYILFNPW(+31.99)**C(+15.99)**EDDAVYLDNEKEREEYVLNDIGVIFYGEVNDIK.T<br>R.NPETDTYILFN(+15.99)P(+15.99)W**C(+15.99)**EDDAVYLDNEKEREEYVLNDIGVIFYGEVNDIK.T<br>R.NPETDTYILFNPW**C(+15.99)**EDDAVYLDNEK(+31.99).E<br>R.NPETDTYILFN(+15.99)PW**C(+15.99)**EDD(+15.99)AVYLDNEK.E		Oxidation	n/c	1-20%	1-20%	n/c	1-20%	1-20%	n/c	20-40%	n/m	
R.SWSYGQFEDGILDT**C(+47.98)**LYVMD(+15.99)R.A<br>R.SWSYGQFEDGILDT**C(+47.98)**LYVMDR(+15.99).A<br>R.SWSY(+15.99)GQFEDGILDT**C(+47.98)**LYVMD(+15.99)R.A<br>R.SWSYGQFEDGILDT**C(+47.98)**LY(-2.02)VMDR.A<br>R.SW(+15.99)SYGQFEDGILDT**C(+47.98)**LYVMDR(+15.99).A<br>R.SWSYGQFEDGILDT**C(+47.98)**LYVM(+15.99)DR.A	**Cys 238**	Trioxidation	n/c	n/m	n/c	n/c	n/m	n/m	n/c	n/m	40-60%	
R.SWSYGQFEDGILDT**C(-33.99)**LYVM(+15.99)DR.A<br>R.SWSY(+31.99)GQFEDGILDT**C(-33.99)**LY.V		Dehydroalanine (from Cysteine)	n/c	n/m	n/c	n/c	n/m	40-60%	n/c	n/m	1-20%	
R.SWSYGQFEDGILDTCLYV**M(+31.99)**D(+15.99)RA.Q	**Met 242**	Dioxidation	n/m	n/m	n/c	n/m	80-100%	n/c	n/m	n/m	n/m	
R.SWSYGQFEDGILDTC(+47.98)LYV**M(+15.99)**DR.A<br>R.SWSYGQFEDGILDTC(-33.99)LYV**M(+15.99)**DR.A<br>R.SWSYGQFEDGILDTC(+31.99)LYV**M(+15.99)**DRA.Q<br>C.LYV**M(+15.99)**DR.A		Oxidation	20-40%	1-20%	n/c	40-60%	n/m	n/c	n/m	n/m	1-20%	
K.VSRVGSA**M(+15.99)**VNAK.D	**Met 265**	Oxidation	n/m	n/c	n/c	n/m	n/c	n/c	n/m	n/q	n/q	
Y.GVPPSA**W(+15.99)**TGSVDILLEYR.S	**Trp 292**	Oxidation	n/m	n/m	n/m	n/m	n/m	n/m	n/m	n/m	1-20%	
R.**Y(+33.96)**GQC(+67.92)WVFAGVFNTFLR.C<br>R.**Y(+33.96)**GQC(+47.98)W(+19.99)VFAGVFNTFLR.C	**Tyr 311**	Chlorination of tyrosine residues	n/c	n/m	n/m	n/c	n/m	n/m	n/c	n/m	1-20%	
R.YGQ**C(+47.98)**WVFAGVFNTFLR.C<br>R.Y(+33.96)GQ**C(+47.98)**W(+19.99)VFAGVFNTFLR.C<br>R.YGQ**C(+47.98)**W(+15.99)VFAGVFNTFLR.C	**Cys 314**	Trioxidation	n/c	n/q	n/q	n/c	n/q	n/q	n/c	n/q	n/q	

R.YGQ**C(+47.98)**W(+31.99)VFAGVFNTFLR.C										
R.YGQC(+47.98)**W(+15.99)**VFAGVFNTFLR.C	**Trp 315**	Oxidation	n/c	n/m	n/m	n/c	n/m	n/m	n/c	1-20%	1-20%
R.Y(+33.96)GQC(+47.98)**W(+19.99)**VFAGVFNTFLR.C		Tryptophan oxidation to hydroxykyn urenin	n/c	n/m	n/m	n/c	n/m	n/m	n/c	n/m	n/q
R.IVTNYFSAHDNDANLQ**M(+15.99)**DIFLEEDGNVNSK.L<br>R.IVTNYFSAHDNDANLQ**M(+15.99)**DIFLEEDGNVNSKLTK.D<br>R.IVTNYFSAHDNDANLQ**M(+15.99)**.D<br>R.IVTNYFSAHDNDANLQ**M(+15.99)**DIF.L<br>Y.FSAHDNDANLQ**M(+15.99)**DIFLEEDGNVNSK.L<br>F.SAHDNDANLQ**M(+15.99)**DIFLEEDGNVNSK.L<br>H.DNDANLQ**M(+15.99)**DIFLEEDGNVNSK.L<br>D.ANLQ**M(+15.99)**DIFLEEDGNVNSK.L		Oxidation	1-20%	40-60%	40-60%	1-20%	20-40%	40-60%	1-20%	80-100%	60-80%
R.IVTNYF(+31.99)SAHDN(+15.99)DANLQ**M(-48.00)**DIFLEEDGNVNSK.L<br>R.IVTNYF(+31.99)SAHDND(+15.99)ANLQ**M(-48.00)**DIFLEEDGNVNSK.L<br>R.IVTNYF(+31.99)SAHD(+15.99)NDANLQ**M(-48.00)**DIFLEEDGNVNSK.L<br>R.IVTNYFSAHDNDANLQ**M(-48.00)**D(+15.99)IF(+31.99)LEEDGNVNSK.L<br>R.IVTNY(+31.99)FSAHDNDAN(+15.99)LQ**M(-48.00)**DIFLEEDGNVNSK.L<br>R.IVTN(+15.99)YF(+31.99)SAHDNDANLQ**M(-48.00)**DIFLEEDGNVNSK.L<br>R.IVTNY(+15.99)F(+31.99)SAHDNDANLQ**M(-48.00)**DIFLEEDGNVNSK.L<br>R.IVTN(+15.99)Y(+31.99)FSAHDNDANLQ**M(-48.00)**DIFLEEDGNVNSK.L	**Met 350**	Prompt loss of side chain from oxidised Metionine	1-20%	n/m	n/m	1-20%	n/m	n/m	1-20%	n/m	n/m
R.IVTNYFSAHDNDANLQ**M(+31.99)**DIFLEEDGNVNSK.L		Dioxidation	n/m	n/m	n/m	1-20%	n/m	n/m	n/m	n/m	n/m
F.GG**W(+15.99)**QAVDSTPQENSDGM(+15.99)YR.C	**Trp 392**	Oxidation	n/m	n/m	n/m	n/m	n/m	n/m	n/m	n/m	1-20%
M.TRPDLPVGFGGWQAVDSTPQENSDG**M(+15.99)**YR.C<br>M.T(-2.02)RPDLPVGFGGWQAVDSTPQENSDG**M(+15.99)**YR.C<br>R.PDLPVGFGGWQAVDSTPQENSDG**M(+15.99)**YR.C<br>G.FGGWQAVDSTPQENSDG**M(+15.99)**YR.C<br>F.GGWQAVDSTPQENSDG**M(+15.99)**YR.C<br>F.GGW(+15.99)QAVDSTPQENSDG**M(+15.99)**YR.C<br>G.W(+31.99)QAVDSTPQENSDG**M(+15.99)**YR.C<br>W.QAVDSTPQENSDG**M(+15.99)**YR.C<br>Q.AVDSTPQENSDG**M(+15.99)**YR.C<br>V.DSTPQENSDG**M(+15.99)**YR.C<br>D.STPQENSDG**M(+15.99)**YR.C<br>T.PQENSDG**M(+15.99)**YR.C	**Met 406**	Oxidation	1-20%	40-60%	40-60%	1-20%	40-60%	40-60%	1-20%	80-100%	80-100%
K.HGHV**C(+47.98)**FQFDAPFVFAEVNSDLIYITAK.K<br>K.HGH(+15.99)V**C(+47.98)**FQFDAPFVFAEVNSDLIYITAK.K	**Cys 423**	Trioxidation	n/c	40-60%	20-40%	n/m	40-60%	60-80%	n/c	80-100%	80-100%

K.HGHV**C(+47.98)**FQFDAPFVFAEVN.S<br>K.H(+15.99)GHV**C(+47.98)**FQFDAPFVFAEVNSDLIYITAK.K<br>K.HGHV**C(+47.98)**FQFDAPFVF.A											
K.H(+15.99)GHV**C(+15.99)**FQFDAPFVFAEVNSDLIYITAK.K<br>K.H(+15.99)GHV**C(+15.99)**F(+31.99)QFDAPFVFAEVNSDLIYITAK.K<br>K.HGHV**C(+15.99)**F(+31.99)QFDAPFVFAEVNSDLIYITAK.K<br>K.HGHV**C(+15.99)**FQFD(+15.99)APFVFAEVNSDLIYITAK.K		Oxidation	n/c	n/m	1-20%	n/m	n/m	n/m	n/c	1-20%	n/m
K.LIVTKQIGGDG**M(+15.99)M(+15.99)**DITDTYK.F<br>K.QIGGDG**M(+31.99)M(+31.99)**DITDTYK.F<br>K.QIGGDG**M(+15.99)M(+15.99)**DITDTYK.F<br>K.QIGGDG**M(+15.99)M(+15.99)**DITDTY(+33.96)K.F<br>Q.IGGDG**M(+15.99)M(+15.99)**DITDTYK.F<br>I.GGDG**M(+15.99)M(+15.99)**DITDTYK.F<br>G.DG**M(+15.99)M(+15.99)**DITDTYK.F<br>D.G**M(+15.99)M(+15.99)**DITDTYK.F<br>G.**M(+15.99)M(+15.99)**DITDTYK.F	**Met 474; Met 475**	Oxidation	1-20%	80-100%	80-100%	1-20%	80-100%	80-100%	1-20%	80-100%	80-100%
R.LALETAL**M(+15.99)**YGAK.K<br>R.LALETAL**M(+15.99)**Y(+33.96)GAK.K<br>R.LALETAL**M(+15.99)**YGAKKPLNTEGVM(+15.99)K.S<br>R.LALETAL**M(+15.99)**YGA.K<br>R.LALETAL**M(+15.99)**Y.G<br>R.LALETAL**M(+15.99)**.Y<br>L.ALETAL**M(+15.99)**YGAK.K<br>A.LETAL**M(+15.99)**YGAK.K<br>T.AL**M(+15.99)**YGAK.K	**Met 499**	Oxidation	20-40%	80-100%	80-100%	20-40%	80-100%	80-100%	1-20%	80-100%	80-100%
R.LALETAL**M(+31.99)**YGAK.K<br>R.LALETAL**M(+31.99)**.Y		Dioxidation	1-20%	n/m	n/m	1-20%	n/m	n/m	n/m	n/m	n/m
K.KPLNTEGV**M(+15.99)**K.S<br>K.KPLNTEGV**M(+15.99)**.K<br>K.KPLNTEGV**M(+15.99)**KSR.S<br>K.KPLN(+.98)TEGV**M(+15.99)**K.S<br>K.KPLNTEGV**M(+15.99)**K(+27.99)SR.S<br>K.KPLNTEGV**M(+15.99)**K(-1.03)SR.S<br>K.KP(+31.99)LNTEGV**M(+15.99)**K.S<br>K.PLNTEGV**M(+15.99)**K.S<br>K.PLN(+.98)TEGV**M(+15.99)**K.S<br>K.PLNTEGVM**(+15.99)**KSR.S<br>P.LNTEGV**M(+15.99)**K.S	**Met 512**	Oxidation	40-60%	80-100%	80-100%	40-60%	80-100%	80-100%	40-60%	80-100%	80-100%
K.KPLNTEGV**M(+31.99)**K.S<br>K.KPLNTEGV**M(+31.99)**.K<br>K.KPLN(+.98)TEGV**M(+31.99)**K.S		Dioxidation	1-20%	n/m	n/m	1-20%	n/m	n/m	1-20%	n/m	n/m

K.KPLNTEGV**M(-48.00)**K.S<br>K.KPLN(+.98)TEGV**M(-48.00)**K.S<br>K.KPLNTEGV**M(-48.00)**K(+15.99).S<br>K.KPLNTEGV**M(-48.00)**K(+31.99)SR(+31.99).S<br>K.PLNTEGV**M(-48.00)**K.S		Prompt loss of side chain from oxidised Metionine	n/m	1-20%	1-20%	n/m	1-20%	1-20%	n/m	1-20%	1-20%	
R.SNVD**M(+15.99)**DFEVENAVLGK.D<br>R.SNVD**M(+15.99)**DFEVENAVLGKDFK.L<br>R.SNVD**M(+15.99)**DF.E	**Met 520**	Oxidation	n/m	1-20%	1-20%	n/m	1-20%	1-20%	n/m	1-20%	20-40%	
K.KEAVLIQAGEY**M(+15.99)**GQLLEQASLHFFVTAR.I<br>K.EAVLIQAGEY**M(+15.99)**GQLLEQASLHF.F<br>K.EAVLIQAGEY**M(+15.99)**GQLLEQASLH.F<br>K.EAVLIQAGEY**M(+15.99)**GQLLEQASLHFF.V<br>K.EAVLIQAGEY**M(+15.99)**GQLLEQASLHFFVTAR.I<br>K.EAVLIQAGEY**M(+15.99)**GQLLEQA.S<br>K.EAVLIQAGEY**M(+15.99)**GQLLEQASLHFFVT.A<br>K.EAVLIQAGEY**M(+15.99)**GQLLEQASL.H<br>K.EAVLIQAGEY**M(+15.99)**GQLLE.Q<br>K.EAVLIQAGEY**M(+15.99)**GQLLEQASLHFFVTA.R<br>K.EAVLIQAGEY(+33.96)**M(+15.99)**GQLLEQASLHFFVTAR.I<br>K.EAVLIQ(+.98)AGEY**M(+15.99)**GQLLEQASLH.F<br>K.EAVLIQAGEY**M(+15.99)**GQLLEQ.A<br>K.EAVLIQ(+.98)AGEY**M(+15.99)**GQLLEQASLHFFVTAR.I<br>V.LIQAGEY**M(+15.99)**GQLLEQASLHFFVTAR.I<br>L.IQAGEY**M(+15.99)**GQLLEQASLHFFVTAR.I<br>I.QAGEY**M(+15.99)**GQLLEQASLHFFVTAR.I<br>I.Q(+.98)AGEY**M(+15.99)**GQLLEQASLHFFVTAR.I<br>Q.AGEY**M(+15.99)**GQLLEQASLHFFVTAR.I<br>A.GEY**M(+15.99)**GQLLEQASLHFFVTAR.I<br>E.Y**M(+15.99)**GQLLEQASLHFFVTAR.I<br>Y.**M(+15.99)**GQLLEQASLHFFVTAR.I	**Met 595**	Oxidation	n/m	20-40%	40-60%	n/m	40-60%	20-40%	n/m	60-80%	80-100%	**β-barrel 1**
R.GTQVVGSD**M(+15.99)**TVTVQFTNPLK.E<br>R.GTQVVGSD**M(+15.99)**TVTVQ(+.98)FTNPLK.E<br>R.GTQVVGSD**M(+15.99)**TVTVQFTN(+.98)PLK.E<br>R.GTQVVGSD**M(+15.99)**TVTVQFTNP(-27.99)LK(+42.01).E	**Met 646**	Oxidation	1-20%	1-20%	1-20%	1-20%	1-20%	1-20%	n/m	60-80%	60-80%	
R.GTQVVGSDMTVTVQFTN**P(-27.99)**LK(+42.01).E	**Pro 655**	Pyrrolidone from Proline	n/m	n/m	n/m	n/m	n/m	n/m	n/m	1-20%	1-20%	**β-barrel 2**
R.NVWVHLDGPGVTRP**M(+15.99)**K.K<br>R.NVWVHLDGPGVTRP**M(+15.99)**.K<br>R.NVWVHLDGPGVTRP**M(+15.99)**KK.M<br>R.NVW(+15.99)VHLDGPGVTRP**M(+15.99)**K.K<br>R.NVW(+15.99)VHLDGPGVTRP**M(+15.99)**KK.M	**Met 676**	Oxidation	1-20%	20-40%	60-80%	1-20%	1-20%	20-40%	1-20%	1-20%	20-40%	

R.NVW(+31.99)VHLDGPGVTRP**M(+15.99)**KK.M<br>R.N(+15.99)VW(+15.99)VHLDGPGVTRP**M(+15.99)**KK.M<br>V.WVHLDGPGVTRP**M(+15.99)**K.K<br>V.WVHLDGPGVTRP**M(+15.99)**KK.M<br>W.VHLDGPGVTRP**M(+15.99)**K.K<br>W.VHLDGPGVTRP**M(+15.99)**KK.M<br>V.HLDGPGVTRP**M(+15.99)**K.K<br>V.HLDGPGVTRP**M(+15.99)**KK.M<br>H.LDGPGVTRP**M(+15.99)**K.K<br>L.DGPGVTRP**M(+15.99)**KK.M<br>D.GPGVTRP**M(+15.99)**K.K<br>G.PGVTRP**M(+15.99)**K.K												
V.HLDGPGVTRP**M(-48.00)**KK.M<br>V.HLDGPGVTRP**M(-48.00)**K.K		Prompt loss of side chain from oxidised Metionine	n/m	n/m	n/m	n/m	1-20%	1-20%	n/m	n/m	n/m	
R.EIRPNSTVQWEEV**C(+47.98)**RPWVSGHR.K<br>N.STVQWEEV**C(+47.98)**RPWVSGHR.K<br>N.STVQWEEV**C(+47.98)**RP(+15.99)WVSGHR.K	**Cys 695**	Trioxidation	n/c	n/c	n/c	n/c	n/c	n/c	n/c	40-60%	60-80%	
N.STVQWEEV**C(+15.99)**R(+31.99)PW(+15.99)VSGHR.K<br>N.STVQW(+15.99)EEV**C(+15.99)**R(+31.99)PWVSGHR.K		Oxidation	n/c	n/c	n/c	n/c	n/c	n/c	n/c	n/m	1-20%	
R.KLIAS**M(+15.99)**SSDSLR.H<br>K.LIAS**M(+15.99)**SSDSLR.H<br>L.IAS**M(+15.99)**SSDSLR.H<br>I.AS**M(+15.99)**SSDSLR.H<br>A.S**M(+15.99)**SSDSLR.H	**Met 709**	Oxidation	1-20%	1-20%	1-20%	1-20%	1-20%	20-40%	1-20%	80-100%	80-100%	
R.KLIAS**M(+31.99)**SSDSLR.H<br>K.LIAS**M(+31.99)**SSDSLR.H		Dioxidation	1-20%	n/m	n/m	1-20%	n/m	n/m	n/m	n/m	n/m	
K.LIAS**M(-48.00)**SSDSLR.H<br>A.S**M(-48.00)**SSDSLR.H		Prompt loss of side chain from oxidised Metionine	n/m	n/m	n/m	n/m	n/m	n/m	n/m	1-20%	1-20%	
		**Chain B**										
K.SF**Y(+33.96)**FPMSIDK.K<br>K.SF**Y(+33.96)**FPM(+15.99)SIDKK.L<br>K.SF**Y(+33.96)**FPM(+15.99)SIDK.K<br>K.SF**Y(+33.96)**FP(+15.99)MSIDK.K	**Tyr 26**	Chlorination of tyrosine residues	n/m	n/m	n/m	n/m	n/m	n/m	n/m	n/m	1-20%	**Sushi 1**
K.SFYFP**M(+15.99)**SIDKK.L<br>K.SFYFP**M(+15.99)**SIDK.K	**Met 29**	Oxidation	1-20%	20-40%	20-40%	1-20%	20-40%	1-20%	20-40%	40-60%	40-60%	

K.SFY(+67.92)FP**M(+15.99)**SIDK.K<br>K.SFY(+33.96)FP**M(+15.99)**SIDKK.L<br>K.SFY(+33.96)FP**M(+15.99)**SIDK.K<br>S.FYFP**M(+15.99)**SIDK.K<br>F.YFP**M(+15.99)**SIDK.K												
K.SFYFP**M(+31.99)**SIDKK.L<br>K.SFYFP**M(+31.99)**SIDK.K		Dioxidation	n/m	n/m	n/m	n/m	n/m	n/m	1-20%	n/m	n/m	
K.LSFF(+31.99)**C(-33.99)**LAGYTTESGR.Q	**Cys 39**	Dehydroalanine (from Cysteine)	n/m	n/m	n/m	n/m	n/m	n/m	n/m	n/m	1-20%	
R.QEEQTT**C(-33.99)**TTEGW(+31.99)SPEPR.C	**Cys 56**	Dehydroalanine (from Cysteine)	n/m	n/m	n/m	n/m	n/m	n/m	n/m	1-20%	1-20%	
K.I(+27.99)QEN(+.98)**M(-48.00)**RYGCASGYK.T	**Met 94**	Prompt loss of side chain from oxidised Metionine	n/m	n/c	n/c	n/m	n/c	n/c	n/m	n/c	80-100%	**Sushi 2**
K.VKD(+79.97)KVQYE**C(-33.99)**ATGYYTAGGK.K<br>K.VKD(+43.99)KVQY(+67.92)E**C(-33.99)**ATGYYTAGGK.K<br>K.VK(+43.99)DKVQY(+67.92)E**C(-33.99)**ATGYYTAGGK.K<br>K.VKD(+79.97)KVQY(+44.99)E**C(-33.99)**ATGYYTAGGK.K<br>K.VKDK(+43.99)VQY(+67.92)E**C(-33.99)**ATGYYTAGGK.K<br>K.VKDKVQY(+79.97)E**C(-33.99)**ATGYYTAGGK.K<br>K.VQYE**C(-33.99)**ATGYYTAGGK.K	**Cys 160**	Dehydroalanine (from Cysteine)	n/c	n/q	n/q	n/c	n/q	n/q	n/c	n/q	n/q	**Sushi 3**
K.VKD(+43.99)K(-1.03)VQYE**C(+47.98)**ATGYYTAGGK.K<br>K.VK(+14.96)DK(+27.99)VQYE**C(+47.98)**ATGYYTAGGK.K<br>K.DKVQYE**C(+47.98)**ATGYYTAGGK.K<br>K.DK(+42.01)VQY(+15.01)E**C(+47.98)**ATGYYTAGGK.K<br>K.DK(+42.01)VQY(+15.01)E**C(+47.98)**ATGYYTAGGKK.T		Trioxidation	n/c	n/q	n/q	n/c	n/q	n/q	n/c	n/q	n/q	
K.SGYLLHGSNEITCNRGK(+43.01)**W(+13.98)**T.L	**Trp 363**	Tryptophan oxidation to oxolactone	1-20%	40-60%	20-40%	1-20%	20-40%	20-40%	60-80%	80-100%	80-100%	**Sushi 6**
K.SGYLLHGSNEITCNR(-43.05)GK**W(+19.99)**.T		Tryptophan oxidation to hydroxykynurenin	n/m	1-20%	n/m	n/m	n/m	n/m	n/m	n/m	n/m	
K.HPPVV**M(+15.99)**NGAVADGILASYATGSSVEYR.C<br>K.HPPVV**M(+15.99)**NGAVADGILASYATGSSVE.Y<br>K.HPPVV**M(+15.99)**N(+.98)GAVADGILASYATGSSVEYR.C<br>K.HPPVV**M(+15.99)**NGAVADGILASYATGS.S<br>K.HPPVV**M(+15.99)**NGAVADGILASY.A	**Met 383**	Oxidation	1-20%	60-80%	60-80%	1-20%	40-60%	80-100%	1-20%	40-60%	60-80%	**Sushi 7**

K.HPPVV**M(+15.99)**N(+.98)GAVADGILASYATGSSVE.Y<br>K.HPPVV**M(+15.99)**NGAVADGIL.A<br>K.HPPVV**M(+15.99)**NGAVADGILAS.Y<br>K.H(-23.02)PPVV**M(+15.99)**NGAVAD(+79.97)GILASYATGSSVEYR.C<br>K.HPPVV**M(+15.99)**NGAVADGILASYA.T<br>K.HPPVV**M(+15.99)**NGAVAD.G<br>K.HPPVV**M(+15.99)**N.G<br>K.HPPVV**M(+15.99)**N(+.98)GAVAD.G<br>K.HPPVV**M(+15.99)**N(+.98)GAVADGILASYA.T<br>K.HPPVV**M(+15.99)**N(+.98)GAVADGILASY.A<br>K.HPPVV**M(+15.99)**.N												
K.WSSPPVCLEPCT(-2.02)VNVDY**M(+15.99)**NR.N<br>S.PPVCLEPCT(-2.02)VNVDY**M(+15.99)**NR.N	**Met 441**	Oxidation	1-20%	1-20%	20-40%	n/m	20-40%	20-40%	1-20%	1-20%	1-20%	
R.NNIE**M(+15.99)**KW(+31.99)KYEGK.V<br>R.NNIE**M(+15.99)**KW(+15.99)K.Y<br>R.NNIE**M(+15.99)**K.W<br>R.NNIE**M(+15.99)**KW(+13.98)K.Y<br>R.NNIE**M(+15.99)**K(+42.01)W(+3.99)KYEGK.V<br>R.N(+15.99)NIE**M(+15.99)**K.W<br>R.NNIE**M(+15.99)**KWK(+15.99).Y<br>R.NNIE**M(+15.99)**KW(+15.99)K(+15.99)YEGK.V	**Met 448**	Oxidation	1-20%	1-20%	1-20%	1-20%	20-40%	1-20%	n/m	1-20%	20-40%	
R.NNIE**M(+31.99)**KWKYEGK.V<br>R.NNIE**M(+31.99)**KW(+15.99)KYEGK.V<br>R.NNIE**M(+31.99)**K.W		Dioxidation	n/m	n/m	n/m	1-20%	n/m	n/m	n/m	20-40%	1-20%	
R.NNIEMK**W(+15.99)**KYEGK.V<br>R.NNIEMK**W(+15.99)**K.Y<br>R.NNIEM(+15.99)K**W(+15.99)**K.Y<br>R.NNIEMK(+15.99)**W(+15.99)**K.Y<br>R.NNIEM(+31.99)K**W(+15.99)**KYEGK.V<br>R.NNIEM(+15.99)K**W(+15.99)**K(+15.99)YEGK.V<br>R.NNIEMK**W(+15.99)**K(+15.99)YEGK.V	**Trp 450**	Oxidation	1-20%	40-60%	60-80%	1-20%	40-60%	40-60%	1-20%	60-80%	60-80%	**Sushi 8**
R.NNIEM(+15.99)K**W(+13.98)**K.Y		Tryptophan oxidation to oxolactone	n/m	1-20%	1-20%	n/m	1-20%	1-20%	n/m	1-20%	1-20%	
R.NNIEM(+15.99)K(+42.01)**W(+3.99)**KYEGK.V<br>R.NNIEMK(+42.01)**W(+3.99)**KYEGK.V		Tryptophan oxidation to kynurenin	n/m	n/m	n/m	n/m	n/m	1-20%	n/m	n/m	n/m	
R.NNIEMK(+42.01)**W(+19.99)**KYEGK.V		Tryptophan oxidation to hydroxykynurenin	n/m	n/m	n/m	n/m	1-20%	n/m	n/m	n/m	n/m	

K.QGYDLSPLTPLSELSVQ**C(-33.99)**NRGEVK(+14.96).Y<br>S.PLTPLSELSVQ**C(-33.99)**NRGEVK(+14.96).Y	**Cys 485**	Dehydroalanine (from Cysteine)	n/q	n/q	n/q	n/q	n/q	n/q	n/q	n/q	n/q	
K.QGYDLSPLTPLSELSVQC(-33.99)NRGEV**K(+14.96)**.Y<br>S.PLTPLSELSVQC(-33.99)NRGEV**K(+14.96)**.Y	**Lys 491**	Lysine oxidation to a-aminoadipic acid	n/q	n/q	n/q	n/q	n/q	n/q	n/q	n/q	n/q	
K.NNLLLK**W(+13.98)**DFDNRPH.I	**Trp 578**	Tryptophan oxidation to oxolactone	n/m	n/m	n/m	n/m	n/m	1-20%	n/m	1-20%	1-20%	**Sushi 10**
K.NNLLLK(+42.01)**W(+3.99)**DFDNRPH.I		Tryptophan oxidation to kynurenin	n/m	n/c	n/m	n/m	n/m	1-20%	n/m	1-20%	n/m	

Notes:
- n/c — not detected in sequence (no coverage);
- n/m — no modifications found for this AAR;
- n/q — no quantitative data for this modification.

Table S2. Export data on peptides and modifications found by Peaks Studio 8.5.

Protein Group	Protein ID	Protein Accession	Peptide	Unique	-10lgP	Mass	Length	ppm	m/z	z	RT	Intensity p13	Intensity c13	Intensity a13	Intensity p13_50 µM	Intensity c13_50µM	Intensity a13_50µM	Intensity p13_150 µM	Intensity c13_150 µM	Intensity a13_150µM	Fraction	Scan	Start	End	PTM	AScore
72	53	P00450	R.MFTTAPDQVDKE.D	Y	62,41	1380,623	12	2,8	691,3207	2	30,6			2,97E+06							19	F19:2600	599	610		
72	53	P00450	E.VGDTIR.V	N	29,37	659,3602	6	3,7	660,3699	1	24,8			3,26E+05							19	F19:2003	457	462		
72	53	P00450	K.GAY(+15.99)P(-27.99)LSIEPIGVR.F	Y	25,79	1358,7557	13	12	680,3932	2	32,9			3,28E+06							19	F19:2829	469	481		Y3:Oxidation or Hydroxylation:95.35; P4:Pyrrolidone from Proline:105.53
56	20	P00734	L.QVVNLPIVERPVC(+47.98)K.D	Y	73,55	1640,892	14	8,1	821,4599	2	32,3			2,02E+06							20	F20:2682	524	537	Cysteine oxidation to cysteic acid	C13:Cysteine oxidation to cysteic acid:1000.00
56	20	P00734	K.YGFYTHVFR.L	Y	71,19	1188,5715	9	3,8	595,2953	2	33,3			1,44E+06							19	F19:2877	600	608		
56	20	P00734	K.IYIHPR.Y	Y	55,57	797,4548	6	1,6	399,7353	2	27,6			2,36E+07							19	F19:2288	447	452		
56	20	P00734	R.TFGSGEADC(+31.99)GLRPLFEK.K	Y	53,34	1857,8567	17	8,7	929,9437	2	33,5			2,77E+06							20	F20:2777	328	344		C9:Dihydroxy:21.15
56	20	P00734	E.GDSGGPFVMK.S	Y	51,39	993,4589	10	2,2	497,7379	2	31,1			4,09E+06							19	F19:2656	566	575		

56	20	P00734	E.KIYIHPR.Y	Y	47,56	925,5497	7	2,5	463,7833	2	27,2			2,42E+06							19	F19:2252	446	452		
56	20	P00734	R.VTGWGN.L	Y	33,72	632,2918	6	2,6	633,3007	1	29			2,51E+06							19	F19:2428	501	506		
56	20	P00734	K.IYIHP.R	Y	28,76	641,3537	5	5,1	321,6858	2	27,6			1,20E+06							20	F20:2257	447	451		
56	20	P00734	R.YPHKPE.I	Y	28,12	769,3759	6	1,7	385,6959	2	24,4			0							19	F19:1963	136	141		
56	20	P00734	L.LVRIGKHSR.T	Y	26,56	1064,6566	9	5,4	355,8947	3	22,6			3,02E+06							21	F21:1791	423	431		
56	20	P00734	L.VRIGKHSR.T	Y	25,49	951,5726	8	4,9	318,1997	3	17,5			2,90E+05							21	F21:1386	424	431		
94	46	P00740	R.YVNWIK.E	Y	39,58	821,4435	6	4,4	411,7309	2	29,4			9,72E+05							21	F21:2427	450	455		
94	46	P00740	E.YTNIFLK.F	Y	35,19	897,496	7	2,9	449,7566	2	33,5			1,92E+06							19	F19:2896	341	347		
94	46	P00740	R.VPLVDR.A	Y	32,81	697,4122	6	2,1	698,421	1	29,1			5,06E+06							19	F19:2442	374	379		
70	38	P00742	K.TGIVSGFGR.T	Y	52,66	892,4766	9	6,9	447,2487	2	28,8			9,40E+06							20	F20:2365	358	366		
70	38	P00742	K.ETYDFDIAVLR.L	Y	45,59	1340,6611	11	6,3	671,342	2	34,5			3,23E+06							20	F20:2859	317	327		
70	38	P00742	K.MLEVPYVDR.N	Y	44,88	1120,5586	9	3	561,2883	2	33,9			3,50E+06							19	F19:2936	379	387		
70	38	P00742	K.M(+15.99)LEVPYVDR.N	Y	41,1	1136,5536	9	5,9	569,2874	2	30,5			6,98E+06							20	F20:2524	379	387	Oxidation (M)	M1:Oxidation (M):1000.00
51	19	P00747	R.HSIFTPETNPR.A	Y	68,06	1297,6415	11	0	649,828	2	28,2	8,08E+05	1,81E+06	3,59E+06	1,71E+06	3,34E+05	1,35E+06	1,81E+06	5,37E+05	2,83E+06	6	F6:2463	513	523		
51	19	P00747	R.AFQYHSK.E	Y	48,89	879,4239	7	1,8	440,72	2	26,6	0									1	F1:2136	63	69		
51	19	P00747	R.FVTWIEGVMR.N	Y	46,77	1236,6324	10	-3,9	619,3211	2	36,1	0									4	F4:3183	799	808		
51	19	P00747	K.EAQLPVIENK.V	Y	35,25	1139,6187	10	7,6	570,8209	2	29,7	7,64E+05	1,77E+06								11	F11:2409	718	727		
51	19	P00747	K.LSSPAVITDK.V	Y	35,21	1029,5706	10	-4,2	1030,5735	1	29	0									5	F5:2541	671	680		
51	19	P00747	R.LFLEPTR.K	Y	29,59	874,4912	7	5,4	438,2552	2	30,6	1,30E+06									3	F3:2498	657	663		
51	19	P00747	K.DIALLK.L	Y	29,56	671,4218	6	5,7	672,4329	1	30,3		4,30E+05	1,21E+07							20	F20:2500	665	670		
93	76	P01009	K.TDTSHHDQDHPTFNK.I	Y	44,13	1778,7609	15	-8,6	890,3801	2	25,9		1,13E+06								10	F10:2074	35	49		
76	49	P01023	K.SLNEEAVKK.D	Y	45,04	1016,5502	9	0,6	509,2827	2	19,3	9,16E+04									4	F4:1599	1169	1177		
76	49	P01023	K.TAQEGDHGSHVYTK.A	Y	34,73	1528,6907	14	-1,4	510,5701	3	13,5	0									6	F6:1054	1134	1147		
76	49	P01023	K.EY(+15.01)EMK.L	Y	27,58	713,3054	5	0,6	357,6602	2	16,3	0	0								5	F5:1316	315	319	Tyrosine oxidation to 2-	Y2:Tyrosine oxidation

																					aminotyrosine	to 2-aminotyrosine:1000.00
30	39	P01024	R.EPGQDLVVLPLSITTDFIPSFR.L	Y	72,49	2443,2998	22	8,3	1222,6674	2	44,7			0	0	5,45E+05	48	F48:2856	509	530		
30	39	P01024	K.AGDFLEANYMNLQR.S	Y	63,97	1640,7617	14	-0,2	821,3879	2	34,3	0	0	0			6	F6:3025	1172	1185		
30	39	P01024	K.GPLLNK.F	Y	49,93	640,3908	6	-1,5	321,2022	2	21,9	0					6	F6:1846	1204	1209		
30	39	P01024	R.WEDPGK.Q	Y	46,91	730,3286	6	-1	366,1712	2	22,2	3,36E+05	2,28E+05	1,49E+05			13	F13:1805	1220	1225		
30	39	P01024	R.WLNEQR.Y	Y	32,48	844,4191	6	1,1	845,4273	1	26,7	0					5	F5:2313	1255	1260		
50	13	P01031	K.LQGTLPVEAR.E	Y	60,16	1082,6084	10	-1,7	542,3105	2	29,5	7,17E+05	7,88E+05	1,88E+06			5	F5:2587	1129	1138		
50	13	P01031	K.GLLVGEILSAVLSQEGINILTHLPK.G	Y	56,72	2613,5105	25	-0,6	872,1769	3	45,4	5,49E+06	2,58E+06				5	F5:3973	981	1005		
50	13	P01031	R.YGGGFYSTQDTINAIEGLTEYSLLVK.Q	Y	56,22	2838,3962	26	4,4	947,1436	3	41,6	0					5	F5:3668	1280	1305		
50	13	P01031	K.KIEEIAAK.Y	Y	53,11	900,528	8	-2,1	451,2703	2	20,4	5,08E+05	8,09E+05	1,08E+06			6	F6:1704	682	689		
50	13	P01031	R.VLGQVNK.Y	Y	42,51	756,4493	7	-4,1	757,4536	1	19,4	2,72E+05	0	0			14	F14:1571	1085	1091		
50	13	P01031	K.ENSQYQPIK.L	Y	42,29	1105,5404	9	-0,1	553,7774	2	26			7,04E+05			22	F22:2177	1120	1128		
50	13	P01031	F.HSDPLIEK.Q	Y	42,07	937,4869	8	0	469,7507	2	20,3			7,14E+04			24	F24:1650	1035	1042		
50	13	P01031	R.IPLDLVPK.T	Y	39,89	893,5586	8	2,6	447,7878	2	35,2	7,50E+05	1,02E+06				1	F1:2950	963	970		
50	13	P01031	R.ESYSGVTLDPR.G	Y	38,38	1222,583	11	7,6	612,3034	2	29,7		1,87E+05				12	F12:2405	937	947		
50	13	P01031	K.THPQFR.S	Y	37,73	784,398	6	4,6	393,2081	2	22,4		5,71E+04				11	F11:1782	1201	1206		
50	13	P01031	K.DINYVNPVIK.W	Y	35,51	1173,6394	10	4,8	587,8298	2	31,5		2,27E+05				11	F11:2558	1263	1272		
35	17	P01042	K.TVGSDTFYSFK.Y	Y	66,9	1250,5819	11	3,1	626,3002	2	33,9	1,74E+06	1,81E+06				1	F1:2827	65	75		
35	17	P01042	K.YFIDFVAR.E	Y	66,16	1029,5283	8	3,4	515,7732	2	36,9	1,65E+06	1,66E+06				1	F1:3103	317	324		
35	17	P01042	R.DIPTNSPELEETLTHTITK.L	Y	65,98	2138,0742	19	-2,1	1070,0421	2	34,5	0	4,44E+06	3,52E+06			5	F5:3043	270	288		
35	17	P01042	K.AVDAALKK.Y	Y	51,87	814,4912	8	-0,4	408,2527	2	15,2	0		1,70E+05			23	F23:1188	36	43		
35	17	P01042	K.YNSQNQSNNQFVLYR.I	Y	46,82	1873,8707	15	6,6	937,9489	2	31,1	0					4	F4:2728	44	58		
35	17	P01042	R.VQVVAGK.K	Y	45,86	699,4279	7	-1,8	700,434	1	17	2,37E+06	1,56E+06	8,00E+05			22	F22:1333	309	315		
35	17	P01042	E.TLTHTITK.L	Y	44,94	913,5233	8	2,3	457,77	2	26,4	8,24E+05	0	0			19	F19:2163	281	288		

35	17	P01042	K.AVDAALK.K	Y	43,55	686,3962	7	-0,2	344,2053	2	21,3	0	8,88E+04								14	F14:1743	36	42		
35	17	P01042	K.RPPGFSPF.R	Y	42,92	903,4602	8	5,2	452,7397	2	31,1			3,49E+06							20	F20:2568	381	388		
35	17	P01042	D.C(+27.99)LGCVHP(-30.01)ISTQSPDLEPILR.H	Y	36,81	2175,0815	20	3,2	726,0368	3	36,1	1,02E+06									1	F1:3032	142	161	Formylation	C1:Formylation:1000.00;P7:Proline oxidation to pyrrolidinone:45.97
35	17	P01042	I.PTNSPELEETLTHTITK.L	Y	33,17	1909,9633	17	7,6	637,6665	3	37									4,09E+05	76	F76:2270	272	288		
35	17	P01042	D.CLGCVHPIS(-2.02)TQSPDLEPILR.H	Y	31,23	2175,0815	20	1,6	726,0356	3	35,8		1,13E+06								10	F10:2991	142	161		S9:2-amino-3-oxo-butanoic_acid:12.28
67	41	P01834	K.VDNALQSGNSQESVTEQDSK.D	Y	120	2134,9614	20	9,2	1068,4978	2	30	0	0	1,41E+05	5,66E+06	5,88E+06	4,49E+06	3,64E+06	5,04E+06	5,76E+06	38	F38:1689	43	62		
67	41	P01834	R.TVAAPSVFIFPPSDEQLK.S	Y	39,86	1945,0197	18	8	973,5248	2	38,9									4,45E+05	79	F79:2414	2	19		
67	41	P01834	E.VTHQGLSSPVTK.S	Y	27,06	1252,6775	12	4,3	627,3487	2	28,2	2,58E+05	1,25E+06								1	F1:2280	89	100		
32	18	P01857	R.VVSVLTVLHQDWLNGK.E	N	106,6	1806,9991	16	6,5	904,5128	2	39,3	2,04E+06	2,31E+06	1,40E+05	5,60E+06	7,04E+06	7,97E+06	7,88E+06	7,56E+06	1,17E+07	59	F59:2454	185	200		
32	18	P01857	K.FNWYVDGVEVHNAK.T	N	88,38	1676,7947	14	8	839,4113	2	35,2				3,91E+06	2,95E+06	2,70E+06	6,51E+06	3,22E+06	6,56E+06	74	F74:2114	158	171		
32	18	P01857	K.TTPPVLDSDGSFFLYSK.L	Y	70,15	1872,9146	17	6,9	937,471	2	38,5						4,44E+05			8,35E+05	74	F74:2387	276	292		
32	18	P01857	V.SVLTVLHQDWLNGK.E	N	64,33	1608,8624	14	6,4	805,4437	2	39,7				6,01E+05			1,62E+05		1,69E+06	76	F76:2496	187	200		
32	18	P01857	K.GFYPSDIAVEWESNGQPENNYK.T	N	48,76	2543,124	22	11	1272,5837	2	37,9						7,44E+05			4,50E+05	48	F48:2331	254	275		
32	18	P01857	E.ALHNHYTQK.S	N	48,75	1110,557	9	3,6	556,2878	2	24,5	3,80E+05	3,09E+05	2,51E+05							1	F1:1951	314	322		
32	18	P01857	I.SRTP(-27.99)EVTC(+31.99)VVVDVSHEDPEVK.F	Y	31,77	2328,1267	21	-2,1	777,0479	3	33,6						1,57E+06	6,88E+05		1,68E+06	60	F60:1961	137	157		P4:Pyrrolidone from Proline:95.14;C8:Dihydroxy:0.00
32	18	P01857	S.R(+43.01)TPEVTC(+47.98)VVVDVSHEDPEVK.F	Y	30,35	2328,0903	20	11	777,0456	3	33,7				1,57E+06		1,10E+06	4,63E+06		1,97E+06	54	F54:1987	138	157	Cysteine oxidation to cysteic acid	R1:Carbamylation:68.89;C7:Cysteine oxidation to cysteic acid:1000.00
32	18	P01857	K.ALPAPIEK.T	N	28	837,496	8	2,6	419,7563	2	30,4		1,23E+06								10	F10:2493	210	217		
32	18	P01857	I.SR(+31.99)TP(-27.99)EVTCVVVDVSHEDPEVK.F	Y	27,63	2328,1267	21	-1,1	777,0486	3	33,5				1,33E+06						34	F34:1979	137	157		R2:Dihydroxy:0.00;P4:Pyrrolidone from Proline:92.72
36	45	P01860	R.VVSVLTVLHQDWLNGK.E	N	106,6	1806,9991	16	6,5	904,5128	2	39,3	2,04E+06	2,31E+06	1,40E+05	5,60E+06	7,04E+06	7,97E+06	7,88E+06	7,56E+06	1,17E+07	59	F59:2454	232	247		

36	45	P01860	V.SVLTVLHQDWLNGK.E	N	64,33	1608,8624	14	6,4	805,4437	2	39,7				6,01E+05			1,62E+05		1,69E+06	76	F76:2496	234	247		
36	45	P01860	K.ALPAPIEK.T	N	28	837,496	8	2,6	419,7563	2	30,4		1,23E+06								10	F10:2493	257	264		
29	28	P01861	R.VVSVLTVLHQDWLNGK.E	N	106,6	1806,9991	16	6,5	904,5128	2	39,3	2,04E+06	2,31E+06	1,40E+05	5,60E+06	7,04E+06	7,97E+06	7,88E+06	7,56E+06	1,17E+07	59	F59:2454	182	197		
29	28	P01861	Q.FNWYVDGVEVHNAK.T	N	88,38	1676,7947	14	8	839,4113	2	35,2				3,91E+06	2,95E+06	2,70E+06	6,51E+06	3,22E+06	6,56E+06	74	F74:2114	155	168		
29	28	P01861	V.SVLTVLHQDWLNGK.E	N	64,33	1608,8624	14	6,4	805,4437	2	39,7				6,01E+05			1,62E+05		1,69E+06	76	F76:2496	184	197		
29	28	P01861	K.GFYPSDIAVEWESNGQPENNYK.T	N	48,76	2543,124	22	11	1272,5837	2	37,9						7,44E+05			4,50E+05	48	F48:2331	251	272		
29	28	P01861	E.ALHNHYTQK.S	N	48,75	1110,557	9	3,6	556,2878	2	24,5	3,80E+05	3,09E+05	2,51E+05							1	F1:1951	311	319		
49	29	P01871	K.QVGSGVTTDQVQAEAK.E	Y	73,99	1616,8005	16	-0,6	809,407	2	27,8	0	0	0							15	F15:2391	154	169		
49	29	P01871	K.ESGPTTYK.V	Y	45,81	881,413	8	-0,5	441,7136	2	15,9	0									5	F5:1275	170	177		
49	29	P01871	R.QIQVSWLR.E	Y	40,3	1028,5767	8	2,7	515,297	2	35		5,87E+05								10	F10:2927	143	150		
49	29	P01871	R.VFAIPPSFASIFLTK.S	Y	39,06	1636,9229	15	3,6	819,4717	2	42,3		2,23E+05								10	F10:3527	224	238		
21	9	P01876	R.QEPSQGTTTFAVTSILR.V	Y	93,05	1834,9425	17	7,1	918,485	2	37,1	1,30E+07	1,07E+07	9,29E+06	5,68E+07	4,97E+07	3,49E+07	6,10E+07	4,09E+07	4,57E+07	34	F34:2292	283	299		
21	9	P01876	R.WLQGSQELPR.E	N	73,95	1212,6251	10	1,2	607,3206	2	30,5	5,12E+06	6,81E+06	1,46E+07							13	F13:2610	264	273		
21	9	P01876	K.SAVQGPPER.D	N	70,39	939,4774	9	-1,4	470,7453	2	21,1	9,04E+05	1,55E+06	4,81E+05							6	F6:1768	169	177		
21	9	P01876	E.ALPLAFTQK.T	N	68,38	987,5753	9	2,6	494,7962	2	34,4	3,88E+06	4,73E+06								1	F1:2871	319	327		
21	9	P01876	R.DASGVTFTWTPSSGK.S	Y	67,94	1539,7205	15	3,2	770,87	2	34,6	0	1,25E+06		2,42E+06	1,15E+06	0	4,48E+06	4,60E+06	5,36E+06	10	F10:2885	154	168		
21	9	P01876	R.VAAEDWK.K	N	64,38	817,397	7	-0,6	409,7055	2	26,3	4,38E+06	2,41E+06	2,94E+06							15	F15:2247	300	306		
21	9	P01876	K.SGNTFRPEVH.L	N	63,92	1142,5469	10	6,1	572,2842	2	28,8	1,37E+05		0	6,86E+06	2,76E+06	2,86E+06	8,50E+06	2,92E+06	6,24E+06	29	F29:1592	222	231		
21	9	P01876	R.LSLHRPALE.D	N	56,1	1034,5873	9	2,5	518,3022	2	31,2	1,46E+06	1,86E+06								10	F10:2571	127	135		
21	9	P01876	K.TPLTATLSK.S	Y	55,62	930,5386	9	6,3	466,2795	2	28,5	2,64E+06	4,54E+06					5,36E+05			11	F11:2305	213	221		
21	9	P01876	K.YLTWASR.Q	N	52,26	895,4552	7	1,6	448,7356	2	32,1	9,52E+05	1,31E+06								10	F10:2661	276	282		
21	9	P01876	K.SGNTFRPE.V	N	43,26	906,4195	8	3	454,2184	2	27,4	4,70E+05	1,10E+06								1	F1:2206	222	229		
21	9	P01876	R.GFSPK.D	N	40,91	534,2802	5	0,4	535,2877	1	14,1	3,12E+05	5,71E+05	0							23	F23:1090	254	258		
21	9	P01876	K.DVLVR.W	N	36,38	600,3595	5	-0,2	601,3666	1	25,2	9,15E+05	3,54E+06	0							23	F23:2126	259	263		
21	9	P018	K.TFTC(+15.99)TAAYPESK(-	Y	25,	1744	16	1,	873,	2	3		3,09E+07								10	F10:	201	216	Oxidation	C4:Oxidati

		76	1.03)TPLT.A		29	,786 5		8	4021		7, 4											313 4			or Hydroxyla tion (C); Lysine oxidation to aminoadip ic semialdeh yde	on or Hydroxylat ion (C):1000.0 0;K12:Lysi ne oxidation to aminoadip ic semialdeh yde:1000.0 0
43	16	P018 77	R.WLQGSQELPR.E	N	73, 95	1212 ,625 1	10	1, 2	607, 3206	2	3 0, 5	5,12E+06	6,81E+06	1,46E+ 07							13	F13: 261 0	251	260		
43	16	P018 77	K.SAVQGPPER.D	N	70, 39	939, 4774	9	- 1, 4	470, 7453	2	2 1, 1	9,04E+05	1,55E+06	4,81E+ 05							6	F6:1 768	156	164		
43	16	P018 77	E.ALPLAFTQK.T	N	68, 38	987, 5753	9	2, 6	494, 7962	2	3 4, 4	3,88E+06	4,73E+06								1	F1:2 871	306	314		
43	16	P018 77	R.VAAEDWK.K	N	64, 38	817, 397	7	- 0, 6	409, 7055	2	2 6, 3	4,38E+06	2,41E+06	2,94E+ 06							15	F15: 224 7	287	293		
43	16	P018 77	K.SGNTFRPEVH.L	N	63, 92	1142 ,546 9	10	6, 1	572, 2842	2	2 8, 8	1,37E+05		0	6,86E+0 6	2,76E+0 6	2,86E+0 6	8,50E+0 6	2,92E+0 6	6,24E+ 06	29	F29: 159 2	209	218		
43	16	P018 77	R.LSLHRPALE.D	N	56, 1	1034 ,587 3	9	2, 5	518, 3022	2	3 1, 2	1,46E+06	1,86E+06								10	F10: 257 1	114	122		
43	16	P018 77	K.YLTWASR.Q	N	52, 26	895, 4552	7	1, 6	448, 7356	2	3 2, 1	9,52E+05	1,31E+06								10	F10: 266 1	263	269		
43	16	P018 77	K.SGNTFRPE.V	N	43, 26	906, 4195	8	3	454, 2184	2	2 7, 4	4,70E+05	1,10E+06								1	F1:2 206	209	216		
43	16	P018 77	R.GFSPK.D	N	40, 91	534, 2802	5	0, 4	535, 2877	1	1 4, 1	3,12E+05	5,71E+05	0							23	F23: 109 0	241	245		
43	16	P018 77	K.DVLVR.W	N	36, 38	600, 3595	5	- 0, 2	601, 3666	1	2 5, 2	9,15E+05	3,54E+06	0							23	F23: 212 6	246	250		
58	33	P026 47	R.EQLGPVTQEFWDNLEK.E	Y	87, 85	1931 ,926 5	16	8, 6	966, 9788	2	3 9, 2			2,89E+ 07			3,54E+0 7			3,89E+ 07	54	F54: 244 6	86	101		
58	33	P026 47	K.LR(+15.99)EQLGP(- 30.01)VTQEFWDNLEK.E	Y	55, 18	2187 ,095 9	18	5, 5	1094 ,561 3	2	3 8, 1						2,33E+0 5				50	F50: 232 3	84	101	Proline oxidation to pyrrolidin one	R2:Oxidati on or Hydroxylat ion:83.24; P7:Proline oxidation to pyrrolidino ne:1000.00
58	33	P026 47	K.DLEEVK.A	Y	43, 46	731, 3701	6	0, 9	732, 3781	1	2 4, 9			2,47E+ 06							22	F22: 206 9	113	118		
58	33	P026 47	N.G(+27.99)GARLAEYHAK.A	Y	42, 07	1199 ,604 6	11	7	600, 8138	2	2 8, 7			7,96E+ 04						0	26	F26: 158 9	209	219		G1:Formyl ation:132. 72
58	33	P026 47	R.QGLLPVLE.S	Y	39	867, 5065	8	4, 7	868, 5179	1	3 8, 2			1,59E+ 06							19	F19: 332 5	240	247		
58	33	P026 47	R.LEALK.E	Y	29, 27	572, 3533	5	0, 3	573, 3608	1	1 9, 7			8,14E+ 05							22	F22: 158 3	202	206		
58	33	P026 47	R.QRLAAR(- 43.05)LEALKENGGAR(+15.99).L	Y	25, 04	1824 ,980 6	17	1, 3	913, 4988	2	4 0, 8		2,74E+06								10	F10: 341 7	196	212		R6:Arginin e oxidation to glutamic semialdeh yde:0.00;R

																					17:Oxidation or Hydroxylation:2.44
14	5	P02671	R.NPSSAGSWNSGSSGPGSTGNR.N	Y	110,9	1962,8416	21	1,1	982,4291	2	26,6	0	0	1,44E+06		24	F24:2250	288	308		
14	5	P02671	R.GGSTSYGTGSETESPR.N	Y	91,94	1571,6699	16	6,6	786,8474	2	24,8	7,25E+06	9,22E+06	2,48E+06	0	11	F11:1975	272	287		
14	5	P02671	K.TFPGFFSPM(+15.99)LGEFVSETESR.G	Y	87,63	2280,0408	20	9,1	1141,0381	2	37,6		4,14E+05			11	F11:3041	528	547	Oxidation (M)	M9:Oxidation (M):1000.00
14	5	P02671	R.HRHPDEAAFFDTASTGK.T	Y	87,63	1885,8706	17	6,4	629,6348	3	28,7	2,02E+07	1,73E+07			2	F2:2330	511	527		
14	5	P02671	R.HPDEAAFFDTASTGK.T	Y	80,29	1592,7107	15	8,3	797,3693	2	29,6	1,15E+07	1,15E+07			2	F2:2413	513	527		
14	5	P02671	K.MADEAGSEADHEGTHSTK.R	Y	73,01	1871,7592	18	-2,9	936,8842	2	15,6		0			14	F14:1213	603	620		
14	5	P02671	K.TVIGPDGHKEVTK.E	Y	73,01	1379,7408	13	9,4	690,8842	2	24,2			2,17E+06		20	F20:1928	468	480		
14	5	P02671	R.EVDLKDYEDQQK.Q	Y	72,49	1508,6995	12	2,2	755,3586	2	28,9		0			13	F13:2452	191	202		
14	5	P02671	R.PGSTGTWNPGSSER.G	Y	72,44	1431,6378	14	1,4	716,8272	2	27,4	0	1,18E+06	2,31E+05		13	F13:2313	354	367		
14	5	P02671	K.ESSSHHPGIAEFPSR.G	Y	70,79	1636,7593	15	7,1	546,5976	3	26,7	8,41E+06	1,08E+07	5,63E+06		20	F20:2170	559	573		
14	5	P02671	R.GSESGIFTNTK.E	Y	69,62	1139,5459	11	5,4	570,7833	2	27,2			4,43E+06		21	F21:2221	548	558		
14	5	P02671	R.PGSTGTW(+15.99)NPGSSER.G	Y	68,64	1447,6328	14	8	724,8295	2	26,2		0			12	F12:2100	354	367	Oxidation (HW)	W7:Oxidation (HW):1000.00
14	5	P02671	K.EKVTSGSTTTTR.R	Y	65,38	1266,6416	12	7,7	634,3329	2	17,3			1,80E+05		20	F20:1360	447	458		
14	5	P02671	K.TFPGFFSPM(+15.99)LGEFVSETE.S	Y	62,73	2036,9077	18	12	1019,4732	2	39	7,32E+05	1,02E+06			2	F2:3161	528	545	Oxidation (M)	M9:Oxidation (M):1000.00
14	5	P02671	M.LGEFVSETESR.G	Y	62,15	1252,5935	11	8,6	627,3094	2	28,9	6,43E+06	3,31E+06	6,73E+05		2	F2:2349	537	547		
14	5	P02671	K.VTSGSTTTTR.R	Y	61,61	1009,504	10	1,9	505,7603	2	11,4	1,20E+06	0	3,55E+05		22	F22:802	449	458		
14	5	P02671	D.LGTLSGIGTLDGFR.H	Y	60,63	1405,7565	14	2,4	703,8872	2	36,6		2,95E+06			10	F10:3069	497	510		
14	5	P02671	K.QFTSSTSYNR.G	Y	58,78	1189,5364	10	6,9	595,7795	2	25,5	3,77E+05	0			2	F2:2061	582	591		
14	5	P02671	R.HRHPDEAAFFDTA.S	Y	57,6	1512,6746	13	7,3	757,3501	2	29,9	5,00E+06	3,00E+06			2	F2:2434	511	523		
14	5	P02671	K.ALTDMPQMR.M	Y	56,56	1061,4998	9	1,4	531,7579	2	32,2	0	1,15E+06			1	F1:2668	250	258		
14	5	P02671	K.TVIGPDGHK.E	Y	56,08	922,4872	9	-0,3	308,5029	3	16,2	2,44E+07	2,40E+07	1,91E+07		23	F23:1286	468	476		
14	5	P02671	F.FDTASTGK.T	Y	53,17	825,3868	8	5,7	413,7031	2	21,6		2,33E+05	4,61E+06		20	F20:1703	520	527		

14	5	P02671	E.VSGNVSPGTR.R	Y	52,42	972,4988	10	3	487,2582	2	25,3	9,33E+05	1,81E+06		10	F10:2014	416	425		
14	5	P02671	L.SGIGTLDGFR.H	Y	52,07	1021,5192	10	1,7	511,7678	2	33,7	1,98E+06	2,44E+06		1	F1:2810	501	510		
14	5	P02671	K.ALTDM(+15.99)PQM(+15.99)R.M	Y	52,03	1093,4896	9	8,3	547,7567	2	24,6	2,64E+06	3,05E+06		2	F2:1983	250	258	Oxidation (M)	M5:Oxidation (M):1000.00;M8:Oxidation (M):1000.00
14	5	P02671	E.SGIFTNTK.E	Y	49,79	866,4498	8	2,2	434,2331	2	30,1	3,11E+06	2,80E+06		1	F1:2463	551	558		
14	5	P02671	R.VSEDLR.S	Y	48,62	717,3657	6	-1,9	359,6895	2	19	5,30E+05	5,41E+05	2,96E+05	23	F23:1546	130	135		
14	5	P02671	R.M(+15.99)ELERPGGNEITR.G	Y	48,26	1516,7303	13	5,2	506,5867	3	26,4	4,19E+06	5,03E+06	2,71E+06	11	F11:2120	259	271	Oxidation (M)	M1:Oxidation (M):1000.00
14	5	P02671	K.TFPGFFSPM(+15.99)LGEF.V	Y	45,52	1491,6744	13	5,2	746,8484	2	39,1			1,81E+06	20	F20:3211	528	540	Oxidation (M)	M9:Oxidation (M):1000.00
14	5	P02671	R.HRHPDEAAF.F	Y	45,5	1078,4944	9	6,7	540,2581	2	24,4			2,79E+07	20	F20:1949	511	519		
14	5	P02671	K.EVVTSED.G	Y	44,78	777,3392	7	0,8	778,3471	1	18,8	2,18E+05	1,71E+05	3,02E+05	24	F24:1511	481	487		
14	5	P02671	K.VPPEWK.A	Y	44,22	754,4014	6	1,8	378,2086	2	29,7	4,57E+05			1	F1:2423	244	249		
14	5	P02671	R.GSAGHWTSES.S	Y	44,17	1017,4152	10	-1,2	509,7143	2	20,6	0	9,09E+04	3,45E+05	23	F23:1692	368	377		
14	5	P02671	P.GIAEFPSR.G	Y	43,72	875,4501	8	1,9	438,7331	2	31,2	3,62E+05			1	F1:2577	566	573		
14	5	P02671	K.TFPGFF.S	Y	42,62	714,3376	6	2,9	715,347	1	39	4,97E+06	4,56E+06	2,05E+06	19	F19:3395	528	533		
14	5	P02671	T.VIGPDGHK.E	Y	41,22	821,4395	8	-1	411,7266	2	13,8	6,16E+05			5	F5:1075	469	476		
14	5	P02671	K.ESSSHHPGIA.E	Y	40,66	1020,4625	10	4,6	511,2408	2	21,7	1,69E+05	4,08E+05	3,83E+05	2	F2:1739	559	568		
14	5	P02671	K.TVIGPDGHKE.V	Y	39,19	1051,5298	10	3,6	526,774	2	26,7	9,39E+06	1,54E+07	1,15E+07	1	F1:2140	468	477		
14	5	P02671	K.QLEQVIAK.D	Y	38,53	927,5389	8	-0,1	464,7767	2	27,9		0		15	F15:2404	203	210		
14	5	P02671	S.SHHPGIAEFPSR.G	Y	38,06	1333,6527	12	1,6	445,5589	3	30,3	1,18E+06			1	F1:2483	562	573		
14	5	P02671	K.TFPGFFSPM(+15.99)LGE.F	Y	37,17	1344,606	12	5,8	673,3141	2	36,7			0	21	F21:3030	528	539	Oxidation (M)	M9:Oxidation (M):1000.00
14	5	P02671	K.TFP(+15.99)GFF.S	Y	36,55	730,3326	6	3,7	731,3426	1	38,1	5,26E+05			1	F1:3206	528	533	Oxidation or Hydroxylation	P3:Oxidation or Hydroxylation:1000.00
14	5	P02671	E.KVTSGSTTTTR.R	Y	36,04	1137,599	11	2,6	569,8082	2	21,8		1,40E+05	2,14E+05	19	F19:1739	448	458		
14	5	P02671	S.GIGTLDGFR.H	Y	35,68	934,4872	9	6,7	468,254	2	31,		3,10E+05		12	F12:254	502	510		

											3								4				
14	5	P02671	K.TFPGFFSPM(+15.99).L	Y	34,9	1045,4579	9	7,4	1046,4729	1	35,4	2,87E+06	2,94E+06	5,43E+05				11	F11:2875	528	536	Oxidation (M)	M9:Oxidation (M):1000.00
14	5	P02671	R.GSESGIFTN.T	Y	33,73	910,4032	9	7,2	911,417	1	29	7,19E+05						3	F3:2362	548	556		
14	5	P02671	K.TFPGFFSP(+15.99)MLG.E	Y	33,25	1215,5634	11	3,4	608,791	2	37			8,12E+04				20	F20:3045	528	538		P8:Oxidation or Hydroxylation:22.45
14	5	P02671	K.TFPGFFSPM(+15.99)L.G	Y	32,43	1158,542	10	4,5	580,2809	2	38	1,77E+05	1,49E+05	1,57E+05				2	F2:3085	528	537	Oxidation (M)	M9:Oxidation (M):1000.00
14	5	P02671	V.IGPDGHK.E	Y	31,45	722,3711	7	2,7	362,1938	2	18,5	0						4	F4:1520	470	476		
14	5	P02671	R.TGKEKVTSGSTTTTRR.S	Y	30,05	1708,9067	16	7,1	428,237	4	18,5			4,54E+04				21	F21:1464	444	459		
14	5	P02671	L.TDMPQMR.M	Y	27,43	877,3786	7	2,8	439,6978	2	29	6,81E+05						1	F1:2357	252	258		
14	5	P02671	R.HPDEAAFFDTA.S	Y	27,1	1219,5145	11	6,6	610,7686	2	31,3	1,24E+06						2	F2:2558	513	523		
14	5	P02671	K.TFPGFFSP(+15.99)ML.G	Y	26,4	1158,542	10	6,7	580,2822	2	37,6			2,52E+05				20	F20:3097	528	537		P8:Oxidation or Hydroxylation:46.87
14	5	P02671	K.TFPGF.F	Y	26,34	567,2693	5	5,4	568,2796	1	32,9			7,47E+05				20	F20:2726	528	532		
14	5	P02671	K.TVIGPD.G	Y	26,23	600,3119	6	3	601,321	1	28,9	2,78E+05						1	F1:2351	468	473		
14	5	P02671	R.GSESGIF.T	Y	26,18	695,3126	7	8,2	696,3256	1	30,3		5,44E+05					11	F11:2456	548	554		
14	5	P02671	K.TFPGFFSP.M	Y	25,91	898,4225	8	2,9	899,4324	1	39	5,92E+05						1	F1:3279	528	535		
14	5	P02671	K.NNKD(+15.99)SHSLTTNIMEILR(+31.99)GDFSSANNR(+31.99).D	Y	25,89	3013,3796	26	6,4	1507,7068	2	39,2				0			35	F35:2460	98	123		D4:Oxidation or Hydroxylation:0.00;R17:Dihydroxy:5.68;R26:Dihydroxy:9.45
40	8	P02675	E.AVSQTSSSSFQYMYLLK.D	Y	75,92	1938,9397	17	3,9	970,4809	2	37,2	4,71E+05						1	F1:3130	136	152		
40	8	P02675	K.QGFGNVATNTDGK.N	Y	72,6	1307,6106	13	1	654,8132	2	29,3	5,36E+05			3,90E+05	1,41E+05		1	F1:2387	301	313		
40	8	P02675	R.MGPTELLIEMEDWK.G	Y	68,43	1690,7946	14	5,6	846,4093	2	41,1	2,22E+05	0	0				1	F1:3449	335	348		
40	8	P02675	K.HGTDDGVVWMNWK.G	Y	67,56	1543,6877	13	11	772,8599	2	36,2	4,76E+05						1	F1:3035	459	471		
40	8	P02675	K.HQLYIDETVNSNIPTNLR.V	Y	59,19	2126,0757	18	5,8	1064,0513	2	35,3						0	76	F76:2128	179	196		
40	8	P02675	E.NGGWTVIQNR.Q	Y	58,01	1143,5785	10	1,6	572,7974	2	32,2	7,86E+05						1	F1:2669	276	285		
40	8	P02675	R.QDGSVDFGR.K	Y	53,85	979,4359	9	7,6	490,7289	2	26,5	9,35E+05		2,05E+06				3	F3:2152	286	294		
40	8	P02675	K.AHYGGFTVQNEANK.Y	Y	53,09	1534,7164	14	-14	768,3551	2	27,7		0					15	F15:2388	354	367		

40	8	P02675	K.ISQLTR.M	Y	46,78	716,4181	6	0,9	359,2166	2	19,1	0	0	0							24	F24:1541	329	334		
40	8	P02675	E.TVNSNIPTNLR.V	Y	45,83	1227,6571	11	3,2	614,8378	2	30,8	3,01E+06									1	F1:2535	186	196		
40	8	P02675	E.MYLIQPDSSVKPYR.V	Y	39,25	1695,8654	14	2,2	566,297	3	33,5	1,88E+06									1	F1:2797	254	267		
40	8	P02675	K.IRPFFPQQ	Y	38,97	1031,5552	8	7,8	516,7889	2	31,4	1,02E+06	7,47E+05								3	F3:2568	484	491		
40	8	P02675	E.ALLQQERPIR.N	Y	38,7	1222,7146	10	2,3	612,366	2	30,2	1,70E+06									1	F1:2470	115	124		
40	8	P02675	K.YQISVNK.Y	Y	38,19	850,4548	7	7,6	426,2379	2	26,7	0	2,89E+05								3	F3:2168	368	374		
40	8	P02675	K.EDGGGWWYNR.C	Y	37,75	1238,5105	10	4	620,265	2	0,52	4,85E+03									1	F1:40	427	436		
40	8	P02675	E.EAPSLRPAPPPISGGGYR.A	Y	32,17	1820,9532	18	2,4	607,9932	3	32		2,16E+06								10	F10:2653	55	72		
40	8	P02675	R.MGPTELLIE.M	Y	31,48	1001,5103	9	3,1	1002,5207	1	37,1	8,78E+05	5,49E+05								10	F10:3113	335	343		
63	15	P02679	K.YEASILTHDSSIR.Y	Y	80,25	1490,7365	13	3,3	746,3779	2	31,9	1,20E+06					1,15E+06			6,73E+05	1	F1:2644	122	134		
63	15	P02679	K.VGPEADKYR.L	Y	67,22	1033,5192	9	1	517,7674	2	24,3	8,94E+05		0							5	F5:2068	293	301		
63	15	P02679	E.IDGSGNGWTVFQK.R	Y	59,38	1407,6782	13	2,7	704,8483	2	34,2		1,52E+06								10	F10:2849	210	222		
63	15	P02679	R.TSTADYAMFK.V	Y	55,96	1133,5063	10	3,1	567,7622	2	32,5	9,74E+05									1	F1:2702	283	292		
63	15	P02679	K.QSGLYFIKPLK.A	Y	53,28	1292,7493	11	2,7	647,3837	2	35	3,68E+06	1,93E+06								1	F1:2930	189	199		
63	15	P02679	K.EGFGHLSPTGTTEFWLGNEK.I	Y	46,33	2206,033	20	1	736,3524	3	34,7	0	0								5	F5:3066	239	258		
63	15	P02679	K.AIQLTYNPDESSKPNMIDAATLK.S	Y	42,81	2519,2578	23	2	840,7616	3	35,1	1,07E+06									1	F1:2939	89	111		
63	15	P02679	Q.SGLYFIK(+27.99)PLK.A	Y	40,19	1192,6855	10	13	597,358	2	34,7			0							19	F19:3014	190	199		K7:Formylation:47.09
63	15	P02679	R.TSTADYAM(+15.99)FK.V	Y	38,45	1149,5012	10	6,9	575,7618	2	27,8	3,05E+05									3	F3:2260	283	292	Oxidation (M)	M8:Oxidation (M):1000.00
63	15	P02679	R.VELEDWNGR.T	Y	38,22	1116,52	9	-14	559,2598	2	30,5		0	0							15	F15:2657	274	282		
63	15	P02679	K.EGFGHLSPTGTTE.F	Y	36,53	1331,5994	13	3,4	666,8092	2	31,8	9,54E+05									1	F1:2633	239	251		
63	15	P02679	E.ASILTHDSSIR.Y	Y	34,56	1198,6306	11	2,3	400,5517	3	30	4,23E+05									1	F1:2451	124	134		
3	4	P02751	R.HTSVQTTSSGSGPFTDVR.A	Y	115,7	1862,8759	18	7,5	932,4522	2	27,8	6,35E+07	1,04E+08	2,07E+07			0		5,66E+04		2	F2:2259	273	290		
3	4	P02751	K.TETITGFQVDAVPANGQTPIQR.T	Y	108,6	2342,1865	22	11	1172,1139	2	32,6	1,36E+06	0	4,32E+06	2,18E+06	1,78E+06	3,71E+06	3,46E+06	3,32E+06	4,70E+06	2	F2:2660	1838	1859		
3	4	P02751	K.EINLAPDSSSVVVSGLM(+15.99)VATK.Y	Y	105,2	2132,1035	21	13	1067,0731	2	34,6	2,64E+05	7,15E+05				1,47E+06		5,62E+05	3,18E+06	11	F11:2809	1767	1787	Oxidation (M)	M17:Oxidation (M):1000.0

																										0
3	4	P02751	R.TKTETITGFQVDAVPANGQTPIQR.T	Y	101,4	2571,3293	24	-8	1286,6616	2	32,2	5,88E+06	0	6,10E+06							6	F6:2836	1836	1859		
3	4	P02751	R.PAQGVVTTLENVSPPR.R	Y	97,49	1663,8893	16	10	832,9602	2	31,7	9,50E+06	8,02E+06								2	F2:2591	1803	1818		
3	4	P02751	R.EESPLLIGQQSTVSDVPR.D	Y	96,28	1954,0007	18	8,7	978,0162	2	32,2	1,41E+07	8,20E+06	4,77E+06	2,20E+06	8,10E+05	2,05E+06	1,59E+06	1,02E+06	2,33E+06	2	F2:2631	1435	1452		
3	4	P02751	R.RPGGEPSPEGTTGQSYNQY.S	Y	94,85	2023,8871	19	8,2	1012,9591	2	27,3	9,26E+05	2,57E+06								11	F11:2195	2335	2353		
3	4	P02751	R.NLQPASEYTVSLVAIK.G	Y	94,17	1731,9407	16	-1,1	866,9766	2	34,5	5,22E+06	3,32E+05	2,44E+06							4	F4:3044	1055	1070		
3	4	P02751	R.FLATTPNSLLVSWQPPR.A	Y	91,79	1926,0363	17	-4,4	964,0212	2	36,7	7,72E+06	2,95E+07	1,85E+07							15	F15:3215	1911	1927		
3	4	P02751	K.FTQVTPTSLSAQWTPPNVQLTGYR.V	Y	90,92	2691,3657	24	-2,7	1346,6865	2	36,1	0	0	0							5	F5:3188	1730	1753		
3	4	P02751	R.TEIDKPSQM(+15.99)QVTDVQDNSISVK.W	Y	90,88	2477,1956	22	9,3	826,7468	3	28,8	1,40E+06	9,43E+05								3	F3:2347	1540	1561	Oxidation (M)	M9:Oxidation (M):1000.00
3	4	P02751	R.RPHETGGYMLEC(-33.99)VC(+31.99)LGNGK.G	Y	90,88	2060,9407	19	1,9	1031,4796	2	34,3	1,10E+07	6,50E+06	2,48E+06							16	F16:2029	156	174		C12:Dehydroalanine (C):35.51;C14:Dihydroxy:35.51
3	4	P02751	R.RPGGEPSPEGTTGQSYNQYSQR.Y	Y	90,6	2395,0789	22	8,5	799,3737	3	26,3	1,34E+07	1,16E+07								2	F2:2131	2335	2356		
3	4	P02751	R.VPGTSTSATLTGLTR.G	Y	89,69	1460,7834	15	8,9	731,4055	2	30,1	5,01E+07	2,57E+07	1,96E+07	3,67E+07	2,11E+07	3,35E+07	3,61E+07	3,15E+07	3,78E+07	11	F11:2441	2150	2164		
3	4	P02751	R.GDSPASSKPISINYR.T	Y	88,11	1590,8002	15	3,5	796,4102	2	28,9	6,76E+06	8,54E+06	4,43E+06	6,20E+05	1,13E+06	2,22E+06	1,28E+06	4,29E+05	1,82E+06	13	F13:2451	1525	1539		
3	4	P02751	R.ITYGETGGNSPVQEFTVPGSK.S	Y	88,03	2167,0432	21	0	1084,5289	2	32,9	1,54E+07	1,60E+07	8,92E+06	0		6,84E+05				6	F6:2903	1480	1500		
3	4	P02751	R.SYTITGLQPGTDYK.I	Y	87,95	1542,7566	14	9,6	772,3929	2	30,7	2,03E+07	2,25E+07	1,45E+07						0	2	F2:2505	1867	1880		
3	4	P02751	K.HYQINQQWER.T	Y	86,12	1400,6586	10	-1,8	701,3353	2	28,3	1,62E+08	1,68E+08	1,13E+08	2,01E+08	1,26E+08	2,30E+08	1,81E+08	1,55E+08	1,39E+08	13	F13:2395	58	67		
3	4	P02751	R.PRPGVTEATITGLEPGTEYTIYVIALK.N	Y	85,73	2888,5535	27	-0,3	963,8582	3	37,4	2,97E+07	1,46E+07	1,06E+07							6	F6:3301	1951	1977		
3	4	P02751	R.SSPVVIDASTAIDAPSNLR.F	Y	83,08	1911,9901	19	0,2	957,0026	2	33,6	3,14E+07	2,22E+07	2,49E+07							13	F13:2896	1892	1910		
3	4	P02751	E.VVAATPTSLLISWDAPAVTVR.Y	Y	82,59	2166,2048	21	6,4	1084,1166	2	40,3	6,12E+05									1	F1:3386	1456	1476		
3	4	P02751	K.LGVRPSQGGEAPR.E	Y	82,17	1322,7054	13	1	662,3607	2	25,5	8,41E+06	6,92E+06	4,53E+06	1,17E+05	2,97E+05	9,95E+04	5,47E+04	6,97E+04	5,33E+05	13	F13:2120	1117	1129		
3	4	P02751	K.TYHVGEQWQK.E	Y	82,03	1274,6044	10	-1,2	638,3087	2	27,5	2,09E+07	1,88E+07	3,42E+06	1,33E+07	5,12E+06	7,47E+06	9,14E+06	4,32E+06	7,08E+06	13	F13:2322	2301	2310		
3	4	P02751	K.HYQINQQW(+31.99)ER.T	Y	80,57	1432,6483	10	6,9	717,3364	2	25,7	1,25E+06	2,82E+06								2	F2:2074	58	67		W8:Dihydroxy:30.36
3	4	P02751	K.HYQINQQW(+15.99)ER.T	Y	78,93	1416,6534	10	7,9	709,3396	2	26,7	1,84E+07	1,40E+07	2,85E+06	2,08E+06	4,21E+05	1,76E+06	3,56E+06	2,29E+06	5,15E+05	2	F2:2164	58	67		W8:Oxidation (HW):186.64
3	4	P02751	K.YEVSVYALK.D	Y	78,39	1070,564	9	-1,	536,2889	2	31,	2,53E+06	2,75E+06								15	F15:275	1788	1796		

						8		5			5											6				
3	4	P02751	K.GLKPGVVYEGQLISIQQYGHQEVTR.F	Y	77,74	2798,4714	25	-5,1	933,8263	3	32,8	0	0								15	F15:2868	670	694		
3	4	P02751	K.TYHVGEQW(+15.99)QK.E	Y	75,2	1290,5992	10	5,6	646,3105	2	26,2	9,32E+05	2,06E+06								2	F2:2117	2301	2310		W8:Oxidation (HW):130.10
3	4	P02751	R.VTWAPPPSIDLTNFLVR.Y	Y	74,11	1925,041	17	2,1	963,5298	2	39,7	2,46E+06	0	1,23E+06							4	F4:3500	1285	1301		
3	4	P02751	K.IYLYTLNDNAR.S	Y	72,91	1354,6881	11	2,4	678,353	2	34,3	8,67E+06	9,23E+06	2,66E+06			2,69E+05			9,20E+05	1	F1:2870	1881	1891		
3	4	P02751	R.RPGGEPSPEGTTGQ.S	Y	70,96	1368,627	14	3,1	685,3229	2	23,6	5,95E+05	1,13E+06	1,83E+05	1,66E+04			0			24	F24:1955	2335	2348		
3	4	P02751	K.HYQINQ(+.98)QWER.T	Y	70,6	1401,6426	10	2,9	701,8306	2	28,7	4,73E+06	4,27E+06	1,32E+06			5,20E+05			1,83E+06	13	F13:2431	58	67		Q6:Deamidation (NQ):16.90
3	4	P02751	R.STTPDITGYR.I	Y	69,77	1109,5353	10	-1,5	555,7741	2	28,8	4,00E+06	0	3,45E+06						4,07E+04	15	F15:2493	1198	1207		
3	4	P02751	R.VGDTYERPK.D	Y	69,72	1063,5298	9	-0,3	532,772	2	17,7	1,20E+08	1,33E+08	6,83E+07	9,27E+05	7,92E+05	1,60E+06	8,84E+05	5,40E+05	3,65E+06	23	F23:1431	108	116		
3	4	P02751	K.VFAVSHGR.E	Y	68,48	871,4664	8	-0,2	436,7404	2	22,4	7,98E+05	7,30E+04	7,68E+04							5	F5:1886	977	984		
3	4	P02751	R.NSITLTNLTPGTEYVVSIVALNGR.E	Y	68,42	2531,3594	24	0,4	1266,6875	2	39,2	0	0				0			1,86E+05	4	F4:3455	1411	1434		
3	4	P02751	R.IGDTWSK.K	Y	68,25	805,397	7	-0,6	403,7055	2	26,5	7,05E+07	7,03E+07	4,66E+07							15	F15:2271	242	248		
3	4	P02751	R.RPGGEPSPEGTTGQSYN.Q	Y	68,23	1732,7653	17	6,7	867,3957	2	29,2	3,01E+05	2,40E+05	3,24E+04							26	F26:1630	2335	2351		
3	4	P02751	R.HTSVQTTSSGSGP.F	Y	67,47	1244,5634	13	5,8	623,2925	2	22,8	6,39E+05	7,77E+05								2	F2:1830	273	285		
3	4	P02751	N.SLLVSWQPPR.A	Y	66,58	1181,6556	10	7,7	591,8397	2	33,2	1,95E+06	1,36E+06								3	F3:2715	1918	1927		
3	4	P02751	R.VGDTY(+15.99)ERPK.D	Y	66,21	1079,5247	9	-1,3	540,7689	2	18,1	4,61E+05	5,89E+05								13	F13:1431	108	116		Y5:Oxidation or Hydroxylation:26.31
3	4	P02751	K.YEKPGSPPR.E	Y	65,88	1029,5243	9	1,2	515,77	2	16,7	6,95E+06	4,13E+06	1,54E+06							24	F24:1324	1937	1945		
3	4	P02751	R.TYLGNALVCT(-2.02)CYGGSR.G	Y	65,55	1674,7494	16	-0,4	838,3817	2	33,2	2,66E+07	1,82E+07	2,48E+07	4,83E+07	3,52E+07	1,05E+07	2,86E+07	3,56E+07	6,70E+06	22	F22:2876	68	83		T10:2-amino-3-oxo-butanoic_acid:9.40
3	4	P02751	R.AQITGYR.L	Y	65,04	807,4239	7	-0,9	404,7189	2	25,2	1,64E+06	0	0							6	F6:2161	1022	1028		
3	4	P02751	Q.TTSSGSGPFTDVR.A	Y	64,5	1310,6102	13	7,8	656,3175	2	28,5		2,59E+06								12	F12:2302	278	290		
3	4	P02751	R.IGDTW(+15.99)SK.K	Y	64,3	821,3919	7	4,8	411,7052	2	25,2	1,31E+07	1,73E+07	7,19E+05							2	F2:2036	242	248	Oxidation (HW)	W5:Oxidation (HW):1000.00
3	4	P02751	R.IGDTWSKK.D	Y	64,18	933,4919	8	0,5	467,7535	2	20,9		0	5,39E+04							23	F23:1723	242	249		
3	4	P02751	S.HYAVGDEWER.M	Y	64,09	1260,5524	10	-0,2	631,2833	2	28,4	0	0	1,69E+06							22	F22:2415	2214	2223		
3	4	P027	R.IGDQWDK.Q	Y	63,	860,	7	-	431,	2	2	1,42E+07	5,81E+06	0							4	F4:2	480	486		

		51			54	4028		0,8	2083		7,3											369				
3	4	P02751	K.DTLTSRPAQGVVTTLENVSPPRR.A	Y	63,42	2493,3298	23	-1,7	832,1158	3	32,9	0	0	2,12E+06							6	F6:2904	1797	1819		
3	4	P02751	K.HYQ(+.98)INQ(+.98)QWER.T	Y	63,28	1402,6266	10	-1,5	702,3195	2	29,2	2,10E+06	1,36E+06	0							6	F6:2553	58	67		Q3:Deamidation (NQ):60.92;Q6:Deamidation (NQ):30.46
3	4	P02751	K.QHDMGHMMR.C	Y	63,01	1141,458	9	0,8	571,7368	2	18,2	1,54E+06	5,40E+05	9,81E+05							24	F24:1456	487	495		
3	4	P02751	K.TYHVGEQW(+31.99)QK.E	Y	62,95	1306,5942	10	8,4	654,3099	2	25,5		2,91E+05								12	F12:2044	2301	2310		W8:Dihydroxy:39.76
3	4	P02751	R.GNLLQCICT(-2.02)GNGR.G	Y	62,08	1345,623	13	6,3	673,8231	2	31,1	4,38E+06	3,93E+06	7,16E+05	2,18E+06	2,12E+06	7,32E+05	6,25E+06	8,92E+05	6,25E+05	2	F2:2540	253	265	2-amino-3-oxo-butanoic_acid	T9:2-amino-3-oxo-butanoic_acid:1000.00
3	4	P02751	K.LLC(-33.99)QC(+31.99)LGFGSGHFR.C	Y	62,07	1534,7351	14	-12	512,5793	3	33,6	3,31E+06	1,28E+06								13	F13:2891	2231	2244		C3:Dehydroalanine (C):46.20;C5:Dihydroxy:22.34
3	4	P02751	V.PGTSTSATLTGLTR.G	Y	61,86	1361,7151	14	7,6	681,87	2	33,6			1,76E+06		2,99E+06	2,99E+06		5,51E+06		68	F68:1961	2151	2164		
3	4	P02751	R.ATITGYR.I	Y	61,82	780,413	7	-0,8	391,2134	2	25,3	1,33E+06	1,58E+06	1,05E+06							23	F23:2138	1383	1389		
3	4	P02751	R.TYLGNALVC(+31.99)TC(-33.99)YGGSR.G	Y	61,32	1674,7672	16	-1,5	838,3896	2	35,7	1,36E+06				8,58E+06		8,77E+06		1,32E+06	63	F63:2183	68	83		C9:Dihydroxy:0.00;C11:Dehydroalanine (C):0.00
3	4	P02751	R.DAPIVNK.V	Y	60,68	755,4177	7	1,6	756,4262	1	20,4	5,58E+06	2,74E+06	1,80E+06							23	F23:1679	1163	1169		
3	4	P02751	K.HYQIN(+.98)QQWER.T	Y	60,49	1401,6426	10	4,6	701,8318	2	28,6			1,53E+06					7,76E+04		22	F22:2432	58	67		N5:Deamidation (NQ):16.90
3	4	P02751	Y.TVSHYAVGDEWER.M	Y	59,77	1547,7004	13	-1,2	774,8566	2	29,4	0	0								13	F13:2499	2211	2223		
3	4	P02751	K.DSMIWDCT(-2.02)CIGAGR.G	Y	59,75	1524,616	14	5,8	763,3197	2	36,9	2,31E+07	1,11E+07	2,73E+07							8	F8:2234	117	130		T8:2-amino-3-oxo-butanoic_acid:69.64
3	4	P02751	R.GNLLQC(-33.99)IC(+31.99)TGNGR.G	Y	59,57	1345,6409	13	-4,6	673,8246	2	34,6	3,89E+06	1,75E+06		1,81E+07	6,29E+06	4,56E+06	1,22E+07	6,11E+06	3,72E+06	51	F51:2035	253	265		C6:Dehydroalanine (C):28.86;C8:Dihydroxy:28.86
3	4	P02751	R.ISC(+15.99)TIANRCHEGGQSYK.I	Y	59,44	1881,8462	17	3,7	941,9339	2	27,2	1,34E+06	1,10E+06	1,83E+06	1,54E+06	2,02E+05	1,84E+05	1,21E+06	1,09E+06	1,50E+05	58	F58:1478	133	149		C3:Oxidation or Hydroxylation (C):11.86
3	4	P02751	R.MSESGFK.L	Y	59,42	784,3425	7	-0,5	785,3494	1	21,4	1,07E+07	2,96E+06	3,96E+06							23	F23:1767	2224	2230		
3	4	P02751	K.YTGNTYR.V	Y	59,27	873,3981	7	6,5	437,7092	2	19,9	1,92E+07	1,51E+07	6,74E+06							11	F11:1576	101	107		
3	4	P02751	R.TYLGNALVC(-33.99)TC(+31.99)YGGSR.G	Y	58,98	1674,7672	16	0,6	838,3914	2	32,4	1,75E+06	1,09E+07	1,22E+06	2,12E+07	1,02E+07	4,90E+06	3,55E+07	1,42E+07	3,06E+06	11	F11:2636	68	83		C9:Dehydroalanine (C):14.63;C11:Dihydroxy:14.63

3	4	P02751	K.HYQ(+.98)INQQWER.T	Y	58,98	1401,6426	10	-1,3	701,8276	2	28,5		1,20E+06				1,07E+06			1,09E+06	14	F14:2440	58	67		Q3:Deamidation (NQ):15.64
3	4	P02751	V.GDTYERPK.D	Y	58,76	964,4614	8	-1,6	483,2372	2	17	1,56E+06	1,33E+06	1,14E+06	8,52E+04		0	0		0	24	F24:1354	109	116		
3	4	P02751	R.RPGGEPSPEGTT.G	Y	58,49	1183,5469	12	-1,3	1184,5526	1	21,3	3,00E+06	1,20E+06	1,06E+06							6	F6:1788	2335	2346		
3	4	P02751	K.WLPSSSPVTGYR.V	Y	58,41	1348,6775	12	-5,7	675,3422	2	31,7	7,88E+06	7,74E+06	8,06E+06							13	F13:2718	1562	1573		
3	4	P02751	K.DTLTSRPAQGVVTTLENVSPPR.R	Y	58,09	2337,2288	22	6,8	780,0888	3	32,9	1,10E+06	1,99E+06	0							11	F11:2672	1797	1818		
3	4	P02751	K.DSM(+15.99)IWDCT(-2.02)CIGAGR.G	Y	58,08	1540,6108	14	5,7	771,3171	2	36		1,99E+06		2,90E+07	9,55E+06		1,73E+07	1,92E+06		42	F42:2163	117	130	Oxidation (M)	M3:Oxidation (M):1000.00;T8:2-amino-3-oxo-butanoic_acid:90.57
3	4	P02751	R.RPHETGGYM(+15.99)LECVCLGNGK(-2.02).G	Y	57,59	2076,918	19	6,4	693,3177	3	33	4,51E+05			2,29E+07	7,47E+06	6,06E+05	2,15E+07	1,04E+07		34	F34:1942	156	174	Oxidation (M)	M9:Oxidation (M):1000.00;K19:2-amino-3-oxo-butanoic_acid:81.27
3	4	P02751	T.SSGSGPFTDVR.A	Y	57,34	1108,5149	11	6,7	555,2684	2	27,3			1,28E+07							20	F20:2230	280	290		
3	4	P02751	S.SGSGPFTDVR.A	Y	56,87	1021,4828	10	5,6	511,7516	2	27,9	1,03E+07	1,18E+07	1,31E+07							2	F2:2263	281	290		
3	4	P02751	R.IGDQWDKQHD.M	Y	56,8	1240,5472	10	2,6	621,2825	2	27,8			1,07E+06							19	F19:2313	480	489		
3	4	P02751	R.N(+.98)LQPASEYTVSLVAIK.G	Y	56,64	1732,9247	16	-3,2	867,4669	2	34,3			0							22	F22:2976	1055	1070		N1:Deamidation (NQ):14.04
3	4	P02751	K.YVHGVR.Y	Y	56	729,3922	6	0,2	365,7035	2	14,3	8,22E+05	6,79E+04	0							4	F4:1112	579	584		
3	4	P02751	K.DSM(+15.99)IWDC(-33.99)TC(+31.99)IGAGR.G	Y	55,86	1540,6287	14	1,8	771,323	2	35,7	4,64E+06			1,18E+06						36	F36:2171	117	130	Oxidation (M)	M3:Oxidation (M):1000.00;C7:Dehydroalanine (C):18.53;C9:Dihydroxy:18.53
3	4	P02751	K.IGDTWR.R	Y	55,74	746,3711	6	-0,3	374,1927	2	27	1,03E+08	9,37E+07	7,28E+07							15	F15:2320	150	155		
3	4	P02751	R.TYLGNALVCTCY(-2.02)GGSR.G	Y	55,66	1674,7494	16	6,6	838,3875	2	36		3,56E+06			6,58E+04				2,23E+06	73	F73:2178	68	83		Y12:2-amino-3-oxo-butanoic_acid:0.00
3	4	P02751	K.SEPLIGR.K	Y	55,61	770,4286	7	0,2	386,2216	2	27,3	1,19E+07	1,02E+07	1,15E+07							15	F15:2349	1982	1988		
3	4	P02751	R.ESKPLTAQQTTK.L	Y	55,46	1330,7092	12	8,6	666,3676	2	23	1,51E+05	3,13E+05								3	F3:1853	985	996		
3	4	P02751	R.HRPRPYPPNVGEEIQIGHIPR.E	Y	54,82	2461,3091	21	5,3	616,3378	4	31,4	6,69E+05	6,67E+05								12	F12:2556	2071	2091		
3	4	P02751	R.RTTPPTTATPIR.H	Y	54,64	1310,7306	12	4,3	656,3754	2	30,1					7,98E+04	2,02E+05		7,64E+04		51	F51:1670	2059	2070		

3	4	P02751	K.HYQINQQWER(+15.99).T	Y	54,36	1416,6534	10	9,2	709,3405	2	29,4					0					40	F40:1631	58	67		R10:Oxidation or Hydroxylation:29.96
3	4	P02751	R.ISCTIANRC(+15.99)HEGGQSYK.I	Y	54,24	1881,8462	17	7,5	941,9374	2	27,5		1,10E+05	0	9,82E+04				2,19E+05	0	70	F70:1513	133	149		C9:Oxidation or Hydroxylation (C):1.99
3	4	P02751	K.D(+15.99)SMIWDCT(-2.02)CIGAGR.G	Y	53,98	1540,6108	14	5,7	771,3171	2	35,9				1,63E+07	1,41E+07	3,48E+06	7,20E+06	9,25E+06		61	F61:2120	117	130		D1:Oxidation or Hydroxylation:96.93; T8:2-amino-3-oxo-butanoic_acid:90.57
3	4	P02751	K.EATIPGHLNSYTIK.G	Y	53,63	1542,8042	14	-4,7	772,4058	2	30,6	0									4	F4:2681	656	669		
3	4	P02751	R.PYPPNVGEEIQIGHIPR.E	Y	53,57	1914,9951	17	6,9	639,3434	3	32,1	2,87E+06	3,07E+06								3	F3:2627	2075	2091		
3	4	P02751	Q.HDMGHMMR.C	Y	53,51	1013,3994	8	0	338,8071	3	17,7			5,42E+04							23	F23:1429	488	495		
3	4	P02751	R.M(+15.99)SESGFK.L	Y	53,41	800,3375	7	-1,3	401,1755	2	14,3	3,25E+06	1,93E+06	1,03E+06							6	F6:1122	2224	2230	Oxidation (M)	M1:Oxidation (M):1000.00
3	4	P02751	K.FGFCPM(+31.99)AAHEEIC(-33.99)TTNEGVMYR.I	Y	52,9	2503,0605	22	0,8	835,3615	3	34,2			0							23	F23:2999	458	479		M6:Dioxidation (M):110.51;C13:Dehydroalanine (C):13.05
3	4	P02751	D.HTVLVQTR.G	Y	52,86	952,5454	8	2,4	477,2811	2	27,5	8,10E+05	1,75E+06								10	F10:2217	404	411		
3	4	P02751	R.ISCTIANR(+15.99)CHEGGQSYK.I	Y	52,75	1881,8462	17	1,8	941,9321	2	24,2		2,22E+05	1,14E+06	2,37E+06	1,94E+06	1,00E+05	7,88E+05	1,12E+05	9,70E+04	13	F13:1995	133	149		R8:Oxidation or Hydroxylation:0.00
3	4	P02751	R.QAQQMVQPQSPVAVSQ(+.98)SK(-1.03).P	Y	52,74	1939,9309	18	8,2	970,9807	2	32,1				1,59E+07	1,10E+07		1,10E+07	7,92E+06		33	F33:1857	32	49	Lysine oxidation to aminoadipic semialdehyde	Q16:Deamidation (NQ):130.36;K18:Lysine oxidation to aminoadipic semialdehyde:1000.00
3	4	P02751	R.HTSVQTT.S	Y	52,44	772,3715	7	-0,2	773,3787	1	11,5	8,92E+05	1,72E+05	0							14	F14:819	273	279		
3	4	P02751	R.RPHETGGYMLE.C	Y	52,37	1288,587	11	1,4	645,3017	2	30,6	1,08E+06									1	F1:2511	156	166		
3	4	P02751	R.TIKPDVR.S	Y	52,36	827,4865	7	0,2	414,7506	2	17,5	8,60E+06	1,02E+07	8,24E+06							24	F24:1399	1860	1866		
3	4	P02751	R.ISCTIAN(+15.99)RCHEGGQSYK.I	Y	52,23	1881,8462	17	3,6	941,9337	2	27,1	3,93E+05	6,76E+05	3,53E+05	7,63E+05	1,60E+06	1,25E+05	3,42E+06	1,98E+06		60	F60:1468	133	149		N7:Oxidation or Hydroxylation:0.00
3	4	P02751	R.IGDTW(+13.98)SK.K	Y	52,09	819,3763	7	5,5	410,6977	2	26,9	1,26E+06	1,84E+06								11	F11:2165	242	248	Tryptophan oxidation to oxolacton	W5:Tryptophan oxidation to oxolactone

																								e	:1000.00
3	4	P02751	K.HYQ(+.98)INQ(+.98)Q(+.98)WER.T	Y	51,91	1403,6106	10	-3,5	702,8101	2	29,6	0								5	F5:2592	58	67		Q3:Deamidation (NQ):60.92;Q6:Deamidation (NQ):27.96;Q7:Deamidation (NQ):55.92
3	4	P02751	R.VGDTYERP.K	Y	51,49	935,4348	8	-0,9	468,7243	2	25	7,86E+06	9,10E+06	8,46E+06						24	F24:2097	108	115		
3	4	P02751	R.RPHETGGYMLEC(+47.98)VC(-33.99)LGNGK.G	Y	51,37	2076,9358	19	2,6	1039,4779	2	32,9				6,25E+06	5,46E+06	0	2,93E+06	8,84E+06	29	F29:1929	156	174		C12:Cysteine oxidation to cysteic acid:18.53;C14:Dehydroalanine (C):18.53
3	4	P02751	R.ITGYIIK.Y	Y	51,34	806,4902	7	-0,7	404,2521	2	29,6	1,90E+06	8,31E+05	3,20E+06						6	F6:2591	1930	1936		
3	4	P02751	R.RPGGEPSPEGTTG.Q	Y	50,98	1240,5684	13	2,7	621,2932	2	27,1	0	0	0				0		18	F18:1461	2335	2347		
3	4	P02751	R.GFNCESKPEAEET(-2.02)CFDKYTGNTYR.V	Y	50,44	2786,1587	24	-1,2	929,7257	3	30,6	1,99E+07	3,86E+07							14	F14:2643	84	107		T13:2-amino-3-oxo-butanoic_acid:0.00
3	4	P02751	Y.AVGDEWER.M	Y	49,98	960,4301	8	-5,1	481,2198	2	27,9	8,07E+05	7,15E+05	0						22	F22:2369	2216	2223		
3	4	P02751	R.VGDTYER.P	Y	49,59	838,3821	7	4	420,2	2	21,6	4,91E+05	9,79E+05							2	F2:1733	108	114		
3	4	P02751	R.MS(-2.02)ESGFK.L	Y	49,5	782,3268	7	0,3	392,1708	2	14,8	0								4	F4:1155	2224	2230		S2:2-amino-3-oxo-butanoic_acid:67.96
3	4	P02751	R.IGDTW(+31.99)SK.K	Y	49,43	837,3868	7	4,9	419,7027	2	22,8		0							12	F12:1812	242	248		W5:Dihydroxy:41.83
3	4	P02751	R.ISCTIAN(+15.99)RCHEGGQ(+.98)SYK.I	Y	48,97	1882,8302	17	1,2	942,4235	2	24,5			5,49E+05						24	F24:2039	133	149	Deamidation (NQ)	N7:Oxidation or Hydroxylation:0.00;Q14:Deamidation (NQ):1000.00
3	4	P02751	R.ISCTIANR(+15.99)CHEGGQ(+.98)SYK.I	Y	48,92	1882,8302	17	-0,9	942,4216	2	24,9	4,53E+05	0	0						4	F4:2132	133	149		R8:Oxidation or Hydroxylation:0.00;Q14:Deamidation (NQ):83.89
3	4	P02751	K.FGFC(-33.99)PMAAHEEIC(+31.99)TTNEGVMYR.I	Y	48,9	2503,0605	22	-13	835,3499	3	34,4	0	2,39E+06							5	F5:3042	458	479		C4:Dehydroalanine (C):10.65;C13:Dihydroxy:10.65
3	4	P02751	R.M(+15.99)S(-2.02)ESGFK.L	Y	48,76	798,3218	7	6,5	400,1707	2	25,3	1,20E+06	2,24E+06							12	F12:2027	2224	2230	Oxidation (M)	M1:Oxidation (M):1000.00;S2:2-amino-3-oxo-butanoic_acid:55.92

3	4	P02751	R.ISC(+15.99)TIANRCHEGGQ(+.98)SYK.I	Y	48,38	1882,8302	17	-1,2	942,4213	2	24,7	2,59E+05	1,59E+06	1,03E+06						14	F14:2072	133	149		C3:Oxidation or Hydroxylation (C):6.92;Q14:Deamidation (NQ):83.89
3	4	P02751	R.LTVGLTR.R	Y	48,15	758,465	7	-0,8	380,2395	2	28,8	0	0	0						23	F23:2489	1029	1035		
3	4	P02751	F.DKYTGNTYR.V	Y	47,86	1116,52	9	-3,4	559,2654	2	18,3	1,78E+05	0	0						14	F14:1466	99	107		
3	4	P02751	R.GATY(-2.02)N(+15.99)VIVEALKDQQR.H	Y	47,63	1817,9271	16	13	909,9828	2	34,4		0							15	F15:3013	2165	2180		Y4:2-amino-3-oxo-butanoic_acid:17.01;N5:Oxidation or Hydroxylation:93.60
3	4	P02751	T.GLQPGTDYK.I	Y	47,57	977,4818	9	0	489,7482	2	26,5	0		0						22	F22:2224	1872	1880		
3	4	P02751	R.TYLGNALVC(+31.99)TCYGGS(-2.02)R.G	Y	47,39	1706,7393	16	-0,9	854,3762	2	33	0								2	F2:2693	68	83		C9:Dihydroxy:0.00;S15:2-amino-3-oxo-butanoic_acid:0.00
3	4	P02751	R.QGENGQMMS(-2.02)CTC(+31.99)LGNGK.G	Y	47,01	1786,6743	17	9,6	894,353	2	29,1					5,76E+05		2,47E+05	3,37E+05	44	F44:1614	2267	2283		S9:2-amino-3-oxo-butanoic_acid:11.50;C12:Dihydroxy:35.36
3	4	P02751	R.TYLGN.A	Y	46,42	566,27	5	5,5	567,2804	1	25,1	8,98E+06	9,27E+06							2	F2:2025	68	72		
3	4	P02751	R.TYLGNALVC(-33.99)TC(+47.98)YGGSR.G	Y	46,19	1690,7621	16	0,2	846,3885	2	32,4		5,27E+05							12	F12:2641	68	83		C9:Dehydroalanine (C):13.67;C11:Cysteine oxidation to cysteic acid:13.67
3	4	P02751	R.EVVPRPR.P	Y	45,71	851,4977	7	3	426,7574	2	21,5			4,96E+04						22	F22:1745	1946	1952		
3	4	P02751	R.HHPEHFSGRPR.E	Y	45,22	1355,6595	11	-0,9	452,8934	3	12,1	0	2,02E+05							5	F5:910	1392	1402		
3	4	P02751	R.VGDTY(+31.99)ERPK.D	Y	45,16	1095,5197	9	-0,7	548,7667	2	18,1	0	1,10E+05							13	F13:1435	108	116		Y5:Dihydroxy:0.00
3	4	P02751	K.IGDTW(+15.99)R.R	Y	45,08	762,366	6	5,3	382,1923	2	25,7	5,69E+06	8,31E+06							2	F2:2080	150	155	Oxidation (HW)	W5:Oxidation (HW):1000.00
3	4	P02751	R.GFNC(-33.99)ESKP(+31.99)EAEETCFDKYTGNTYR.V	Y	45,06	2786,1765	24	-2,9	929,7301	3	30,5	1,99E+07								6	F6:2683	84	107		C4:Dehydroalanine (C):7.40;P8:Dihydroxy:0.00
3	4	P02751	R.QGENGQMMSCT(-2.02)C(+31.99)LGNGK.G	Y	44,63	1786,6743	17	9,4	894,3528	2	28,8				2,65E+05					36	F36:1597	2267	2283		T11:2-amino-3-oxo-butanoic_acid:9.40;C12:Dihydroxy:14.04

3	4	P02751	R.QYNVGPSVSK.Y	Y	44,6	1077,5454	10	6,3	539,7834	2	26,5	2,32E+06	1,19E+06							3	F3:2145	1041	1050	
3	4	P02751	R.RPHETGGY(-2.02)MLECVC(+15.99)LGNGK.G	Y	44,45	2076,918	19	9,3	1039,476	2	33,3					1,40E+06				40	F40:1948	156	174	Y8:2-amino-3-oxo-butanoic_acid:9.42;C14:Oxidation or Hydroxylation (C):0.00
3	4	P02751	L.YTLNDNAR.S	Y	44,28	965,4567	8	7,4	483,7392	2	25,2			1,01E+06						21	F21:2032	1884	1891	
3	4	P02751	R.QGENGQMM(+31.99)SC(-33.99)TC(+31.99)LGNGK.G	Y	44,13	1786,692	17	0,1	894,3533	2	29				9,17E+04			3,43E+05		63	F63:1620	2267	2283	M8:Dioxidation (M):0.00;C10:Dehydroalanine (C):35.36;C12:Dihydroxy:35.36
3	4	P02751	R.TYLGNALVC(+15.99)T(-2.02)CYGGSR.G	Y	43,53	1690,7443	16	7,9	846,386	2	31,9	4,74E+05								3	F3:2609	68	83	C9:Oxidation or Hydroxylation (C):0.00;T10:2-amino-3-oxo-butanoic_acid:11.10
3	4	P02751	F.QVDAVPANGQTPIQR.T	Y	43,4	1592,827	15	-1,3	797,4197	2	28,8	0	0	0						13	F13:2445	1845	1859	
3	4	P02751	R.GFNCES(-2.02)KPEAEETCFDKYTGNTYR.V	Y	43,05	2786,1587	24	1,9	929,7286	3	30,6	1,89E+07	1,40E+07							13	F13:2616	84	107	S6:2-amino-3-oxo-butanoic_acid:1.11
3	4	P02751	K.PYQGWM(+31.99)MVDCT(-2.02)CLGEGSGR.I	Y	42,67	2118,8267	19	8,2	1060,4293	2	35,4								6,83E+05	66	F66:2139	204	222	M6:Dioxidation (M):0.00;T11:2-amino-3-oxo-butanoic_acid:41.57
3	4	P02751	R.VGDTYERP(+31.99)K.D	Y	42,51	1095,5197	9	-3,8	548,765	2	17,6		9,38E+04							15	F15:1409	108	116	P8:Dihydroxy:0.00
3	4	P02751	R.QAQQMVQPQSPVAVSQSK.P	Y	42,5	1939,9785	18	-15	970,9824	2	32,5							1,16E+06	1,12E+06	70	F70:1880	32	49	
3	4	P02751	K.IGDTW(+31.99)R.R	Y	42,14	778,361	6	5,5	390,1899	2	23	3,21E+05	1,47E+06	0						2	F2:1846	150	155	W5:Dihydroxy:16.90
3	4	P02751	R.TEIDKPSQMQ(+.98)VTDVQDNSISVK.W	Y	41,71	2462,1846	22	4,9	821,7395	3	31		0							14	F14:2675	1540	1561	Q10:Deamidation (NQ):9.40
3	4	P02751	R.TYLGNALVC(+31.99)T(-2.02)CYGGSR.G	Y	41,23	1706,7393	16	-8,2	854,3699	2	33,8		0	1,77E+06						13	F13:2909	68	83	C9:Dihydroxy:0.00;T10:2-amino-3-oxo-butanoic_acid:3.92
3	4	P02751	Q.STVSDVPR.D	Y	41,19	859,4399	8	8,1	430,7307	2	24,7			0						23	F23:2082	1445	1452	
3	4	P02751	K.HYQINQQWE.R	Y	40,98	1244,5574	9	1,8	623,2871	2	31,4	1,49E+06	1,67E+06							10	F10:2589	58	66	
3	4	P02751	K.FGFC(+15.99)PMAAHEEICTTNEGVMYR.I	Y	40,69	2521,053	22	8	841,3651	3	35,	1,40E+06	1,26E+06							18	F18:211	458	479	C4:Oxidation or

						5					5						9				Hydroxylat ion (C):11.94
3	4	P027 51	K.TGPMK.E	Y	40, 45	532, 2679	5	- 5, 1	533, 2725	1	1 1, 6			0		23	F23: 843	1762	176 6		
3	4	P027 51	R.HTSVQTTS.S	Y	40, 33	859, 4036	8	2, 1	860, 4127	1	1 1, 9	7,95E+05	4,03E+05	1,72E+ 05		13	F13: 843	273	280		
3	4	P027 51	R.ISC(+31.99)T(-2.02)IANR.C	Y	39, 36	906, 4229	8	6, 6	454, 2217	2	2 5, 4	3,89E+05				2	F2:2 050	133	140		C3:Dihydro xy:40.91;T 4:2-amino- 3-oxo- butanoic_ acid:26.31
3	4	P027 51	R.TYLGNALVCT(- 2.02)C(+31.99)YGGSR.G	Y	38, 84	1706 ,739 3	16	- 4, 3	854, 3732	2	3 2, 9	3,67E+05			0	3	F3:2 697	68	83		T10:2- amino-3- oxo- butanoic_ acid:0.00;C 11:Dihydro xy:0.00
3	4	P027 51	R.GNLLQC(+15.99)ICT(-2.02)GNGR.G	Y	38, 35	1361 ,618	13	8	681, 8217	2	3 0, 6	7,84E+05				3	F3:2 497	253	265	2-amino- 3-oxo- butanoic_ acid	C6:Oxidati on or Hydroxylat ion (C):0.00;T9 :2-amino- 3-oxo- butanoic_ acid:1000. 00
3	4	P027 51	K.FGFC(+31.99)PMAAHEEIC(- 33.99)TTNEGVMYR.I	Y	37, 92	2503 ,060 5	22	3, 2	835, 3635	3	3 4, 5		0			13	F13: 297 0	458	479		C4:Dihydro xy:0.00;C1 3:Dehydro alanine (C):0.00
3	4	P027 51	R.GN(+.98)LLQC(- 33.99)IC(+31.99)TGNGR.G	Y	37, 55	1346 ,624 9	13	- 0, 2	674, 3196	2	3 1, 8	0				6	F6:2 799	253	265		N2:Deami dation (NQ):47.09 ;C6:Dehydr oalanine (C):14.04;C 8:Dihydrox y:14.04
3	4	P027 51	K.C(+27.99)DP(- 30.01)HEATCYDDGK.T	Y	37, 48	1450 ,512 9	13	6, 8	484, 5149	3	2 4, 4	5,74E+05				3	F3:1 966	2288	230 0	Proline oxidation to pyrrolidin one	C1:Formyl ation:54.4 0;P3:Prolin e oxidation to pyrrolidino ne:1000.00
3	4	P027 51	R.CT(-2.02)CVGNGR.G	Y	37, 24	806, 3163	8	1, 6	807, 3249	1	1 3, 9	9,42E+05	0	0		5	F5:1 087	496	503	2-amino- 3-oxo- butanoic_ acid	T2:2- amino-3- oxo- butanoic_ acid:1000. 00
3	4	P027 51	K.HYQIN.Q	Y	37, 19	673, 3184	5	1, 1	674, 3264	1	1 7, 3	1,28E+06	1,19E+06	1,59E+ 05		14	F14: 137 4	58	62		
3	4	P027 51	K.GLK(+27.99)PGVVYE.G	Y	37, 15	988, 5229	9	14	495, 2754	2	3 0, 8			1,25E+ 06		19	F19: 263 0	670	678		K3:Formyl ation:8.22
3	4	P027 51	R.VGDTYERPK(+31.99).D	Y	36, 62	1095 ,519 7	9	- 4, 3	366, 1789	3	1 7, 3		1,49E+05			15	F15: 138 0	108	116		K9:Dihydr oxy:0.00
3	4	P027 51	K.IGDTW(+13.98)R.R	Y	36, 13	760, 3504	6	5, 3	381, 1845	2	2 7, 3	6,10E+05	1,01E+06	0		3	F3:2 217	150	155	Tryptopha n oxidation to oxolacton e	W5:Trypto phan oxidation to oxolactone :1000.00
3	4	P027	R.WTPPR.A	Y	35,	655,	5	-	328,	2	2	4,12E+05	0	4,44E+		6	F6:2	1017	102		

		51			91	3442		2,7	6785		6,4			05								287		1		
3	4	P02751	R.GFNC(+31.99)ESKPEAEETC(-33.99)FDK.Y	Y	35,83	1930,7891	17	1,1	966,4029	2	29	3,15E+06	1,74E+06		4,88E+07		2,33E+06	1,77E+07	1,37E+07	1,82E+06	12	F12:2346	84	100		C4:Dihydroxy:17.01;C14:Dehydroalanine (C):34.74
3	4	P02751	R.VGDTYER(+31.99)PK.D	Y	35,75	1095,5197	9	-0,5	366,1803	3	18,2		9,96E+04								13	F13:1439	108	116		R7:Dihydroxy:0.00
3	4	P02751	K.CDPHEAT(-2.02)CYDDGK.T	Y	35,24	1450,5129	13	6,6	484,5148	3	24,5	1,59E+06				3,10E+05	5,11E+04				2	F2:1966	2288	2300		T7:2-amino-3-oxo-butanoic_acid:32.28
3	4	P02751	R.RPHETGGY(-2.02)MLEC(+15.99)VCLGNGK.G	Y	34,9	2076,918	19	7,7	1039,4742	2	33,3						6,95E+05				54	F54:1953	156	174		Y8:2-amino-3-oxo-butanoic_acid:11.12;C12:Oxidation or Hydroxylation (C):0.00
3	4	P02751	K.FGFCPM(+15.99)AAHEEICTTNEGVMYR.I	Y	34,83	2521,0535	22	9	841,366	3	35,5	1,06E+06	2,43E+06								17	F17:2095	458	479		M6:Oxidation (M):44.11
3	4	P02751	R.HTSVQ(+.98)TTS.S	Y	34,64	860,3876	8	-1,9	861,3932	1	12,5	1,86E+05									4	F4:934	273	280	Deamidation (NQ)	Q5:Deamidation (NQ):1000.00
3	4	P02751	K.YTGNTY.R	Y	34,22	717,297	6	-2,2	718,3027	1	22,5	0	0								5	F5:1892	101	106		
3	4	P02751	K.DTLTSR.P	Y	34,19	691,35	6	-0,6	692,3569	1	13,8	3,57E+05	0	1,28E+05							24	F24:1053	1797	1802		
3	4	P02751	N.ALVCT(-2.02)CYGGSR.G	Y	34,18	1126,49	11	6,9	564,2562	2	28,9	3,20E+06	1,43E+06								2	F2:2354	73	83		T5:2-amino-3-oxo-butanoic_acid:11.10
3	4	P02751	R.FLATTPN.S	Y	34,16	762,3912	7	-2,2	763,3968	1	29,1	0	0	0							23	F23:2522	1911	1917		
3	4	P02751	R.SYTITGLQPGTDYK(-1.03).I	Y	33,92	1541,725	14	-13	771,8597	2	33,7			4,94E+06							19	F19:2913	1867	1880	Lysine oxidation to aminoadipic semialdehyde	K14:Lysine oxidation to aminoadipic semialdehyde:1000.00
3	4	P02751	R.QGENGQM(+15.99)M(+15.99)SCT(-2.02)CLGNGK.G	Y	33,23	1786,6743	17	9	894,3525	2	29					2,30E+05					39	F39:1621	2267	2283	Oxidation (M)	M7:Oxidation (M):1000.00;M8:Oxidation (M):1000.00;T11:2-amino-3-oxo-butanoic_acid:12.81
3	4	P02751	R.GFNC(-33.99)ESKPEAEETC(+31.99)FDK.Y	Y	33,14	1930,7891	17	2,5	966,4042	2	32,1					0	1,05E+06		2,72E+07		51	F51:1825	84	100		C4:Dehydroalanine (C):0.00;C14:Dihydroxy:0.00
3	4	P02751	S.TTSNYEQ(+.98)DQKYSFC(-33.99)TDHTVLVQTR.G	Y	33,03	2830,3047	24	-2,8	944,4396	3	33,7									0	74	F74:1986	388	411	Dehydroalanine (C)	Q7:Deamidation (NQ):0.00;C14:Dehydroalanine

																									(C):1000.00
3	4	P02751	R.VDVIPVNLPGEHGQR.L	Y	32,75	1628,8634	15	6,9	543,9655	3	31,9	3,00E+05								2	F2:2602	939	953		
3	4	P02751	R.TYLGNAL.V	Y	32,66	750,3912	7	8,7	751,405	1	30,9	0		8,64E+05						20	F20:2550	68	74		
3	4	P02751	D.TYERPK.D	Y	32,65	792,413	6	0,3	793,4205	1	20,2	1,22E+05								4	F4:1677	111	116		
3	4	P02751	K.HYQINQ(+.98).Q	Y	31,86	802,361	6	-0,3	803,368	1	23,7			0						24	F24:1968	58	63		Q6:Deamidation (NQ):17.01
3	4	P02751	K.FGFCP(+13.98)MAAHEEICTTNEGVM(+15.99)YR.I	Y	31,35	2535,0327	22	6,4	846,0236	3	35,3				5,39E+06	0		3,09E+06	0	29	F29:2136	458	479	Proline oxidation to pyroglutamic acid	P5:Proline oxidation to pyroglutamic acid:1000.00;M20:Oxidation (M):51.01
3	4	P02751	R.QGENGQMM(+31.99)SCT(-2.02)CLGNGK.G	Y	31,02	1786,6743	17	9	894,3525	2	29,2					1,30E+05				42	F42:1609	2267	2283		M8:Dioxidation (M):0.00;T11:2-amino-3-oxo-butanoic_acid:14.04
3	4	P02751	R.IGDT(-2.02)WSK.K	Y	30,65	803,3813	7	5,3	402,7001	2	26,2		5,30E+05							11	F11:2105	242	248		T4:2-amino-3-oxo-butanoic_acid:32.28
3	4	P02751	K.HYQINQ.Q	Y	30,63	801,377	6	-1,1	802,3834	1	21,3	0		8,48E+04						4	F4:1780	58	63		
3	4	P02751	R.TIKP(+31.99)DVR.S	Y	30,35	859,4763	7	-3,2	430,744	2	18,8	0								6	F6:1563	1860	1866		P4:Dihydroxy:0.00
3	4	P02751	R.VEYELSEEGDEPQYLDLPSTATSVNIPDLLPGRK(-1.03).Y	Y	29,86	3772,8206	34	7,4	1258,6234	3	50,4					2,75E+04				38	F38:3187	752	785	Lysine oxidation to aminoadipic semialdehyde	K34:Lysine oxidation to aminoadipic semialdehyde:1000.00
3	4	P02751	R.ISCTIANRC(+15.99)HEGGQ(+.98)SYK.I	Y	29,67	1882,8302	17	-0,6	628,6169	3	24,6			0						23	F23:2072	133	149		C9:Oxidation or Hydroxylation (C):0.00;Q14:Deamidation (NQ):34.75
3	4	P02751	T.NFLVR.Y	N	29,61	647,3755	5	2	648,384	1	30,7			7,54E+06						19	F19:2617	1297	1301		
3	4	P02751	G.DTYERPK.D	Y	29,44	907,4399	7	-1,8	454,7264	2	26,1				1,69E+04					31	F31:1425	110	116		
3	4	P02751	S.TTSN(+.98)YEQDQKYSFC(-33.99)TDHTVLVQTR.G	Y	29,08	2830,3047	24	-4,2	944,4382	3	33,9					2,64E+06	1,60E+06			51	F51:1969	388	411	Dehydroalanine (C)	N4:Deamidation (NQ):0.00;C14:Dehydroalanine (C):1000.00
3	4	P02751	R.QGENGQM(+15.99)MSC(+47.98)TC(-33.99)LGNGK.G	Y	28,74	1786,692	17	1,6	894,3547	2	29,2								1,79E+05	71	F71:1618	2267	2283		M7:Oxidation (M):17.01;

																										C10:Cysteine oxidation to cysteic acid:0.00;C12:Dehydroalanine (C):0.00
3	4	P02751	L.TR(+31.99)GATY(+44.99)NVIVEALKDQQR.H	Y	28,73	2138,0715	18	1,3	713,6987	3	34,3			2,69E+06							22	F22:2979	2163	2180	Oxidation to nitro	R2:Dihydroxy:54.32;Y6:Oxidation to nitro:1000.00
3	4	P02751	K.FGFCPMAAHEEIC(+15.99)TTNEGVMYR.I	Y	28,4	2521,0535	22	14	841,3702	3	35,1	0									9	F9:2103	458	479		C13:Oxidation or Hydroxylation (C):0.00
3	4	P02751	R.QGENGQM(+31.99)MSCT(-2.02)CLGNGK.G	Y	28,28	1786,6743	17	9,2	894,3527	2	29,1								1,75E+05		65	F65:1621	2267	2283		M7:Dioxidation (M):0.00;T11:2-amino-3-oxo-butanoic_acid:14.04
3	4	P02751	R.QGEN(+.98)GQMMS(-2.02)CTC(+31.99)LGNGK.G	Y	27,87	1787,6583	17	10	894,8458	2	29,2					1,25E+05					39	F39:1641	2267	2283		N4:Deamidation (NQ):18.00;S9:2-amino-3-oxo-butanoic_acid:12.81;C12:Dihydroxy:12.81
3	4	P02751	K.FGFCP(+13.98)MAAHEEICTTNEGVMYR(+15.99).I	Y	27,78	2535,0327	22	10	1268,5365	2	35,5					0					39	F39:2162	458	479	Proline oxidation to pyroglutamic acid	P5:Proline oxidation to pyroglutamic acid:1000.00;R22:Oxidation or Hydroxylation:0.00
3	4	P02751	K.PYQGWMM(+31.99)VDCT(-2.02)CLGEGSGR.I	Y	27,66	2118,8267	19	9	1060,4302	2	35,4								0		64	F64:2145	204	222		M7:Dioxidation (M):0.00;T11:2-amino-3-oxo-butanoic_acid:19.69
3	4	P02751	R.GNLLQC(+15.99)IC(-33.99)TGN(+15.99)GR.G	Y	27,58	1345,6409	13	-6,3	673,8235	2	34,6									6,82E+05	80	F80:2064	253	265		C6:Oxidation or Hydroxylation (C):13.67;C8:Dehydroalanine (C):13.67;N11:Oxidation or Hydroxylation:5.03
3	4	P02751	K.HYQINQQW(+13.98).E	Y	27,43	1129,4941	8	7,3	565,7585	2	28,6		5,74E+05								11	F11:2307	58	65	Tryptophan oxidation to oxolactone	W8:Tryptophan oxidation to oxolactone:1000.00
3	4	P02751	R.GFNCES(-2.02)KPEAEETCFDK.Y	Y	27,36	1930,771	17	4,9	966,3976	2	31,	5,05E+07									8	F8:1784	84	100		S6:2-amino-3-

						2					7															oxo-butanoic_acid:8.94
3	4	P02751	S.TTSNYEQDQ(+.98)KYSFC(-33.99)TDHTVLVQTR.G	Y	27,19	2830,3047	24	-5,2	944,4373	3	33,8					1,41E+06	0				44	F44:1992	388	411	Dehydroalanine (C)	Q9:Deamidation (NQ):0.00; C14:Dehydroalanine (C):1000.00
3	4	P02751	R.RPHETGGY(+15.99)MLECVCLGNGK(-2.02).G	Y	26,84	2076,918	19	8,6	1039,4752	2	33,2						1,26E+06				47	F47:1933	156	174		Y8:Oxidation or Hydroxylation:11.90; K19:2-amino-3-oxo-butanoic_acid:44.80
3	4	P02751	R.GNLLQCICT(-2.02)GN(+.98)GR.G	Y	26,37	1346,6071	13	6,1	674,3149	2	34,9				2,84E+05						31	F31:2097	253	265	2-amino-3-oxo-butanoic_acid	T9:2-amino-3-oxo-butanoic_acid:1000.00;N11:Deamidation (NQ):26.50
3	4	P02751	K.FGFCP(+13.98)MAAHEEICTTNEGVMY(+15.99)R.I	Y	26,27	2535,0327	22	15	1268,5424	2	35,3							0			56	F56:2144	458	479	Proline oxidation to pyroglutamic acid	P5:Proline oxidation to pyroglutamic acid:1000.00;Y21:Oxidation or Hydroxylation:0.00
3	4	P02751	K.DSM(+15.99)IWDC(+31.99)T(-2.02)CIGAGR.G	Y	25,98	1572,6007	14	-2,7	787,3055	2	33,5		2,09E+05								11	F11:2725	117	130	Oxidation (M)	M3:Oxidation (M):1000.00;C7:Dihydroxy:0.00; T8:2-amino-3-oxo-butanoic_acid:111.20
3	4	P02751	K.C(+15.99)DP(+15.99)HEATC(-33.99)YDDGK.T	Y	25,97	1450,5306	13	-2,5	726,2708	2	28,1			1,75E+05							26	F26:1555	2288	2300		C1:Oxidation or Hydroxylation (C):13.94;P3:Oxidation or Hydroxylation:27.33; C8:Dehydroalanine (C):13.94
3	4	P02751	R.F(+31.99)LATTP(-30.01)NSLLVSWQPPR.A	Y	25,78	1928,0155	17	10	965,0248	2	36,5		0								15	F15:3204	1911	1927		F1:Dihydroxy:40.43;P6:Proline oxidation to pyrrolidinone:77.76
3	4	P02751	R.TEIDKP(+15.99)SQMQVTDVQDNSISVK.W	Y	25,39	2477,1956	22	9,8	826,7472	3	32,4									0	73	F73:1870	1540	1561		P6:Oxidation or Hydroxylation:0.00
3	4	P02751	I.GDTWSK.K	Y	25,38	692,3129	6	2,8	693,3221	1	26,6		0								15	F15:2283	243	248		
73	35	P027	E.AALVAIDYINQNLPWGYK.H	Y	83,	2048	18	4,	1025	2	4	5,96E+05									1	F1:3	40	57		

		65			47	,073		3	,0482		0,1											366				
73	35	P02765	R.AHYDLR.H	Y	41,51	773,382	6	-1,6	387,6977	2	18,6			2,73E+05							22	F22:1482	312	317		
73	35	P02765	K.HTLNQIDEVK.V	Y	30,31	1195,6196	10	6,3	598,8209	2	30,1						0			3,22E+04	80	F80:1695	58	67		
22	14	P02768	K.VFDEFKPLVEEPQNLIK.Q	Y	97,82	2044,088	17	3,8	1023,0552	2	37,9	2,19E+07	6,94E+06	2,05E+06							1	F1:3186	397	413		
22	14	P02768	K.KVPQVSTPTLVEVSR.N	Y	83	1638,9304	15	7,7	820,4788	2	33,8			6,86E+05			2,21E+07		0	6,71E+06	54	F54:1995	438	452		
22	14	P02768	K.VPQVSTPTLVEVSR.N	Y	75,75	1510,8354	14	5,6	756,4292	2	34,9			9,66E+05			6,53E+05			1,97E+06	47	F47:2079	439	452		
22	14	P02768	R.RHPDYSVVLLLR.L	Y	65,9	1466,8357	12	3,2	489,9541	3	36,2	3,12E+06	1,25E+06								1	F1:3041	361	372		
22	14	P02768	K.LDELRDEGK.A	Y	61,33	1073,5353	9	0,7	537,7753	2	24			1,74E+05		4,19E+04			0		23	F23:2011	206	214		
22	14	P02768	R.HPYFYAPELLFFAK.R	Y	59,65	1741,8867	14	4,7	871,9547	2	40,7					0			1,52E+05		40	F40:2567	170	183		
22	14	P02768	R.HPDYSVVLLLR.L	Y	51,73	1310,7346	11	3,1	656,3766	2	37	5,99E+05									1	F1:3113	362	372		
22	14	P02768	L.GEYK(+42.01)FQNALLVR.Y	Y	50,92	1478,7881	12	6,3	740,406	2	37,3			2,23E+06			9,51E+06			2,83E+06	54	F54:2286	423	434	Acetylation (K)	K4:Acetylation (K):1000.00
22	14	P02768	K.FQNALLVR.Y	Y	50,49	959,5552	8	2,5	480,7861	2	33,2	1,19E+06	1,27E+06								1	F1:2762	427	434		
22	14	P02768	E.FKPLVEEPQNLIK.Q	Y	42,41	1553,8817	13	3,1	777,9506	2	34,7	5,68E+06	4,38E+06								1	F1:2907	401	413		
22	14	P02768	K.KVPQVSTPTL.V	Y	37,82	1068,6179	10	6,1	535,3195	2	29,8			7,00E+05							20	F20:2462	438	447		
22	14	P02768	K.KVPQVSTPTLVE.V	Y	37,33	1296,7289	12	3,4	649,3739	2	32,7			2,41E+06							19	F19:2810	438	449		
22	14	P02768	K.TC(-33.99)VADESAENC(+31.99)DK.S	Y	29,96	1381,5304	13	-2,5	691,7708	2	24,3	2,05E+05									6	F6:2074	76	88		C2:Dehydroalanine (C):32.47;C11:Dihydroxy:39.76
22	14	P02768	K.VFDEFKPLVEEPQN(+.98)LIK(-1.03).Q	Y	28,59	2044,0404	17	-10	1023,0168	2	40,6	1,94E+05									1	F1:3409	397	413		N14:Deamidation (NQ):10.11;K17:Lysine oxidation to aminoadipic semialdehyde:52.48
22	14	P02768	K.AVMDDFAAFVEK.C	Y	28,34	1341,6274	12	4,2	671,8238	2	37,9	8,44E+05									1	F1:3190	570	581		
57	30	P04003	K.LNNGEITQHR.K	Y	64,34	1180,5948	10	-1,7	591,3037	2	21,4	4,58E+04									5	F5:1797	370	379		
57	30	P04003	K.EDVYVVGTVLR.Y	Y	62,23	1248,6714	11	4,3	625,3456	2	32,6	2,88E+05	0								3	F3:2672	319	329		
57	30	P04003	R.HPGELR.N	Y	44,18	707,3715	6	-0,8	354,6927	2	13,4	0									4	F4:1020	115	120		
57	30	P040	R.NGQVEIK.T	Y	36,	786,	7	-	787,	1	1	9,25E+04									4	F4:1	121	127		

		03			95	4235		1,4	4297		9,2											587				
2	3	P05160	C.GFPHVENGR.I	Y	57,15	1011,4886	9	7,2	506,7552	2	26,8	1,56E+06	7,53E+05								12	F12:2154	26	34		
2	3	P05160	F.PHVENGR.I	Y	45,53	807,3987	7	5,1	404,7087	2	32,4	1,73E+05	2,82E+05	5,62E+05	4,96E+04	3,23E+06	7,01E+06	0,00E+00	4,69E+06	1,82E+06	51	F51:1846	28	34		
2	3	P05160	R.IAQYYYTFK.S	Y	77,69	1195,5913	9	3,2	598,8049	2	32,8	3,03E+08	2,62E+08	1,35E+08	3,20E+08	4,17E+07	4,48E+07	2,93E+08	1,36E+07	1,71E+08	19	F19:2828	35	43		
2	3	P05160	R.IAQ(+.98)YYYTFK.S	Y	74,45	1196,5753	9	4,6	599,2977	2	34,6	2,33E+08	1,65E+08	1,41E+08	3,66E+07	1,63E+07		4,80E+07	1,33E+07		62	F62:2030	35	43	Deamidation (NQ)	Q3:Deamidation (NQ):1000.00
2	3	P05160	R.IAQY(+33.96)YYTFK.S	Y	61,21	1229,5524	9	6	615,7872	2	35,8						3,22E+06	6,02E+05		1,68E+07	78	F78:2091	35	43		Y4:Chlorination of tyrosine residues:21.94
2	3	P05160	R.IAQYYYT.F	Y	41,45	920,428	7	1,5	921,4366	1	31,6	9,68E+06	4,30E+06	1,47E+07							10	F10:2611	35	41		
2	3	P05160	R.IAQYY.Y	Y	35,72	656,317	5	-0,8	657,3237	1	28,5	3,67E+06		3,44E+06							23	F23:2456	35	39		
2	3	P05160	R.IAQYYY(+33.96)TFK.S	Y	35,57	1229,5524	9	5	615,7866	2	35,2				1,20E+06						32	F32:2106	35	43		Y6:Chlorination of tyrosine residues:0.00
2	3	P05160	R.IAQYYY.T	Y	35,55	819,3802	6	2,7	820,3897	1	32,2		4,50E+06								10	F10:2666	35	40		
2	3	P05160	I.AQYYYTFK.S	Y	47,95	1082,5073	8	6,7	542,2646	2	34,1	1,26E+06			7,93E+06			4,86E+06		2,27E+06	59	F59:2018	36	43		
2	3	P05160	A.QYYYTFK.S	Y	46,05	1011,4702	7	7,3	506,7461	2	34,1	8,20E+06			2,88E+07	6,55E+06	1,52E+06	1,72E+07	2,84E+06	7,20E+06	59	F59:2016	37	43		
2	3	P05160	Y.YYTFK.S	Y	39,93	720,3483	5	-1,1	361,181	2	28	0,00E+00	8,58E+05	0,00E+00							14	F14:2395	39	43		
2	3	P05160	K.SFYFPMSIDKK.L	Y	84,02	1361,6689	11	1,5	681,8428	2	35	1,76E+08	1,62E+08	2,06E+08	1,61E+08	1,05E+08	3,71E+07	1,45E+08	1,13E+08	3,69E+07	19	F19:3042	44	54		
2	3	P05160	K.SFYFPM(+15.99)SIDKK.L	Y	77,12	1377,6638	11	2,6	689,8409	2	33,1		2,15E+06	9,45E+07	2,31E+07	2,56E+07	2,00E+07	2,07E+07	7,46E+06	1,58E+07	19	F19:2853	44	54	Oxidation (M)	M6:Oxidation (M):1000.00
2	3	P05160	K.SFYFPMSIDK.K	Y	76,45	1233,574	10	2	617,7955	2	36,6	1,82E+08	1,92E+08	1,53E+08	2,90E+08	1,47E+08	9,15E+07	2,60E+08	1,18E+08	1,16E+08	19	F19:3180	44	53		
2	3	P05160	K.SFYFPM(+31.99)SIDKK.L	Y	73,31	1393,6587	11	7,3	697,8417	2	30,8			6,32E+06							21	F21:2552	44	54	Dioxidation (M)	M6:Dioxidation (M):1000.00
2	3	P05160	K.SFYFPM(+15.99)SIDK.K	Y	71,68	1249,5688	10	2,8	625,7935	2	35,7	7,17E+06	1,89E+07	4,84E+07	1,01E+08	4,23E+07	1,27E+08	1,06E+08	1,75E+07	1,75E+08	58	F58:2143	44	53	Oxidation (M)	M6:Oxidation (M):1000.00
2	3	P05160	K.SFYFP(+15.99)MSIDK.K	Y	66,94	1249,5688	10	3,7	625,794	2	35,8					1,78E+07	8,07E+05	3,68E+07	3,16E+07	1,61E+05	67	F67:2152	44	53		P5:Oxidation or Hydroxylation:46.20
2	3	P05160	K.SFYFP(+15.99)MSIDKK.L	Y	66,65	1377,6638	11	7,9	689,8446	2	33,9				1,19E+07		5,50E+06	3,61E+06	1,14E+07		63	F63:2029	44	54		P5:Oxidation or Hydroxylation:31.20
2	3	P05160	K.SFY(+67.92)FPM(+15.99)SIDK.K	Y	60,04	1317,491	10	6,1	659,7568	2	37,5									4,35E+05	77	F77:2315	44	53	Dichlorination of tyrosine residues; Oxidation	Y3:Dichlorination of tyrosine residues:1000.00;M6

																									(M)	:Oxidation (M):1000.00
2	3	P05160	K.SFY(+33.96)FPM(+15.99)SIDKK.L	Y	52,72	1411,6249	11	5,3	706,8234	2	35,3						3,99E+05				50	F50:2090	44	54	Chlorination of tyrosine residues; Oxidation (M)	Y3:Chlorination of tyrosine residues:1000.00;M6:Oxidation (M):1000.00
2	3	P05160	K.SFY(+33.96)FPM(+15.99)SIDK.K	Y	48,82	1283,5299	10	5,7	642,7759	2	36,7									8,33E+06	80	F80:2244	44	53	Chlorination of tyrosine residues; Oxidation (M)	Y3:Chlorination of tyrosine residues:1000.00;M6:Oxidation (M):1000.00
2	3	P05160	K.SFY(+33.96)FP(+15.99)MSIDK.K	Y	48,74	1283,5299	10	6,1	642,7761	2	37									3,75E+06	78	F78:2188	44	53	Chlorination of tyrosine residues	Y3:Chlorination of tyrosine residues:1000.00;P5:Oxidation or Hydroxylation:20.54
2	3	P05160	K.SFY(+67.92)FPMSIDK.K	Y	48,56	1301,496	10	6,3	651,7593	2	39,7									1,66E+05	78	F78:2401	44	53	Dichlorination of tyrosine residues	Y3:Dichlorination of tyrosine residues:1000.00
2	3	P05160	K.SFYFP(+31.99)MSIDK.K	Y	44,28	1265,5638	10	6,8	633,7935	2	32,6		4,03E+05	2,83E+06							20	F20:2700	44	53		P5:Dihydroxy:4.75
2	3	P05160	K.SFY(+33.96)FPMSIDK.K	Y	43,9	1267,535	10	5,3	634,7781	2	38,7						0,00E+00			1,64E+06	77	F77:2416	44	53	Chlorination of tyrosine residues	Y3:Chlorination of tyrosine residues:1000.00
2	3	P05160	K.SFYFPMSIDK(+15.99).K	Y	42,85	1249,5688	10	5	625,7948	2	55,3					1,05E+04				0,00E+00	40	F40:3497	44	53		K10:Oxidation or Hydroxylation:0.00
2	3	P05160	K.SFYFPMSID.K	Y	42,26	1105,479	9	-2,7	1106,4833	1	38,6	2,72E+06		1,99E+06							5	F5:3403	44	52		
2	3	P05160	K.SFYFPM.S	Y	39,85	790,336	6	0,4	791,3436	1	36,9	2,29E+07	9,08E+06	1,19E+07							6	F6:3251	44	49		
2	3	P05160	K.SFYFP.M	Y	37,01	659,2955	5	-1	660,3021	1	34,8			2,70E+06							24	F24:3037	44	48		
2	3	P05160	K.SFYFP(+31.99)M(-48.00)SID(+15.99)K.K	Y	36,98	1233,5553	10	6,5	617,7889	2	56,7					0,00E+00					44	F44:3597	44	53	Dethiomethyl	P5:Dihydroxy:0.00;M6:Dethiomethyl:1000.00;D9:Oxidation or Hydroxylation:0.00
2	3	P05160	K.SFYFPM(+31.99)SIDK.K	Y	35,04	1265,5638	10	7,3	633,7938	2	32,8		4,96E+05								11	F11:2670	44	53	Dioxidation (M)	M6:Dioxidation (M):1000.00
2	3	P05160	S.FYFPMSIDK.K	Y	39,98	1146,542	9	5,8	574,2816	2	37,9					1,20E+06		1,96E+06		3,97E+05	43	F43:2337	45	53		
2	3	P05160	S.FYFPM(+15.99)SIDK.K	Y	38,5	1162,5369	9	6,5	582,2795	2	35,8						1,15E+06				51	F51:2133	45	53	Oxidation (M)	M5:Oxidation (M):1000.00

2	3	P05160	F.YFPMSIDKK.L	Y	50,21	1127,5685	9	-0,8	564,7911	2	29,5			1,76E+06							24	F24:2536	46	54		
2	3	P05160	F.YFPM(+15.99)SIDK.K	Y	45,26	1015,4684	8	5,9	508,7445	2	35,7				1,21E+06		6,25E+06	2,21E+06		9,83E+06	77	F77:2164	46	53	Oxidation (M)	M4:Oxidation (M):1000.00
2	3	P05160	F.YFPMSIDK.K	Y	43,7	999,4735	8	5,1	500,7466	2	38,1				5,12E+06			1,35E+06		5,94E+05	78	F78:2280	46	53		
2	3	P05160	Y.FPMSIDKK.L	Y	50,52	964,5051	8	2,3	483,2609	2	30,5		1,40E+06								10	F10:2500	47	54		
2	3	P05160	Y.FPMSIDKK.K	Y	48,38	836,4102	7	2,2	419,2133	2	32,3	9,67E+05	3,24E+06								1	F1:2685	47	53		
2	3	P05160	P.MSIDKK.L	Y	36,53	720,384	6	0,5	361,1994	2	13,4	9,15E+05	0,00E+00	0,00E+00							5	F5:1032	49	54		
2	3	P05160	S.IDKKLSFFC(+31.99)LAGYTTESGR.Q	Y	36,1	2167,062	19	-1,2	1084,537	2	36,7			8,25E+05							26	F26:2237	51	69		C9:Dihydroxy:12.28
2	3	P05160	K.K(+42.01)LS(+79.97)FFC(+15.99)LAGYTTESGR.Q	Y	49,18	1916,8379	16	4,4	959,4304	2	39,2						0,00E+00				51	F51:2410	54	69	Acetylation (K); Oxidation or Hydroxylation (C)	K1:Acetylation (K):1000.00;S3:Phosphorylation (STY):43.57;C6:Oxidation or Hydroxylation (C):1000.00
2	3	P05160	K.LSFFCLAGYTTESGR.Q	Y	75,48	1650,7711	15	7,6	826,3991	2	41,6									2,28E+05	78	F78:2539	55	69		
2	3	P05160	K.LS(-2.02)FFCLAGYTTESGR.Q	Y	63,5	1648,7555	15	7,5	825,3912	2	47,1	3,57E+05	2,29E+04		9,31E+05	5,94E+05	2,96E+05	9,29E+05	6,36E+05	6,31E+05	74	F74:3014	55	69		S2:2-amino-3-oxo-butanoic_acid:34.57
2	3	P05160	K.LSFFCLAGY(+15.99)TTESGRQEEQTTCTTEGWSPEPR.C	Y	51,33	3626,593	32	5,9	907,6609	4	37,4	5,49E+07	3,43E+07		3,43E+07		6,17E+06	8,36E+07	1,38E+07		54	F54:2303	55	86		Y9:Oxidation or Hydroxylation:5.14
2	3	P05160	K.LSFF(+31.99)C(-33.99)LAGYTTESGR.Q	Y	48,42	1648,7733	15	-5,5	825,3894	2	47						3,27E+04		8,43E+04	7,01E+04	68	F68:2969	55	69	Dehydroalanine (C)	F4:Dihydroxy:12.60;C5:Dehydroalanine (C):1000.00
2	3	P05160	K.LSFFCLAGYTTESGR(+15.99)QEEQTTCTTEGWSPEPR.C	Y	46,01	3626,593	32	7,4	907,6622	4	37,4		6,36E+07	2,11E+07	4,37E+07	1,04E+08		3,38E+07	2,13E+07		40	F40:2295	55	86		R15:Oxidation or Hydroxylation:2.81
2	3	P05160	K.LS(-2.02)FFC(+15.99)LAGYTTESGR.Q	Y	45,22	1664,7504	15	2,6	833,3846	2	37,5	1,73E+05	1,46E+05								11	F11:3035	55	69	Oxidation or Hydroxylation (C)	S2:2-amino-3-oxo-butanoic_acid:0.00;C5:Oxidation or Hydroxylation (C):1000.00
2	3	P05160	K.LSFFCLAGYTTESGRQEEQTTC(+15.99)TTEGWSPEPR.C	Y	45,13	3626,593	32	1	907,6564	4	35,1	5,96E+07	2,39E+07	7,35E+07	4,36E+07	6,46E+07	7,12E+06	4,97E+07		2,72E+06	4	F4:3094	55	86		C22:Oxidation or Hydroxylation (C):5.38
2	3	P05160	K.LSFFC(+15.99)LAGYTTESGRQEEQTTCTTEGWSPEPR.C	Y	43,63	3626,593	32	6,4	907,6614	4	37,			2,49E+07	3,60E+07	3,27E+07	3,09E+06		2,86E+07		64	F64:230	55	86		C5:Oxidation or

											3											3				Hydroxylation (C):5.38
2	3	P05160	K.LSFFCLAGY(-2.02)TTESGR.Q	Y	40,81	1648,7555	15	4,7	825,3889	2	47,1					0,00E+00			9,46E+04		40	F40:2999	55	69		Y9:2-amino-3-oxo-butanoic_acid:7.43
2	3	P05160	K.LSFFCLAGYTTESGRQEEQTTCTTEGWSPEPR.C	Y	40,14	3610,5979	32	-0,7	1204,5391	3	41,2	8,47E+05	4,66E+05	0,00E+00							14	F14:3582	55	86		
2	3	P05160	C.LAGYTTESGR.Q	Y	62,43	1053,509	10	1,3	527,7625	2	25,3	3,17E+06	7,42E+06	5,36E+06							24	F24:2120	60	69		
2	3	P05160	L.AGYTTESGR.Q	Y	51,36	940,425	9	-1	471,2193	2	14	1,50E+06	1,43E+06	1,35E+05							23	F23:1075	61	69		
2	3	P05160	A.GYTTESGR.Q	Y	49,45	869,3879	8	-0,1	435,7012	2	13,3	8,42E+05	1,85E+05	9,36E+05							14	F14:993	62	69		
2	3	P05160	R.QEEQTTC(-33.99)TTEGW(+31.99)SPEPR.C	Y	66,66	1975,8395	17	2,9	988,9299	2	31,5				3,27E+05		3,78E+05	1,50E+06	2,14E+05	3,89E+05	72	F72:1792	70	86	Dehydroalanine (C)	C7:Dehydroalanine (C):1000.00;W12:Dihydroxy:60.92
2	3	P05160	R.QEEQTTCTT(-2.02)EGWSPEPR.C	Y	52,27	1975,8218	17	9,9	988,928	2	31,5				5,94E+05	4,90E+05					40	F40:1793	70	86		T9:2-amino-3-oxo-butanoic_acid:0.00
2	3	P05160	R.QEEQTTCT(-2.02)TEGWSPEPR.C	Y	46,48	1975,8218	17	11	988,9288	2	31,4					4,09E+05					41	F41:1780	70	86		T8:2-amino-3-oxo-butanoic_acid:0.00
2	3	P05160	E.GWSPEPR.C	Y	41,93	827,3926	7	2,3	828,4018	1	29,1	3,49E+07		2,63E+07							1	F1:2370	80	86		
2	3	P05160	W.SPEPRCFK(-1.03)K(-1.03)CTKPDLSNGYISDVK.L	Y	39,74	2709,2778	24	8,5	904,1076	3	34,3	8,52E+06	7,53E+06			4,17E+06					9	F9:2033	82	105		K8:Lysine oxidation to aminoadipic semialdehyde:24.67;K9:Lysine oxidation to aminoadipic semialdehyde:30.72
2	3	P05160	W.SPEPRCFKK(-1.03)CTK(-1.03)PDLSNGYISDVK.L	Y	37,52	2709,2778	24	5,2	904,1046	3	34,6							1,61E+06			63	F63:2089	82	105		K9:Lysine oxidation to aminoadipic semialdehyde:0.00;K12:Lysine oxidation to aminoadipic semialdehyde:0.00
2	3	P05160	E.P(+27.99)RCFKKCTK(+15.99)PDLSNGYISDVK.L	Y	35,42	2442,2036	21	-15	815,063	3	31,9			5,34E+05							25	F25:1778	85	105		P1:Formylation:0.00;K9:Oxidation or Hydroxylation:0.00
2	3	P05160	P.RCFK(+43.99)K(+43.01)CTKPDLSNGYISDVK.L	Y	46,82	2388,156	20	3	1195,089	2	32,			0,00E+00		4,86E+05			5,61E+05		64	F64:192	86	105		K4:Carboxylation

						5			1		8											2				(DKW):8.69;K5:Carbamylation:3.56
2	3	P05160	P.RCFK(+43.01)K(+43.99)CTKPDLSNGYISDVK.L	Y	46,38	2388,1565	20	10	1195,0979	2	32,6	1,99E+06			6,32E+05	5,32E+05					29	F29:1906	86	105		K4:Carbamylation:0.00;K5:Carboxylation (DKW):8.14
2	3	P05160	R.CFK(+31.99)KC(-33.99)TKPDLSNGYISDVK.L	Y	84,91	2143,062	19	2,4	1072,5409	2	31,1			3,15E+05		5,00E+06		7,29E+05	7,63E+06		55	F55:1776	87	105		K3:Dihydroxy:0.00;C5:Dehydroalanine (C):9.85
2	3	P05160	R.C(+31.99)FKKC(-33.99)TKPDLSNGYISDVK.L	Y	79,68	2143,062	19	3,3	1072,5419	2	31,4			0,00E+00	1,24E+07	1,11E+07	3,85E+06	2,00E+07	9,73E+06		54	F54:1794	87	105		C1:Dihydroxy:0.00;C5:Dehydroalanine (C):12.42
2	3	P05160	R.CF(+31.99)KKC(-33.99)TKPDLSNGYISDVK.L	Y	79,41	2143,062	19	2,8	1072,5413	2	31,1				1,41E+07	1,41E+07	2,65E+06	1,71E+07			63	F63:1790	87	105		F2:Dihydroxy:0.00;C5:Dehydroalanine (C):0.00
2	3	P05160	R.CFKK(+31.99)C(-33.99)TKPDLSNGYISDVK.L	Y	77,66	2143,062	19	-0,6	1072,5376	2	31,5				2,75E+06	5,81E+06	2,80E+05	6,82E+06	0,00E+00		52	F52:1796	87	105		K4:Dihydroxy:6.63;C5:Dehydroalanine (C):5.55
2	3	P05160	R.C(-33.99)F(+31.99)KKCTKPDLSNGYISDVK.L	Y	70,78	2143,062	19	1,4	1072,5398	2	31,3	0,00E+00				8,41E+06	1,54E+06	8,97E+06	6,76E+06		68	F68:1774	87	105		C1:Dehydroalanine (C):4.73;F2:Dihydroxy:0.00
2	3	P05160	R.CFKKCT(-2.02)KPDLSNGYISDVK.L	Y	70,78	2143,0442	19	-1,7	1072,5276	2	28,4	0,00E+00	1,98E+06	1,00E+07	3,27E+07	3,20E+07		1,55E+07	1,10E+07		6	F6:2476	87	105		T6:2-amino-3-oxo-butanoic_acid:86.16
2	3	P05160	R.C(-33.99)FK(+31.99)KCTKPDLSNGYISDVK.L	Y	68,69	2143,062	19	3,5	1072,542	2	31,4								2,26E+06		70	F70:1792	87	105		C1:Dehydroalanine (C):11.01;K3:Dihydroxy:0.00
2	3	P05160	R.C(-33.99)FKKC(+31.99)TKPDLSNGYISDVK.L	Y	66,96	2143,062	19	1,1	1072,5394	2	31,1				8,09E+06			5,64E+06			60	F60:1760	87	105		C1:Dehydroalanine (C):8.87;C5:Dihydroxy:0.00
2	3	P05160	R.C(+27.99)FK(+14.96)K(-1.03)CTKPDLSNGYISDVK.L	Y	64,84	2186,9863	19	7,4	1094,5085	2	31,1	9,81E+06	9,12E+06	4,35E+06	4,80E+06	9,55E+06	6,55E+05	4,79E+06	2,59E+06		17	F17:1737	87	105		C1:Formylation:27.94;K3:Alpha-amino adipic acid:0.00;K4:Lysine oxidation to aminoadipic semialdehyde:0.00
2	3	P05160	R.C(+27.99)FK(+27.99)K(-1.03)CTKPDLSNGYISDVK.L	Y	62,91	2200,0181	19	9,4	1101,0266	2	30,8	9,26E+06	5,42E+06	2,73E+06	9,56E+06	5,77E+06		3,15E+06	6,70E+05		17	F17:1718	87	105		C1:Formylation:17.08;K3:Formylation:0.00;K4:Lysine oxidation to aminoadipic semialdehyde:0.00

2	3	P05160	R.C(-33.99)FK(+43.99)KCTKPDLSNGYISDVK.L	Y	61,46	2155,062	19	12	1078,5509	2	32,4		4,68E+06	5,46E+05	3,66E+05	3,30E+06			2,87E+05		44	F44:1873	87	105		C1:Dehydroalanine (C):0.00;K3:Carboxylation (DKW):8.69
2	3	P05160	R.CFK(+43.99)KC(-33.99)TKPDLSNGYISDVK.L	Y	59,98	2155,062	19	10	719,3687	3	32,2	0,00E+00	3,02E+06		6,01E+06	7,04E+06		1,04E+06	8,94E+05		38	F38:1870	87	105		K3:Carboxylation (DKW):0.00;C5:Dehydroalanine (C):4.08
2	3	P05160	R.CFKKCT(-2.02)KPDLSN(+.98)GYISDVK.L	Y	57,99	2144,0281	19	14	1073,0358	2	31,6				7,61E+05	4,65E+03			1,15E+05		69	F69:1792	87	105	Deamidation (NQ)	T6:2-amino-3-oxo-butanoic_acid:68.60;N12:Deamidation (NQ):1000.00
2	3	P05160	R.CFK(+42.01)K(+14.96)CT(-2.02)KPDLSNGYISDVK.L	Y	57,39	2200,0181	19	5,7	1101,0226	2	30,9	4,10E+06			1,13E+06	4,81E+05		2,05E+06	1,35E+06		43	F43:1753	87	105		K3:Acetylation (K):0.00;K4:Alpha-amino adipic acid:0.00;T6:2-amino-3-oxo-butanoic_acid:65.71
2	3	P05160	R.C(+43.01)FKK(-1.03)CTKPDLSNGYISDVK.L	Y	52,47	2187,0339	19	-8,2	1094,5153	2	31		2,27E+06			7,25E+05					37	F37:1785	87	105		C1:Carbamylation:0.00;K4:Lysine oxidation to aminoadipic semialdehyde:0.00
2	3	P05160	R.CFK(+15.99)K(+27.99)C(-33.99)TKPDLSNGYISDVK.L	Y	52,22	2155,062	19	8,4	719,3673	3	32	5,40E+06									8	F8:1816	87	105		K3:Oxidation or Hydroxylation:21.94;K4:Formylation:3.64;C5:Dehydroalanine (C):13.63
2	3	P05160	R.CFKK(+43.99)C(-33.99)TKPDLSNGYISDVK.L	Y	50,54	2155,062	19	8,7	719,3676	3	32,1			2,57E+06	2,15E+06	2,78E+06		4,15E+06	4,35E+06		34	F34:1863	87	105		K4:Carboxylation (DKW):0.00;C5:Dehydroalanine (C):6.65
2	3	P05160	R.C(-33.99)FKK(+43.99)CTKPDLSNGYISDVK.L	Y	49,59	2155,062	19	9,4	719,368	3	32,5		2,26E+06			1,00E+06			5,85E+05		70	F70:1881	87	105		C1:Dehydroalanine (C):0.00;K4:Carboxylation (DKW):0.00
2	3	P05160	R.C(+27.99)FK(-1.03)K(+27.99)CTKPDLSNGYISDVK.L	Y	49,34	2200,0181	19	3	1101,0197	2	30,9			1,26E+05	1,57E+06	2,10E+05					42	F42:1743	87	105		C1:Formylation:16.36;K3:Lysine oxidation to aminoadipic semialdehyde:0.00;K

																									4:Formylation:0.00
2	3	P05160	R.CFK(+14.96)K(+42.01)CT(-2.02)KPDLSNGYISDVK.L	Y	49,03	2200,0181	19	5,3	1101,0221	2	30,9	2,37E+06	2,21E+06	1,36E+06		6,28E+05		8,60E+04	1,33E+06	44	F44:1749	87	105		K3:Alpha-amino adipic acid:8.69;K4:Acetylation (K):7.64;T6:2-amino-3-oxo-butanoic_acid:68.60
2	3	P05160	R.C(+27.99)FK(-1.03)K(+14.96)CTKPDLSNGYISDVK.L	Y	47,62	2186,9863	19	9,3	1094,5106	2	30,7	4,45E+06			6,75E+06	9,93E+05		9,58E+05	1,70E+06	9	F9:1723	87	105		C1:Formylation:23.15;K3:Lysine oxidation to aminoadipic semialdehyde:0.00;K4:Alpha-amino adipic acid:0.00
2	3	P05160	R.CFKKC(-33.99)TK(+43.99)PDLSNGYISDVK.L	Y	46,99	2155,062	19	9,4	719,368	3	32,1	4,86E+06	0,00E+00		3,07E+06			9,37E+05		32	F32:1840	87	105		C5:Dehydroalanine (C):0.00;K7:Carboxylation (DKW):0.00
2	3	P05160	R.CFK(+43.01)K(-1.03)CTKPDLSNGYISDVK.L	Y	43,5	2187,0339	19	-9,3	730,0118	3	31,2		3,74E+05							17	F17:1740	87	105		K3:Carbamylation:0.00;K4:Lysine oxidation to aminoadipic semialdehyde:0.00
2	3	P05160	R.CFKK(+43.99)CT(-2.02)KPDLSNGYISDVK.L	Y	40,56	2187,0339	19	-12	1094,5117	2	31			2,20E+06			6,57E+05			27	F27:1775	87	105		K4:Carboxylation (DKW):12.28;T6:2-amino-3-oxo-butanoic_acid:52.68
2	3	P05160	R.C(-33.99)FKKCTK(+31.99)PDLSNGYISDVK.L	Y	39,51	2143,062	19	-2,5	715,3595	3	31,5						1,04E+06			52	F52:1794	87	105		C1:Dehydroalanine (C):0.00;K7:Dihydroxy:0.00
2	3	P05160	R.CFK(+15.99)KCTKPDLSNGYISDVK.L	Y	36,95	2161,0547	19	4,7	721,3622	3	30,9				0,00E+00					32	F32:1740	87	105		K3:Oxidation or Hydroxylation:17.01
2	3	P05160	F.KK(+43.99)C(+47.98)TKPDLSNGYISDVK.L	Y	56,22	1986,9568	17	-0,6	994,4851	2	30,7					1,08E+06			3,85E+05	18	F18:1718	89	105	Cysteine oxidation to cysteic acid	K2:Carboxylation (DKW):0.00;C3:Cysteine oxidation to cysteic acid:1000.00
2	3	P05160	F.K(+43.99)KC(+47.98)TKPDLSNGYISDVK.L	Y	44,66	1986,9568	17	-1,5	994,4842	2	30,8							9,42E+05		16	F16:1731	89	105	Cysteine oxidation to cysteic acid	K1:Carboxylation (DKW):0.00;C3:Cysteine

																										oxidation to cysteic acid:1000. 00
2	3	P051 60	K.KC(- 33.99)T(+79.96)KPDLSNGYISDVK.L	Y	65, 3	1812 ,856 3	16	9, 1	907, 4437	2	3 0, 2	3,22E+06	4,78E+06								12	F12: 245 6	90	105	Dehydroal anine (C)	C2:Dehydr oalanine (C):1000.0 0;T3:Sulfat ion:38.30
2	3	P051 60	K.C(+31.99)T(- 2.02)KPDLSNGYISDVK.L	Y	88, 87	1668 ,766 5	15	8	835, 3972	2	3 1	6,20E+06	8,17E+06	0,00E+ 00						0,00E+ 00	12	F12: 252 2	91	105		C1:Dihydro xy:0.00;T2: 2-amino-3- oxo- butanoic_ acid:95.62
2	3	P051 60	K.C(+47.98)TKPDLSNGYISDVK.L	Y	87, 93	1686 ,777	15	7, 9	844, 4024	2	3 0, 3					3,31E+0 4					12	F12: 246 2	91	105	Cysteine oxidation to cysteic acid	C1:Cystein e oxidation to cysteic acid:1000. 00
2	3	P051 60	K.CTK(+42.01)P(- 30.01)DLSNGYISDVK.L	Y	72, 71	1650 ,792 2	15	4, 9	826, 4074	2	3 3, 2				3,87E+0 7	1,38E+0 7		4,34E+0 7	2,43E+0 7		55	F55: 195 9	91	105	Proline oxidation to pyrrolidin one	K3:Acetyla tion (K):169.05; P4:Proline oxidation to pyrrolidino ne:1000.00
2	3	P051 60	K.C(+27.99)TKPDLSNGYISDVK.L	Y	62, 65	1666 ,787 2	15	7, 9	834, 4075	2	3 3, 5		1,86E+06		2,17E+0 7	9,83E+0 6		1,73E+0 7	3,72E+0 6		65	F65: 198 1	91	105		C1:Formyl ation:0.00
2	3	P051 60	K.C(+31.99)TKPDLSNGYISDVK.L	Y	56, 32	1670 ,782 1	15	7, 2	836, 4044	2	3 2, 9	1,37E+07		4,02E+ 06	1,74E+0 7	7,20E+0 6		9,05E+0 6	3,39E+0 6		30	F30: 191 9	91	105		C1:Dihydro xy:0.00
2	3	P051 60	K.CT(- 2.02)K(+31.99)PDLSNGYISDVK.L	Y	52, 45	1668 ,766 5	15	7	835, 3964	2	3 4, 4			3,52E+ 05		1,07E+0 5					25	F25: 196 0	91	105		T2:2- amino-3- oxo- butanoic_ acid:61.26; K3:Dihydr oxy:0.00
2	3	P051 60	K.C(+27.99)(- 33.99)TK(+43.99)PDLSNGYISDVK.L	Y	52	1676 ,789 3	15	6, 9	839, 4077	2	3 3, 5								1,70E+0 6		72	F72: 195 5	91	105		C1:Formyl ation:135. 57;C1:Deh ydroalanin e (C):1000.0 0;K3:Carbo xylation (DKW):36. 05
2	3	P051 60	K.C(+27.99)(+47.98)TK(+43.01)PDLSN GYISDVK.L	Y	48, 4	1757 ,777 7	15	8, 8	879, 9039	2	2 9, 6	1,08E+06									4	F4:2 591	91	105		C1:Formyl ation:149. 52;C1:Cyst eine oxidation to cysteic acid:1000. 00;K3:Carb amylation: 151.17
2	3	P051 60	K.CTK(+31.99)PDLSNGYISDVK.L	Y	47, 61	1670 ,782 1	15	7, 1	836, 4043	2	3 3, 2		3,51E+06					2,17E+0 6			17	F17: 190 4	91	105		K3:Dihydr oxy:0.00
2	3	P051 60	K.CTKPDLSNGYISDVK.L	Y	40, 63	1638 ,792 2	15	- 8, 9	820, 3961	2	3 3, 6								0,00E+0 0		67	F67: 196 8	91	105		
2	3	P051 60	K.CTK(+27.99)PDLSNGYISDVK.L	Y	39, 02	1666 ,787 2	15	8, 8	834, 4083	2	3 3, 3				2,63E+0 6						35	F35: 195 9	91	105		K3:Formyl ation:0.00
2	3	P051 60	C.TKPDLSNGYISDVK.L	Y	70, 84	1535 ,783 1	14	8, 8	768, 9056	2	2 8, 8	2,74E+06									3	F3:2 344	92	105		
2	3	P051	K.PDLSNGYISDVK.L	Y	81,	1306	12	7,	654,	2	2	1,33E+07	9,12E+06	7,32E+	2,98E+0	1,60E+0	5,05E+0	2,47E+0	1,46E+0	2,52E+	2	F2:2	94	105		

		60			38	,640 5		7	3326		9, 8			06	7	7	6	7	7	06		432				
2	3	P051 60	P.DLSNGYISDVK.L	Y	59, 31	1209 ,587 6	11	- 2, 1	605, 7998	2	3 0, 7	0,00E+00		0,00E+ 00							24	F24: 265 6	95	105		
2	3	P051 60	D.LSNGYISDVK.L	Y	62, 98	1094 ,560 8	10	0, 8	548, 2881	2	2 8, 5	3,03E+06	2,15E+06	0,00E+ 00							24	F24: 243 8	96	105		
2	3	P051 60	N.GYISDVK.L	Y	55, 12	780, 4017	7	0, 4	391, 2083	2	2 6, 9	3,38E+05	3,82E+05	0,00E+ 00							6	F6:2 339	99	105		
2	3	P051 60	G.YISDVK.L	Y	35, 16	723, 3802	6	2, 3	724, 3892	1	2 0, 1			4,68E+ 04							24	F24: 163 1	100	105		
2	3	P051 60	K.IQENMR.Y	Y	57, 04	789, 3803	6	-3	395, 6963	2	1 5, 7	4,48E+06	1,79E+06	2,57E+ 06							15	F15: 123 1	110	115		
2	3	P051 60	K.I(+27.99)QEN(+.98)M(-48.00)RYGCASGYK.T	Y	37, 3	1599 ,698 7	14	- 0, 2	800, 8564	2	3 1, 4									3,76E+ 05	77	F77: 180 3	110	123	Dethiomet hyl	I1:Formyla tion:106.2 5;N4:Dea midation (NQ):0.00; M5:Dethio methyl:10 00.00
2	3	P051 60	K.IQ(+.98)ENMR.Y	Y	35, 35	790, 3643	6	- 1, 6	396, 1888	2	1 9, 9	1,73E+05									4	F4:1 650	110	115		Q2:Deami dation (NQ):54.01
2	3	P051 60	K.TTGGKDEEVVQC(-33.99)LSDGWSSQP(+31.99)TCR.K	Y	63, 84	2580 ,139 9	24	1, 6	1291 ,079 3	2	3 5, 2	6,51E+07		3,48E+ 06		2,11E+0 8				1,05E+ 07	40	F40: 210 9	124	147		C12:Dehyd roalanine (C):30.64;P 21:Dihydro xy:0.00
2	3	P051 60	K.TTGGKDEEVVQC(-33.99)LSDGWSSQPTCR(+31.99).K	Y	58, 63	2580 ,139 9	24	0, 2	1291 ,077 5	2	3 5, 1		0,00E+00		0,00E+0 0	8,73E+0 7				4,43E+ 06	39	F39: 212 4	124	147		C12:Dehyd roalanine (C):36.25;R 24:Dihydro xy:0.00
2	3	P051 60	K.TTGGKDEEVVQC(-33.99)LSDGWSSQPTC(+31.99)R.K	Y	56, 67	2580 ,139 9	24	1, 2	1291 ,078 7	2	3 5	1,04E+08	7,05E+07	7,89E+ 07	0,00E+0 0	1,25E+0 8		5,87E+0 7	2,82E+0 7		61	F61: 204 9	124	147		C12:Dehyd roalanine (C):21.04;C 23:Dihydro xy:0.00
2	3	P051 60	K.TTGGKDEEVVQC(+31.99)LSDGWSS QPTC(-33.99)R.K	Y	47, 77	2580 ,139 9	24	1, 1	1291 ,078 6	2	3 5, 3									5,47E+ 06	81	F81: 211 6	124	147		C12:Dihydr oxy:0.00;C 23:Dehydr oalanine (C):0.00
2	3	P051 60	K.TTGGKDEEVVQCLSDGWS(-2.02)SQPTCR.K	Y	47, 15	2580 ,122 1	24	1, 8	1291 ,070 7	2	3 5, 7		2,03E+08							3,21E+ 04	75	F75: 197 0	124	147		S18:2-amino-3-oxo-butanoic_ acid:0.00
2	3	P051 60	K.TTGGKDEEVVQ(+.98)CLS(-2.02)DGWSSQPTCR.K	Y	47, 01	2581 ,106	24	- 2, 6	1291 ,556 9	2	3 3	0,00E+00									6	F6:2 908	124	147		Q11:Deam idation (NQ):14.78 ;S14:2-amino-3-oxo-butanoic_ acid:0.00
2	3	P051 60	K.TTGGKDEEVVQ.C	Y	45, 28	1161 ,551 3	11	- 1, 7	581, 7819	2	2 0	3,18E+05	6,07E+04	1,71E+ 05							5	F5:1 664	124	134		
2	3	P051 60	K.TTGGKDEEVVQCLSDGWSSQPT(-2.02)CR.K	Y	43, 44	2580 ,122 1	24	- 2, 2	861, 0461	3	3 1, 7	0,00E+00		1,16E+ 08							6	F6:2 790	124	147		T22:2-amino-3-oxo-butanoic_ acid:0.00
2	3	P051 60	K.TTGGKDEEVVQCLSDGWSSQP(+13.9 8)TC(-33.99)R.K	Y	42, 84	2562 ,129 2	24	-1	1282 ,070 6	2	3 5, 2								1,76E+0 6		66	F66: 212 5	124	147	Proline oxidation to pyrogluta mic acid	P21:Prolin e oxidation to pyrogluta

																										mic acid:1000.00;C23:Dehydroalanine (C):11.49
2	3	P05160	K.TTGGKDEEVVQC(+15.99)LSDGW(+13.98)SSQPTCR.K	Y	40,76	2612,1118	24	4,5	1307,0691	2	36,2								0,00E+00		70	F70:2190	124	147	Tryptophan oxidation to oxolactone	C12:Oxidation or Hydroxylation (C):0.00;W17:Tryptophan oxidation to oxolactone:1000.00
2	3	P05160	K.TTGGKDEEVVQC(-33.99)LSDGW(+13.98)SSQPTCR.K	Y	39,96	2562,1292	24	1,7	855,0518	3	35,2					0,00E+00					44	F44:2113	124	147	Tryptophan oxidation to oxolactone	C12:Dehydroalanine (C):19.79;W17:Tryptophan oxidation to oxolactone:1000.00
2	3	P05160	K.TTGGKDEEVVQC(-33.99)LSDGWSSQPTC(+47.98)R.K	Y	36,67	2596,1348	24	1,9	1299,0771	2	34,6		9,52E+05								17	F17:2026	124	147		C12:Dehydroalanine (C):11.49;C23:Cysteine oxidation to cysteic acid:11.49
2	3	P05160	K.DEEVVQC(-33.99)LSDGWSSQPTC(+31.99)R.K	Y	51,2	2135,9065	19	1,3	1068,9619	2	36,4	1,07E+06	8,31E+05	7,74E+07	1,33E+08	2,03E+08	2,17E+07	2,78E+08	1,38E+08	1,39E+07	42	F42:2202	129	147		C7:Dehydroalanine (C):20.33;C18:Dihydroxy:11.10
2	3	P05160	K.DEEVVQC(-33.99)LSDGWSSQP(+31.99)TCR.K	Y	46,86	2135,9065	19	1,6	1068,9623	2	35,7		8,82E+05		0,00E+00						17	F17:2115	129	147		C7:Dehydroalanine (C):12.53;P16:Dihydroxy:3.13
2	3	P05160	K.DEEVVQC(-33.99)LSD(+43.99)GWSSQPTCR.K	Y	46,32	2117,896	19	-0,1	1059,9552	2	36,5		2,09E+06								16	F16:2209	129	147		C7:Dehydroalanine (C):10.97;D10:Carboxylation (DKW):0.00;P16:Proline oxidation to pyrrolidinone:1000.00
2	3	P05160	K.DEEVVQC(-33.99)LSDGW(+43.99)SSQPTCR.K	Y	39,18	2117,896	19	0,3	1059,9556	2	36,6			1,68E+06							26	F26:2228	129	147		C7:Dehydroalanine (C):14.00;W12:Carboxylation (DKW):5.03;P16:Proline oxidation to pyrrolidinone:1000.00
2	3	P05160	K.DEEVVQC(-33.99)LSDGW(+15.99)SSQP(+31.99)TCR.K	Y	37,81	2151,9014	19	3,2	1076,9614	2	35,6		1,60E+06								18	F18:2128	129	147	Oxidation (HW)	C7:Dehydroalanine (C):24.15;W12:Oxidation (HW):1000.00;P16:Di

																										hydroxy:0.00
2	3	P05160	K.DEEVVQCLS(-2.02)DGWSSQPTCR.K	Y	37,77	2135,8889	19	2,7	1068,9546	2	58,4	8,13E+04	1,85E+05								9	F9:3664	129	147		S9:2-amino-3-oxo-butanoic_acid:4.51
2	3	P05160	K.DEEVVQCLSDGWSS(-2.02)QPTCR.K	Y	37,67	2135,8889	19	2,3	1068,9542	2	34,4			0,00E+00							22	F22:2988	129	147		S14:2-amino-3-oxo-butanoic_acid:0.00
2	3	P05160	K.DEEVVQCLSDGWSSQPT(-2.02)CR.K	Y	37,4	2135,8889	19	-1,1	1068,9506	2	34,4	5,14E+06	1,40E+08								4	F4:3032	129	147		T17:2-amino-3-oxo-butanoic_acid:7.77
2	3	P05160	K.DEEVVQC(-33.99)LSDGWSSQP(+13.98)TCR.K	Y	36,88	2117,896	19	-3,1	1059,952	2	36,6								9,62E+05		65	F65:2244	129	147	Proline oxidation to pyroglutamic acid	C7:Dehydroalanine (C):7.03;P16:Proline oxidation to pyroglutamic acid:1000.00
2	3	P05160	K.DEEVVQC(-33.99)LSD(+15.99)GWSSQP(+31.99)TCR.K	Y	36,85	2151,9014	19	2,8	1076,9609	2	35,9			4,36E+05							25	F25:2078	129	147		C7:Dehydroalanine (C):7.06;D10:Oxidation or Hydroxylation:7.81;P16:Dihydroxy:0.00
2	3	P05160	K.DEEVVQC(-33.99)LSDGWSSQPTCR(+31.99).K	Y	36,17	2135,9065	19	-9,6	1068,9503	2	34,4	0,00E+00									6	F6:3036	129	147		C7:Dehydroalanine (C):17.89;R19:Dihydroxy:0.00
2	3	P05160	K.DEEVVQC(+31.99)LSDGWSSQPTC(-33.99)R.K	Y	35,44	2135,9065	19	0,6	1068,9612	2	36,5					1,82E+07			1,16E+07		69	F69:2197	129	147		C7:Dihydroxy:0.00;C18:Dehydroalanine (C):0.00
2	3	P05160	F.KVKDKVQY(+15.01)EC(+67.92)ATGYYTAGGK.K	Y	37,25	2291,0215	20	10	1146,5298	2	31,3		0,00E+00								17	F17:1752	171	190		Y8:Tyrosine oxidation to 2-aminotyrosine:69.64;C10:Dichlorination of tyrosine residues:88.98
2	3	P05160	K.VKD(+79.97)KVQYEC(-33.99)ATGYYTAGGK.K	Y	69,34	2125,9722	19	14	1064,0083	2	31,4			2,52E+06		2,88E+06	0,00E+00		1,65E+06	0,00E+00	39	F39:1812	172	190	Phosphorylation (HCDR); Dehydroalanine (C)	D3:Phosphorylation (HCDR):1000.00;C9:Dehydroalanine (C):1000.00
2	3	P05160	K.VKD(+43.99)K(+14.96)VQY(+31.99)ECATGYYTAGGK.K	Y	59,51	2170,9365	19	6,7	1086,4828	2	29,6	3,46E+06	1,86E+06	1,30E+06	1,10E+06	3,01E+05		8,68E+05		2,06E+05	28	F28:1656	172	190		D3:Carboxylation (DKW):12.28;K4:Alpha-amino adipic acid:9.40;Y7:Dihydroxy:0.00
2	3	P051	K.VKD(+43.99)K(+14.96)VQYEC(+31.9	Y	58,	2170	19	8,	1086	2	2	1,26E+06			2,01E+0	9,70E+0	1,13E+0	3,87E+0	1,76E+0		29	F29:	172	190		D3:Carbox

		60	9)ATGYYTAGGK.K		93	,936<br>5		1	,484<br>4		9,<br>6				6	5	5	5	6		165<br>2				ylation<br>(DKW):10.<br>11;K4:Alph<br>a-amino<br>adipic<br>acid:7.32;C<br>9:Dihydrox<br>y:0.00
2	3	P051<br>60	K.VK(-1.03)DK(-<br>1.03)VQY(+79.97)ECATGYYTAGGK.K	Y	53,<br>4	2157<br>,896<br>5	19	8,<br>9	1079<br>,965<br>1	2	2<br>9,<br>9	2,51E+06			2,42E+0<br>6	8,43E+0<br>5			9,41E+0<br>5	29	F29:<br>167<br>6	172	190		K2:Lysine<br>oxidation<br>to<br>aminoadip<br>ic<br>semialdeh<br>yde:114.35<br>;K4:Lysine<br>oxidation<br>to<br>aminoadip<br>ic<br>semialdeh<br>yde:110.25<br>;Y7:Phosp<br>horylation<br>(STY):16.5<br>1
2	3	P051<br>60	K.VKD(+43.99)K(-<br>1.03)VQYEC(+47.98)ATGYYTAGGK.K	Y	52,<br>18	2170<br>,936<br>5	19	2,<br>6	1086<br>,478<br>4	2	2<br>9,<br>8							0,00E+0<br>0		61	F61:<br>165<br>4	172	190	Cysteine<br>oxidation<br>to cysteic<br>acid	D3:Carbox<br>ylation<br>(DKW):10.<br>11;K4:Lysi<br>ne<br>oxidation<br>to<br>aminoadip<br>ic<br>semialdeh<br>yde:7.32;C<br>9:Cysteine<br>oxidation<br>to cysteic<br>acid:1000.<br>00
2	3	P051<br>60	K.VK(+14.96)DK(+27.99)VQYEC(+47.9<br>8)ATGYYTAGGK.K	Y	51,<br>91	2170<br>,936<br>5	19	4,<br>5	1086<br>,480<br>5	2	2<br>9,<br>9		5,27E+05							17	F17:<br>165<br>2	172	190	Cysteine<br>oxidation<br>to cysteic<br>acid	K2:Alpha-<br>amino<br>adipic<br>acid:5.99;K<br>4:Formylat<br>ion:4.52;C<br>9:Cysteine<br>oxidation<br>to cysteic<br>acid:1000.<br>00
2	3	P051<br>60	K.VKD(+43.99)KVQY(+67.92)EC(-<br>33.99)ATGYYTAGGK.K	Y	49,<br>98	2157<br>,917<br>7	19	1,<br>2	1079<br>,967<br>4	2	3<br>0,<br>2			5,37E+<br>05					0,00E+0<br>0	64	F64:<br>170<br>4	172	190	Dehydroal<br>anine (C)	D3:Carbox<br>ylation<br>(DKW):0.0<br>0;Y7:Dichl<br>orination<br>of tyrosine<br>residues:7<br>1.27;C9:De<br>hydroalani<br>ne<br>(C):1000.0<br>0
2	3	P051<br>60	K.VK(+43.99)DK(+14.96)VQYEC(+31.9<br>9)ATGYYTAGGK.K	Y	49,<br>73	2170<br>,936<br>5	19	6,<br>3	1086<br>,482<br>4	2	2<br>9,<br>8						1,69E+0<br>5	0,00E+0<br>0		63	F63:<br>168<br>6	172	190		K2:Carbox<br>ylation<br>(DKW):0.0<br>0;K4:Alpha<br>-amino<br>adipic<br>acid:0.00;C<br>9:Dihydrox<br>y:8.18
2	3	P051<br>60	K.VKDKVQYE.C	Y	48,<br>69	1007<br>,528	8	2,<br>9	504,<br>7731	2	2<br>5,			1,39E+<br>07						19	F19:<br>207	172	179		

						7					5										2				
2	3	P051 60	K.VKDKVQY(+44.99)EC(+31.99)ATGY YTAGGK.K	Y	47, 59	2200 ,958 3	19	11	1101 ,498 3	2	2 9, 6		1,21E+ 06				0,00E+0 0	0,00E+0 0		64	F64: 165 7	172	190		Y7:Oxidati on to nitro:69.64 ;C9:Dihydr oxy:14.62
2	3	P051 60	K.VK(+43.99)DKVQY(+67.92)EC(- 33.99)ATGYYTAGGK.K	Y	47, 24	2157 ,917 7	19	- 0, 5	1079 ,965 6	2	3 0, 1	1,36E+06								18	F18: 167 0	172	190	Dehydroal anine (C)	K2:Carbox ylation (DKW):0.0 0;Y7:Dichl orination of tyrosine residues:9 0.32;C9:De hydroalani ne (C):1000.0 0
2	3	P051 60	K.VK(-1.03)D(+79.97)K(- 1.03)VQYECATGYYTAGGK.K	Y	45, 38	2157 ,896 5	19	9, 3	1079 ,965 6	2	3 0, 2							9,93E+0 5		65	F65: 170 3	172	190		K2:Lysine oxidation to aminoadip ic semialdeh yde:137.15 ;D3:Phosp horylation (HCDR):0.0 0;K4:Lysin e oxidation to aminoadip ic semialdeh yde:148.55
2	3	P051 60	K.VKD(+79.97)KVQY(+44.99)EC(- 33.99)ATGYYTAGGK.K	Y	43, 94	2170 ,957 3	19	4, 1	1086 ,490 4	2	3 0							0,00E+0 0		71	F71: 168 6	172	190	Phosphory lation (HCDR); Dehydroal anine (C)	D3:Phosph orylation (HCDR):10 00.00;Y7:O xidation to nitro:72.73 ;C9:Dehydr oalanine (C):1000.0 0
2	3	P051 60	K.VK(+14.96)D(+43.99)KVQY(+31.99) ECATGYYTAGGK.K	Y	42, 83	2170 ,936 5	19	5, 8	1086 ,481 8	2	2 9, 9					2,24E+0 5				48	F48: 166 8	172	190		K2:Alpha- amino adipic acid:0.00; D3:Carbox ylation (DKW):0.0 0;Y7:Dihyd roxy:11.10
2	3	P051 60	K.VKDK(+43.99)VQY(+67.92)EC(- 33.99)ATGYYTAGGK.K	Y	42, 46	2157 ,917 7	19	- 0, 2	1079 ,965 9	2	3 0, 2	2,92E+05	1,24E+ 06							27	F27: 170 9	172	190	Dehydroal anine (C)	K4:Carbox ylation (DKW):0.0 0;Y7:Dichl orination of tyrosine residues:7 8.19;C9:De hydroalani ne (C):1000.0 0
2	3	P051 60	K.VKDKVQY(- 2.02)EC(+79.96)ATGYYTAGGK.K	Y	40, 78	2157 ,934 6	19	- 7, 6	1079 ,966 3	2	3 0, 5		7,98E+ 04							25	F25: 167 8	172	190	O- Sulfonatio n (C)	Y7:2- amino-3- oxo- butanoic_ acid:49.79; C9:O- Sulfonatio n

																										(C):1000.00
2	3	P05160	K.VK(+31.99)D(+43.99)K(+14.96)VQYECATGYYTAGGK.K	Y	40,48	2170,9365	19	8,5	1086,4847	2	29,8		9,41E+05			6,77E+05					38	F38:1671	172	190		K2:Dihydroxy:0.00;D3:Carboxylation (DKW):129.22;K4:Alpha-amino adipic acid:0.00
2	3	P05160	K.VKDKVQY(+79.97)EC(-33.99)ATGYYTAGGK.K	Y	38,01	2125,9722	19	13	709,6742	3	31,1										30	F30:1772	172	190	Dehydroalanine (C)	Y7:Phosphorylation (STY):25.18;C9:Dehydroalanine (C):1000.00
2	3	P05160	K.VK(-1.03)DK(-1.03)VQYECAT(+79.97)GYYTAGGK.K	Y	36,69	2157,8965	19	9,1	1079,9653	2	30				0,00E+00						28	F28:1682	172	190		K2:Lysine oxidation to aminoadipic semialdehyde:88.40;K4:Lysine oxidation to aminoadipic semialdehyde:70.02;T11:Phosphorylation (STY):0.00
2	3	P05160	K.VKD(+43.99)KVQY(+15.99)EC(+31.99)ATGYYTAGGK.K	Y	35,53	2171,968	19	7,6	1086,9995	2	30,9		0,00E+00								18	F18:1732	172	190		D3:Carboxylation (DKW):0.00;Y7:Oxidation or Hydroxylation:0.00;C9:Dihydroxy:8.82
2	3	P05160	K.DKVQYEC(+47.98)ATGYYTAGGK.K	Y	113,7	1900,8148	17	9,8	951,424	2	28,8		3,94E+06								2	F2:2342	174	190	Cysteine oxidation to cysteic acid	C7:Cysteine oxidation to cysteic acid:1000.00
2	3	P05160	K.DK(+42.01)VQY(+15.01)EC(+47.98)ATGYYTAGGK.K	Y	46,41	1957,8363	17	12	979,9373	2	29,5	0,00E+00	3,60E+07	3,25E+07		0,00E+00	0,00E+00		1,33E+08	1,29E+08	62	F62:1628	174	190	Cysteine oxidation to cysteic acid	K2:Acetylation (K):148.55;Y5:Tyrosine oxidation to 2-aminotyrosine:72.73;C7:Cysteine oxidation to cysteic acid:1000.00
2	3	P05160	K.D(+43.01)(+15.99)KVQYEC(+31.99)ATGYYTAGGK.K	Y	42,89	1943,8207	17	-1,2	972,9164	2	29,9		1,95E+05								16	F16:1659	174	190		D1:Carbamylation:0.00;D1:Oxidation or Hydroxylation:0.00;C7:Dihydroxy:0.00
2	3	P05160	K.D(+43.01)K(+15.99)VQY(+31.99)ECATGYYTAGGK.K	Y	41,66	1943,820	17	-2,	972,9156	2	29,		2,09E+05	9,35E+04							18	F18:165	174	190		D1:Carbamylation:0

						7		1			9											4				.00;K2:Oxidation or Hydroxylation:0.00;Y5:Dihydroxy:13.67
2	3	P05160	K.DK(+42.01)VQY(+15.01)EC(+47.98)ATGYYTAGGKK.T	Y	39,96	2085,9314	18	10	1043,9834	2	26,3		4,71E+05								14	F14:2227	174	191	Cysteine oxidation to cysteic acid	K2:Acetylation (K):124.59;Y5:Tyrosine oxidation to 2-aminotyrosine:47.92;C7:Cysteine oxidation to cysteic acid:1000.00
2	3	P05160	K.VQYEC(-33.99)ATGYYTAGGK.K	Y	54,5	1575,7205	15	8,2	788,874	2	32,4									3,25E+05	73	F73:1866	176	190	Dehydroalanine (C)	C5:Dehydroalanine (C):1000.00
2	3	P05160	C.ATGYYTAGGK.K	Y	51,37	987,4661	10	4,9	494,7427	2	25,2	3,28E+05	9,86E+05								12	F12:2012	181	190		
2	3	P05160	A.TGYYTAGGK.K	Y	53,51	916,429	9	6,2	459,2246	2	24,2	2,59E+05	2,38E+05								11	F11:1931	182	190		
2	3	P05160	T.A(+43.01)GGKK(+31.99)TEEVECLTYGWSLTPK.C	Y	39,73	2371,1365	21	12	1186,5894	2	35,9	1,69E+06									9	F9:2162	187	207		A1:Carbamylation:0.00;K5:Dihydroxy:0.00
2	3	P05160	K.KTEEVEC(+47.98)LTYGWSLTPK.C	Y	62,68	2046,9456	17	11	1024,4917	2	33		8,51E+05								11	F11:2684	191	207		C1:Cysteine oxidation to cysteic acid:0.00
2	3	P05160	K.T(+43.01)EEVECLTYGWSLTPK.C	Y	57,77	1897,8767	16	12	633,6403	3	35,4	2,81E+07		4,06E+07							19	F19:3072	192	207		T1:Carbamylation:73.26
2	3	P05160	L.RLIENGYFHPVK.Q	Y	53,88	1471,7936	12	7,4	491,6088	3	29,6	4,16E+05	9,14E+05								12	F12:2403	217	228		
2	3	P05160	R.LIENGYFHPVK.Q	Y	79,65	1315,6924	11	3,6	658,8558	2	32,4	3,91E+08	3,27E+08	1,70E+08	3,27E+08	1,65E+08	2,15E+08	3,10E+08	1,72E+08	2,71E+08	30	F30:1878	218	228		
2	3	P05160	R.LIEN(+.98)GYFHPVK.Q	Y	75,9	1316,6764	11	-2,7	659,3437	2	30,7	3,70E+07	3,32E+07	3,52E+06	1,79E+07	4,86E+06	7,98E+06	1,74E+07	5,95E+06	1,32E+07	6	F6:2702	218	228	Deamidation (NQ)	N4:Deamidation (NQ):1000.00
2	3	P05160	L.IENGYFHPVK.Q	Y	56,79	1202,6084	10	7,7	602,3161	2	33,1		1,81E+06		3,61E+06	4,02E+06	4,04E+06	2,44E+06	5,86E+06	4,80E+06	69	F69:1912	219	228		
2	3	P05160	I.ENGYFHPVK.Q	Y	48,19	1089,5243	9	0,5	545,7697	2	30,2		0,00E+00		2,94E+06		6,41E+05	3,01E+06		9,78E+05	13	F13:2573	220	228		
2	3	P05160	E.NGYFHPVK.Q	Y	53,55	960,4817	8	2,4	481,2493	2	28,9	3,41E+07	2,79E+07	1,22E+07							19	F19:2425	221	228		
2	3	P05160	E.N(+.98)GYFHPVK.Q	Y	46,26	961,4658	8	2,4	481,7413	2	29,9	2,98E+07	3,62E+07	2,56E+07							10	F10:2445	221	228	Deamidation (NQ)	N1:Deamidation (NQ):1000.00
2	3	P05160	W.YPESP(+15.99)VCEGR(+15.99)RNRC(+31.99)PPPPLPINSK.I	Y	37,85	2769,3325	24	2,4	924,1204	3	33,6			2,76E+06							26	F26:1982	261	284		P5:Oxidation or Hydroxylation:0.00;R10:Oxidation or Hydroxylation:9.34;C

																										14:Dihydroxy:5.08
2	3	P05160	W.YPESP(+31.99)VCEGRRNRC(+31.99)PPPPLPINSK.I	Y	35,83	2769,3325	24	4,7	924,1225	3	33,4				4,25E+06						29	F29:1972	261	284		P5:Dihydroxy:0.00;C14:Dihydroxy:17.01
2	3	P05160	P.VCEGRRNRCPPPPLPINSK.I	Y	39,8	2132,1094	19	12	1067,0751	2	33						7,23E+05				52	F52:1916	266	284		
2	3	P05160	R.R(+43.01)(+15.99)NRC(+31.99)PPPPLPINSK.I	Y	66,8	1678,8573	14	-1,2	840,4349	2	30,4		1,30E+07	6,65E+06							19	F19:2578	271	284		R1:Carbamylation:183.32;R1:Oxidation or Hydroxylation:0.00;C4:Dihydroxy:33.98
2	3	P05160	R.R(+43.01)(+15.99)NR(+31.99)CPPPPLPINSK.I	Y	63,35	1678,8573	14	0,5	840,4363	2	30,8	8,37E+06									1	F1:2537	271	284		R1:Carbamylation:166.43;R1:Oxidation or Hydroxylation:0.00;R3:Dihydroxy:0.00
2	3	P05160	R.R(+27.99)(+79.97)NRCP(+13.98)PPPLPINSK.I	Y	50,56	1709,8073	14	2	855,9126	2	27,7	2,34E+07	3,30E+07	3,57E+07	1,85E+08	2,20E+08	4,08E+08	1,75E+08	3,29E+08	4,55E+08	22	F22:2343	271	284		R1:Formylation:81.10;R1:Phosphorylation (HCDR):5.47;P5:Proline oxidation to pyroglutamic acid:40.00
2	3	P05160	R.R(+27.99)NR(+79.97)CP(+13.98)PPPLPINSK.I	Y	43,14	1709,8073	14	3,1	855,9135	2	30,6						6,71E+07	4,83E+07	3,69E+07		70	F70:1724	271	284		R1:Formylation:92.20;R3:Phosphorylation (HCDR):0.00;P5:Proline oxidation to pyroglutamic acid:40.00
2	3	P05160	R.R(+27.99)NRC(+79.96)P(+13.98)PPPLPINSK.I	Y	41,24	1709,7977	14	-1,2	855,9052	2	27,8			0,00E+00							24	F24:2375	271	284	O-Sulfonation (C)	R1:Formylation:92.20;C4:O-Sulfonation (C):1000.00;P5:Proline oxidation to pyroglutamic acid:40.00
2	3	P05160	R.R(+27.99)NRC(+79.97)P(+13.98)PPPLPINSK.I	Y	36,87	1709,8073	14	2,9	570,9447	3	27,7		1,00E+07		4,39E+07				3,69E+07		15	F15:2385	271	284		R1:Formylation:49.75;C4:Phosphorylation (HCDR):0.00;P5:Proline oxidation to pyrogluta

																										mic acid:33.98
2	3	P05160	R.N(+43.01)RC(+79.96)P(-30.01)PPPLPINSK.I	Y	44,64	1524,7177	13	-4,8	763,3624	2	31,7						8,34E+06		4,94E+05	4,96E+06	68	F68:1802	272	284	O-Sulfonation (C)	N1:Carbamylation:100.30;C3:O-Sulfonation (C):1000.00;P4:Proline oxidation to pyrrolidinone:40.00
2	3	P05160	R.CPPPPLPINSK.I	Y	67,92	1209,6063	11	6,6	605,8145	2	31	2,91E+06	3,91E+06								12	F12:2524	274	284		
2	3	P05160	R.C(+15.99)P(+13.98)PPPLPINSK.I	Y	40,25	1191,5957	11	6,6	596,8091	2	32,5	7,69E+05	8,32E+05								11	F11:2644	274	284		C1:Oxidation or Hydroxylation (C):0.00;P2:Proline oxidation to pyroglutamic acid:0.00
2	3	P05160	R.CP(+13.98)P(+15.99)PPLPINSK.I	Y	35,38	1191,5957	11	8,1	596,8099	2	32,4	8,74E+05									2	F2:2650	274	284		P2:Proline oxidation to pyroglutamic acid:0.00;P3:Oxidation or Hydroxylation:0.00
2	3	P05160	C.PPPPLPINSK.I	Y	56,65	1058,6124	10	6	530,3167	2	30,6				5,96E+06	5,21E+06	6,81E+06	7,79E+06	3,60E+06	1,21E+07	52	F52:1725	275	284		
2	3	P05160	P.PPPLPINSK.I	Y	56,32	961,5596	9	5,2	481,7896	2	30,4	3,49E+05	4,67E+05	1,37E+06	2,32E+07	1,29E+07	1,49E+07	2,50E+07	1,10E+07	2,44E+07	53	F53:1711	276	284		
2	3	P05160	P.PPLPINSK.I	Y	47,51	864,5068	8	5,7	433,2632	2	30,6			1,01E+06	5,34E+06	7,21E+06	8,03E+06	5,61E+06	6,95E+06	9,86E+06	80	F80:1737	277	284		
2	3	P05160	K.IQTHSTTYR.H	Y	72,14	1105,5516	9	-0,4	553,7829	2	14,5	2,08E+08	3,07E+08	1,90E+08	4,17E+06	2,54E+07	3,53E+07	2,92E+06	2,21E+07	4,83E+07	15	F15:1122	285	293		
2	3	P05160	K.IQ(+.98)THSTTYR.H	Y	64,4	1106,5356	9	0,5	554,2754	2	17,3	1,54E+07	4,12E+06	3,43E+06	0,00E+00	0,00E+00	0,00E+00		2,36E+04		6	F6:1419	285	293	Deamidation (NQ)	Q2:Deamidation (NQ):1000.00
2	3	P05160	K.IQTHSTTY(+15.99)R.H	Y	62,84	1121,5465	9	-0,5	561,7803	2	15,1	4,51E+05	4,29E+05								13	F13:1146	285	293		Y8:Oxidation or Hydroxylation:33.98
2	3	P05160	K.IQTHSTTY.R	Y	52,46	949,4505	8	-0,4	475,7323	2	20,2	9,09E+05	8,52E+05	6,67E+06							23	F23:1655	285	292		
2	3	P05160	K.IQTHSTTYR(+31.99).H	Y	48,69	1137,5415	9	-0,9	569,7775	2	14,5		1,27E+05								15	F15:1120	285	293		R9:Dihydroxy:0.00
2	3	P05160	K.IQTHSTT.Y	Y	39,46	786,3872	7	1,7	787,3958	1	11,3	4,84E+05		8,41E+05							22	F22:795	285	291		
2	3	P05160	I.QTHSTTYR.H	Y	52,4	992,4675	8	0,8	497,2414	2	14,1	2,94E+06	1,71E+06	2,35E+06	2,32E+05	2,93E+06	1,80E+06	4,81E+04	1,74E+06	3,76E+06	24	F24:1075	286	293		
2	3	P05160	I.Q(+.98)THSTTYR.H	Y	40,38	993,4516	8	0,5	497,7333	2	17,4	3,78E+05									5	F5:1423	286	293	Deamidation (NQ)	Q1:Deamidation (NQ):1000.

																									00
2	3	P05160	Q.THSTTYR.H	Y	41,23	864,409	7	2	433,2126	2	26,4			5,12E+05	1,09E+05	8,56E+05	1,50E+06	0,00E+00	7,23E+05	1,44E+06	76	F76:1438	287	293	
2	3	P05160	T.HSTTYR.H	Y	50,48	763,3613	6	5,5	382,69	2	25,9		4,26E+04	5,23E+05	1,19E+05	2,09E+06	2,04E+06	2,30E+04	1,96E+06	2,07E+06	27	F27:1406	288	293	
2	3	P05160	R.HGEIVHIEC(+47.98)ELNFEIHGSAEIR.C	Y	106,9	2579,2073	22	10	860,7518	3	33,8	1,79E+06	3,33E+06			8,30E+04					3	F3:2764	294	315	C9:Cysteine oxidation to cysteic acid:0.00
2	3	P05160	R.HGEIVHIE.C	Y	53,88	932,4716	8	0,6	467,2433	2	27,3	0,00E+00	2,52E+06	8,48E+05							22	F22:2302	294	301	
2	3	P05160	R.HGEIVHIECELNFEIHGSAEIR.C	Y	45,4	2531,2227	22	6,1	633,8168	4	52,7								4,66E+04	0,00E+00	73	F73:3346	294	315	
2	3	P05160	R.HGEIVHIECELNFEIHGSAEIR(+15.99)CEDGK.W	Y	36,81	3079,4126	27	5,4	616,8931	5	51,6				9,49E+04						30	F30:3267	294	320	R22:Oxidation or Hydroxylation:5.77
2	3	P05160	R.HGEIVHIECELN(+15.99)FEIHGSAEIRCEDGK.W	Y	36,66	3079,4126	27	6,3	616,8937	5	52,2				5,78E+04						28	F28:3300	294	320	N12:Oxidation or Hydroxylation:4.78
2	3	P05160	L.NFEIHGSAEIR.C	Y	48,6	1271,6259	11	5,4	636,8236	2	67,7			1,98E+04		1,39E+04	2,07E+04		2,93E+04	1,71E+05	74	F74:4260	305	315	
2	3	P05160	N.FEIHGSAEIR.C	Y	43,52	1157,5829	10	6,5	579,8025	2	52			1,99E+04			8,55E+04		2,68E+04	7,76E+04	73	F73:3304	306	315	
2	3	P05160	F.EIHGSAEIR.C	Y	44,62	1010,5145	9	3,7	506,2664	2	27							2,82E+04	0,00E+00		57	F57:1479	307	315	
2	3	P05160	E.IHGSAEIR.C	Y	45,16	881,4719	8	2,9	441,7445	2	26,2	2,67E+05		4,21E+04			9,12E+03		8,05E+04	2,86E+04	1	F1:2099	308	315	
2	3	P05160	I.HGSAEIR.C	Y	53,52	768,3878	7	4,3	385,2029	2	43,8	1,81E+05	2,29E+05	6,21E+05	2,55E+05	9,27E+05	1,46E+06	1,12E+05	1,50E+06	6,77E+05	73	F73:2798	309	315	
2	3	P05160	R.CEDGKWTEPPK.C	Y	58	1288,5758	11	7,6	645,3	2	32,6				0,00E+00		4,29E+05				51	F51:1863	316	326	
2	3	P05160	E.DGKWTEPPK.C	Y	47,51	1056,5239	9	3,2	529,2709	2	27,6			1,04E+06							19	F19:2289	318	326	
2	3	P05160	K.WTEPPK.C	Y	49,7	756,3806	6	2,2	379,1984	2	27,5	5,55E+07	8,50E+07	1,67E+07							1	F1:2213	321	326	
2	3	P05160	K.W(+31.99)TEPPK.C	Y	48,68	788,3704	6	6,9	395,1952	2	25,4	2,34E+06	5,93E+06								12	F12:2033	321	326	W1:Dihydroxy:57.45
2	3	P05160	K.W(+15.99)TEPPK.C	Y	45,88	772,3755	6	6,3	387,1975	2	25,5	4,19E+06	4,97E+06								12	F12:2040	321	326	W1:Oxidation (HW):0.00
2	3	P05160	K.W(+43.01)T(-2.02)EPP(-30.01)KCIEGQEK.V	Y	35,18	1554,7136	13	11	778,3723	2	29							7,12E+05			57	F57:1619	321	333	W1:Carbamylation:4.75;T2:2-amino-3-oxo-butanoic_acid:88.25;P5:Proline oxidation to pyrrolidinone:8.14
2	3	P05160	P.PKC(+15.99)IEGQEK(+42.01)VACEEPPFIENGAANLHSK.I	Y	38,01	3095,4692	28	1,9	1032,8323	3	35,3				1,34E+06						29	F29:2138	325	352	C3:Oxidation or Hydroxylation (C):0.71;K9:Acetylatio

																										n (K):0.00
2	3	P05160	K.CIEGQEKVAC(+15.99)EEPPFIENGAANLHSK.I	Y	67,53	2828,3108	26	-1,6	943,7761	3	30,2	8,07E+06	4,63E+06	3,21E+06	4,35E+06	3,04E+06		4,80E+06	0,00E+00		5	F5:2651	327	352		C10:Oxidation or Hydroxylation (C):3.75
2	3	P05160	K.C(+15.99)IEGQEKVACEEPPFIENGAANLHSK.I	Y	60,46	2828,3108	26	7,3	943,7844	3	32,6	5,58E+06		2,94E+06	6,83E+06	5,43E+06		2,11E+06	1,15E+06		38	F38:1899	327	352		C1:Oxidation or Hydroxylation (C):0.00
2	3	P05160	K.CIEGQEK(+15.99)VACEEPPFIENGAANLHSK.I	Y	60,17	2828,3108	26	7	943,7841	3	32,5	6,84E+06	5,14E+06		1,81E+06	1,60E+06		8,71E+05			62	F62:1857	327	352		K7:Oxidation or Hydroxylation:28.13
2	3	P05160	G.QEK(+43.01)VAC(-33.99)EEPPFIENGAANLHSK.I	Y	43,58	2419,1768	22	-8,3	1210,5856	2	33,1		1,15E+06	4,55E+05							17	F17:1893	331	352		K3:Carbamylation:11.10;C6:Dehydroalanine (C):0.00
2	3	P05160	K.VAC(-33.99)EEPPFIENGAANLHS(+79.96)K.I	Y	65,13	2070,9316	19	6,9	1036,4802	2	31,7	1,27E+06	0,00E+00								3	F3:2594	334	352	Sulfation	C3:Dehydroalanine (C):0.00;S18:Sulfation:1000.00
2	3	P05160	E.PPFIENGAANLHSK.I	Y	76,38	1493,7626	14	8	747,8945	2	34,2	1,05E+07	9,80E+06	1,36E+07	5,68E+07	5,07E+07	6,38E+07	5,29E+07	4,92E+07	3,79E+07	52	F52:2019	339	352		
2	3	P05160	P.PFIENGAANLHSK.I	Y	74,3	1396,7098	13	8,2	699,3679	2	34,2			6,93E+05	6,94E+06	1,16E+07	1,67E+07	6,31E+06	1,03E+07	1,08E+07	77	F77:2031	340	352		
2	3	P05160	I.ENGAANLHSK.I	Y	48,27	1039,5046	10	6	520,7627	2	34,3				7,60E+05	3,17E+06	2,89E+06	1,49E+06		1,04E+06	77	F77:2044	343	352		
2	3	P05160	E.NGAANLHSK.I	Y	61,48	910,462	9	3,1	456,2397	2	22,7	3,32E+06	4,62E+06	1,90E+06							19	F19:1818	344	352		
2	3	P05160	E.N(+.98)GAANLHSK.I	Y	56,06	911,446	9	4,3	456,7323	2	22,4	2,48E+05	6,82E+05	2,85E+05							19	F19:1789	344	352		N1:Deamidation (NQ):59.20
2	3	P05160	S.KIYYNGDK.V	Y	39,68	999,5025	8	3,2	1000,513	1	19,3	0,00E+00									4	F4:1592	352	359		
2	3	P05160	K.IYYNGDK.V	Y	66,21	871,4075	7	1,1	872,4158	1	22,3	2,05E+08	2,51E+08	5,95E+07	1,38E+06			1,17E+06			24	F24:1830	353	359		
2	3	P05160	K.IYYN(+.98)GDK.V	Y	55,95	872,3915	7	7,6	873,4055	1	24,9	1,64E+07	1,75E+07	2,44E+06				8,45E+04			2	F2:2006	353	359	Deamidation (NQ)	N4:Deamidation (NQ):1000.00
2	3	P05160	K.IYYNGDKVTY.A	Y	54,45	1234,5869	10	-4,2	618,2982	2	29,8	0,00E+00	0,00E+00								15	F15:2590	353	362		
2	3	P05160	K.IY(+15.99)YNGDK.V	Y	52,53	887,4025	7	-4	888,4062	1	23,9	4,09E+06	2,69E+06								13	F13:1965	353	359		Y2:Oxidation or Hydroxylation:22.99
2	3	P05160	K.IYY(+15.99)NGDK.V	Y	50,44	887,4025	7	0,5	888,4102	1	23,5	7,41E+05	6,27E+05								15	F15:1964	353	359		Y3:Oxidation or Hydroxylation:0.00
2	3	P05160	K.IY(+31.99)YNGDK.V	Y	45,16	903,3974	7	-5,8	904,3994	1	23,3	2,55E+05	1,07E+06								15	F15:1942	353	359		Y2:Dihydroxy:0.00
2	3	P05160	I.YYNGDK.V	Y	44,36	758,3235	6	0,1	759,3309	1	23,3	3,69E+05	1,07E+06	1,30E+06							24	F24:1926	354	359		
2	3	P05160	K.VTYACK.S	Y	48,84	731,316	6	6,2	732,3278	1	18,2	1,27E+06	1,48E+06								12	F12:1440	360	365		
2	3	P05160	K.SGYLLHGSNEITC(+47.98)NR.G	Y	100,8	1710,7632	15	10	856,3977	2	29,2	2,54E+06	3,48E+06			0,00E+00					2	F2:2375	366	380		C13:Cysteine oxidation to cysteic

																										acid:0.00
2	3	P05160	K.SGYLLHGSNEITCNR.G	Y	88,38	1662,7784	15	7,9	832,4031	2	32,8						1,06E+06	0,00E+00	7,68E+05	9,49E+05	65	F65:1920	366	380		
2	3	P05160	K.SGYLLHGSNEIT(-2.02)C(+31.99)NR.G	Y	69,36	1692,7526	15	8,5	847,3907	2	29,9	2,28E+06	2,37E+06								11	F11:2420	366	380		T12:2-amino-3-oxo-butanoic_acid:97.69;C13:Dihydroxy:11.10
2	3	P05160	K.SGYLLHGSNEIT(-2.02)C(+15.99)NR.G	Y	69,04	1676,7577	15	9,1	839,3937	2	30,6	1,97E+06	1,98E+06								2	F2:2500	366	380	Oxidation or Hydroxylation (C)	T12:2-amino-3-oxo-butanoic_acid:28.70;C13:Oxidation or Hydroxylation (C):100.00
2	3	P05160	K.SGYLLHGSNEITCNRGKWTLP.P	Y	63,94	2344,127	21	-1	1173,0696	2	31,8	2,68E+07	2,53E+07	0,00E+00	6,99E+06	1,12E+07		3,73E+06	4,96E+06		70	F70:1821	366	386		
2	3	P05160	K.SGYLLHGSN.E	Y	62,8	946,4508	9	0,6	474,2329	2	27,3	1,72E+07	1,25E+07	8,44E+06							24	F24:2320	366	374		
2	3	P05160	K.SGYLLHGSNEITCNRGK(+43.01)W(+13.98)T.L	Y	59,44	2192,0068	19	-6,5	1097,0035	2	34,2	3,98E+06	6,55E+06	1,24E+07	3,02E+07	2,94E+07	4,82E+07	3,30E+07	4,40E+07	3,26E+07	81	F81:2020	366	384	Tryptophan oxidation to oxolactone	K17:Carbamylation:167.58;W18:Tryptophan oxidation to oxolactone:1000.00
2	3	P05160	K.SGYLLHGSNEITCNRGK(+43.99)W(+44.99)T.L	Y	54,54	2223,9968	19	8,1	1113,0146	2	30,2	4,55E+06	8,19E+06		3,61E+06	1,64E+06		2,07E+06	5,16E+05		18	F18:1676	366	384	Carboxylation (DKW)	K17:Carboxylation (DKW):1000.00;W18: Oxidation to nitro:110.25
2	3	P05160	K.SGYLLHGSNE.I	Y	53,71	1075,4934	10	0,2	1076,5009	1	29,6	6,71E+07	7,46E+07	2,19E+07							19	F19:2499	366	375		
2	3	P05160	K.SGYLLHGSNEITCNR(-43.05)GKW(+19.99).T	Y	50,47	2010,9105	18	6,1	1006,4686	2	29,9	5,00E+05		0,00E+00	3,08E+06	7,89E+05					8	F8:1648	366	383	Tryptophan oxidation to hydroxykynurenin	R15:Arginine oxidation to glutamic semialdehyde:86.00;W18:Tryptophan oxidation to hydroxykynurenin:1000.00
2	3	P05160	K.SGYLLHGSNEITCNRGK(+43.99)W(+31.99)T.L	Y	50,11	2211,0015	19	-4	1106,5035	2	30,4		1,85E+06								18	F18:1696	366	384		K17:Carboxylation (DKW):0.00;W18:Dihydroxy:0.00
2	3	P05160	K.SGYLLHGSNEITC(+47.98)NR(+31.99)GKW(+15.99)TL.P	Y	47,06	2344,0754	20	6,4	1173,0525	2	29		0,00E+00								14	F14:2483	366	385	Cysteine oxidation to cysteic acid	C13:Cysteine oxidation to cysteic acid:1000.00;R15:Dihydroxy:8.22;W18:Oxi

																										dation (HW):77.56
2	3	P05160	K.SGYLLHGSNEITCNRGK(+31.99)W(+43.99)T.L	Y	41,46	2211,0015	19	-4	1106,5035	2	30,4	0,00E+00	2,24E+06		1,16E+06						16	F16:1704	366	384		K17:Dihydroxy:0.00; W18:Carboxylation (DKW):0.00
2	3	P05160	K.SGYLLHGSNEITCNR(+31.99)GKW(+43.99)T.L	Y	39,55	2211,0015	19	-2,5	1106,5052	2	30,2	1,73E+06									7	F7:1722	366	384		R15:Dihydroxy:0.00; W18:Carboxylation (DKW):0.00
2	3	P05160	K.SGYLLH.G	Y	37,57	688,3544	6	11	689,3691	1	28,1	1,09E+06									2	F2:2283	366	371		
2	3	P05160	K.SGYLLHGSNEITCN(+.98)RGK.W	Y	36,37	1848,8788	17	7,6	617,3049	3	30,9	9,17E+05									7	F7:1779	366	382		N14:Deamidation (NQ):23.70
2	3	P05160	K.WTLPPEC(-33.99)VENNENC(+31.99)K.H	Y	41,73	1772,7676	15	0,2	887,3912	2	34,9									5,86E+05	78	F78:2026	383	397		C7:Dehydroalanine (C):1.11;C14:Dihydroxy:0.00
2	3	P05160	K.WTLPPEC(+31.99)VENNENC(-33.99)K.H	Y	40,59	1772,7676	15	-4,8	887,3868	2	32,3	1,05E+07			6,82E+06	5,39E+06	7,76E+05	6,77E+07	4,11E+07	3,49E+06	6	F6:2848	383	397		C7:Dihydroxy:8.82;C14:Dehydroalanine (C):8.82
2	3	P05160	K.WTLPPECVENNENCK(-2.02).H	Y	40,24	1772,7498	15	6,6	887,388	2	34,7	2,35E+07		3,42E+06	2,08E+07	6,27E+07		4,45E+07	5,29E+07		70	F70:2066	383	397		K15:2-amino-3-oxo-butanoic_acid:60.73
2	3	P05160	K.WTLPPECVENN(+15.99)ENCK(-1.03)HPPVVMNGAVADGILASYATGSSVEYR.C	Y	35,18	4532,0723	42	5,3	1134,0314	4	38,3					8,53E+00					40	F40:2365	383	424	Lysine oxidation to aminoadipic semialdehyde	N11:Oxidation or Hydroxylation:0.00;K15:Lysine oxidation to aminoadipic semialdehyde:1000.00
2	3	P05160	K.HPPVVMNGAVADGILASYATGSSVEYR.C	Y	113,1	2760,354	27	9,9	1381,198	2	37,7	4,74E+08	5,72E+08	3,78E+08	5,61E+06	8,07E+06	8,24E+06	0,00E+00	1,16E+06	8,62E+05	39	F39:2352	398	424		
2	3	P05160	K.HP(+15.99)P(+31.99)VVM(-48.00)NGAVADGILASYATGSSVEYR.C	Y	107,3	2760,3354	27	2,2	1381,178	2	35,3	0,00E+00	0,00E+00								13	F13:3041	398	424	Dethiomethyl	P2:Oxidation or Hydroxylation:0.00;P3:Dihydroxy:0.00;M6:Dethiomethyl:1000.00
2	3	P05160	K.HPPVVM(+15.99)NGAVADGILASYATGSSVEYR.C	Y	95,21	2776,3489	27	11	1389,1965	2	36,5	6,25E+07	3,34E+07	2,91E+07	6,60E+08	2,35E+08	4,56E+08	9,09E+08	7,02E+08	5,41E+08	59	F59:2213	398	424	Oxidation (M)	M6:Oxidation (M):1000.00
2	3	P05160	K.HPPVVMN(+15.99)GAVADGILASYATGSSVEYR.C	Y	91,96	2776,3489	27	6,6	926,463	3	35,8	4,42E+07	5,04E+07	9,03E+06	2,63E+08	3,25E+08	3,55E+08	2,69E+08	8,07E+06	1,70E+08	81	F81:2152	398	424		N7:Oxidation or Hydroxylation:1.86
2	3	P05160	K.HPP(+15.99)VVMNGAVADGILASYATGSSVEYR.C	Y	88,75	2776,3489	27	10	1389,1959	2	36,5	1,14E+07	3,50E+05		1,23E+06	1,16E+08	5,29E+06	9,94E+05	7,72E+05	1,41E+06	42	F42:2211	398	424		P3:Oxidation or Hydroxylation:0.00
2	3	P05160	K.HPPVVMN(+.98)GAVADGILASYATGSSVEYR.C	Y	88,57	2761,3381	27	9,8	1381,6899	2	37,8	1,14E+07	4,43E+07	2,91E+07					0,00E+00		7	F7:2372	398	424	Deamidation (NQ)	N7:Deamidation (NQ):1000.

																										00
2	3	P05160	K.HPPVVMNGAVADGILASYATGSSVE.Y	Y	87,04	2441,1897	25	5,7	1221,6091	2	37,7	1,16E+07	8,06E+06								1	F1:3170	398	422		
2	3	P05160	K.HPPVVM(+31.99)NGAVADGILASYATGSSVEYR.C	Y	81,96	2792,344	27	9,7	931,7977	3	33,4	2,89E+06	4,17E+06								2	F2:2731	398	424	Dioxidation (M)	M6:Dioxidation (M):1000.00
2	3	P05160	K.HPPVVMNGAVADGILASYATGSSVEYR.C	Y	81,59	2776,3489	27	9,1	1389,1943	2	36,3	8,86E+04	6,84E+06		9,50E+05	1,29E+06	5,40E+06	3,65E+06	5,54E+07	1,53E+06	66	F66:2220	398	424		
2	3	P05160	K.HP(+15.99)PVVMNGAVADGILASYATGSSVEYR.C	Y	77,52	2776,3489	27	4	926,4606	3	44,8	6,20E+04	9,21E+05	3,84E+06	5,86E+05	1,71E+06	4,52E+05	4,37E+05	1,19E+06	2,87E+06	62	F62:2820	398	424		P2:Oxidation or Hydroxylation:0.00
2	3	P05160	K.HPPVVMN(+.98)GAVADGILASYATGSSVE.Y	Y	76,99	2442,1736	25	6,4	1222,1019	2	38	3,34E+05	5,96E+06								1	F1:3198	398	422	Deamidation (NQ)	N7:Deamidation (NQ):1000.00
2	3	P05160	K.HPPVVM(+15.99)NGAVADGILASYATGSSVE.Y	Y	76,3	2457,1846	25	3,4	1229,6038	2	36,3	3,99E+06									1	F1:3045	398	422	Oxidation (M)	M6:Oxidation (M):1000.00
2	3	P05160	K.H(+43.01)P(-30.01)P(-30.01)VVMNGAVADGILASYATGSSVEYR.C	Y	75,92	2743,3386	27	6,7	1372,6858	2	37,9	1,63E+07	6,30E+06	5,01E+06							7	F7:2378	398	424	Proline oxidation to pyrrolidinone	H1:Carbamylation:54.02;P2:Proline oxidation to pyrrolidinone:1000.00;P3:Proline oxidation to pyrrolidinone:1000.00
2	3	P05160	K.HPPVVM(+15.99)N(+.98)GAVADGILASYATGSSVEYR.C	Y	74,82	2777,333	27	8,4	926,7927	3	37,7	6,08E+06	3,66E+06		7,54E+07	9,27E+07	2,35E+07	7,40E+07	5,31E+07	4,27E+07	45	F45:2091	398	424	Oxidation (M); Deamidation (NQ)	M6:Oxidation (M):1000.00;N7:Deamidation (NQ):1000.00
2	3	P05160	K.HPPVVMNGAVADGILASY.A	Y	73,83	1809,9083	18	7,5	905,9683	2	37,3	1,16E+07	1,39E+07	2,07E+07							9	F9:2270	398	415		
2	3	P05160	K.HPPVVMNGAVADGILASYATGS.S	Y	72,58	2126,0466	22	6,6	1064,0376	2	37,7	0,00E+00	1,05E+06	0,00E+00							7	F7:2366	398	419		
2	3	P05160	K.HPPVVMNGAVAD(+15.99)GILASYATGSSVEYR.C	Y	72,24	2776,3489	27	2,4	926,4591	3	33,7	4,58E+06	6,79E+06		2,39E+06	1,72E+06	1,90E+06	1,61E+06	1,97E+06	2,87E+06	15	F15:2956	398	424		D12:Oxidation or Hydroxylation:0.00
2	3	P05160	K.HPPVVM(+15.99)NGAVADGILASYATGS.S	Y	71,11	2142,0415	22	7,6	1072,0361	2	36,3				5,88E+06	2,58E+06	4,30E+06	7,84E+06	6,19E+06	3,27E+06	30	F30:2219	398	419	Oxidation (M)	M6:Oxidation (M):1000.00
2	3	P05160	K.HPPVVMN(+.98)GAVADGILASYATG.S	Y	71,08	2039,9985	21	5,8	1021,0125	2	37,7	1,80E+06									1	F1:3171	398	418	Deamidation (NQ)	N7:Deamidation (NQ):1000.00
2	3	P05160	K.HPP(+15.99)VVMN(+.98)GAVADGILASYATGSSVEYR.C	Y	69,78	2777,333	27	9,3	926,7935	3	37,4				5,20E+06		4,15E+06	8,80E+03	2,57E+07	4,95E+06	80	F80:2303	398	424	Deamidation (NQ)	P3:Oxidation or Hydroxylation:0.00;N7:Deamidation (NQ):1000.00
2	3	P05160	K.H(+27.99)PP(-30.01)VVMN(+.98)GAVADGILASYATGSSVEYR.C	Y	69,75	2759,3225	27	8,7	1380,6805	2	37,1	0,00E+00			0,00E+00	2,43E+06	0,00E+00		2,76E+06	0,00E+00	39	F39:2299	398	424	Deamidation (NQ)	H1:Formylation:68.05;P3:Proline oxidation

																										to pyrrolidinone:0.00;N7:Deamidation (NQ):1000.00
2	3	P05160	K.HP(+31.99)P(+31.99)VVM(-48.00)NGAVADGILASYATGSSVEYR.C	Y	69,31	2776,3303	27	5	926,4554	3	57				0,00E+00	3,20E+05	3,10E+05	7,13E+05	6,61E+04	2,78E+05	52	F52:3575	398	424	Dethiomethyl	P2:Dihydroxy:15.89;P3:Dihydroxy:16.63;M6:Dethiomethyl:1000.00
2	3	P05160	K.HPPVVMNGAVADGILASYA.T	Y	68,91	1880,9454	19	4,3	941,4841	2	37,7	2,23E+07	1,34E+07	8,95E+06							1	F1:3169	398	416		
2	3	P05160	K.HPPVVM(+15.99)NGAVADGILASY.A	Y	68,04	1825,9032	18	8,1	913,9663	2	36,1				3,57E+07	1,01E+07	1,22E+07	3,54E+07	1,81E+07	5,73E+06	62	F62:2162	398	415	Oxidation (M)	M6:Oxidation (M):1000.00
2	3	P05160	K.HPPVVMNGAVADGILASYATG.S	Y	66,81	2039,0145	21	4,1	1020,5187	2	37,5	2,84E+06									1	F1:3150	398	418		
2	3	P05160	K.HP(+15.99)PVVMN(+.98)GAVADGILASYATGSSVEYR.C	Y	66,64	2777,333	27	6,9	926,7913	3	37				1,41E+07				1,60E+07		29	F29:2281	398	424	Deamidation (NQ)	P2:Oxidation or Hydroxylation:0.00;N7:Deamidation (NQ):1000.00
2	3	P05160	K.HPPVVMN(+.98)GAVAD(+15.99)GILASYATGSSVEYR.C	Y	66,17	2777,333	27	9,1	926,7934	3	37,4	0,00E+00				8,62E+06	2,41E+06	1,61E+03		7,52E+06	43	F43:2293	398	424	Deamidation (NQ)	N7:Deamidation (NQ):1000.00;D12:Oxidation or Hydroxylation:1.49
2	3	P05160	K.HPP(+31.99)VVM(-48.00)N(+15.99)GAVADGILASYATGSSVEYR.C	Y	64,63	2760,3354	27	1,5	1381,177	2	36,9	2,42E+04			0,00E+00						36	F36:2267	398	424	Dethiomethyl	P3:Dihydroxy:0.00;M6:Dethiomethyl:1000.00;N7:Oxidation or Hydroxylation:0.00
2	3	P05160	K.HPP(-27.99)VVMN(+.98)GAVAD(+43.99)GILASYATGSSVEYR.C	Y	63,48	2777,333	27	9,2	926,7935	3	36,9				2,34E+07				1,26E+06		30	F30:2268	398	424	Deamidation (NQ); Carboxylation (DKW)	P3:Pyrrolidone from Proline:0.00;N7:Deamidation (NQ):1000.00;D12:Carboxylation (DKW):1000.00
2	3	P05160	K.HP(+13.98)P(+13.98)VVMNGAVADGILASYATGSSVEYR.C	Y	62,5	2816,3076	27	4,2	939,7804	3	66,3	5,42E+04	3,14E+04								8	F8:4092	398	424	Proline oxidation to pyroglutamic acid	P2:Proline oxidation to pyroglutamic acid:1000.00;P3:Proline oxidation to pyroglutamic acid:1000.00
2	3	P05160	K.HP(-30.01)PVVMN(+.98)GAVADGILASYATGSSVEYR.C	Y	62,46	2759,3225	27	8,3	920,7891	3	33,8		4,38E+05		3,20E+06	0,00E+00		0,00E+00		1,39E+05	11	F11:2746	398	424	Deamidation (NQ)	P2:Proline oxidation to

																										pyrrolidinone:0.00;N7:Deamidation (NQ):1000.00
2	3	P05160	K.HPPVVM(+15.99)N(+.98)GAVADGILASYATGSSVE.Y	Y	60,51	2458,1685	25	5,7	1230,0985	2	36,6	1,29E+06	2,03E+06								10	F10:3068	398	422	Oxidation (M); Deamidation (NQ)	M6:Oxidation (M):1000.00;N7:Deamidation (NQ):1000.00
2	3	P05160	K.HP(+31.99)PVVM(-48.00)NGAVAD(+15.99)GILASYATGSSVEYR.C	Y	60,47	2760,3354	27	7,3	921,1258	3	47,4		3,90E+05	2,63E+05	0,00E+00		0,00E+00				27	F27:3029	398	424	Dethiomethyl	P2:Dihydroxy:7.65;M6:Dethiomethyl:1000.00;D12:Oxidation or Hydroxylation:0.00
2	3	P05160	K.HPPVVMN(+.98)GAVAD.G	Y	60,16	1206,5703	12	3	1207,5812	1	31	5,65E+06	1,72E+07								10	F10:2549	398	409	Deamidation (NQ)	N7:Deamidation (NQ):1000.00
2	3	P05160	K.H(-22.03)P(+13.98)PVVMNGAVAD(+79.97)GILASYATGSSVEYR.C	Y	59,91	2832,2678	27	8,5	945,1046	3	66,7				1,45E+05	0,00E+00					29	F29:4139	398	424		H1:his2asp:0.00;P2:Proline oxidation to pyroglutamic acid:0.00;D12:Phosphorylation (HCDR):110.25
2	3	P05160	K.H(+15.99)PP(+31.99)VVM(-48.00)NGAVADGILASYATGSSVEYR.C	Y	59,68	2760,3354	27	5,5	921,1241	3	65,7		3,69E+05	0,00E+00							16	F16:4065	398	424	Dethiomethyl	H1:Oxidation (HW):100.00;P3:Dihydroxy:0.00;M6:Dethiomethyl:1000.00
2	3	P05160	K.HP(+31.99)P(+15.99)VVM(-48.00)NGAVADGILASYATGSSVEYR.C	Y	58	2760,3354	27	7,1	921,1256	3	55,1	0,00E+00	0,00E+00		0,00E+00	0,00E+00					7	F7:3570	398	424	Dethiomethyl	P2:Dihydroxy:0.00;P3:Oxidation or Hydroxylation:0.00;M6:Dethiomethyl:1000.00
2	3	P05160	K.HPPVVMNGAVAD.G	Y	57,98	1205,5863	12	1,3	1206,5951	1	30,7	2,09E+07	3,07E+07	2,36E+06							10	F10:2527	398	409		
2	3	P05160	K.HPPVVMN(+.98)GAVADGILASYA.T	Y	57,43	1881,9294	19	3,7	941,9755	2	37,9	1,47E+06									1	F1:3192	398	416	Deamidation (NQ)	N7:Deamidation (NQ):1000.00
2	3	P05160	K.H(+27.99)P(-30.01)PVVMNGAVADGILASYATGSSVEYR.C	Y	56,24	2758,3384	27	6,9	920,4597	3	36					7,29E+05			0,00E+00	0,00E+00	77	F77:2192	398	424	Formylation	H1:Formylation:1000.00;P2:Proline oxidation to pyrrolidinone:0.00
2	3	P05160	K.HPPVVMNGAVADGILAS.Y	Y	55,96	1646,845	17	7,7	824,4361	2	36	2,91E+06	1,82E+06								7	F7:2217	398	414		
2	3	P05160	K.HP(+13.98)P(+13.98)VVMNGAVAD(+43.99)GILASYATGSSVEYR.C	Y	55,02	2832,3025	27	2,8	945,1108	3	67,3				2,57E+04			0,00E+00			30	F30:4206	398	424	Proline oxidation to	P2:Proline oxidation to

																									pyroglutamic acid; Carboxylation (DKW)	pyroglutamic acid:1000.00;P3:Proline oxidation to pyroglutamic acid:1000.00;D12:Carboxylation (DKW):1000.00
2	3	P05160	K.HPPVVMNG.A	Y	52,98	849,4167	8	-1,5	850,4227	1	26,4	0,00E+00	0,00E+00	0,00E+00							5	F5:2286	398	405		
2	3	P05160	K.HPPVVMNGAVADGILASYAT.G	Y	52,66	1981,9932	20	-0,9	992,0029	2	35,3			0,00E+00							22	F22:3065	398	417		
2	3	P05160	K.H(+15.99)P(+31.99)PVVM(-48.00)NGAVADGILASYATGSSVEYR.C	Y	50,54	2760,3354	27	-7,4	1381,1648	2	36,9				0,00E+00						29	F29:2271	398	424	Oxidation (HW); Dethiomethyl	H1:Oxidation (HW):1000.00;P2:Dihydroxy:0.00;M6:Dethiomethyl:1000.00
2	3	P05160	K.HPPVVM(+15.99)NGAVADGIL.A	Y	50,41	1504,7708	15	7,7	753,3984	2	32,5	1,09E+06	7,63E+05	2,22E+06							12	F12:2647	398	412	Oxidation (M)	M6:Oxidation (M):1000.00
2	3	P05160	K.HPPVVM(+15.99)NGAVADGILAS.Y	Y	49,76	1662,84	17	5,6	832,4319	2	34,4	3,21E+06			4,97E+06			1,88E+06	2,36E+06		29	F29:2060	398	414	Oxidation (M)	M6:Oxidation (M):1000.00
2	3	P05160	K.H(-23.02)PPVVMNGAVADGILAS(+79.96)YATGSSVEYR.C	Y	49,72	2817,2949	27	3,7	940,1091	3	67,2	6,69E+04									7	F7:4279	398	424	his2asn	H1:his2asn:1000.00;S17:Sulfation:17.01
2	3	P05160	K.H(-22.03)P(+15.99)PVVMNGAVAD(+79.97)GILASYATGSSVEYR.C	Y	49,56	2834,2834	27	4,4	945,7726	3	67,4									3,10E+03	80	F80:4159	398	424	his2asp	H1:his2asp:1000.00;P2:Oxidation or Hydroxylation:0.00;D12:Phosphorylation (HCDR):83.43
2	3	P05160	K.HPPVVMN(+15.99)GAVADGILASYATGS.S	Y	49,37	2142,0415	22	6,9	1072,0354	2	36,5				7,91E+05						35	F35:2229	398	419		N7:Oxidation or Hydroxylation:25.91
2	3	P05160	K.HPP(+31.99)VVM(-48.00)NGAVAD(+15.99)GILASYATGSSVEYR.C	Y	49,24	2760,3354	27	3,8	921,1226	3	37,1			1,83E+05			0,00E+00				48	F48:2268	398	424	Dethiomethyl	P3:Dihydroxy:0.00;M6:Dethiomethyl:1000.00;D12:Oxidation or Hydroxylation:1.60
2	3	P05160	K.H(-23.02)PPVVM(+15.99)NGAVAD(+79.97)GILASYATGSSVEYR.C	Y	49,04	2833,2993	27	6,4	945,4464	3	65,9				0,00E+00						30	F30:4119	398	424	his2asn; Oxidation (M)	H1:his2asn:1000.00;M6:Oxidation (M):1000.00;D12:Phosphorylation (HCDR):82.83
2	3	P05160	K.HPPVVM(+15.99)NGAVADGILASYA.T	Y	48,37	1896,940	19	9,4	949,4864	2	36,						0,00E+00				46	F46:225	398	416	Oxidation (M)	M6:Oxidation

						3					9									5				(M):1000.0 0
2	3	P051 60	K.HP(+13.98)P(+15.99)VVM(-48.00)NGAVADGILASYATGSSVEYR.C	Y	47, 75	2742 ,325	27	14	915, 1287	3	3 7		0,00E+00						18	F18: 224 6	398	424	Dethiomet hyl	P2:Proline oxidation to pyrogluta mic acid:0.00;P 3:Oxidatio n or Hydroxylat ion:0.00;M 6:Dethiom ethyl:1000 .00
2	3	P051 60	K.HPPVVMN.G	Y	47, 23	792, 3953	7	2, 5	793, 4045	1	2 8, 9	3,74E+06	1,04E+07	9,19E+ 06					1	F1:2 342	398	404		
2	3	P051 60	K.H(-22.03)PP(+13.98)VVMNGAVAD(+79.97)GILASYATGSSVEYR.C	Y	47, 22	2832 ,267 8	27	9, 3	945, 1053	3	6 6, 9					0,00E+0 0		2,41E+0 4	63	F63: 416 7	398	424	his2asp	H1:his2asp :1000.00;P 3:Proline oxidation to pyrogluta mic acid:0.00; D12:Phosp horylation (HCDR):83. 43
2	3	P051 60	K.HPPVVM(+15.99)NGAVAD.G	Y	46, 58	1221 ,581 2	12	2, 8	1222 ,591 9	1	2 8, 5	3,95E+06	8,83E+06						10	F10: 230 8	398	409	Oxidation (M)	M6:Oxidat ion (M):1000.0 0
2	3	P051 60	K.H(+15.99)PP(+13.98)VVM(-48.00)NGAVADGILASYATGSSVEYR.C	Y	45, 46	2742 ,325	27	14	1372 ,189 3	2	3 7, 1		9,47E+05						17	F17: 222 7	398	424	Dethiomet hyl	H1:Oxidati on (HW):0.00; P3:Proline oxidation to pyrogluta mic acid:0.00; M6:Dethio methyl:10 00.00
2	3	P051 60	K.H(-23.02)PP(+15.99)VVMNGAVAD(+79.97)GILASYATGSSVEYR.C	Y	45, 16	2833 ,299 3	27	- 3, 1	945, 4374	3	6 5, 9				0,00E+0 0			0,00E+0 0	55	F55: 413 6	398	424	his2asn	H1:his2asn :1000.00;P 3:Oxidatio n or Hydroxylat ion:0.00;D 12:Phosph orylation (HCDR):12 4.59
2	3	P051 60	K.HP(+31.99)PVVM(-48.00)NGAVADGILASY(+31.99)ATGSSVEYR.C	Y	44, 17	2776 ,330 3	27	7, 2	926, 4574	3	5 4, 1				0,00E+0 0		3,92E+0 4		51	F51: 338 2	398	424	Dethiomet hyl	P2:Dihydr oxy:14.02; M6:Dethio methyl:10 00.00;Y18: Dihydroxy: 1.05
2	3	P051 60	K.HPPVVM(+15.99)N.G	Y	43, 93	808, 3901	7	5, 9	405, 2047	2	2 3, 4	8,28E+05							2	F2:1 877	398	404	Oxidation (M)	M6:Oxidat ion (M):1000.0 0
2	3	P051 60	K.HPPVVM(+15.99)N(+.98)GAVAD.G	Y	42, 76	1222 ,565 2	12	2, 5	612, 2914	2	2 9	1,12E+06							1	F1:2 358	398	409	Oxidation (M); Deamidati on (NQ)	M6:Oxidat ion (M):1000.0 0;N7:Dea midation (NQ):1000. 00
2	3	P051	K.HPPVVM.N	Y	42,	678,	6	-	679,	1	2	1,49E+07	9,90E+06	8,97E+					15	F15:	398	403		

		60			45	3523		2,2	3581		7,1			06								2322				
2	3	P05160	K.HPPVVMNGAVA.D	Y	42,43	1090,5593	11	2	546,288	2	31,4	3,97E+06	1,98E+06								1	F1:2587	398	408		
2	3	P05160	K.HPPVVMNGA.V	Y	42,32	920,4538	9	-9	921,4528	1	27,6	0,00E+00									4	F4:2395	398	406		
2	3	P05160	K.H(+43.01)P(+13.98)PVVMN(+15.99)GAVADGILASYATGSSVEYR.C	Y	41,52	2833,334	27	8,7	945,4601	3	66,3							0,00E+00			60	F60:4107	398	424	Carbamylation	H1:Carbamylation:1000.00;P2:Proline oxidation to pyroglutamic acid:0.00;N7:Oxidation or Hydroxylation:2.44
2	3	P05160	K.HPPVVMNGAVADGILA.S	Y	41,06	1559,813	16	1,5	780,915	2	36,1	9,49E+05									1	F1:3027	398	413		
2	3	P05160	K.HPPVVMNGAVADGILASY(+15.99)ATGSSVEYR.C	Y	40,96	2776,3489	27	7,1	926,4634	3	55				0,00E+00			3,82E+04			28	F28:3470	398	424		Y18:Oxidation or Hydroxylation:4.12
2	3	P05160	K.HPPVVMN(+.98).G	Y	40,68	793,3793	7	3,1	794,389	1	29,6	2,70E+06	6,04E+06								1	F1:2419	398	404	Deamidation (NQ)	N7:Deamidation (NQ):1000.00
2	3	P05160	K.H(-23.02)PP(+15.99)VVMNGAVADGILAS(+79.96)YATGSSVEYR.C	Y	40,57	2833,2898	27	3,5	945,4406	3	67,2				0,00E+00			0,00E+00			56	F56:4222	398	424	his2asn	H1:his2asn:1000.00;P3:Oxidation or Hydroxylation:0.00;S17:Sulfation:17.01
2	3	P05160	K.HPPVVM(+15.99)N(+.98)GAVADGILASYA.T	Y	40,39	1897,9243	19	4,7	949,9739	2	36,8	3,03E+05									1	F1:3095	398	416	Oxidation (M); Deamidation (NQ)	M6:Oxidation (M):1000.00;N7:Deamidation (NQ):1000.00
2	3	P05160	K.H(-23.02)PPVVMNGAVAD(+79.97)GILASYATGSSVEYR.C	Y	38,83	2817,3044	27	-4,9	940,1041	3	65,8	6,69E+04									7	F7:4191	398	424	his2asn	H1:his2asn:1000.00;D12:Phosphorylation (HCDR):88.89
2	3	P05160	K.HPP(-27.99)VVMNGAVADGILASY(+44.99)ATGSSVEYR.C	Y	37,59	2777,3442	27	6,9	926,7951	3	50,8						0,00E+00				54	F54:3238	398	424		P3:Pyrrolidone from Proline:0.00;Y18:Oxidation to nitro:73.16
2	3	P05160	K.HPPVVMNGAVADGIL.A	Y	37,49	1488,7759	15	-1,2	745,3943	2	34,3	0,00E+00		2,00E+06							5	F5:3026	398	412		
2	3	P05160	K.HPPVVM(+15.99)N(+.98)GAVADGILASY.A	Y	37,34	1826,8872	18	12	914,4621	2	37									1,10E+06	74	F74:2265	398	415	Oxidation (M); Deamidation (NQ)	M6:Oxidation (M):1000.00;N7:Deamidation (NQ):1000.00
2	3	P05160	K.HP(+31.99)PVVM(-48.00)NGAVADGILASY(+15.99)ATGSSVEYR.C	Y	36,57	2760,3354	27	8,7	921,1271	3	66,1		6,12E+04								16	F16:4088	398	424	Dethiomethyl	P2:Dihydroxy:0.00;M6:Dethiomethyl:1000.00;Y18:Oxidation

																										or Hydroxylation:9.45
2	3	P05160	K.HPPVVM(+15.99).N	Y	35,85	694,3472	6	7,2	695,3595	1	24,1	1,56E+06	1,66E+06								11	F11:1921	398	403	Oxidation (M)	M6:Oxidation (M):1000.00
2	3	P05160	K.HPPVVM(-48.00)N(+15.99)GAVADGILASY(+31.99)ATGSSVEYR.C	Y	35,63	2760,3354	27	7,4	921,1259	3	55			0,00E+00							26	F26:3479	398	424	Dethiomethyl	M6:Dethiomethyl:1000.00;N7:Oxidation or Hydroxylation:0.00;Y18:Dihydroxy:0.00
2	3	P05160	K.HP(+31.99)P(+31.99)VVM(-48.00)NGAVADGILASYATG.S	Y	35,26	2054,991	21	-0,5	1028,5022	2	35,9	0,00E+00									1	F1:3016	398	418	Dethiomethyl	P2:Dihydroxy:72.17; P3:Dihydroxy:75.35; M6:Dethiomethyl:1000.00
2	3	P05160	M.NGAVADGILASYATGSSVEYR.C	Y	103,1	2100,0122	21	6,4	1051,0201	2	36,8	2,48E+07	2,30E+07	1,22E+07							26	F26:2246	404	424		
2	3	P05160	N.GAVADGILASYATGSSVEYR.C	Y	100,6	1985,9694	20	4,8	993,9968	2	36,8	3,44E+07	2,69E+07	3,39E+07	4,44E+06	2,01E+06	5,95E+06	2,82E+06	4,62E+06	4,18E+06	26	F26:2245	405	424		
2	3	P05160	G.AVADGILASYATGSSVEYR.C	Y	57,91	1928,9479	19	1,1	965,4823	2	34			0,00E+00							23	F23:2982	406	424		
2	3	P05160	A.VADGILASYATGSSVEYR.C	Y	80,13	1857,9108	18	0,7	929,9633	2	33,3	1,70E+06	2,58E+06	6,34E+06							22	F22:2884	407	424		
2	3	P05160	A.DGILASYATGSSVEYR.C	Y	60,72	1687,8053	16	8,5	844,9171	2	35,6		3,41E+05	2,24E+06			6,27E+05				26	F26:2152	409	424		
2	3	P05160	D.GILASYATGSSVEYR.C	Y	74,51	1572,7783	15	2,3	787,3983	2	35			3,47E+05	1,39E+06	3,86E+06	5,44E+06	4,93E+06	2,32E+06	7,15E+06	75	F75:1927	410	424		
2	3	P05160	G.ILASYATGSSVEYR.C	Y	82,82	1515,7568	14	8,3	758,892	2	33,3	3,64E+06	0,00E+00	1,70E+06	1,54E+06	1,47E+05	0,00E+00	9,87E+06	0,00E+00	1,03E+07	79	F79:1952	411	424		
2	3	P05160	I.LASYATGSSVEYR.C	Y	63,01	1402,6729	13	5,8	702,3478	2	36,4					5,09E+06			8,90E+06	4,96E+05	65	F65:2230	412	424		
2	3	P05160	L.ASYATGSSVEYR.C	Y	68,83	1289,5887	12	-1,2	645,8008	2	27,2	9,67E+06	1,49E+07	5,15E+07	5,68E+06	4,20E+06	2,69E+06	6,53E+06	8,64E+06	1,71E+06	23	F23:2329	413	424		
2	3	P05160	A.SYATGSSVEYR.C	Y	69,61	1218,5516	11	0,4	610,2833	2	27	2,40E+06	2,91E+06	2,68E+06		7,12E+05	9,06E+05	1,31E+05	1,49E+06	2,86E+06	22	F22:2274	414	424		
2	3	P05160	S.YATGSSVEYR.C	Y	62,2	1131,5197	10	6,1	566,7706	2	25,8	3,27E+07	5,06E+07	3,36E+07	1,47E+06			0,00E+00		0,00E+00	11	F11:2071	415	424		
2	3	P05160	Y.ATGSSVEYR.C	Y	55,61	968,4563	9	0,7	485,2357	2	18,8	1,87E+07	2,23E+07	1,89E+07							6	F6:1554	416	424		
2	3	P05160	Y.ATGSSVEY(+15.99)R.C	Y	53,41	984,4512	9	2	493,2339	2	18,7	0,00E+00	1,76E+05								13	F13:1493	416	424		Y8:Oxidation or Hydroxylation:0.00
2	3	P05160	A.TGSSVEYR.C	Y	58,76	897,4192	8	-0,2	449,7168	2	16,6	7,61E+06	5,73E+06	7,44E+06							24	F24:1311	417	424		
2	3	P05160	T.GSSVEYR.C	Y	51,17	796,3715	7	-1,1	399,1926	2	14,5	1,02E+06	1,61E+06	2,36E+06							23	F23:1128	418	424		
2	3	P05160	G.S(+27.99)SVEYRCNEYYLLR.G	Y	36,42	1821,8356	14	2,5	911,9274	2	31,5	0,00E+00									4	F4:2769	419	432	Formylation	S1:Formylation:1000.00
2	3	P05160	K.WSSPPVC(-33.99)LEP(+31.99)CTVNVDYMNR.N	Y	80,32	2307,03	20	-1,	1154,520	2	36,	3,26E+08	2,20E+08	1,54E+08	9,12E+07	6,45E+07	2,52E+07	1,45E+08	2,39E+08	7,54E+07	19	F19:316	444	463		C7:Dehydroalanine

								9	1		4											8				(C):18.33;P10:Dihydroxy:0.00
2	3	P05160	K.WSSPPVC(-33.99)LEPC(+31.99)TVNVDYMNR.N	Y	78,27	2307,03	20	0,7	1154,5231	2	37,6	5,92E+08	1,65E+08	8,55E+07	4,78E+08	4,00E+08	1,80E+08	6,05E+08	2,66E+08	4,81E+07	62	F62:2289	444	463		C7:Dehydroalanine (C):23.44;C11:Dihydroxy:8.69
2	3	P05160	K.WSSPPVC(+31.99)LEPC(-33.99)TVNVDYMNR.N	Y	63,49	2307,03	20	-0,1	1154,5222	2	37,6		1,43E+08					1,06E+08			18	F18:2294	444	463		C7:Dihydroxy:0.00;C11:Dehydroalanine (C):0.00
2	3	P05160	K.WSSPPVCLEPCT(-2.02)VNVDYMNR.N	Y	62,85	2307,0122	20	7,1	1154,5216	2	37,7	1,30E+08	2,32E+08	1,01E+08	3,28E+06	3,52E+04		5,41E+07			59	F59:2320	444	463		T12:2-amino-3-oxo-butanoic_acid:35.88
2	3	P05160	K.WSSPPVCLEP(+13.98)C(+15.99)TVNVDYMNR.N	Y	62,55	2339,002	20	-0,1	1170,5082	2	38,5	0,00E+00	0,00E+00	3,00E+06		1,17E+06	5,34E+06		2,78E+06		50	F50:2355	444	463		P10:Proline oxidation to pyroglutamic acid:33.54;C11:Oxidation or Hydroxylation (C):14.19
2	3	P05160	K.WSSPPVCLEPCT(-2.02)VNVDYM(+15.99)NR.N	Y	61,54	2323,0071	20	6,5	1162,5183	2	36,7	2,29E+07	2,58E+06	1,64E+07	1,76E+08	4,14E+08	5,40E+07	3,09E+08	3,38E+08	2,39E+07	31	F31:2247	444	463	Oxidation (M)	T12:2-amino-3-oxo-butanoic_acid:27.44;M18:Oxidation (M):1000.00
2	3	P05160	K.WSSPPVCLEPCT(-2.02)VNVDYMN(+15.99)R.N	Y	60,62	2323,0071	20	5,2	1162,5168	2	37	3,32E+06	3,24E+07		1,61E+08	1,42E+08	4,56E+07	1,55E+08	1,74E+08	4,25E+07	51	F51:2233	444	463		T12:2-amino-3-oxo-butanoic_acid:44.14;N19:Oxidation or Hydroxylation:0.00
2	3	P05160	K.WSSPPVC(+15.99)LEP(+13.98)CTVNVDYMNR.N	Y	57,9	2339,002	20	0,7	1170,509	2	38,3		1,71E+06	2,06E+06		0,00E+00				1,74E+06	38	F38:2376	444	463		C7:Oxidation or Hydroxylation (C):3.56;P10:Proline oxidation to pyroglutamic acid:24.47
2	3	P05160	K.WSSPPVCLEPCT(-2.02)VNVDYMNR(+15.99).N	Y	57,24	2323,0071	20	6	1162,5178	2	36,6	9,94E+06		1,10E+06	2,11E+08		1,40E+07	1,16E+08	1,14E+05		29	F29:2245	444	463		T12:2-amino-3-oxo-butanoic_acid:44.14;R20:Oxidation or Hydroxylation:0.00
2	3	P05160	K.W(+15.99)SSPPVC(-33.99)LEP(+31.99)CTVNVDYMNR.N	Y	57,14	2323,0249	20	-1,5	1162,5179	2	37,5		1,58E+07	5,94E+06							16	F16:2294	444	463		W1:Oxidation (HW):0.00;C7:Dehydroalanine (C):26.57;P10:Dihydro

																									xy:8.14
2	3	P05160	K.WSS(-2.02)PPVCLEPCTVNVDYMNR.N	Y	54,73	2307,0122	20	4,5	770,0148	3	37,8				1,49E+06	1,19E+06		5,25E+06			38	F38:2340	444	463	S3:2-amino-3-oxo-butanoic_acid:11.06
2	3	P05160	K.W(+15.99)SSPPVCLEPCT(-2.02)VNVDYMNR.N	Y	54,33	2323,0071	20	0,5	1162,5114	2	36,8	1,57E+06						0,00E+00			1	F1:3094	444	463	W1:Oxidation (HW):0.00;T12:2-amino-3-oxo-butanoic_acid:31.37
2	3	P05160	K.WSSPPVCLEPC(+31.99)T(-2.02)VNVDYMNR.N	Y	53,3	2339,002	20	-0,7	1170,5074	2	38,4		1,06E+06				1,11E+06			1,65E+06	74	F74:2379	444	463	C11:Dihydroxy:3.56;T12:2-amino-3-oxo-butanoic_acid:39.35
2	3	P05160	K.W(+31.99)SSPPVC(-33.99)LEP(+31.99)CTVNVDYMNR.N	Y	52,23	2339,0198	20	-2,2	1170,5146	2	37,5		3,23E+06			1,03E+05					45	F45:2076	444	463	W1:Dihydroxy:5.08;C7:Dehydroalanine (C):31.27;P10:Dihydroxy:0.00
2	3	P05160	K.W(+43.99)SSPPVC(-33.99)LEPCTVNVDYMNR.N	Y	50,7	2319,03	20	-2,1	1160,5199	2	38,3			1,99E+06		2,09E+05		1,88E+06	4,52E+06		65	F65:2388	444	463	W1:Carboxylation (DKW):85.47;C7:Dehydroalanine (C):8.26
2	3	P05160	K.WSS(-2.02)PPVCLEPCTVNVDYMNR(+15.99).N	Y	48,33	2323,0071	20	4,9	775,3468	3	36,7							2,92E+06			56	F56:2266	444	463	S3:2-amino-3-oxo-butanoic_acid:0.00;R20:Oxidation or Hydroxylation:0.00
2	3	P05160	K.WSSPP(+15.99)VC(-33.99)LEP(+31.99)CTVNVDYMNR.N	Y	46,69	2323,0249	20	2,4	1162,5225	2	37,6			7,62E+06							26	F26:2314	444	463	P5:Oxidation or Hydroxylation:11.06;C7:Dehydroalanine (C):31.27;P10:Dihydroxy:0.00
2	3	P05160	K.WS(-2.02)SPPVCLEPCTVNVDYMNR.N	Y	45,77	2307,0122	20	5,9	770,0159	3	37,4	7,28E+06									7	F7:2335	444	463	S2:2-amino-3-oxo-butanoic_acid:0.00
2	3	P05160	K.W(+31.99)SSPPVCLEPCT(-2.02)VNVDYMNR.N	Y	44,33	2339,002	20	8,2	1170,5178	2	36,7	1,74E+06									7	F7:2277	444	463	W1:Dihydroxy:12.33;T12:2-amino-3-oxo-butanoic_acid:34.96
2	3	P05160	K.WSSPP(+15.99)VCLEPCT(-2.02)VNVDYMNR.N	Y	36,76	2323,0071	20	1,6	1162,5127	2	37,2							0,00E+00			56	F56:2309	444	463	P5:Oxidation or Hydroxylation:0.00;T12:2-amino-3-oxo-butanoic_acid:18.77
2	3	P05160	K.WSSPPVCLEP(+31.99)C(-33.99)TVNVDYMNR.N	Y	35,66	2307,03	20	8,2	1154,531	2	50,		0,00E+00								16	F16:316	444	463	P10:Dihydroxy:0.00;C

									7		4											8				11:Dehydr oalanine (C):0.00
2	3	P051 60	W.SSPPVC(- 33.99)LEP(+31.99)CTVNVDYMNR.N	Y	52, 32	2120 ,950 7	19	- 0, 4	1061 ,482 2	2	3 6, 3		7,86E+05								18	F18: 218 5	445	463		C6:Dehydr oalanine (C):19.86;P 9:Dihydrox y:0.00
2	3	P051 60	S.SPPVC(+31.99)LEPC(- 33.99)TVNVDYMNR.N	Y	63, 66	2033 ,918 7	18	- 3, 9	1017 ,962 7	2	3 5, 3	3,48E+06									1	F1:2 960	446	463		C5:Dihydro xy:0.00;C9: Dehydroal anine (C):0.00
2	3	P051 60	S.SPPVC(- 33.99)LEPC(+31.99)TVNVDYMNR.N	Y	58, 55	2033 ,918 7	18	- 3, 5	1017 ,963 1	2	3 5		0,00E+00								10	F10: 292 9	446	463		C5:Dehydr oalanine (C):52.34;C 9:Dihydrox y:8.69
2	3	P051 60	S.PPVC(- 33.99)LEPC(+31.99)TVNVDYMNR.N	Y	77, 88	1946 ,886 6	17	- 1, 3	974, 4493	2	3 7, 7	5,44E+06	1,32E+07	1,05E+ 07	3,95E+0 6	8,50E+0 6		1,96E+0 6	8,38E+0 6		55	F55: 234 5	447	463		C4:Dehydr oalanine (C):74.79;C 8:Dihydrox y:11.06
2	3	P051 60	S.PPVC(- 33.99)LEP(+31.99)CTVNVDYMNR.N	Y	58, 42	1946 ,886 6	17	- 5, 3	974, 4454	2	3 7, 9					0,00E+0 0					41	F41: 232 7	447	463		C4:Dehydr oalanine (C):58.99;P 7:Dihydrox y:0.00
2	3	P051 60	S.PPVCLEPCT(- 2.02)VNVDYM(+15.99)NR.N	Y	42, 03	1962 ,863 8	17	7	982, 446	2	3 6, 9								7,93E+0 5		65	F65: 227 0	447	463	Oxidation (M)	T9:2- amino-3- oxo- butanoic_ acid:119.5 4;M15:Oxi dation (M):1000.0 0
2	3	P051 60	R.NNIEMKW(+15.99)KYEGK.V	Y	68, 77	1554 ,75	12	8, 5	778, 3889	2	2 8, 9					6,30E+0 4	9,71E+0 4	4,24E+0 4	1,00E+0 5	8,78E+ 04	68	F68: 159 5	464	475	Oxidation (HW)	W7:Oxidat ion (HW):1000 .00
2	3	P051 60	R.NNIEMKW(+15.99)K.Y	Y	63, 61	1077 ,527 7	8	5, 1	539, 7739	2	3 0, 2	3,65E+05	1,25E+05	3,24E+ 05	1,54E+0 7	2,81E+0 6	5,10E+0 6	1,87E+0 7	2,84E+0 6	1,57E+ 07	80	F80: 169 8	464	471	Oxidation (HW)	W7:Oxidat ion (HW):1000 .00
2	3	P051 60	R.NNIEM(+15.99)KW(+31.99)KYEGK. V	Y	58, 9	1586 ,739 9	12	2, 4	794, 3792	2	2 7, 8				1,11E+0 6	1,98E+0 6	0,00E+0 0	1,70E+0 6	0,00E+0 0	5,69E+ 06	43	F43: 152 5	464	475	Oxidation (M)	M5:Oxidat ion (M):1000.0 0;W7:Dihy droxy:23.1 0
2	3	P051 60	R.NNIEM(+15.99)KW(+15.99)K.Y	Y	50, 65	1093 ,522 6	8	1, 7	547, 7695	2	2 7	1,22E+05			4,93E+0 5	4,01E+0 5	3,17E+0 5	3,66E+0 5	8,03E+0 4	1,18E+ 06	51	F51: 145 9	464	471	Oxidation (M); Oxidation (HW)	M5:Oxidat ion (M):1000.0 0;W7:Oxid ation (HW):1000 .00
2	3	P051 60	R.NNIEMKW(+31.99)KYEGK.V	Y	50, 21	1570 ,745	12	4, 3	786, 3831	2	2 9, 2				8,14E+0 6	4,76E+0 6	3,78E+0 6	1,04E+0 7	1,75E+0 6	3,52E+ 06	46	F46: 161 6	464	475		W7:Dihydr oxy:11.06
2	3	P051 60	R.NNIEMK.W	Y	49, 51	747, 3585	6	- 0, 6	374, 6863	2	1 6	4,29E+07	2,56E+07	3,60E+ 07							15	F15: 126 0	464	469		
2	3	P051 60	R.N(+.98)NIEMK.W	Y	46, 43	748, 3425	6	- 0, 2	375, 1785	2	1 8, 9	5,86E+06	5,62E+06	7,55E+ 06							24	F24: 152 9	464	469		N1:Deami dation (NQ):11.06
2	3	P051 60	R.NNIEM(+15.99)K.W	Y	45, 89	763, 3535	6	0, 8	382, 6843	2	1 6, 6	1,47E+06	1,58E+06								13	F13: 129 3	464	469	Oxidation (M)	M5:Oxidat ion (M):1000.0 0
2	3	P051 60	R.NNIEMK(+15.99)W(+15.99)K.Y	Y	45, 32	1093 ,522 6	8	5, 7	547, 7717	2	2 7, 4				6,16E+0 4		0,00E+0 0			2,06E+ 05	31	F31: 151 5	464	471	Oxidation (HW)	K6:Oxidati on or Hydroxylat

																										ion:22.87;<br>W7:Oxidat<br>ion<br>(HW):1000<br>.00
2	3	P051<br>60	R.NNIEM(+15.99)KW(+13.98)K.Y	Y	44,<br>98	1091<br>,507	8	4,<br>8	546,<br>7634	2	2<br>7,<br>5				2,48E+0<br>6	6,64E+0<br>5	7,81E+0<br>5	1,96E+0<br>6	6,20E+0<br>5	3,67E+<br>06	79	F79:<br>151<br>4	464	471	Oxidation<br>(M);<br>Tryptopha<br>n<br>oxidation<br>to<br>oxolacton<br>e	M5:Oxidat<br>ion<br>(M):1000.0<br>0;W7:Tryp<br>tophan<br>oxidation<br>to<br>oxolactone<br>:1000.00
2	3	P051<br>60	R.NNIEMKW(+15.99)K(+15.99)YEGK.<br>V	Y	44,<br>53	1570<br>,745	12	5,<br>1	786,<br>3838	2	2<br>8,<br>9					1,44E+0<br>6		4,67E+0<br>6	0,00E+0<br>0	7,94E+<br>05	39	F39:<br>162<br>0	464	475	Oxidation<br>(HW)	W7:Oxidat<br>ion<br>(HW):1000<br>.00;K8:Oxi<br>dation or<br>Hydroxylat<br>ion:0.00
2	3	P051<br>60	R.NNIEM(+15.99)K(+42.01)W(+3.99)K<br>YEGK.V	Y	43,<br>6	1600<br>,755<br>5	12	4,<br>3	801,<br>3885	2	2<br>7,<br>4					1,04E+0<br>5		1,82E+0<br>5	1,42E+0<br>5		63	F63:<br>151<br>3	464	475	Oxidation<br>(M);<br>Tryptopha<br>n<br>oxidation<br>to<br>kynurenin	M5:Oxidat<br>ion<br>(M):1000.0<br>0;K6:Acety<br>lation<br>(K):12.19;<br>W7:Trypto<br>phan<br>oxidation<br>to<br>kynurenin:<br>1000.00
2	3	P051<br>60	R.N(+15.99)NIEM(+15.99)K.W	Y	42,<br>91	779,<br>3483	6	0,<br>6	390,<br>6817	2	1<br>6,<br>2	2,76E+05	0,00E+00								6	F6:1<br>313	464	469	Oxidation<br>(M)	N1:Oxidati<br>on or<br>Hydroxylat<br>ion:17.01;<br>M5:Oxidat<br>ion<br>(M):1000.0<br>0
2	3	P051<br>60	R.NNIEM(+31.99)KWKYEGK.V	Y	41,<br>79	1570<br>,745	12	4,<br>4	786,<br>3832	2	2<br>9,<br>1					0,00E+0<br>0	1,49E+0<br>6				42	F42:<br>160<br>5	464	475	Dioxidatio<br>n (M)	M5:Dioxid<br>ation<br>(M):1000.0<br>0
2	3	P051<br>60	R.NNIEM(+31.99)KW(+15.99)KYEGK.<br>V	Y	40,<br>87	1586<br>,739<br>9	12	2,<br>4	794,<br>3792	2	2<br>7,<br>2						4,41E+0<br>6			9,65E+<br>05	54	F54:<br>148<br>6	464	475	Dioxidatio<br>n (M);<br>Oxidation<br>(HW)	M5:Dioxid<br>ation<br>(M):1000.0<br>0;W7:Oxid<br>ation<br>(HW):1000<br>.00
2	3	P051<br>60	R.NNIEMK(+42.01)W(+3.99)KYEGK.V	Y	39,<br>38	1584<br>,760<br>6	12	9,<br>5	793,<br>3951	2	2<br>9,<br>6					0,00E+0<br>0			0,00E+0<br>0		41	F41:<br>163<br>6	464	475	Tryptopha<br>n<br>oxidation<br>to<br>kynurenin	K6:Acetyla<br>tion<br>(K):0.00;W<br>7:Tryptoph<br>an<br>oxidation<br>to<br>kynurenin:<br>1000.00
2	3	P051<br>60	R.NNIEM(+15.99)KWK(+15.99).Y	Y	38,<br>7	1093<br>,522<br>6	8	4,<br>6	547,<br>7711	2	2<br>7,<br>8						1,77E+0<br>4		4,84E+0<br>4		70	F70:<br>153<br>2	464	471	Oxidation<br>(M)	M5:Oxidat<br>ion<br>(M):1000.0<br>0;K8:Oxida<br>tion or<br>Hydroxylat<br>ion:15.73
2	3	P051<br>60	R.NNIEMKWKY(+15.99)EGK.V	Y	37,<br>43	1554<br>,75	12	10	778,<br>3904	2	2<br>9					6,38E+0<br>4					42	F42:<br>159<br>6	464	475		Y9:Oxidati<br>on or<br>Hydroxylat<br>ion:0.00
2	3	P051<br>60	R.NNIEM(+31.99)K.W	Y	37,<br>23	779,<br>3483	6	8,<br>4	780,<br>3621	1	1<br>3,<br>2	1,46E+05	3,65E+05								3	F3:1<br>052	464	469	Dioxidatio<br>n (M)	M5:Dioxid<br>ation<br>(M):1000.0

																										0
2	3	P05160	R.NNIEM(+15.99)KW(+15.99)K(+15.99)YEGK.V	Y	35,57	1586,7399	12	3,1	794,3797	2	27,1						1,61E+06			9,19E+05	50	F50:1470	464	475	Oxidation (M); Oxidation (HW)	M5:Oxidation (M):1000.00;W7:Oxidation (HW):1000.00;K8:Oxidation or Hydroxylation:22.87
2	3	P05160	R.NNIEMK(+42.01)W(+19.99)KYEGK.V	Y	35,22	1600,7555	12	6,5	801,3903	2	27,6					1,48E+05					44	F44:1513	464	475	Tryptophan oxidation to hydroxykynurenin	K6:Acetylation (K):32.28; W7:Tryptophan oxidation to hydroxykynurenin:1000.00
2	3	P05160	K.WKYEGK.V	Y	54,8	809,4072	6	1,9	405,7116	2	18,6	5,44E+06	3,07E+06	7,09E+06							22	F22:1479	470	475		
2	3	P05160	K.WK(+15.99)YEGK.V	Y	38,54	825,4021	6	4,3	413,7101	2	25,9									1,81E+04	77	F77:1402	470	475		K2:Oxidation or Hydroxylation:13.27
2	3	P05160	K.YEGKVLHGDLID.F	Y	60,99	1357,6877	12	7	679,8559	2	29			7,83E+06							20	F20:2386	472	483		
2	3	P05160	K.YEGKVLHGDL.I	Y	39,79	1129,5768	10	6,3	377,5352	3	27,3			1,33E+06							21	F21:2233	472	481		
2	3	P05160	K.VLHGDLIDFVC(+31.99)K.Q	Y	52,53	1387,6805	12	5	694,851	2	36,3	8,45E+05	9,12E+05								2	F2:2952	476	487		C11:Dihydroxy:80.00
2	3	P05160	K.VLHGDLIDFVCK(+43.01)QGYDLSP.L	Y	44,68	2161,0513	19	8	1081,5415	2	34,6	1,44E+07	1,07E+07	7,97E+05							17	F17:2022	476	494		K12:Carbamylation:148.71
2	3	P05160	K.VLHGDLIDFVCK(+27.99)QGY.D	Y	42,2	1733,8446	15	14	867,9414	2	33,6		1,18E+07					9,93E+05			57	F57:2013	476	490		K12:Formylation:118.56
2	3	P05160	K.VLHGDLID.F	Y	40,5	880,4654	8	5,4	441,2423	2	28,9		2,61E+06	1,25E+07							21	F21:2383	476	483		
2	3	P05160	K.VLHGDLIDFVCK(-1.03)QGYDLSPLTPLSELSVQCNR.G	Y	38,39	3557,7534	32	5,3	1186,9314	3	41,1				3,90E+05						36	F36:2585	476	507	Lysine oxidation to aminoadipic semialdehyde	K12:Lysine oxidation to aminoadipic semialdehyde:1000.00
2	3	P05160	K.VLHGDLIDFVCKQGY.D	Y	38,14	1705,8497	15	12	853,942	2	33,8		0,00E+00								17	F17:1951	476	490		
2	3	P05160	K.VLHGDLIDFVCK(-1.03)QGYDLSPLTP.L	Y	36,54	2428,1985	22	2,7	810,409	3	37,1		1,13E+07								18	F18:2257	476	497	Lysine oxidation to aminoadipic semialdehyde	K12:Lysine oxidation to aminoadipic semialdehyde:1000.00
2	3	P05160	K.QGYDLSPLTPLSELSVQC(-33.99)NRGEVK(+14.96).Y	Y	56,73	2613,2922	24	-9,4	872,0965	3	38,1	1,02E+07			1,02E+07	8,57E+06		7,36E+06	2,15E+06		44	F44:2363	488	511	Dehydroalanine (C); Alpha-amino adipic acid	C18:Dehydroalanine (C):1000.00;K24:Alpha-amino adipic acid:1000.00
2	3	P051	K.QGYDLSPLTPLSEL.S	Y	53,	1531	14	6,	766,	2	3	6,87E+05									2	F2:3	488	501		

		60			22	,777		9	9011		8,3						109				
2	3	P05160	K.QGYDLSPLTPLSELSVQ.C	Y	45,37	1845,9359	17	6,8	923,9815	2	40,9				0,00E+00	29	F29:2570	488	504		
2	3	P05160	K.QGYDLSPLTPLSE.L	Y	45,17	1418,6929	13	4,6	710,357	2	35			4,09E+05		20	F20:2896	488	500		
2	3	P05160	K.QGYDLSPLTPL.S	Y	35,52	1202,6183	11	6,2	1203,6331	1	36,1		8,58E+05			12	F12:2940	488	498		
2	3	P05160	S.PLTPLSELSVQC(-33.99)NRGEVK(+14.96).Y	Y	37,22	1950,0057	18	-14	975,9963	2	39,8			2,95E+05		25	F25:2381	494	511	Dehydroalanine (C); Alpha-amino adipic acid	C12:Dehydroalanine (C):1000.00;K18:Alpha-amino adipic acid:1000.00
2	3	P05160	K.YPLC(+47.98)TR.K	Y	40,46	799,3535	6	7,5	800,3668	1	28,2		1,12E+06			12	F12:2280	512	517		C4:Cysteine oxidation to cysteic acid:100.00
2	3	P05160	R.KESK(+43.99)GMC(+79.96)TSPPLIK.H	Y	44,21	1593,7378	14	-1,1	797,8753	2	30,6		1,48E+05			17	F17:1700	518	531	O-Sulfonation (C)	K4:Carboxylation (DKW):9.42;C7:O-Sulfonation (C):1000.00
2	3	P05160	R.K(+42.01)ESK(+14.96)GMC(+31.99)TSPPLIK.H	Y	42,93	1606,7582	14	3,3	804,389	2	30,1		5,33E+05			16	F16:1677	518	531		K1:Acetylation (K):0.00;K4:Alpha-amino adipic acid:0.00;C7:Dihydroxy:83.88
2	3	P05160	R.K(+14.96)ESK(+42.01)GM(+31.99)CTSPPLIK.H	Y	37,6	1606,7582	14	5	804,3904	2	29,8	8,78E+05				9	F9:1653	518	531		K1:Alpha-amino adipic acid:0.00;K4:Acetylation (K):0.00;M6:Dioxidation (M):300.00
2	3	P05160	K.HGVIISSTVDTYE.N	Y	53,75	1419,6881	13	3,6	710,8539	2	66	1,26E+05	2,31E+06			1	F1:5438	532	544		
2	3	P05160	K.HGVIIS.S	Y	45,18	624,3595	6	-0,5	625,3665	1	25,4	0,00E+00	0,00E+00	3,25E+06		23	F23:2148	532	537		
2	3	P05160	S.VE(+43.99)YR(-43.05)CFDHHFLEGSR.E	Y	42,02	1894,7944	15	-2,6	948,402	2	29,7	6,76E+05				9	F9:1640	549	563		E2:Carboxylation (E):132.01;R4:Arginine oxidation to glutamic semialdehyde:151.14
2	3	P05160	E.Y(+43.01)RCF(+31.99)DHHFLEGSR.E	Y	38,02	1740,7427	13	13	871,3895	2	31		7,68E+05			16	F16:1749	551	563	Carbamylation	Y1:Carbamylation:1000.00;F4:Dihydroxy:14.02
2	3	P05160	R.C(+47.98)FDHHFLEGSR.E	Y	43,96	1394,567	11	5,8	465,8657	3	29,		6,20E+05			11	F11:236	553	563	Cysteine oxidation	C1:Cysteine

						3					2											7			to cysteic acid	oxidation to cysteic acid:1000.00
2	3	P05160	D.HHFLEGSR.E	Y	40,05	981,478	8	4,2	491,7484	2	37,6						1,95E+05				53	F53:2313	556	563		
2	3	P05160	K.NNLLLKWD(+15.99)FDNRPH.I	Y	62,74	1796,8958	14	4,6	899,4593	2	33,9				5,03E+06	4,70E+06	3,87E+06	4,26E+06	1,05E+07	6,06E+06	52	F52:1993	592	605		D8:Oxidation or Hydroxylation:15.73
2	3	P05160	K.NNLLLKW(+15.99)DFDNRPH.I	Y	61,97	1796,8958	14	6,4	899,4609	2	33,7				1,89E+07	1,61E+07	2,94E+07	2,05E+07	2,64E+07	2,12E+07	47	F47:1971	592	605		W7:Oxidation (HW):110.43
2	3	P05160	K.NNLLLKW(+31.99)DFDNRPH.I	Y	55,29	1812,8907	14	9,7	907,4615	2	33,8								3,70E+06	1,25E+06	74	F74:1992	592	605		W7:Dihydroxy:12.28
2	3	P05160	K.NNLLLKW(+13.98)DFDNRPH.I	Y	46,42	1794,8801	14	7,3	898,4539	2	34,9						1,17E+06		3,66E+06	1,35E+06	73	F73:2079	592	605	Tryptophan oxidation to oxolactone	W7:Tryptophan oxidation to oxolactone:1000.00
2	3	P05160	K.NNLLLK.W	Y	45,87	713,4435	6	2,6	714,4527	1	31,1	1,20E+08	8,68E+07	8,65E+06							1	F1:2565	592	597		
2	3	P05160	K.NNLLLK(+42.01)W(+3.99)DFDNRPH.I	Y	42,08	1826,9064	14	8,9	914,4686	2	33,8						1,63E+06		2,71E+06		66	F66:2004	592	605	Acetylation (K); Tryptophan oxidation to kynurenin	K6:Acetylation (K):1000.00;W7:Tryptophan oxidation to kynurenin:1000.00
2	3	P05160	K.NNLLLK(+15.99)WDFDNRPH.I	Y	38,58	1796,8958	14	6,8	599,9766	3	33,8				0,00E+00					1,53E+06	73	F73:1984	592	605		K6:Oxidation or Hydroxylation:14.04
2	3	P05160	K.W(+15.99)DFDNRPH.I	Y	55,22	1101,4628	8	6,4	551,7422	2	30,8				6,93E+06	1,11E+06		3,63E+06		9,15E+05	39	F39:1765	598	605		W1:Oxidation (HW):126.40
2	3	P05160	K.WDFDNRPHILHGEYIE.F	Y	52,91	2039,949	16	3,3	1020,9852	2	34,7		4,20E+06								10	F10:2899	598	613		
2	3	P05160	K.WDFDNRPH.I	Y	52,65	1085,4679	8	-1,4	543,7405	2	29	4,18E+07	3,07E+07	1,34E+07				4,50E+05		0,00E+00	15	F15:2515	598	605		
2	3	P05160	K.W(+31.99)DFDNRPH.I	Y	52,22	1117,4576	8	7,8	559,7405	2	27	1,01E+06	1,23E+06								11	F11:2174	598	605		W1:Dihydroxy:39.76
2	3	P05160	K.WD(+15.99)FDNRPH.I	Y	47,63	1101,4628	8	6,3	551,7421	2	30,7				8,78E+05						31	F31:1751	598	605		D2:Oxidation or Hydroxylation:52.04
2	3	P05160	K.WDFDNRPHILHGEYIEF.I	Y	35,22	2187,0173	17	8,5	1094,5253	2	37,5			0,00E+00							27	F27:2317	598	614		
2	3	P05160	H.ILHGEYIEFIC(+47.98)R.G	Y	65,71	1539,7391	12	6,2	770,8816	2	33,2	2,19E+06	2,22E+06								12	F12:2710	606	617	Cysteine oxidation to cysteic acid	C11:Cysteine oxidation to cysteic acid:1000.00
2	3	P05160	R.GDTYPAELYITGSILR.M	Y	110,5	1767,9043	16	5,8	884,9645	2	38,8	2,87E+08	2,48E+08	1,64E+08	5,95E+08	4,46E+08	6,75E+08	7,58E+08	6,09E+08	6,76E+08	62	F62:2393	618	633		
2	3	P05160	R.GDTYPAELY(+33.96)ITGSILR.M	Y	104,3	1801,8652	16	2,3	901,942	2	40				4,37E+06	2,98E+06	4,00E+06	9,00E+06	7,11E+06	5,11E+06	62	F62:2486	618	633		Y9:Chlorination of tyrosine residues:105.53

2	3	P05160	R.GDTY(+33.96)PAELYITGSILR.M	Y	87,27	1801,8652	16	6,7	901,9459	2	40,1									4,71E+06	77	F77:2529	618	633		Y4:Chlorination of tyrosine residues:53.98
2	3	P05160	R.GDT(-2.02)Y(+31.99)P(-30.01)AELYITGSILR.M	Y	53,02	1767,8679	16	5	884,9457	2	47,7						0,00E+00		0,00E+00	0,00E+00	77	F77:3034	618	633	Proline oxidation to pyrrolidinone	T3:2-amino-3-oxo-butanoic_acid:41.55;Y4:Dihydroxy:46.62;P5:Proline oxidation to pyrrolidinone:1000.00
2	3	P05160	R.GDTY(+33.96)PAELY(+33.96)ITGSILR.M	Y	47,97	1835,8263	16	10	918,9296	2	37,7	0,00E+00									3	F3:3074	618	633	Chlorination of tyrosine residues	Y4:Chlorination of tyrosine residues:1000.00;Y9:Chlorination of tyrosine residues:1000.00
2	3	P05160	T.YPAELYITGSILR.M	Y	84,41	1494,8082	13	4,6	748,4148	2	39,2		4,12E+06	5,03E+06	2,99E+06	8,13E+06	1,28E+07	1,67E+06	1,10E+07	5,66E+06	50	F50:2417	621	633		
2	3	P05160	Y.PAELYITGSILR.M	Y	85,89	1331,7449	12	5,5	666,8834	2	39,1	1,96E+07	3,37E+07	3,80E+07	5,07E+07	7,87E+07	9,42E+07	6,63E+07	9,61E+07	6,69E+07	46	F46:2434	622	633		
2	3	P05160	E.LYITGSILR.M	Y	66,05	1034,6124	9	1,4	518,3142	2	35,4	3,06E+07	4,14E+07	1,91E+07							1	F1:2965	625	633		
2	3	P05160	L.YITGSILR.M	Y	53,99	921,5283	8	4,1	461,7733	2	29,5		2,58E+05	1,39E+07							20	F20:2434	626	633		
2	3	P05160	R.GQLKYPR.C	Y	56,76	860,4868	7	1,2	431,2512	2	22,3	5,22E+06	5,80E+06	4,75E+06							13	F13:1810	639	645		
2	3	P05160	R.QSTLSYQEPLRT	Y	69,67	1421,7151	12	4,6	711,8681	2	29,4	3,25E+07	2,90E+07	1,46E+06							2	F2:2395	650	661		
2	3	P05160	R.QSTLSYQEPLR.T	Y	67,6	1320,6674	11	5,9	661,3448	2	29,5	3,22E+07	3,47E+07		2,55E+07	7,12E+06	4,30E+06	2,32E+07	5,53E+06	1,21E+07	12	F12:2390	650	660		
2	3	P05160	R.QSTLSYQEPL.R	Y	46,91	1164,5663	10	7,4	583,2947	2	31,3	1,00E+06	4,62E+05								2	F2:2553	650	659		
2	3	P05160	L.SYQEPLRT	Y	44,53	992,4927	8	5,6	497,2564	2	26,9	7,81E+06	6,95E+06	3,94E+07							11	F11:2167	654	661		
2	3	P05160	L.SYQEPLR.T	Y	41	891,445	7	1,8	446,7306	2	28,2	5,98E+06	7,64E+06	3,42E+06							10	F10:2279	654	660		
55	24	P07996	R.FVFGTTPEDILR.N	Y	66,98	1393,7241	12	5,2	697,873	2	34,8			2,88E+06							21	F21:2887	217	228		
55	24	P07996	K.GTSQNDPNWVVR.H	Y	57,45	1371,6531	12	2,9	686,8358	2	30,8			2,00E+06							19	F19:2632	969	980		
55	24	P07996	K.FQDLVDAVR.A	Y	56,81	1061,5505	9	5,6	531,7855	2	30,8			2,56E+06							20	F20:2544	75	83		
55	24	P07996	K.GPDPSSPAFR.I	Y	54,48	1029,4879	10	6,6	515,7546	2	26,4			2,21E+06							20	F20:2143	51	60		
55	24	P07996	R.AQGYSGLSVK.V	Y	47,5	1008,5241	10	5,1	505,2718	2	26,9			1,56E+06							21	F21:2193	1055	1064		
9	6	P08603	K.HGGLYHENMR.R	Y	92,34	1212,545	10	-0,	607,2796	2	18,	6,24E+06	7,98E+05	2,49E+06							24	F24:146	332	341		

						8		9			2											2				
9	6	P08603	R.NTEILTGSWSDQTYPEGTQAIYK.C	Y	88,67	2601,2234	23	-1,7	1301,6167	2	34,3	0	0	2,82E+06					0	0	13	F13:2953	29	51		
9	6	P08603	K.SSNLIILEEHLK.N	Y	83,52	1394,7769	12	7,2	698,4008	2	32,3	4,94E+06	3,85E+06	1,92E+06							2	F2:2635	755	766		
9	6	P08603	K.SPDVINGSPISQK.I	Y	80,43	1340,6936	13	1,7	671,3552	2	31,1	8,93E+06	1,10E+07	1,30E+07	1,05E+06	0	6,72E+05	7,33E+05	0	1,15E+04	1	F1:2558	212	224		
9	6	P08603	R.SSQESYAHGTK.L	Y	78,81	1193,5312	11	-3	597,7711	2	11,9	1,73E+06	7,84E+05	9,83E+05							24	F24:872	886	896		
9	6	P08603	R.KGEWVALNPLR.K	Y	77,51	1281,7194	11	-0,6	641,8666	2	32,3	7,60E+05	1,58E+06	9,88E+04	1,92E+06	1,20E+06	1,49E+06	2,33E+06	1,40E+06	1,16E+06	15	F15:2824	68	78		
9	6	P08603	K.IDVHLVPDR.K	Y	72,3	1062,5822	9	1,2	532,299	2	30,2	6,05E+06	3,72E+06	2,33E+06							22	F22:2590	574	582		
9	6	P08603	K.IVSSAM(+15.99)EPDR.E	Y	69,81	1119,5229	10	5,1	560,7716	2	24,4	3,60E+06	5,16E+06								2	F2:1963	157	166	Oxidation (M)	M6:Oxidation (M):1000.00
9	6	P08603	K.HGGLYHENM(+15.99)R.R	Y	69,54	1228,5408	10	1,8	615,2787	2	13,6	1,19E+06	2,35E+06	1,13E+05			2,64E+04				6	F6:1059	332	341	Oxidation (M)	M9:Oxidation (M):1000.00
9	6	P08603	H.MSDSYQYGEEVTYK.C	Y	68,86	1698,7083	14	-0,3	850,3611	2	29,7	0	0	0							24	F24:2559	945	958		
9	6	P08603	K.EFDHNSNIR.Y	Y	67,75	1130,5105	9	-0,3	566,2623	2	20,9	3,96E+06	2,99E+06	3,37E+06							24	F24:1710	770	778		
9	6	P08603	R.NGQWSEPPK.C	N	67,11	1041,4879	9	4,1	521,7534	2	28,3	6,35E+05	7,19E+05	2,66E+05							1	F1:2285	1154	1162		
9	6	P08603	R.EIMENYNIALR.W	N	67,05	1364,6758	11	-0,9	683,3445	2	32,8	0	1,49E+06	2,48E+06							13	F13:2818	1172	1182		
9	6	P08603	R.EYHFGQAVR.F	Y	65,43	1105,5305	9	6,7	553,7762	2	27,8	0	2,02E+06								12	F12:2241	167	175		
9	6	P08603	R.RPYFPVAVGK.Y	Y	64,98	1132,6393	10	2,9	567,3286	2	32,8	5,70E+06	1,00E+07		2,64E+05	2,80E+05	1,21E+06	2,60E+05		1,56E+06	1	F1:2731	342	351		
9	6	P08603	R.NGFYPATR.G	Y	63,31	924,4453	8	-0,2	463,2298	2	28	5,64E+06	2,80E+06	2,59E+06							15	F15:2414	296	303		
9	6	P08603	K.IVSSAM(+31.99)EPDR.E	Y	61,46	1135,5179	10	7,6	568,7706	2	24,8	8,44E+05	7,26E+05								2	F2:1996	157	166	Dioxidation (M)	M6:Dioxidation (M):1000.00
9	6	P08603	E.GFGIDGPAIAK.C	Y	61,11	1044,5603	11	2,4	523,2887	2	33,2	3,87E+06	4,14E+06	2,44E+06							10	F10:2759	962	972		
9	6	P08603	K.IVSSAMEPDRE.Y	Y	61	1232,5707	11	3,1	617,2946	2	28,8	7,46E+05	3,68E+06								10	F10:2338	157	167		
9	6	P08603	K.IVSSAMEPDREYHFGQAVR.F	Y	59,07	2191,0481	19	2,5	731,3585	3	30	2,35E+06	7,11E+06	0							15	F15:2610	157	175		
9	6	P08603	K.HGGLYHEN.M	Y	57,34	925,4042	8	5,2	463,7118	2	23,4	7,54E+04	2,32E+05								12	F12:1859	332	339		
9	6	P08603	R.KGEWVALNPLRK.C	Y	57,21	1409,8142	12	-2	470,9444	3	30,7	0									6	F6:2700	68	79		
9	6	P08603	E.SFTMIGHR.S	Y	56,86	947,4647	8	2,6	474,7408	2	30,7	1,74E+06	1,90E+06								1	F1:2519	722	729		
9	6	P08603	D.VHLVPDR.K	Y	56,78	834,4712	7	1,9	418,2437	2	28,9	6,58E+05	1,06E+06								10	F10:2345	576	582		

9	6	P08603	R.TKNDFTWFK.L	Y	56,74	1185,5818	9	3,1	593,8	2	34,4	1,05E+06			1	F1:2877	519	527		
9	6	P08603	K.IVSSAM(+15.99)EPDRE.Y	Y	56,45	1248,5656	11	2,9	625,2919	2	26,6		1,15E+06		10	F10:2135	157	167	Oxidation (M)	M6:Oxidation (M):1000.00
9	6	P08603	K.HGGLYHENM.R	Y	54,42	1056,4447	9	-2,2	529,2285	2	25,3	0			5	F5:2177	332	340		
9	6	P08603	K.GEWVALNPLR.K	Y	53,49	1153,6244	10	2,8	577,8211	2	33,5		0		11	F11:2723	69	78		
9	6	P08603	K.IIYKENER.F	Y	52,93	1063,5662	8	-0,8	532,7899	2	18,8	1,26E+06	9,05E+04	9,16E+05	22	F22:1504	225	232		
9	6	P08603	K.HGGLYHE.N	Y	51,14	811,3613	7	3,4	406,6893	2	26,2	1,37E+05	1,05E+06		10	F10:2098	332	338		
9	6	P08603	K.VGEVLK.F	Y	46,37	643,3904	6	-1,2	322,7021	2	24,3	9,72E+06	6,08E+06	5,00E+06	6	F6:2072	589	594		
9	6	P08603	E.FDHNSNIR.Y	Y	46,11	1001,4679	8	2,4	501,7424	2	25,9	1,69E+05	1,82E+06		10	F10:2066	771	778		
9	6	P08603	E.YHFGQAVR.F	Y	45,64	976,4879	8	2,3	489,2523	2	29,6	1,50E+06	1,64E+06		1	F1:2411	168	175		
9	6	P08603	R.CT(-2.02)LKPCDYPDIK.H	Y	44,82	1392,6417	12	-2,6	697,3264	2	29,5	0	0		4	F4:2581	320	331		T2:2-amino-3-oxo-butanoic_acid:38.00
9	6	P08603	K.NDFTWFK.L	Y	44,61	956,4392	7	0,7	479,2272	2	36	7,00E+05			1	F1:3020	521	527		
9	6	P08603	K.SPPEISHGVVAH.M	Y	44,39	1228,62	12	0,1	410,5473	3	27,1	0			5	F5:2357	933	944		
9	6	P08603	E.NYNIALR.W	N	43,78	862,4661	7	2,7	432,2415	2	31,5	3,94E+06	4,13E+06		1	F1:2600	1176	1182		
9	6	P08603	E.GTQAIYK.C	Y	43,54	779,4177	7	2,9	390,7173	2	26,7	1,74E+05	1,84E+06		1	F1:2146	45	51		
9	6	P08603	L.GEINYR.E	Y	40,55	750,366	6	-1,3	376,1898	2	21,3		4,09E+04	0	22	F22:1726	122	127		
9	6	P08603	E.WVALNPLR.K	Y	39,97	967,5603	8	2,7	484,7887	2	35,2	2,29E+06	3,26E+06		10	F10:2942	71	78		
9	6	P08603	K.LGYVTADGE.T	Y	39,91	923,4236	9	2	924,4327	1	30,7		1,92E+06		10	F10:2524	479	487		
9	6	P08603	K.DGWSAQPTCIK(-1.03)SCDIPVFMNAR.T	Y	39,08	2437,0864	22	4,9	813,3734	3	35	0			5	F5:3091	497	518	Lysine oxidation to aminoadipic semialdehyde	K11:Lysine oxidation to aminoadipic semialdehyde:1000.00
9	6	P08603	K.WSSPPQCEGLPCK(-2.02).S	Y	38,05	1428,6166	13	1,6	715,3167	2	30,6	5,17E+06	1,39E+06	1,66E+06	14	F14:2634	920	932		K13:2-amino-3-oxo-butanoic_acid:66.25
9	6	P08603	K.FVQGK.S	Y	36,89	577,3224	5	5,7	578,3329	1	23,5	4,87E+05	3,54E+05		1	F1:1867	406	410		
9	6	P08603	K.IVSSAMEPD(+15.99)REYHFGQAVR.F	Y	36,7	2207,043	19	-9	736,6816	3	29		0		15	F15:2513	157	175		D9:Oxidation or Hydroxylation:0.00
9	6	P086	K.WSSPPQCEGLP(-30.01)CK(+27.99).S	Y	34,	1428	13	1,	715,	2	3		1,69E+06	0	13	F13:	920	932		P11:Prolin

		03			28	,6166		8	3168		0,6						2612				e oxidation to pyrrolidinone:53.33;K13:Formylation:115.90
9	6	P08603	K.SSNLIILE.E	Y	33,74	887,4964	8	2,5	888,5059	1	36,6	1,16E+06	1,75E+06			1	F1:3080	755	762		
9	6	P08603	R.NTEILTGSW(+3.99)S(-2.02)DQTYPEGTQAIYK.C	Y	33,63	2603,2026	23	15	1302,6277	2	34,2			0		22	F22:2964	29	51	Tryptophan oxidation to kynurenin	W9:Tryptophan oxidation to kynurenin:1000.00;S10:2-amino-3-oxo-butanoic_acid:16.66
9	6	P08603	E.NGWSPTPR.C	Y	33,33	913,4406	8	0,8	457,7279	2	29,1	5,29E+05	4,92E+05			10	F10:2368	434	441		
9	6	P08603	R.EIM(+15.99)ENYNIALR.W	N	33,13	1380,6708	11	8,3	691,3484	2	30,5	6,85E+05				2	F2:2488	1172	1182	Oxidation (M)	M3:Oxidation (M):1000.00
9	6	P08603	K.C(-33.99)LHPC(+31.99)VISR.E	N	32,13	1024,5125	9	-13	513,257	2	25,8			5,10E+05		24	F24:2168	1163	1171		C1:Dehydroalanine (C):20.26;C5:Dihydroxy:11.06
9	6	P08603	K.YPSGER.V	N	28,7	707,3239	6	-6,1	354,6671	2	12,9			0		24	F24:966	1067	1072		
9	6	P08603	K.C(+27.99)LHP(-30.01)CVISR.E	N	28,18	1024,4946	9	1,8	513,2555	2	26			1,15E+06		22	F22:2179	1163	1171	Formylation; Proline oxidation to pyrrolidinone	C1:Formylation:1000.00;P4:Proline oxidation to pyrrolidinone:1000.00
9	6	P08603	H.FGQAVR.F	Y	27,27	676,3656	6	-0,5	339,1899	2	21,2	0				4	F4:1770	170	175		
9	6	P08603	E.RFQYK.C	Y	26,85	740,3969	5	2,4	371,2066	2	26,5		4,62E+05			10	F10:2122	232	236		
9	6	P08603	V.QGK(+43.01)SIDVAC(+31.99)HPGYALPK.A	Y	26,23	1857,9043	17	14	620,3174	3	30,9			2,25E+06		19	F19:2635	408	424		K3:Carbamylation:0.00;C9:Dihydroxy:36.80
9	6	P08603	Y.QCQNLYQLEGNKRITC(+15.99)R.N	N	25,4	2082,0098	17	5,6	1042,0179	2	37				0	59	F59:2262	1137	1153		C16:Oxidation or Hydroxylation (C):58.78
27	23	P0C0L4	K.ITQVLHFTK.D	N	61,9	1085,6233	9	6,8	543,8226	2	28,6			3,10E+07		20	F20:2344	1647	1655		
27	23	P0C0L4	R.DSSTWLTAFVLK.V	Y	60,62	1366,7133	12	3,2	684,3661	2	40,3			4,88E+06		19	F19:3501	1073	1084		
27	23	P0C0L4	K.VLSLAQEQVGGSPEK.L	N	46,54	1540,8097	15	9,3	771,4193	2	29,5			0		21	F21:2435	1085	1099		
27	23	P0C0L4	K.ITQVLHFT.K	N	46,31	957,5284	8	6,9	479,7748	2	30,9			2,39E+06		20	F20:2551	1647	1654		
27	23	P0C0L4	W.IEEM(+15.99)PSER.L	N	35,02	1005,4437	8	7,3	503,7328	2	22,5			5,21E+06		20	F20:1771	1709	1716	Oxidation (M)	M4:Oxidation (M):1000.0

																			0
27	23	P0C0L4	K.ITQVLHFTKDVK.A	N	31,71	1427,8136	12	-12	476,9395	3	27,8		2,96E+06	21	F21:2282	1647	1658		
27	23	P0C0L4	E.LQFSLGSK.I	N	30,3	878,4861	8	2,8	440,2516	2	32,7		2,53E+06	19	F19:2809	1358	1365		
27	23	P0C0L4	E.T(+27.99)KITQVLHFTK.D	N	30,21	1342,7609	11	11	448,599	3	32,6		4,32E+06	19	F19:2805	1645	1655		T1:Formylation:12.28
27	23	P0C0L4	K.VLREDSR.A	N	29,73	873,4668	7	7,2	437,7438	2	16,5		2,70E+05	20	F20:1295	1631	1637		
27	23	P0C0L4	R.NFLVR.A	N	29,61	647,3755	5	2	648,384	1	30,7		7,54E+06	19	F19:2617	1666	1670		
27	23	P0C0L4	R.YLDKTE.Q	N	27,49	767,3701	6	3,1	768,3798	1	25,3		4,77E+05	19	F19:2049	1027	1032		
27	23	P0C0L4	K.VLSLAQEQVGGSP(-30.01)EK(+31.99).L	N	26,02	1542,7889	15	1	772,4025	2	31,9	1,16E+06		1	F1:2645	1085	1099	Proline oxidation to pyrrolidinone; Dihydroxy	P13:Proline oxidation to pyrrolidinone:1000.00;K15:Dihydroxy:1000.00
26	25	P0C0L5	K.ITQVLHFTK.D	N	61,9	1085,6233	9	6,8	543,8226	2	28,6		3,10E+07	20	F20:2344	1647	1655		
26	25	P0C0L5	K.VLSLAQEQVGGSPEK.L	N	46,54	1540,8097	15	9,3	771,4193	2	29,5		0	21	F21:2435	1085	1099		
26	25	P0C0L5	K.ITQVLHFT.K	N	46,31	957,5284	8	6,9	479,7748	2	30,9		2,39E+06	20	F20:2551	1647	1654		
26	25	P0C0L5	W.IEEM(+15.99)PSER.L	N	35,02	1005,4437	8	7,3	503,7328	2	22,5		5,21E+06	20	F20:1771	1709	1716	Oxidation (M)	M4:Oxidation (M):1000.00
26	25	P0C0L5	K.ITQVLHFTKDVK.A	N	31,71	1427,8136	12	-12	476,9395	3	27,8		2,96E+06	21	F21:2282	1647	1658		
26	25	P0C0L5	E.LQFSLGSK.I	N	30,3	878,4861	8	2,8	440,2516	2	32,7		2,53E+06	19	F19:2809	1358	1365		
26	25	P0C0L5	E.T(+27.99)KITQVLHFTK.D	N	30,21	1342,7609	11	11	448,599	3	32,6		4,32E+06	19	F19:2805	1645	1655		T1:Formylation:12.28
26	25	P0C0L5	K.VLREDSR.A	N	29,73	873,4668	7	7,2	437,7438	2	16,5		2,70E+05	20	F20:1295	1631	1637		
26	25	P0C0L5	R.NFLVR.A	N	29,61	647,3755	5	2	648,384	1	30,7		7,54E+06	19	F19:2617	1666	1670		
26	25	P0C0L5	R.YLDKTE.Q	N	27,49	767,3701	6	3,1	768,3798	1	25,3		4,77E+05	19	F19:2049	1027	1032		
26	25	P0C0L5	K.VLSLAQEQVGGSP(-30.01)EK(+31.99).L	N	26,02	1542,7889	15	1	772,4025	2	31,9	1,16E+06		1	F1:2645	1085	1099	Proline oxidation to pyrrolidinone; Dihydroxy	P13:Proline oxidation to pyrrolidinone:1000.00;K15:Dihydroxy:1000.00
19	21	P12259	E.VIITGIQTQGAK.H	Y	67,8	1227,7186	12	3,5	614,8687	2	31,8		8,28E+06	19	F19:2726	1971	1982		
19	21	P12259	K.EVIITGIQTQGAK.H	Y	58,93	1356,7612	13	7,6	679,3931	2	30,2		3,63E+06	21	F21:2500	1970	1982		

19	21	P12259	K.ASKPGWWLLN(+.98)TE.V	Y	58,3	1401,6929	12	4,8	701,8571	2	37,8		4,99E+06	19	F19:3296	1876	1887	Deamidation (NQ)	N10:Deamidation (NQ):1000.00
19	21	P12259	Y.SLHAHGLSYEK.S	Y	50,58	1240,62	11	6	414,5498	3	25,1		0	20	F20:2014	1669	1679		
19	21	P12259	K.NFFNPPIISR.F	Y	42,76	1203,64	10	5,8	602,8307	2	33,4		2,77E+06	21	F21:2774	2190	2199		
19	21	P12259	K.DIHSGLIGPLL.I	Y	41,43	1133,6444	11	4,1	567,8318	2	36		7,86E+05	21	F21:2979	1739	1749		
19	21	P12259	K.YLDSTFTK.R	Y	37,06	973,4756	8	7,3	487,7487	2	27,9		2,01E+06	20	F20:2287	1623	1630		
19	21	P12259	S.SLTPK.H	N	28,48	544,322	5	4	273,1694	2	19,5	1,63E+05		11	F11:1540	310	314		
64	51	P13591	E.ASGDPIPSITWR.T	Y	51,28	1298,6619	12	3,2	650,3403	2	34,9		2,13E+06	19	F19:3033	331	342		
64	51	P13591	K.VFAKPK.I	Y	35,57	688,4271	6	4,5	345,2224	2	22		1,98E+05	21	F21:1736	304	309		
15	7	P19823	K.KFYNQVSTPLLR.N	Y	84,46	1464,8088	12	2,1	733,4133	2	33,1		2,21E+07	19	F19:2858	488	499		
15	7	P19823	K.IQPSGGTNINEALLR.A	Y	82,15	1581,8474	15	8,9	791,938	2	30,3		3,46E+07	20	F20:2506	380	394		
15	7	P19823	L.IILVSDGDPTVGELK.L	Y	78,54	1554,8505	15	7,4	778,4382	2	32,5		3,88E+06	20	F20:2696	416	430		
15	7	P19823	K.LWAYLTINQLLAER.S	Y	76,02	1702,9406	14	6,9	852,4835	2	38		1,14E+06	20	F20:3128	583	596		
15	7	P19823	K.FYNQVSTPLLR.N	Y	73,82	1336,7139	11	1,5	669,3652	2	33,9		1,90E+08	19	F19:2934	489	499		
15	7	P19823	R.KLGSYEHR.I	Y	63,5	988,509	8	3,8	495,2636	2	21,2		1,84E+07	20	F20:1672	190	197		
15	7	P19823	K.VQFELHYQEVK.W	Y	61,91	1418,7194	11	9,1	710,3734	2	29,6		6,35E+07	20	F20:2440	177	187		
15	7	P19823	L.VIENEAGDER.M	Y	59,14	1130,5204	10	2,7	566,269	2	23,2		2,06E+07	20	F20:1834	629	638		
15	7	P19823	R.KLWAYLTINQLLAE.R	Y	58,71	1674,9344	14	5,2	838,4789	2	40,9		3,99E+06	19	F19:3544	582	595		
15	7	P19823	K.LGSYEHR.I	Y	58,55	860,4141	7	4,7	431,2163	2	20,1		7,74E+06	20	F20:1580	191	197		
15	7	P19823	K.MKQTVEAMK.T	Y	58,44	1064,5359	9	7,2	533,2791	2	24,1		1,93E+06	20	F20:1916	326	334		
15	7	P19823	K.IQPSGGTNINE.A	Y	56,6	1128,5411	11	5,5	565,2809	2	26,1		9,92E+06	20	F20:2112	380	390		
15	7	P19823	K.TQVADAK.R	Y	55,91	731,3813	7	3,7	732,3913	1	18,2		4,46E+05	19	F19:1448	368	374		
15	7	P19823	K.NILFVIDVSGSM(-48.00)W(+19.99)GVK(+42.01).M	Y	54,66	1777,925	16	13	889,9818	2	66,3		1,65E+06	19	F19:5611	310	325	Dethiomethyl; Tryptophan oxidation to hydroxykynurenin; Acetylation (K)	M12:Dethiomethyl:1000.00;W13:Tryptophan oxidation to hydroxykynurenin:1000.00;K16:

																		Acetylation (K):1000.00
15	7	P19823	K.MKQTVEAM(+15.99)K.T	Y	54,52	1080,5308	9	6,8	541,2763	2	20,2	2,66E+05	21	F21:1601	326	334		M8:Oxidation (M):137.02
15	7	P19823	Y.LTINQLLAER.S	Y	51,95	1169,6768	10	5,8	585,8491	2	33,6	6,73E+06	20	F20:2788	587	596		
15	7	P19823	K.LWAYLTINQ(+.98)LLAER.S	Y	50,67	1703,9246	14	-7,8	852,9629	2	40,7	6,18E+06	19	F19:3536	583	596		Q9:Deamidation (NQ):30.46
15	7	P19823	K.M(+15.99)KQTVEAM(+15.99)K.T	Y	50,36	1096,5256	9	6,7	549,2737	2	14,5	8,18E+05	20	F20:1130	326	334	Oxidation (M)	M1:Oxidation (M):1000.00;M8:Oxidation (M):1000.00
15	7	P19823	L.TINQLLAER.S	Y	50,27	1056,5928	9	6,3	529,307	2	29,8	7,79E+06	20	F20:2460	588	596		
15	7	P19823	K.LWAYLTINQLLAE.R	Y	49,96	1546,8395	13	4	774,4301	2	54,9	2,03E+05	19	F19:4685	583	595		
15	7	P19823	K.KFYNQVSTPLL.R	Y	48,96	1308,7078	11	2,4	655,3627	2	35,1	1,23E+07	19	F19:3051	488	498		
15	7	P19823	K.TILDDLR.A	Y	48,32	844,4654	7	2,4	423,241	2	33,9	1,04E+08	19	F19:2930	335	341		
15	7	P19823	K.FYNQVSTPLL.R	Y	47,33	1180,6128	10	5,6	591,317	2	34,5	4,43E+06	20	F20:2862	489	498		
15	7	P19823	R.KLWAYLTINQLLAER.S	Y	46,79	1831,0355	15	4	611,3549	3	36,4	1,39E+06	21	F21:3012	582	596		
15	7	P19823	Q.DFLSKDK.H	Y	46,15	851,4388	7	2	852,4478	1	28,3	4,04E+06	19	F19:2362	567	573		
15	7	P19823	K.LWAYLTIN(+.98)QLLAER.S	Y	45,47	1703,9246	14	-12	852,9594	2	41,3	6,18E+06	19	F19:3577	583	596		N8:Deamidation (NQ):0.00
15	7	P19823	K.VQSTITSR.M	Y	45,3	890,4821	8	2,7	446,2495	2	25,4	1,04E+08	19	F19:2061	77	84		
15	7	P19823	E.LHYQEVK.W	Y	42,85	915,4814	7	6,4	458,7509	2	24,1	2,66E+06	20	F20:1922	181	187		
15	7	P19823	K.QTVEAM(+15.99)K.T	Y	42,18	821,3953	7	3,5	411,7064	2	13,6	9,56E+04	20	F20:1057	328	334	Oxidation (M)	M6:Oxidation (M):1000.00
15	7	P19823	R.KLGSYEH.R	Y	41,52	832,4079	7	5,9	417,2137	2	21,7	1,06E+06	20	F20:1710	190	196		
15	7	P19823	F.DVDYDFLKR.L	Y	40,82	1169,5717	9	5,2	585,7961	2	30,6	3,02E+05	21	F21:2536	456	464		
15	7	P19823	Q.NVVFDVQIPK.G	Y	38,67	1157,6444	10	6,6	579,8333	2	33	1,09E+06	21	F21:2740	103	112		
15	7	P19823	D.YDFLKR.L	Y	37,94	840,4493	6	2,4	421,2329	2	30,5	3,98E+06	19	F19:2599	459	464		
15	7	P19823	K.TAGLVR.S	Y	35,9	615,3704	6	2,3	616,379	1	26,4	2,98E+06	19	F19:2161	151	156		
15	7	P19823	R.KLWAYLTINQL.L	Y	35,85	1361,7708	11	4,6	681,8958	2	35,8	1,05E+06	21	F21:2963	582	592		
15	7	P19823	R.ALYAQA.R	Y	35,69	635,3279	6	6,1	636,3391	1	26,	3,73E+07	20	F20:215	140	145		

											6			8				
15	7	P19823	W.AYLTINQLLAER.S	Y	35,36	1403,7772	12	6,6	702,9005	2	34,6	3,00E+05	21	F21:2873	585	596		
15	7	P19823	E.VFNGYFVHFFAPD(+15.99)N(+15.99)LDPIPK.N	Y	33,59	2368,1528	20	-4,5	790,388	3	39,4	8,26E+05	19	F19:3426	290	309		D13:Oxidation or Hydroxylation:8.69;N14:Oxidation or Hydroxylation:5.99
15	7	P19823	S.FKPTVAQQR.I	Y	32,66	1073,5981	9	3,4	537,8082	2	27	1,50E+06	19	F19:2227	251	259		
15	7	P19823	K.DKHADP(-27.99)DFTRK.L	Y	31,22	1300,6523	11	-5,7	651,3297	2	25,2	1,07E+07	19	F19:2041	572	582	Pyrrolidone from Proline	P6:Pyrrolidone from Proline:1000.00
15	7	P19823	H.FFAP(+31.99)D(+15.99)NLDPIPK.N	Y	31,21	1420,6874	12	-5,7	711,3469	2	30,6	1,35E+07	20	F20:2530	298	309		P4:Dihydroxy:34.83;D5:Oxidation or Hydroxylation:12.28
15	7	P19823	R.KLGSYE.H	Y	30,45	695,349	6	2,4	696,358	1	26,3	1,24E+07	19	F19:2155	190	195		
15	7	P19823	E.GHFDGVP(-30.01)VISK(+15.99).G	Y	29,87	1140,5928	11	1,8	571,3047	2	30,8	3,62E+06	19	F19:2623	232	242	Proline oxidation to pyrrolidinone	P7:Proline oxidation to pyrrolidinone:1000.00;K11:Oxidation or Hydroxylation:23.76
15	7	P19823	F.DVQIPK.G	Y	29,77	698,3962	6	4,4	699,4066	1	26,6	5,52E+07	21	F21:2168	107	112		
15	7	P19823	H.FFAP(+31.99)DN(+15.99)LDPIPK.N	Y	28,28	1420,6874	12	-7,5	711,3456	2	30,6	1,25E+07	21	F21:2539	298	309		P4:Dihydroxy:23.70;N6:Oxidation or Hydroxylation:0.00
15	7	P19823	K.LWAYLT.I	N	28,1	765,4061	6	3,9	766,4164	1	36,7	1,81E+06	19	F19:3190	583	588		
15	7	P19823	E.IVVAGK.F	Y	26,68	585,3849	6	5,3	586,3953	1	23,2	2,99E+07	20	F20:1835	527	532		
15	7	P19823	Q.N(+.98)VVFDVQIPK.G	Y	26,62	1158,6284	10	6,4	580,3252	2	33,8	0	20	F20:2802	103	112		N1:Deamidation (NQ):35.17
15	7	P19823	K.MKQTVE.A	Y	26,57	734,3633	6	6,3	735,3752	1	22,4	4,58E+05	19	F19:1790	326	331		
15	7	P19823	Q.DFLSK.D	Y	25,71	608,317	5	1,4	609,3251	1	29,3	4,05E+07	19	F19:2466	567	571		
15	7	P19823	H.FFAP(+31.99)D(+15.99)N(+15.99)LDPIPK.N	Y	25,52	1436,6823	12	-3,3	719,346	2	31,3	1,13E+06	20	F20:2587	298	309		P4:Dihydroxy:23.70;D5:Oxidation or Hydroxylation:10.22;N6:Oxidation or Hydroxylation:13.67
52	12	P19827	K.AAISGENAGLVR.A	Y	76,25	1156,62	12	3,6	579,3193	2	27,3	7,71E+07	20	F20:2224	126	137		

52	12	P19827	R.GHM(+31.99)LENHVER.L	Y	69,03	1252,5619	10	7,4	627,2928	2	22,7	1,05E+07	20	F20:1790	555	564	Dioxidation (M)	M3:Dioxidation (M):1000.00
52	12	P19827	R.GHM(+15.99)LENHVER.L	Y	68,49	1236,5669	10	7	619,295	2	22,3	3,78E+07	20	F20:1757	555	564	Oxidation (M)	M3:Oxidation (M):1000.00
52	12	P19827	R.FPLYNLGFGHN(+.98)VD.F	Y	58,34	1492,6986	13	2,8	747,3586	2	37,5	6,98E+06	19	F19:3264	428	440		N11:Deamidation (NQ):42.99
52	12	P19827	R.GHMLENHVER.L	Y	57,14	1220,572	10	5,7	407,8669	3	23,9	3,25E+06	21	F21:1911	555	564		
52	12	P19827	R.GRFPLYNLGFG.H	Y	55,95	1239,64	11	3,7	620,8296	2	37,9	1,92E+07	19	F19:3299	426	436		
52	12	P19827	R.GRFPLYNLGFGHN(+.98)VD.F	Y	51,38	1705,8212	15	4,1	853,9213	2	36,4	7,39E+06	19	F19:3164	426	440		N13:Deamidation (NQ):95.35
52	12	P19827	R.ERGHM(+15.99)LENHVER.L	Y	47,05	1521,7107	12	4,9	381,4368	4	22,7	3,11E+06	20	F20:1787	553	564	Oxidation (M)	M5:Oxidation (M):1000.00
52	12	P19827	R.KAAISGENAGLV.R	Y	47,03	1128,6139	12	7,4	565,3184	2	27,9	9,76E+06	20	F20:2286	125	136		
52	12	P19827	G.FVTPLTSMSIR.G	Y	46,8	1250,6693	11	2,6	626,3436	2	35,4	8,35E+06	19	F19:3076	602	612		
52	12	P19827	G.FVTPLTSM(+15.99)SIR.G	Y	45,72	1266,6642	11	9,2	634,3452	2	30,4	1,28E+07	21	F21:2517	602	612	Oxidation (M)	M8:Oxidation (M):1000.00
52	12	P19827	F.VTPLTSMSIR.G	Y	45,48	1103,6008	10	1,5	552,8085	2	32,4	3,27E+06	19	F19:2785	603	612		
52	12	P19827	R.IYEDHDATQQLQGF.Y	Y	44,9	1663,7478	14	9,7	832,8893	2	30,3	2,50E+07	20	F20:2507	458	471		
52	12	P19827	R.GRFPLYNLGF.G	Y	44,59	1182,6185	10	4,3	592,3191	2	35,6	3,17E+05	20	F20:2945	426	435		
52	12	P19827	R.FPLYNLGFG.H	N	41,48	1026,5175	9	6	1027,5309	1	39,7	5,17E+06	19	F19:3452	428	436		
52	12	P19827	K.SFSGK.K	N	35,07	524,2595	5	4,7	525,2692	1	21,6	1,64E+05	19	F19:1725	224	228		
52	12	P19827	K.AAISGENAGL.V	Y	34,95	901,4505	10	9,2	902,4661	1	28,4	1,57E+06	21	F21:2336	126	135		
52	12	P19827	K.VTAWK.Q	Y	33,18	603,338	5	5,2	302,6779	2	24,5	1,39E+07	21	F21:1967	117	121		
52	12	P19827	R.GRFPLYNL.G	Y	32,43	978,5287	8	7,3	490,2752	2	33,1	9,13E+05	20	F20:2748	426	433		
52	12	P19827	R.KAAISGE.N	Y	32,1	674,3599	7	3,6	675,3696	1	18,5	2,32E+05	19	F19:1469	125	131		
52	12	P19827	R.GHM(+15.99)LENHVERL.W	Y	31,75	1349,651	11	6,6	450,8939	3	25,7	1,29E+06	20	F20:2077	555	565	Oxidation (M)	M3:Oxidation (M):1000.00
52	12	P19827	K.NVVFVID.I	Y	31,67	804,4381	7	2,2	805,4471	1	36,7	5,22E+06	19	F19:3198	292	298		
52	12	P19827	K.DKVTAW(+15.99)K.Q	Y	31,14	862,4548	7	5,9	432,2372	2	24,1	1,61E+06	20	F20:1919	115	121	Oxidation (HW)	W6:Oxidation (HW):1000.00
52	12	P19827	K.AAISGENAGLV.R	Y	29,56	1000,518	11	11	1001,536	1	29,	1,12E+06	21	F21:246	126	136		

						9			9		8											2		
52	12	P198 27	K.TAFISD.F	Y	28, 11	652, 3068	6	7, 9	653, 3192	1	2 8, 6			5,58E+ 06							21	F21: 235 6	94	99
52	12	P198 27	R.LWAYLT.I	N	28, 1	765, 4061	6	3, 9	766, 4164	1	3 6, 7			1,81E+ 06							19	F19: 319 0	565	570
52	12	P198 27	R.LWAYLTIQELLAK.R	Y	27, 19	1560 ,891 5	13	5, 9	781, 4576	2	3 8, 8			3,50E+ 05							20	F20: 319 2	565	577
80	27	P223 52	K.FLVGPDGIPIMR.W	Y	52, 2	1313 ,716 6	12	- 0, 1	657, 8655	2	3 5, 5	7,15E+05	0	9,18E+ 06							24	F24: 310 1	186	197
80	27	P223 52	R.TTVSNVK.M	Y	42, 39	747, 4127	7	- 0, 9	374, 7133	2	1 3, 6	2,33E+05	1,27E+05	1,83E+ 05							4	F4:1 048	202	208
80	27	P223 52	K.QEPGENSEILPTLK.Y	Y	37, 7	1553 ,793 7	14	- 6, 3	777, 8992	2	3 2, 1		0	0							14	F14: 277 9	107	120
80	27	P223 52	K.FYTFLK.N	Y	32, 37	817, 4374	6	2, 5	818, 4467	1	3 5, 2	0									1	F1:2 945	148	153
80	27	P223 52	R.WNFEK.F	Y	30, 29	722, 3387	5	- 0, 6	362, 1764	2	2 8, 8	0	0								5	F5:2 517	181	185
80	27	P223 52	K.VHDIR.W	Y	28, 57	638, 35	5	2, 8	320, 1832	2	1 1, 6	0	0								15	F15: 848	176	180
61	26	P231 42	R.SAATLQQEK.T	Y	59, 72	974, 5032	9	7, 4	488, 2625	2	2 3, 5	8,16E+05	9,33E+05	8,50E+ 04							12	F12: 186 8	563	571
61	26	P231 42	R.GYHLNEEGTR.C	Y	58, 6	1174 ,536 6	10	- 0, 7	588, 2751	2	2 4, 7	1,40E+06	0	0	1,05E+0 5	4,68E+0 4	2,14E+0 5	1,49E+0 5	4,61E+0 4	5,22E+ 05	24	F24: 206 3	344	353
61	26	P231 42	K.TGYYFDGISR.M	Y	57, 9	1177 ,540 4	10	8, 2	589, 7823	2	3 0, 7	5,96E+05	1,10E+06								3	F3:2 509	386	395
61	26	P231 42	R.LSVDGR.S	Y	40, 99	645, 3445	6	- 0, 3	323, 6794	2	2 0, 8	2,10E+06	9,00E+05	6,22E+ 05							23	F23: 170 9	432	437
61	26	P231 42	R.LAPNGR.N	Y	30, 85	626, 35	6	1, 9	314, 1829	2	1 3	0									4	F4:9 84	516	521
61	26	P231 42	N.IIFDITEGNLR.D	Y	25, 86	1289 ,697 9	11	6, 9	645, 8607	2	3 4	0									2	F2:2 778	632	642
82	66	P369 80	R.NGQWSEPPK.C	N	67, 11	1041 ,487 9	9	4, 1	521, 7534	2	2 8, 3	6,35E+05	7,19E+05	2,66E+ 05							1	F1:2 285	194	202
91	44	P607 09	R.VAPEEHPVL.L	N	35, 57	989, 5182	9	6, 4	495, 7695	2	2 8			2,67E+ 06							20	F20: 229 1	96	104
91	44	P607 09	L.TEAPLNPK.A	N	33, 74	868, 4654	8	5, 9	435, 2425	2	2 4, 8			4,10E+ 05							20	F20: 198 6	106	113
91	44	P607 09	E.SGPSIVHR.K	N	33, 1	851, 4613	8	1, 8	426, 7387	2	2 6, 4			0							19	F19: 216 4	365	372
92	43	P632 61	R.VAPEEHPVL.L	N	35, 57	989, 5182	9	6, 4	495, 7695	2	2 8			2,67E+ 06							20	F20: 229 1	96	104
92	43	P632 61	L.TEAPLNPK.A	N	33, 74	868, 4654	8	5, 9	435, 2425	2	2 4, 8			4,10E+ 05							20	F20: 198 6	106	113
92	43	P632 61	E.SGPSIVHR.K	N	33, 1	851, 4613	8	1, 8	426, 7387	2	2 6, 4			0							19	F19: 216 4	365	372
17	11	P981 60	K.AQIHGGILR.L	Y	66, 4	963, 5614	9	8	482, 7918	2	2 7, 4		3,34E+05								12	F12: 220 8	1911	191 9
17	11	P981 60	R.HLISTHFAPGDFQGFALVNPQR.N	Y	63, 04	2451 ,244 6	22	3, 7	818, 0919	3	3 7, 5	5,54E+05									1	F1:3 153	1342	136 3
17	11	P981	R.AVLHVHGGGGPR.V	Y	55,	1155	12	0,	386,	3	1	2,56E+05	1,27E+05								5	F5:1	1946	195

		60			81	,6261		5	2162		8,9									559		7		
17	11	P98160	R.HQTHGSLLR.L	Y	52,85	1047,5574	9	1	350,1934	3	14,2	4,55E+05	0						5	F5:1114	2196	2204		
17	11	P98160	E.WTGGPGGQLPAK.A	Y	52,65	1167,6036	12	1,9	584,8102	2	30,3	1,05E+06							1	F1:2488	1899	1910		
17	11	P98160	R.ASYAQQPAESR.V	Y	45,12	1206,5629	11	7,3	604,2931	2	24	1,46E+05							3	F3:1935	1091	1101		
17	11	P98160	R.EDGRPVPSGTQQR.H	Y	43,45	1425,696	13	11	713,8628	2	24,2	1,21E+05	0						12	F12:1931	1713	1725		
17	11	P98160	K.RGGSLPAR.H	Y	41,4	812,4617	8	5,3	407,2403	2	22,7		1,03E+05						12	F12:1803	2188	2195		
17	11	P98160	R.VQVSPER.T	Y	40,54	813,4344	7	-0,3	407,7244	2	19,1	1,13E+06	1,34E+06	4,76E+05					15	F15:1551	1958	1964		
17	11	P98160	E.VAQPGPSNRPR.A	Y	39,61	1177,6316	11	3,3	589,825	2	25,2	3,26E+05	4,18E+05						10	F10:2006	1513	1523		
17	11	P98160	R.FTVTQR.S	Y	35,94	750,4024	6	5,8	376,2107	2	25,6	0							2	F2:2068	1011	1016		
17	11	P98160	R.YQLGSGEAR.L	Y	30,55	979,4723	9	6,4	490,7466	2	30,1						3,88E+05		46	F46:1686	4273	4281		
17	11	P98160	R.AM(+15.99)DFNGILTIR.N	Y	27,51	1265,6438	11	6,3	633,8332	2	33,2	3,87E+05							2	F2:2716	1818	1828	Oxidation (M)	M2:Oxidation (M):1000.00
17	11	P98160	R.GGSLPAR.H	Y	26,9	656,3605	7	5,6	657,3715	1	22,4	5,80E+04	1,30E+05						2	F2:1801	2189	2195		
41	31	Q03591	R.NGQWSEPPK.C	N	67,11	1041,4879	9	4,1	521,7534	2	28,3	6,35E+05	7,19E+05	2,66E+05					1	F1:2285	253	261		
41	31	Q03591	R.EIMENYNIALR.W	N	67,05	1364,6758	11	-0,9	683,3445	2	32,8	0	1,49E+06	2,48E+06					13	F13:2818	271	281		
41	31	Q03591	E.NYNIALR.W	N	43,78	862,4661	7	2,7	432,2415	2	31,5	3,94E+06	4,13E+06						1	F1:2600	275	281		
41	31	Q03591	R.EIM(+15.99)ENYNIALR.W	N	33,13	1380,6708	11	8,3	691,3484	2	30,5	6,85E+05							2	F2:2488	271	281	Oxidation (M)	M3:Oxidation (M):1000.00
41	31	Q03591	K.C(-33.99)LHPC(+31.99)VISR.E	N	32,13	1024,5125	9	-13	513,257	2	25,8			5,10E+05					24	F24:2168	262	270		C1:Dehydroalanine (C):20.26;C5:Dihydroxy:11.06
41	31	Q03591	K.YPSGER.V	N	28,7	707,3239	6	-6,1	354,6671	2	12,9			0					24	F24:966	166	171		
41	31	Q03591	K.C(+27.99)LHP(-30.01)CVISR.E	N	28,18	1024,4946	9	1,8	513,2555	2	26			1,15E+06					22	F22:2179	262	270	Formylation; Proline oxidation to pyrrolidinone	C1:Formylation:1000.00;P4:Proline oxidation to pyrrolidinone:1000.00
41	31	Q03591	Y.QCQNLYQLEGNKRITC(+15.99)R.N	N	25,4	2082,0098	17	5,6	1042,0179	2	37							0	59	F59:2262	236	252		C16:Oxidation or Hydroxylation (C):58.78
45	22	Q06033	E.ASFITNDLLGSALTK.S	Y	66,51	1549,8351	15	3	775,9271	2	37,6			3,94E+06					19	F19:3275	201	215		
45	22	Q06	R.NAIGGKFPLYNLGFG.N	Y	64,	1566	15	3,	784,	2	3			1,44E+					19	F19:	414	428		

		033			78	,8195		3	4196		8,7			06								3371				
45	22	Q06033	R.NAIGGKFPLYNLGFGNN.L	Y	56,52	1794,9053	17	6,8	898,466	2	35,2			0							21	F21:2920	414	430		
45	22	Q06033	K.KGHVSFKPSLDQQR.S	Y	56,08	1625,8638	14	3,2	542,9636	3	29,2			1,24E+06							19	F19:2451	221	234		
45	22	Q06033	M.LTDGDANVGESRPEK.I	Y	43,18	1586,7535	15	9,7	794,3917	2	24,5			9,44E+06							21	F21:1966	393	407		
45	22	Q06033	K.FPLYNLGFG.N	N	41,48	1026,5175	9	6	1027,5309	1	39,7			5,17E+06							19	F19:3452	420	428		
45	22	Q06033	T.YDVNR.E	Y	39,28	665,3133	5	4,5	333,6654	2	19,4			3,20E+05							20	F20:1528	252	256		
45	22	Q06033	K.EVSFDVELPK.T	Y	38,31	1161,5917	10	7	581,8072	2	32,8			0							20	F20:2719	76	85		
45	22	Q06033	K.IQENVR.N	Y	36,54	757,4082	6	5,2	379,7133	2	21,9			1,18E+06							20	F20:1728	408	413		
45	22	Q06033	K.SFSGK.K	N	35,07	524,2595	5	4,7	525,2692	1	21,6			1,64E+05							19	F19:1725	216	220		
45	22	Q06033	F.ITNDLLGSALTK.S	Y	32,09	1244,6976	12	7,3	623,3607	2	31,9			0							20	F20:2641	204	215		
45	22	Q06033	R.LWAYLT.I	N	28,1	765,4061	6	3,9	766,4164	1	36,7			1,81E+06							19	F19:3190	557	562		
45	22	Q06033	K.VQPK(-1.03)Q(+.98)LVKHFE.I	Y	26,48	1351,7135	11	9,8	676,8707	2	32,4			1,26E+07							19	F19:2786	173	183		K4:Lysine oxidation to aminoadipic semialdehyde:33.81; Q5:Deamidation (NQ):19.16
84	62	Q08380	K.YSSDYFQAPSDYR.Y	Y	80,92	1597,6685	13	9	799,8487	2	30,3	8,85E+05									3	F3:2474	442	454		
84	62	Q08380	R.RGPLVK.Y	Y	26,98	668,4333	6	4,9	335,2256	2	21,7		3,50E+04								11	F11:1721	436	441		
20	10	Q12805	R.SVPSDIFQIQATTIYANTINTFR.I	Y	92,62	2599,3281	23	7,5	1300,681	2	42,2	6,19E+05	2,57E+05	3,80E+05	3,53E+06	2,36E+06	6,12E+06	2,65E+06	7,32E+05	8,37E+06	81	F81:2669	405	427		
20	10	Q12805	R.EHIVDLEMLTVSSIGTFR.T	Y	86,7	2046,0455	18	6,4	1024,0366	2	40,3									5,59E+05	77	F77:2542	460	477		
20	10	Q12805	R.QTSPVSAM(+15.99)LVLVK.S	Y	72,37	1387,7744	13	6,3	694,8989	2	32,1	3,07E+06	1,53E+06		8,00E+05			1,59E+06		1,67E+06	2	F2:2623	441	453	Oxidation (M)	M8:Oxidation (M):1000.00
20	10	Q12805	R.IPSNPSHR.I	Y	71,32	906,4672	8	0,4	454,2411	2	13,5	2,07E+06	2,17E+06	1,72E+06							15	F15:1027	149	156		
20	10	Q12805	K.SGNENGEFYLR.Q	Y	71,07	1284,5735	11	8,4	643,2994	2	32,7	4,10E+06	9,58E+05	0	2,65E+06	2,13E+06	0	4,27E+06	0	0	63	F63:1923	430	440		
20	10	Q12805	R.QTSPVSAMLVLVK.S	Y	69,49	1371,7795	13	3,6	686,8995	2	38,8	3,19E+06	1,19E+06								1	F1:3264	441	453		
20	10	Q12805	R.TSSYLCQY(-2.02)QCVN(+15.99)EPGK.F	Y	65,46	1832,7709	16	7,9	917,4	2	32,3	0	1,12E+07	5,31E+06	1,06E+07	7,18E+06		3,72E+06	5,32E+06		29	F29:1880	300	315		Y8:2-amino-3-oxo-butanoic_acid:22.37; N12:Oxidation or

																										Hydroxylation:55.92
20	10	Q12805	R.ELPQSIVYK.Y	Y	47,2	1075,5913	9	7,1	538,8068	2	29,9	4,14E+06	1,26E+06								2	F2:2438	388	396		
20	10	Q12805	R.RNPADPQR.I	Y	46,2	952,4838	8	-1,1	477,2487	2	11,7	0	0								14	F14:843	141	148		
20	10	Q12805	R.TSSVLR.L	Y	37,47	661,3759	6	-0,5	331,695	2	16,8	1,16E+06	1,86E+05	0							4	F4:1359	478	483		
20	10	Q12805	K.YMSIR.S	Y	36,31	668,3315	5	3,2	669,3409	1	29,1	0									1	F1:2367	397	401		
20	10	Q12805	K.YM(+15.99)SIR.S	Y	34,32	684,3265	5	5,7	343,1725	2	23,3	1,72E+05	1,03E+05								11	F11:1854	397	401	Oxidation (M)	M2:Oxidation (M):1000.00
20	10	Q12805	A.VAGPEMQTGR(+15.99).N	Y	34,04	1060,4971	10	9	531,2606	2	27,4				1,50E+04	3,29E+04	2,41E+05	4,54E+05	1,31E+05	7,22E+05	57	F57:1506	125	134		R10:Oxidation or Hydroxylation:68.95
20	10	Q12805	S.PVSAMLVLVK.S	Y	34	1055,6412	10	4,1	528,8301	2	42,3			0							25	F25:2560	444	453		
20	10	Q12805	Y.ILTPENR.C	Y	32,37	841,4658	7	5,3	421,7424	2	25,9	1,46E+06									3	F3:2094	370	376		
20	10	Q12805	N.TINTFR.I	Y	31,25	750,4024	6	8,8	751,4163	1	26,2	1,12E+06									3	F3:2122	422	427		
20	10	Q12805	K.SLSGPR.E	Y	31,24	615,334	6	5,2	616,3445	1	19,1	2,91E+05									2	F2:1527	454	459		
20	10	Q12805	R.EHIVDLEM(+15.99)LTVSSIGTFR.T	Y	26,15	2062,0405	18	6,7	688,3587	3	35,9	6,86E+05									3	F3:2929	460	477	Oxidation (M)	M8:Oxidation (M):1000.00
8	2	Q16610	R.NVALVSGDTENAK.G	Y	100,8	1316,6572	13	5,1	659,3392	2	30,6	8,11E+07	7,12E+07	5,11E+07	9,61E+07	3,91E+07	3,43E+07	1,04E+08	3,64E+07	6,01E+07	81	F81:1732	506	518		
8	2	Q16610	K.GQGEQGSTGGTNISSTSEPKEE	Y	100	2178,9512	22	6,4	1090,4899	2	28,8		1,61E+05	3,00E+05		1,50E+06	2,19E+06		2,04E+06	5,36E+04	42	F42:1582	519	540		
8	2	Q16610	R.QHVVYGPWNLPQSSYSHLTR.Q	Y	86,14	2368,1711	20	5,9	1185,0999	2	35,3	2,47E+07	1,57E+07		9,79E+07	8,33E+07	4,63E+07	1,29E+08	6,94E+07	8,63E+07	54	F54:2122	186	205		
8	2	Q16610	R.QGETLNFLEIGYSR.C	Y	85,65	1625,8049	14	5,4	813,9141	2	38	4,72E+07	3,87E+07	1,17E+07	5,98E+07	3,03E+07	9,96E+06	5,14E+07	3,35E+07	1,02E+07	30	F30:2357	206	219		
8	2	Q16610	R.ELLALIQLER.E	Y	79,91	1196,7129	10	6,9	599,3679	2	35,5	2,15E+07	1,62E+07	7,25E+06	2,49E+07	1,19E+07	8,38E+06	2,51E+07	9,76E+06	6,69E+06	11	F11:2884	334	343		
8	2	Q16610	R.Q(+.98)HVVYGPWNLPQSSYSHLTR.Q	Y	77,54	2369,1553	20	-0,2	790,7255	3	32,8	2,20E+07	7,63E+06	3,02E+06							5	F5:2894	186	205		Q1:Deamidation (NQ):98.53
8	2	Q16610	K.ELPSLQHPNEQK.E	Y	76,04	1418,7153	12	2,4	710,3666	2	30,6	4,87E+07	4,21E+07	3,01E+07	6,15E+07	3,76E+07	2,88E+07	5,67E+07	3,52E+07	2,70E+07	17	F17:1704	116	127		
8	2	Q16610	W.NLPQSSYSHLTR.Q	Y	75,77	1401,7001	12	4,9	701,8607	2	30,7	1,06E+07	3,64E+06	1,21E+07	2,57E+07	1,46E+07	1,85E+07	1,00E+07	1,18E+07	2,44E+07	16	F16:1728	194	205		
8	2	Q16610	R.NLPATDPLQR.E	Y	73,99	1123,5985	10	5,7	562,8098	2	29,3	1,01E+08	1,00E+08	7,74E+07	4,33E+07	1,70E+04	4,64E+06	3,88E+07		2,25E+07	2	F2:2389	324	333		
8	2	Q16610	A.ASEGGFTATGQR.Q	Y	73,52	1180,5472	12	7,3	591,2852	2	25,4	2,73E+06	2,48E+06	1,68E+06							2	F2:2049	20	31		
8	2	Q16610	R.APYPNYDR.D	Y	73,48	994,4508	8	-0,8	498,2323	2	26,6	7,25E+06	1,37E+07	6,02E+06	4,17E+06			3,89E+06		3,59E+06	24	F24:2249	400	407		
8	2	Q16610	R.QHVVYGPWNLPQSSYSH.L	Y	72,98	1997,938	17	6,9	999,9833	2	35				4,65E+06	2,34E+06		6,30E+06	1,72E+06	1,97E+06	30	F30:210	186	202		

						4																8			
8	2	Q16610	R.SQGGWGHR.L	Y	72,54	883,4049	8	-0,4	442,7096	2	13,2	7,89E+06	3,37E+06	3,78E+06							24	F24:993	156	163	
8	2	Q16610	K.LVWEEAMSR.F	Y	72,18	1119,5382	9	1,6	560,7773	2	32,2	1,14E+07	2,50E+06	0	6,59E+06			3,82E+06			13	F13:2768	235	243	
8	2	Q16610	K.EVGPPLPQEAVPLQK.E	Y	71,85	1600,8824	15	7,2	801,4543	2	34,8	1,99E+07	1,47E+07	4,62E+06	3,27E+07	2,09E+07	5,32E+06	3,45E+07	2,27E+07	3,41E+06	7	F7:2119	101	115	
8	2	Q16610	R.QHVVYGPWNLPQSSY.S	Y	71,11	1773,8474	15	5,6	887,9359	2	36,9		4,01E+06	1,09E+06	1,42E+07	5,75E+06	6,20E+06	1,72E+07	8,26E+06	8,35E+06	64	F64:2267	186	200	
8	2	Q16610	E.LPFPPGVPTLDNIK.N	Y	70,51	1506,8446	14	2,6	754,4315	2	38,2	1,55E+06	2,89E+06								1	F1:3210	297	310	
8	2	Q16610	E.LPSLQHPNEQK.E	Y	70,1	1289,6727	11	1,4	645,8445	2	29,3	4,16E+06	3,84E+06								1	F1:2391	117	127	
8	2	Q16610	L.PQSSYSHLTR.Q	Y	67,83	1174,5731	10	6,2	588,2975	2	30,7	1,47E+06	1,66E+06	5,81E+06	2,21E+07	1,73E+07	1,48E+07	2,23E+07	1,81E+07	1,63E+07	63	F63:1758	196	205	
8	2	Q16610	R.RAPYPNYDR.D	Y	67,73	1150,5519	9	-0,1	576,2831	2	25,6	7,00E+06	1,57E+07	1,11E+07	3,28E+07	2,44E+07	4,07E+07	5,42E+07	4,09E+07	3,49E+07	13	F13:2124	399	407	
8	2	Q16610	R.HPPSPTR.D	Y	65,73	790,4086	7	-0,4	396,2114	2	11,4	8,88E+06	5,25E+06	5,18E+06							24	F24:828	386	392	
8	2	Q16610	A.SEGGFTATGQR.Q	Y	65,4	1109,5101	11	8,7	555,7672	2	24,7	1,27E+06	1,66E+06	7,45E+05							20	F20:1975	21	31	
8	2	Q16610	R.SLPMDHPDSSQHGPPFE.G	Y	64,08	1876,8049	17	4,2	939,4137	2	32,3	2,39E+06	6,42E+06								10	F10:2674	54	70	
8	2	Q16610	L.PSLQHPNEQK.E	Y	62,86	1176,5887	10	5,4	589,3048	2	30,5	2,48E+06	3,54E+06	3,11E+06	1,10E+07	1,17E+07	5,21E+06	1,06E+07	1,18E+07	3,26E+06	38	F38:1733	118	127	
8	2	Q16610	D.ISSGLELPFPPGVPTLDNIK.N	Y	62,79	2093,1409	20	9,4	1047,5875	2	41	5,68E+05	1,04E+06	2,17E+05		3,93E+04			1,60E+05	6,74E+05	26	F26:2590	291	310	
8	2	Q16610	R.DILTIDIGR.V	Y	62,24	1014,5709	9	5	508,2953	2	36,7	3,10E+06	3,54E+06		2,30E+06	6,24E+05		2,00E+06			55	F55:2261	408	416	
8	2	Q16610	L.PATDPLQR.E	Y	62,02	896,4716	8	1,2	449,2436	2	33,2	2,07E+07	1,25E+07	6,50E+06	7,58E+07	3,36E+07	3,17E+07	6,68E+07	2,82E+07	4,33E+07	75	F75:1813	326	333	
8	2	Q16610	R.APYPNYDRDILTIDIGR.V	Y	61,17	1991,0112	17	0,1	996,513	2	34,4	0	0	2,93E+06							13	F13:2962	400	416	
8	2	Q16610	K.EVGPPLPQ(+.98)EAVPLQK.E	Y	59,77	1601,8665	15	6,3	801,9456	2	35,6			3,87E+06			1,07E+07			1,28E+07	74	F74:2142	101	115	Q8:Deamidation (NQ):59.68
8	2	Q16610	R.NVALVSGDTEN(+.98)AK.G	Y	59,32	1317,6412	13	8,7	659,8336	2	30,8							1,63E+06		4,49E+05	55	F55:1753	506	518	N11:Deamidation (NQ):100.45
8	2	Q16610	E.LLALIQLER.E	Y	58,93	1067,6703	9	3,1	534,8441	2	37,4	1,22E+06	1,59E+06								1	F1:3147	335	343	
8	2	Q16610	F.PPGVPTLDNIK.N	Y	58,48	1149,6393	11	4,5	575,8295	2	38,1	1,04E+06	2,14E+06	1,81E+06	1,16E+06	3,75E+06	6,88E+06	1,64E+06	1,81E+06	4,14E+06	74	F74:2357	300	310	
8	2	Q16610	K.AWEDTLDK.Y	Y	57,58	976,4501	8	-0,1	977,4573	1	29,6	0	0								15	F15:2574	361	368	
8	2	Q16610	R.NLPATDPLQRE.L	Y	57,45	1252,6411	11	2,9	627,3297	2	31,5	3,61E+06	2,19E+06	3,31E+06							19	F19:2692	324	334	
8	2	Q16610	E.EAPQPHYQLR.A	Y	55,74	1237,6204	10	2	619,8187	2	28,7	7,22E+06	8,59E+06	7,54E+05							10	F10:2330	273	282	
8	2	Q16610	R.NVALVSGDTE.N	Y	55,16	1003,482	10	2,7	1004,492	1	30,		2,10E+06								10	F10:254	506	515	

						2			2		9											5				
8	2	Q16610	K.LLPAQLPAEK.E	Y	53,18	1078,6385	10	7,6	540,3306	2	30,1	1,28E+07	1,45E+07	9,86E+06							2	F2:2458	91	100		
8	2	Q16610	A.APP(+15.99)SPPLSR.S	Y	52,41	936,5028	9	2,1	469,2596	2	27,9	2,99E+06	3,08E+06								10	F10:2257	45	53		P3:Oxidation or Hydroxylation:26.02
8	2	Q16610	E.GGFTATGQR.Q	Y	52,07	893,4355	9	3,7	447,7267	2	26,9	2,13E+05									1	F1:2160	23	31		
8	2	Q16610	N.LPATDPLQR.E	Y	51,37	1009,5556	9	6,1	505,7881	2	32,7				3,55E+05		1,50E+05	7,48E+05		1,58E+06	58	F58:1893	325	333		
8	2	Q16610	S.LQHPNEQK.E	Y	50,83	992,5039	8	-2,2	497,2581	2	11,7	0	0	0							15	F15:855	120	127		
8	2	Q16610	Y.GPWNLPQSSYSHLTR.Q	Y	50,24	1741,8536	15	7,1	871,9403	2	34,8						2,28E+05				51	F51:2048	191	205		
8	2	Q16610	L.PAQLPAEK.E	Y	47,85	852,4705	8	5,8	427,245	2	33,2				2,77E+06					1,43E+05	34	F34:1957	93	100		
8	2	Q16610	Q.SSYSHLTR.Q	Y	47,8	949,4617	8	-0,6	475,7379	2	19,4	1,13E+06	3,52E+05	8,02E+05						0	4	F4:1607	198	205		
8	2	Q16610	R.APYPNYDRD.I	Y	47,56	1109,4778	9	2,4	555,7475	2	28,5		5,75E+05								10	F10:2311	400	408		
8	2	Q16610	K.TRPHWCCT(-2.02)R.Q	Y	42,39	1156,5018	9	-0,5	579,2579	2	24,7	5,97E+06	6,05E+06	1,59E+06	1,00E+05	5,93E+04	1,18E+05	6,05E+04			13	F13:2042	253	261		T8:2-amino-3-oxo-butanoic_acid:59.53
8	2	Q16610	R.EYAVK.T	Y	41,78	608,317	5	-1,2	609,3235	1	14	1,22E+06	2,24E+06	7,21E+05							24	F24:1066	373	377		
8	2	Q16610	S.EGGFTATGQR.Q	Y	41,39	1022,4781	10	7,8	512,2503	2	24,4			6,62E+05							20	F20:1952	22	31		
8	2	Q16610	E.APQPHYQLR.A	Y	40,63	1108,5778	9	2,3	555,2974	2	28,6	1,97E+05									1	F1:2322	274	282		
8	2	Q16610	R.NVALVSGDTENAKG.Q	Y	40,18	1373,6786	14	-3,5	687,8442	2	27,9			0							23	F23:2396	506	519		
8	2	Q16610	N.LPQSSYSHLTR.Q	Y	39,84	1287,6571	11	6,1	644,8398	2	30,8			0							27	F27:1759	195	205		
8	2	Q16610	R.QHVVYGP(-30.01)W(+13.98)NLPQSSYSHLTR.Q	Y	39,41	2352,1399	20	0,8	785,0545	3	33,8		0	9,15E+04							22	F22:2928	186	205	Tryptophan oxidation to oxolactone	P7:Proline oxidation to pyrrolidinone:28.70; W8:Tryptophan oxidation to oxolactone:1000.00
8	2	Q16610	G.PPLPQEAVPLQK.E	Y	39,01	1315,7499	12	5,5	658,8859	2	35,2								1,10E+06		66	F66:2126	104	115		
8	2	Q16610	K.ELPSLQHPN(+.98)EQK.E	Y	38,4	1419,6993	12	8,3	710,8629	2	31,2						1,16E+05			2,23E+05	77	F77:1785	116	127		N9:Deamidation (NQ):28.64
8	2	Q16610	R.RNIWR.D	Y	37,94	743,4191	5	0,3	372,7169	2	19,5	8,81E+04	7,28E+04	3,50E+04							23	F23:1591	477	481		
8	2	Q16610	R.SQGGWG.H	Y	37,7	590,2449	6	-0,5	591,2518	1	20,4	0	3,03E+05	9,56E+04							24	F24:1665	156	161		
8	2	Q16610	D.ILTIDIGR.V	Y	37,03	899,544	8	3,1	450,7807	2	34,	5,62E+05									1	F1:2920	409	416		

											9															
8	2	Q16610	K.ELPSLQHPNEQ(+.98)K.E	Y	36,65	1419,6993	12	5,8	710,8611	2	31,1						0				50	F50:1747	116	127		Q11:Deamidation (NQ):18.53
8	2	Q16610	K.TRPHW(+13.98)CCTR.Q	Y	36,52	1172,4968	9	1,1	587,2563	2	27,2				3,74E+05	4,08E+05		5,11E+05			59	F59:1479	253	261	Tryptophan oxidation to oxolactone	W5:Tryptophan oxidation to oxolactone:1000.00
8	2	Q16610	K.TRPHW(+15.99)CCT(-2.02)R.Q	Y	36,47	1172,4968	9	2,3	587,257	2	27,7		1,22E+04		1,84E+05			6,93E+04	2,37E+05		65	F65:1530	253	261		W5:Oxidation (HW):27.96;T8:2-amino-3-oxo-butanoic_acid:59.53
8	2	Q16610	R.QLRPEHFQEVGYAAPP(+15.99)SPPLSR.S	Y	35,03	2491,2607	22	9,1	623,8281	4	30,1	4,41E+06	2,15E+06								3	F3:2457	32	53		P16:Oxidation or Hydroxylation:10.11
8	2	Q16610	K.TRPHWC(-33.99)C(+31.99)TR.Q	Y	34,99	1156,5197	9	-9,9	579,2614	2	28,5		1,54E+06	1,97E+06							27	F27:1583	253	261		C6:Dehydroalanine (C):12.28;C7:Dihydroxy:12.28
8	2	Q16610	K.TRPHWC(+31.99)C(-33.99)TR.Q	Y	34,81	1156,5197	9	-10	579,2612	2	28,1	2,40E+06					1,18E+05				7	F7:1562	253	261		C6:Dihydroxy:0.00;C7:Dehydroalanine (C):0.00
8	2	Q16610	A.K(+43.01)(+31.99)GQGEQGSTGGTNISSTSEPKEE	Y	34,77	2382,0417	23	7,8	1192,0375	2	29,2						9,11E+04		1,14E+05		67	F67:1628	518	540		K1:Carbamylation:104.22;K1:Dihydroxy:106.58
8	2	Q16610	V.WEEAMSR.F	Y	32,3	907,3858	7	5,8	454,7028	2	34,4	1,87E+06									7	F7:2080	237	243		
8	2	Q16610	K.LVWEE.A	Y	32,11	674,3275	5	2,4	675,3364	1	33,6	3,59E+06	4,57E+06								1	F1:2805	235	239		
8	2	Q16610	R.QH(-23.02)VVY(+33.96)GP(-27.99)WNLPQSSYSHLTR.Q	Y	31,94	2351,1213	20	1,3	1176,5695	2	33,9	0	8,64E+05								15	F15:2974	186	205		H2:his2asn:93.20;Y5:Chlorination of tyrosine residues:102.98;P7:Pyrrolidone from Proline:82.63
8	2	Q16610	Y.AAPP(+15.99)SPPLSR.S	Y	30,4	1007,5399	10	1,9	504,7782	2	28,3		1,84E+06								10	F10:2288	44	53		P4:Oxidation or Hydroxylation:17.01
8	2	Q16610	R.APYPN(+.98)YDRDILTIDIGR.V	Y	30,23	1991,9952	17	7,3	665,0106	3	34,1			0							23	F23:2995	400	416	Deamidation (NQ)	N5:Deamidation (NQ):1000.00
8	2	Q16610	R.Q(+43.01)HVVYGP(-30.01)WNLP(-30.01)QSSYSHLTR.Q	Y	29,11	2351,156	20	4,9	1176,5911	2	36,8						1,28E+07		2,14E+06		53	F53:2244	186	205	Carbamylation; Proline oxidation to pyrrolidinone	Q1:Carbamylation:1000.00;P7:Proline oxidation to pyrrolidinone:1000.00;P11:Proline oxidation to pyrrolidino

																					ne:1000.00
8	2	Q16610	K.TRPHW(+15.99)C(+31.99)C(-33.99)TR.Q	Y	28,63	1172,5145	9	-9,7	587,2589	2	27,4				1,37E+05	63	F63:1514	253	261		W5:Oxidation (HW):26.02;C6:Dihydroxy:0.00; C7:Dehydroalanine (C):0.00
8	2	Q16610	R.Q(+.98)HVVY(-2.02)GPWNLPQSSYSHLTR.Q	Y	27,38	2367,1396	20	7	790,0593	3	32,9		0			12	F12:2678	186	205		Q1:Deamidation (NQ):4.32; Y5:2-amino-3-oxo-butanoic_acid:59.08
8	2	Q16610	L.IQLER.E	Y	26,48	657,381	5	-1,1	329,6974	2	25,2			8,37E+05		23	F23:2128	339	343		
8	2	Q16610	A.LIQLER.E	Y	26,46	770,465	6	1,2	386,2403	2	28,5		0	0		15	F15:2466	338	343		
5	40	Q8WZ42	K.SPEPR.V	N	30,49	584,2918	5	1,9	585,3002	1	29,3	4,43E+05				1	F1:2386	33595	33599		
5	40	Q8WZ42	K.VGDTLR.L	N	29,37	659,3602	6	3,7	660,3699	1	24,8			3,26E+05		19	F19:2003	18159	18164		
5	40	Q8WZ42	L.SWSRPK(-1.03)DDGGSR(+15.99)VTGYYIER(+15.99).K	Y	27,31	2359,0828	20	3,5	1180,5527	2	32,2		2,52E+06			18	F18:1844	31276	31295	Lysine oxidation to aminoadipic semialdehyde	K6:Lysine oxidation to aminoadipic semialdehyde:1000.00;R12:Oxidation or Hydroxylation:2.87;R20:Oxidation or Hydroxylation:22.45
5	40	Q8WZ42	D.TTETKEVVK.L	Y	26,94	1033,5656	9	6,2	517,7933	2	21,2			1,85E+05		21	F21:1679	1970	1978		
5	40	Q8WZ42	K.K(+43.01)(+14.96)PEAP(+13.98)PPKVPEAPK.E	Y	25,92	1555,7881	14	-1,7	519,6024	3	29,1			0		22	F22:2485	11444	11457		K1:Carbamylation:79.54;K1:Alpha-amino adipic acid:79.54; P5:Proline oxidation to pyroglutamic acid:12.33
47	59	Q9BQS7	R.HLGILGPVIR.A	Y	53,99	1073,6709	10	2	537,8438	2	34,5			6,99E+06		19	F19:2989	452	461		
71	65	Q9H8M2	K.ILHAGFK.M	Y	45,51	784,4595	7	2,7	785,4689	1	29,3			3,13E+07		19	F19:2468	229	235		
71	65	Q9H8M2	K.ILHAGF.K	Y	32,59	656,3646	6	5,4	329,1913	2	29			1,91E+06		20	F20:2383	229	234		
